# The CgHaa1-Regulon Mediates Response and Tolerance to Acetic Acid Stress in the Human Pathogen *Candida glabrata*

**DOI:** 10.1534/g3.116.034660

**Published:** 2016-11-04

**Authors:** Ruben T. Bernardo, Diana V. Cunha, Can Wang, Leonel Pereira, Sónia Silva, Sara B. Salazar, Markus S. Schröder, Michiyo Okamoto, Azusa Takahashi-Nakaguchi, Hiroji Chibana, Toshihiro Aoyama, Isabel Sá-Correia, Joana Azeredo, Geraldine Butler, Nuno Pereira Mira

**Affiliations:** * Department of Bioengineering, Institute of Bioengineering and Biosciences, Instituto Superior Técnico, Universidade de Lisboa, 1049-001, Portugal; † School of Biomolecular and Biomedical Sciences, Conway Institute, University College of Dublin, Belfield, Dublin 4, Ireland; ‡ Centre of Biological Engineering, University of Minho, 4710-057 Braga, Portugal; § Medical Mycology Research Center, Chiba University, Chuo-ku, 260-8673, Japan; ** Department of Electronic and Information Engineering, Suzuka National College of Technology, Unver, Mie 510-0294, Japan

**Keywords:** CgHaa1, acetic acid, *Candida glabrata*, vaginal dysbiosis, vaginal candidiasis

## Abstract

To thrive in the acidic vaginal tract, *Candida glabrata* has to cope with high concentrations of acetic acid. The mechanisms underlying *C. glabrata* tolerance to acetic acid at low pH remain largely uncharacterized. In this work, the essential role of the CgHaa1 transcription factor (encoded by ORF CAGL0L09339g) in the response and tolerance of *C. glabrata* to acetic acid is demonstrated. Transcriptomic analysis showed that CgHaa1 regulates, directly or indirectly, the expression of about 75% of the genes activated under acetic acid stress. CgHaa1-activated targets are involved in multiple physiological functions including membrane transport, metabolism of carbohydrates and amino acids, regulation of the activity of the plasma membrane H^+^-ATPase, and adhesion. Under acetic acid stress, CgHaa1 increased the activity and the expression of the CgPma1 proton pump and contributed to increased colonization of vaginal epithelial cells by *C. glabrata*. *CgHAA1*, and two identified CgHaa1-activated targets, CgTPO3 and CgHSP30, are herein demonstrated to be determinants of *C. glabrata* tolerance to acetic acid. The protective effect of CgTpo3 and of CgHaa1 was linked to a role of these proteins in reducing the accumulation of acetic acid inside *C. glabrata* cells. In response to acetic acid stress, marked differences were found in the regulons controlled by CgHaa1 and by its *S. cerevisiae* ScHaa1 ortholog, demonstrating a clear divergent evolution of the two regulatory networks. The results gathered in this study significantly advance the understanding of the molecular mechanisms underlying the success of *C. glabrata* as a vaginal colonizer.

*Candida glabrata* is commonly found as a commensal in the microflora that colonizes the human gastrointestinal and genitourinary tracts. Under certain conditions, such as reduced activity of the host immune system, *C. glabrata* colonization can result in infections ranging from mucocutaneous candidiasis to life-threatening disseminated mycosis, where the yeasts cross the bloodstream and colonize major internal organs (Lim *et al.* 2012). Vaginal candidiasis is the most common form of superficial candidiasis, with 75% of all women being estimated to suffer from this infection, a substantial percentage, in a recurrent manner (Fidel *et al.* 1999; Sobel *et al.* 1998). Although *C. albicans* is the most prevalent causative agent of vulvovaginal and invasive candidiasis, the proportion of infections caused by *C. glabrata* is increasing, in many cases already surpassing the levels reported for *C. albicans* (Krcmery and Barnes 2002; Falagas *et al.* 2010; Tortorano *et al.* 2006; Richter *et al.* 2005; Sobel *et al.* 1998; Zhang *et al.* 2014).

To succeed in colonization of the vaginal tract, *C. glabrata* cells have to cope with multiple environmental challenges, including the activity of the immune system, the panoply of nutrients available, alterations in extracellular pH (which varies during the menstrual cycle), and the presence of a cocolonizing microbiota, among others. Metagenomic analyses undertaken with different female populations revealed that the vaginal microbiota is essentially composed of lactic acid bacteria, although differences in species and in their rank-abundance had been found (Ravel *et al.* 2011; Zhou *et al.* 2010). This observation suggests that one key ecological function of the vaginal microbiota is the production of organic acids that restrain the overgrowth of fungal and/or bacterial pathogens (Hickey *et al.* 2012; O’Hanlon *et al.* 2013; Parolin *et al.* 2015). Other interference effects attributed to vaginal lactic acid bacteria include competition for nutrients and adhesion sites, and the secretion of H_2_O_2_, bacteriocine-like substances, and biosurfactants (Morales and Hogan 2010). Consistently, the use of broad-spectrum antibiotics, which reduce commensal microflora abundance, is a recognized risk factor for the development of candidiasis (Tortorano *et al.* 2006; Sobel *et al.* 1998).

Like lactic acid, acetic acid is also found in the vaginal fluid, the production of these organic acids being thought to result from bacterial metabolic activity (Owen and Katz 1999). Consistently, the concentration of acetic acid in the vaginal tract is particularly high when an overgrowth of anaerobic bacteria occurs, a condition known as bacterial vaginosis (Chaudry *et al.* 2004). In conditions of eubiosis, the amount of acetic acid is estimated to range between 1 and 4 mM, while in conditions of dysbiosis, the concentration can increase to > 100 mM (Chaudry *et al.* 2004). In the acidic environment of the vaginal tract (pH ∼3.5–4.2) (O’Hanlon *et al.* 2013; Owen and Katz 1999) acetic acid (pKa 4.7) will be predominately in its undissociated form (RCOOH), which has a well described antifungal effect (Piper *et al.* 2001; Mira *et al.* 2010c; Trckek *et al.* 2015). Undissociated acetic acid molecules can permeate the microbial plasma membrane simply by passive diffusion, dissociating in the near-neutral cytosol, and leading to the accumulation of protons and acetate. The internal acidification and the accumulation of acetate has been shown to cause multiple deleterious effects in yeast cells, including increase in turgor pressure, oxidative stress, reduced activity of metabolic enzymes, and dissipation of the electrochemical gradient maintained across the plasma membrane, an essential feature for secondary transport (Mira *et al.* 2010c; Mollapour *et al.* 2008; Piper *et al.* 2001; Trckek *et al.* 2015)

To thrive in the vaginal environment, it is likely that *C. glabrata* cells have evolved dedicated mechanisms to cope with stress induced by the presence of acetic acid at low pH. Indeed, it has been shown that the vaginal strain *C. glabrata* BG2 is more tolerant to acetic acid than the CBS138 strain, which has an intestinal origin (Gregori *et al.* 2007). Little is known about the key players mediating response and tolerance to acetic acid stress in *C. glabrata*, contrasting with the extensive knowledge that has already been gathered in the experimental model yeast *Saccharomyces cerevisiae* and, to a lesser extent, in *C. albicans* (Mira *et al.* 2010c; Mollapour *et al.* 2008; Piper *et al.* 2001; Cottier *et al.* 2015a). Besides passive diffusion, acetic acid was also found to enter *S. cerevisiae* cells through the aquaglyceroporin Fps1 (Mollapour and Piper 2007). Prominent intracellular acidification was found to occur upon exposure of unadapted *S. cerevisiae* cells to an inhibitory concentration of acetic acid, this being counteracted by the activity of two proton pumps, the plasma membrane H^+^-ATPase Pma1 and the vacuolar ATPase, which catalyze the extrusion of the exceeding protons from the cytosol to the cell exterior or to the vacuole lumen, respectively (Ullah *et al.* 2013; Mira *et al.* 2010c; Carmelo *et al.* 1997). *C. glabrata* cells challenged with inhibitory concentrations of acetic acid also experience a reduction in internal pH (pH_i_); however, this drop is much more subtle than the one observed in *S. cerevisiae* (Ullah *et al.* 2013). Due to its negative charge, acetate cannot cross the plasma membrane lipid bilayer and therefore its extrusion depends on the activity of inducible transporters. In *S. cerevisiae*, the expression of the multidrug resistance transporters of the Major Facilitator Superfamily Tpo2, Tpo3, and Aqr1 was found to confer protection against acetic acid (Fernandes *et al.* 2005; Tenreiro *et al.* 2002). Recently, CgAqr1, an ortholog of ScAqr1, was shown to mediate *C. glabrata* tolerance to acetic acid (Costa *et al.* 2013). However, this protective effect exerted by CgAqr1 could not be linked to an effect of this pump in reducing the internal accumulation of acetic acid inside *C. glabrata* cells (Costa *et al.* 2013).

mRNA profiling of acetic acid-stressed *S. cerevisiae* cells revealed that the transcriptional response to acetic acid is largely controlled by the transcription factor Haa1, which was found to regulate, directly or indirectly, the transcription level of around 80% of the acetic acid-activated genes (Mira *et al.* 2010a, 2011). The expression of the *HAA1* gene and of several Haa1 target genes decreased *S. cerevisiae* susceptibility to acetic acid. These genes included the drug efflux pumps Tpo2, Tpo3, and Aqr1; the protein kinase Hrk1; and Sap30, a subunit of a histone deacetylase complex (Fernandes *et al.* 2005; Mira *et al.* 2010a). In *C. albicans*, response and tolerance to acetic acid was found to be largely dependent on the transcription factor Mnl1, which controlled a substantial percentage of the genes activated by the acid (Ramsdale *et al.* 2008). More recently, it was also shown that the glucose-regulated transcription factor Mig1 plays an important role in providing protection against the toxic effect exerted by acetic acid and other organic acids in acidic environments (Cottier *et al.* 2015b). No significant homolog of ScHaa1 was found in *C. albicans*, but in *C. glabrata* the ORF CAGL0L09339g encodes a protein with similarity to ScHaa1, particularly in the N-terminal region. In this work, we carried out a detailed functional analysis of this putative *C. glabrata* gene, demonstrating that it is indeed a functional Haa1 homolog that plays a crucial role in the tolerance and response of this pathogenic yeast to acetic acid stress.

## Materials and Methods

### Strains and growth media

The set of *C. glabrata* strains used in this work are listed in Table 1. The different strains were batch-cultured at 30° in liquid minimal medium (MM) or in RPMI growth medium with orbital agitation (250 rpm). MM contains, per L, 20 g glucose (Merck), 1.7 g yeast nitrogen base with amino acids (Difco), and 2.65 g (NH_4_)_2_SO_4_ (Merck). The RPMI growth medium contains, per L, 20 g of RPMI medium powder without glutamine (Sigma, St. Louis, MO), 20 g of glucose (Merck), and 0.3 g of glutamine (Sigma). Whenever needed, the MM and the RPMI growth medium were adjusted to pH 4 using HCl as the acidulant. Cell viability was assessed in Yeast Peptone Dextrose (YPD) growth medium which contains, per L, 20 g of glucose (Merck), 20 g bactopeptone (Difco), and 10 g yeast extract (Difco). Solid media was obtained supplementing the corresponding liquid growth medium with 2% agar (Iberagar). 

**Table 1:** List of strains used in this work

Strain	Parent	Description	Reference
KUE100	2001H	Parent strain, histidine auxotroph, the recipient enable high efficient gene targeting in which *yku80* is repressed with a *SAT1* flipper	Ueno *et al.* (2011)
KUE100*_*Δ*CgBag7*	KUE100	Δ*CgBag7* strain, *CgBAG7* gene (ORF CAGL0I07249g) was replaced with *CgHIS3* marker	This study
KUE100*_*Δ*CgCmr3*	KUE100	Δ*CgCmr3* strain, *CgCMR3* gene (ORF CAGL0L05786g) was replaced with *CgHIS3* marker	This study
*KUE100_*Δ*CgEno2*	KUE100	Δ*CgEno2* strain, CgENO2 gene (ORF CAGL0F08261g) was replaced with *CgHIS3* marker	This study
KUE100*_*Δ*CgFps1*	KUE100	Δ*CgFps1* strain, *CgFPS1* gene (ORF CAGL0C03267g) was replaced with *CgHIS3* marker	This study
*KUE100_*Δ*CgFps2*	KUE100	Δ*CgFps2* strain, *CgFPS2* (Acs# CAGL0E03894g) was replaced with *CgHIS3* marker	This study
KUE100*_*Δ*CgGad1*	KUE100	Δ*CgGad1* strain, *CgGAD1* gene (ORF CAGL0H02585g) was replaced with *CgHIS3* marker	This study
KUE100*_*Δ*CgHaa1*	KUE100	Δ*CgHaa1* strain, *CgHAA1* gene (ORF CAGL0L09339g) was replaced with *CgHIS3* marker	This study
*KUE100_*Δ*CgHsp30*	KUE100	Δ*CgHsp30* strain, *CgHSP30* gene (ORF CAGL0K07337g) was replaced with *CgHIS3* marker	This study
KUE100*_*Δ*CgMit1*	KUE100	Δ*CgMit1* strain, *CgMIT1* gene (ORF CAGL0C03740g) was replaced with *CgHIS3* marker	This study
KUE100*_*Δ*CgRsb1*	KUE100	Δ*CgRsb1* strain, *CgRSB1* gene (ORF CAGL0L10142g) was replaced with *CgHIS3* marker	This study
KUE100_ΔCg*Ssa3*	KUE100	ΔCgSsa3 strain, *CgSSA3* gene (ORF CAGL0G03289g) was replaced with *CgHIS3* marker	This study
KUE100*_*Δ*CgSut2*	KUE100	Δ*CgSut2* strain, *CgSUT2* gene (ORF CAGL0I04246g) was replaced with *CgHIS3* marker	This study
KUE100*_*Δ*CgTpo3*	KUE100	Δ*CgTpo3* strain, *CgTPO3* gene (ORF CAGL0I10384g) was replaced with *CgHIS3* marker	Costa *et al.* (2014)
*KUE100_*Δ*CgYps4*	KUE100	Δ*CgYps4* strain, *CgYPS4* gene (ORF CAGL0E01749g) was replaced with *CgHIS3* marker	This study
KUE100*_*Δ*CAGL0E03740g*	KUE100	Δ*CAGL0E03740g* strain, ORF CAGL0E03740g was replaced with *CgHIS3* marker	This study
KUE100*_*Δ*CAGL0G05632g*	KUE100	Δ*CAGL0G05632g* strain, ORF CAGL0G05632g was replaced with *CgHIS3* marker	This study
ATCC2011	ATCC2001	Parent strain	—
ATCC2001*_* Δ*CgHaa1*	ATCC2001	Δ*CgHaa1* strain, *CgHAA1* (ORF CAGL0L09339g) was replaced by the norseotruchin resistance *SAT1* flipper cassette	Scwarzmuller *et al.* (2014)

### Gene disruption

The KUE100 strain (Ueno *et al.* 2011) was used as the host for the individual disruption of *CgHAA1* or of the selected CgHaa1 target genes. To create a mutant devoid of *CgHAA1*, this gene was replaced by a DNA cassette containing the *CgHIS3* gene using homologous recombination. The replacement cassette was prepared by PCR using an appropriate set of primers (sequences available upon request). The pHIS906 plasmid containing the *CgHIS3* sequence was used as a template and the transformation procedures of KUE100 cells were performed as described before (Ueno *et al.* 2011). The recombination locus and gene deletion were verified by PCR using appropriate primers (sequences available upon request). The same methodology was used to disrupt the CgHaa1-regulated genes *CgGAD1*, *CgTPO3*, *CgCAGL0G05632g*, *CAGL0G07249g*, *CAGL0I04246g*, *CgRSB1*, *CgFPS1*, and *CgFPS2*.

### Susceptibility assays

The comparison of the susceptibility of *C. glabrata* KUE100 and of the derived deletion mutant Δ*Cghaa1* to organic acids (acetic, propionic, lactic, and benzoic acids), azoles, NaCl, H_2_O_2_, and to heat stress was first based on spot assays. Midexponential KUE100 or Δ*Cghaa1* cells (OD_600nm_ 0.5 ± 0.05) cultivated in liquid MM medium (at pH 4.0) were diluted in water to a standardized OD_600nm_ of 0.05 ± 0.005. Cell suspensions and subsequent dilutions (1:5 and 1:25) were applied as spots (of 4 µl) onto the surface of agarized MM plates or, in this same growth medium, supplemented with inhibitory concentrations of the different stressors. The concentrations used were the following: NaCl (0.4–0.5 M), H_2_O_2_ (0.3–0.5 mM), ketoconazole (45–55 μg/ml), fluconazole (150–175 μg/ml), clotrimazole (7.5–10 μg/ml), itraconazole (80–100 μg/ml), miconazole (0.5–0.75 μg/ml), tioconazole (0.25–0.5 μg/ml), acetic acid (30–60 mM), propionic acid (17–20 mM), lactic acid (90–120 mM), and benzoic acid (0.8–1 mM). Stock solutions of the different antifungals tested were prepared in DMSO while the stock solutions of the remaining chemicals were prepared in water. In the case of organic acids, the pH of the solid MM and of the stock solution used was adjusted to 4.5 using NaOH and HCl before the medium supplementation. A similar procedure was used to compare the susceptibility of wild-type ATCC2001 and ATCC2001_Δ*Cghaa1* cells in the presence or absence of acetic acid. However, in this case, the susceptibility of the two strains to acetic acid was compared in rich YPD growth medium.

The susceptibility to acetic acid of wild-type KUE100, of the Δ*Cghaa1* mutant, and of the deletion mutants devoid of the selected set of CgHaa1 target genes was also tested based on the comparison of the growth curve of the strains in liquid MM (at pH 4.0) supplemented with the acid (60 mM). The stock solution of acetic acid used was adjusted to pH 4 using NaOH before supplementation of the growth medium. For this, cells of the different strains were cultivated in liquid MM (at pH 4.0), harvested in midexponential phase (OD_600nm_ 0.8 ± 0.05), and then used to reinoculate (at initial OD_600nm_ of 0.05) this same basal medium either supplemented or not with acetic acid. Growth in the presence or absence of acetic acid was monitored by accompanying the increase in the OD_600nm_ of the cultures.

### Microarray experiments

*C. glabrata* KUE100 and the derived strain lacking *CgHAA1* were cultivated in MM (adjusted to pH 4.0 with HCl) until midexponential phase and then reinoculated (at initial OD_600nm_ of 0.2 ± 0.05) into this same growth medium (at pH 4) either supplemented or not with 30 mM acetic acid. Cell viability during growth of the two strains in the presence or absence of acetic acid stress was estimated based on the number of colony forming units (CFUs) on the surface of YPD plates. After 30 min of incubation in the presence or absence of acetic acid, cells were harvested by centrifugation and immediately frozen and stored at −80° until further use. Three independent cultures from each strain in the presence and absence of acetic acid were used for transcriptional profiling. RNA extraction was performed using the RiboPure RNA Isolation Kit (Ambion, Life Technologies, CA). The quality and integrity of the purified RNA was confirmed using a Bioanalyzer. The DNA chips used for this microarray analysis were manufactured by Agilent using a design for *C. glabrata* (Rossignol *et al.* 2007). cDNA synthesis, hybridization, and scanning were performed using protocols similar to those described by (Rossignol *et al.* 2007), except that hybridzation was carried out using an Agilent hybridization oven at 65° for 17 hr at 100 rpm. In brief, 24 µg of total RNA was incubated with 1.4 μg of anchored Oligo(dT)_20_ primer (Invitrogen) in a total volume of 18.5 µl for 10 min at 70°. First-strand buffer (Invitrogen); 0.5 mM dATP, dTTP, and dGTP; 50 µM dCTP; 10 mM dithiothreitol; 2 µl Superscript III reverse transcriptase (Invitrogen); and 2 µl Cy3-dCTP or Cy5-dCTP (Amersham, PA53021 and A55021) were added to a total volume of 40 µl, incubated at 42° for 2 hr, and followed by 1 hr at 42° with an additional 1 µl of Superscript III. RNA was degraded by addition of 1 µl of RNase A at 50 µg/ml and 1 µl of RNase H at 1 unit/µl and incubated at 37° for 30 min. The labeled cDNAs were purified using a QIAquick PCR purification kit (QIAGEN, Crawley, UK). Samples were prepared for hybridization using Agilent’s Two-Color Microarray-Based Gene expression (Quick Amp labeling protocol) and the Gene Expression Hybridization Kit. 20 μl each Cy3-labeled and Cy5-labeled cDNA were used per array, in a total volume of 100 μl. Hybridized microarrays were scanned with an Axon 4000B scanner (Axon Instruments) and data were acquired with GenePix Pro 5.1 software (Axon Instruments). Data were analyzed using the LIMMA package in Bioconductor (www.bioconductor.org). The expression of log-ratios was normalized so that they averaged to zero within each array using Lowess normalization and no background correction. Afterward, normalization of expression intensities was performed to assure a similar distribution across a set of arrays and a linear model was fitted for each gene. After statistical analysis, the obtained p values were corrected for multiple testing using the Benjamini–Hochberg method. Lowess normalization and background correction were applied to each array separately, and quantile normalization was used to allow log-ratios to be compared across arrays. Each gene is represented by two probes spotted in duplicate, which were used separately to calculate logFC (Supplemental Material, Table S1, Table S2, and Table S3). Only genes exhibiting log_2_FC > 1 and a p < 0.01 for at least one probe were selected for further analysis. The datasets were deposited at the Array Express Database with reference number E-MTAB-4875.

### Comparison of gene transcription levels based on real-time RT-PCR

Real-time RT-PCR was used to compare the transcript levels from *CgPMP2*, *CgPMA1*, *CgTPO3*, and *CAGL0G05632g* genes in the wild-type strain and in the deletion mutant Δ*Cghaa1*, cultivated in the presence or absence of acetic acid. For this purpose, cells were cultivated under the same experimental conditions used for the microarray analysis, and the same protocol was used to extract total RNA. 1 μg of total RNA collected from each sample was used for cDNA synthesis. The reverse transcription step was performed using the multiscribe reverse transcriptase kit (Applied Biosystems) in a C1000 Thermal Cycler (Bio-Rad, Hercules). Approximately 125 ng of the synthesized cDNA were used for the quantitative PCR step. In all experiments, the transcript level of Cg*ACT1* mRNA was used as an internal control. The primers used for the amplification of the probes selected to monitor gene expression were designed using Primer Express Software (Applied Biosystems) (sequences available upon request). The relative values obtained for the wild-type strain in control conditions were set as 1 and the remaining values are presented relative to that control.

### [1-^14^C]-acetic acid accumulation assays

The accumulation ratio (intracellular/extracellular concentration) of radiolabeled [1-^14^C]-acetic acid was compared in KUE100 and in the derived deletion mutants Δ*Cghaa1*, Δ*Cgtpo3*, and Δ*Cghsp30* during the first 30 min of incubation in the presence of cold acetic acid (60 mM, at pH 4.0). Cells of the different strains grown in MM (at pH 4.0) until midexponential phase (OD_600nm_ = 0.8 ± 0.05) were harvested by centrifugation, washed with fresh medium, and finally resuspended in 5 ml of this same medium to obtain dense cell suspensions (OD_600nm_ = 0.7 ± 0.05). This cell suspension was incubated for 5 min at 30° with agitation (150 rpm). After that time, 20 µM of labeled [1-^14^C]-acetic acid (sodium salt from GE Healthcare, Piscataway, NJ; 9.25 MBq) were added to the cell suspension along with 60 mM cold acetic acid (at pH 4.0). The intracellular accumulation of radiolabeled acetic acid was followed for 30 min by filtering, at intervals of 5 min, 200 μl of the cell suspension through prewetted glass microfiber filters (Whatman GF/C). The filters were washed with cold water. Extracellular concentration of [1-^14^C]-acetic acid was estimated by measuring the radioactivity of 100 μl of the culture supernatant, recovered by centrifugation in a tabletop centrifuge. The radioactivity was measured in a Beckman LS 5000TD scintillation counter. Nonspecific adsorption of acetic acid to the filters and to the cells was assessed and taken into consideration (< 5% of the total bound radioactivity). Intracellular concentration of [1-^14^C]-acetic acid was calculated considering the internal cell volume (V_i_) of the strains constant and equal to 2.5 μl (mg dry weight)^−1^ (Carmelo *et al.* 1997).

### Quantification of CgPma1 protein levels

The levels of CgPma1 present in the plasma membrane of KUE100 or of KEU100_Δ*Cghaa1*, either subjected or not to acetic acid stress, were quantified by western blot using a rabbit polyclonal antibody targeting CgPma1 (Santa Cruz Biotech). For this, cells of both strains were cultivated under the same experimental conditions used for the transcriptomic analysis and were harvested by centrifugation after 30 and 60 min of incubation in the absence or presence of acetic acid. The pellets obtained were resuspended in 2 ml of buffer A (100 mM Tris, 5 mM EDTA, and 2 mM DTT), rapidly frozen in liquid nitrogen, and maintained at −80° until further use. Cell suspensions were thawed at room temperature, after which a protease inhibitor cocktail (10 mg/ml leupeptine, 1 mg/ml pepstatine A, 20 mg/ml aprotinin, 1.5 mg/ml benzamidine, 2 mg/ml trypsin/quimotrypsin inhibitor, and 1 mM PMSF) was added. Protein extracts enriched in membrane proteins were obtained using a previously described method (Fernandes and Sa-Correia 1999). 20 µg of the protein extract obtained from the two strains, enriched in plasma membrane proteins, were separated in a 12.5% SDS-PAGE acrylamide gel. After separation, proteins were transferred by electroblotting to a nitrocellulose membrane using a Mini Trans-Blot Cell (Bio-Rad) at 115 Volts for 90 min. The nitrocellulose membrane was washed with PBST buffer (2.7 mM KCl, 140 mM NaCl, 1.8 mM KH_2_PO_4_, 10 mM Na_2_HPO_4_, and 0.05% Tween 20 at pH 7.4) and incubated for 1 hr with 25 ml of a 2.5%-skimmed milk solution (prepared in PBST). After this, the membrane was incubated overnight at 4° with 25 ml of PBST-skimmed milk solution supplemented with the anti-CgPma1 antibody (1:2000 proportion). On the next day, the membrane was washed three times with PBST buffer and then incubated for 2 hr with the secondary antibody, goat anti-IgG rabbit coupled with HRP (1:1250 proportion). The HRP signal was detected and further quantified using an ECL Plus Detection kit in a Fusion SOLO Chemiluminescence System (Analis, Namur, Belgium). Equal loading of total proteins was confirmed by Ponceau staining of the membranes.

### Quantification of CgPma1 activity

The activity of *C. glabrata* PM-H^+^-ATPase was compared in wild-type KUE100 cells and in the deletion mutant Δ*Cghaa1* based on the rate of ATP hydrolysis prompted by total membrane protein extracts obtained from the two strains, a methodology that has been widely used to quantify PM H^+^-ATPase activity in *C. glabrata* and in *S. cerevisiae* (Bairwa and Kaur 2011; Fernandes and Sa-Correia 1999). KUE100 and Δ*Cghaa1* cells were cultivated under the same experimental conditions as those described above for the transcriptomic analysis. Total membrane protein extracts enriched in CgPma1 were obtained using the methodology described above for the western blot experiments. 2 µg of protein extracts enriched in membrane proteins were used in each enzymatic assay. The activity of CgPma1 in each protein extract was determined in a 1 ml volume reaction containing 300 µl of MI buffer [50 mM MES (Sigma), 10 mM MgSO_4_0.7H_2_0 (Merck), 50 mM KCl (Merck), 5 mM NaN_3_ (Merck), 50 mM KNO_3_ (Merck), and 0.2 mM (NH_4_)_6_Mo_7_O_24_0.4H_2_O (Merck), pH of 5.7 adjusted with 2.5 M TRIS]. 2 µg of protein were added to the MI buffer and the mixture was left in a water bath at 30° for 5 min for temperature equilibration. After this time, the enzymatic reaction was started upon addition of 2 mM ATP and samples were taken after 1, 3, 5, 7, and 10 min. At each time point, the reaction was stopped by adding 300 µl of 10% (w/v) trichloroacetic acid (Merck). As a blank, the same procedure was performed in a sample containing MI buffer instead of protein extract. Each sample was centrifuged in a tabletop centrifuge (12,000 rpm for 3 min at room temperature) to remove the protein fraction. 500 µl of the supernatant was transferred to a new tube and the amount of inorganic phosphate present in the mixture was estimated by adding 450 µl of 0.8 M HCl containing 0.5% (w/v) (NH_4_)_6_Mo_7_O_24_0.4H_2_O (Merck) and 50 µl of Fiske-Subbarow reagent [0.25 g/L 1-amino-2-naphthol-4-sulfonic acid (Sigma), 10 g/L Na_2_SO_3_ (Merck), and 150 g/L Na_2_S_2_O_5_ (Merck)]. The sample was mixed vigorously and left to rest for 30 min at room temperature. Absorbance was measured at 700 nm. The amount of P_i_ present in each sample was calculated based on calibration curves using Na_2_HPO_4_.H_2_0 (Merck) as a standard (concentration range: 0.04–1.6 mM) and subtracting the value obtained in the blank sample.

### Infection of reconstituted human vaginal epithelium (RHVE)

To study the effect of acetic acid on the infection of vaginal epithelium by KUE100 and Δ*Cghaa1* cells, a commercially available RHVE (SkinEthic Laboratories; Nice, France) was used as an *in vitro* model of vaginal candidosis. The method used is similar to the one described before (Alves *et al.* 2014a). RHVE tissues were inoculated for 24 hr with 1 ml of standardized suspensions of the two *C. glabrata* strains in RPMI medium adjusted to pH 4 (about 2 × 10^6^ cells/ml) either supplemented or not with 30 mM acetic acid. As a control, two RHVE tissue preparations incubated only with 1 ml RPMI or RPMI and acetic acid were prepared. All the infected tissues were incubated at 37° in a 5% CO_2_ environment in saturated humidity for the respective times. After incubation, the tissue was rinsed twice in 1 ml of PBS to remove nonadherent *Candida* cells, and the tissue was then bisected, with one half being used for fluorescence microscopy analysis and the other for molecular studies. For fluorescence microscopy analysis, the tissue preparations were fixed in 2% (v/v) formalin and stored at 4° until histological processing. Tissues were then dehydrated, cleared, and infiltrated with paraffin wax embedding material. The formalin-fixed, paraffin-embedded (FFPE) tissues were stored at room temperature. The tissues were cut (5 μm sections) and placed on Histobond+-coated microscope slides (Raymond A Lamb, East Sussex, UK), dewaxed, and processed through xylene, ethanol, and water before peptide nucleic acid probe hybridization. Peptide nucleic acid probe fluorescence *in situ* hybridization (PNA FISH) was employed on tissue sections using the Light PNA FISHTM kit (AdvanDx Inc., Woburn, MA). This species-specific probe was used to study the colonization of the RHVE by *C. glabrata*. The Light PNA FISHTM kit had previously been developed and evaluated using a multicolor-labeled fluorescent PNA probe targeting specific 26S rRNA sequences of *C. glabrata* (Alves *et al.* 2014b). Tissue sections on microscope slides were overlaid with one drop of the respective PNA probe. After 90 min of incubation in the dark in a humidified chamber at 55°, unbound probe was removed by washing the slides using a previously warmed wash solution at 55° for 30 min. The preparation was then mounted with a medium suitable for fluorescence microscopy (Vectashield, Vector laboratories, CA). Tissue sections (5 μm) hybridized with PNA probes were observed by fluorescence microscopy, using a BX51 Olympus fluorescence microscope with a DP71 digital camera coupled (Olympus Portugal SA, Porto, Portugal) to analyze the level of colonization in the presence or absence of 30 mM of acid acetic on the surface of RHVE tissues. Quantification of *Candida* cells in the different tissue preparations was performed based on the quantification of genomic DNA. For this, the infected tissues were placed in sterile 1.5 ml microcentrifuge tubes (Eppendorf AG, Hamburg, Germany) with approximately 300 μl of glass beads (0.5 mm diameter, Sigma) and 600 μl of sorbitol buffer (GRiSP, Porto, Portugal). This final mix was homogenized three times for 60 sec, using a Mini-Beadbeater-8 (Stratech Scientific, Soham, UK). After tissue disruption, the supernatant was carefully removed and placed in another sterile microcentrifuge tube. Then, DNA extraction was performed using the GRS Genomic DNA kit – Tissue (GRiSP), in accordance with the manufacturer’s protocol. After extraction, the DNA from each experimental condition was quantified using the NanoDrop 1000 Spectrophotometer (Thermo Fisher Scientific, Wilmington, DE). *C. glabrata* genomic DNA was quantified using real-time PCR in a CF X96 Real-Time PCR System (Bio-Rad, Berkeley). Each reaction mixture consisted of 10 µl of working concentration of SsoFast EvaGreen Supermix (Bio-Rad), 0.2 µl of each primer (50 µM) designed previously (forward: 5′-ATTTGCATGCGCTTGCCCACGAATCC-3′ and reverse: 5′-GGTGGACGTTACCGCCGCAAGCAATGTT-3′), and 4 µl of DNA, in a final reaction volume of 20 µl. Negative controls were performed using a reaction mixture with dH_2_O (Cleaver Scientific Ltd, UK) substituting for the template DNA. Template DNA for each positive control was obtained from FFPE tissues after the step of DNA extraction described above. PCR cycling conditions consisted of an initial denaturation step at 98° for 2 min, followed by 40 cycles of denaturation at 98° for 5 sec, and primer annealing at 60° for 5 sec. In each cycle, a dissociation stage at 60° was run to generate a melting curve for confirming the specificity of the amplification product. Previously, calibration curves (Ct *vs.* Log cells) for each *C. glabrata* strain were constructed using the same PCR protocol as described above. For these, serial dilutions of the *Candida* cells were prepared and the DNA for PCR analysis extracted from the planktonic cell pellet using the DNA extraction kit (QIAamp DNA FFPE Tissue, QIAGEN) with some modifications.

### Data availability

Strains are available upon request. Table S1 contains the list of *C. glabrata* genes whose expression was modified under acetic acid stress (30 mM; pH 4). Table S2 contains the list of genes whose expression was modified under acetic acid stress in the mutant devoid of *CgHAA1* expression. Table S3 describes the list of CgHaa1-regulated genes under acetic acid stress. Table S4 describes the comparison of the genes regulated by ScHaa1 and CgHaa1. Figure S1 shows the effect of *CgHAA1* expression in growth of *C. glabrata* in acidic MM using HCl as the acidulant. Figure S2 shows the effect of *CgHAA1* expression in tolerance of *C. glabrata* to multiple environmental stresses. Figure S3 shows the functional clustering of acetic acid-responsive genes in wild-type *C. glabrata* cells genes, using the MIPS Functional Catalogue Database. Figure S4 and Figure S5 shows clustering of CgHaa1-regulated genes involved in carbohydrate and amino acid metabolism according to the pathways they are involved in, according to the KEGG database. The microarray data are available on the Array Express Database (E-MTAB-4875).

## Results

### CgHAA1 gene (ORF CAGL0L09339g) is required for maximal tolerance of C. glabrata to acetic acid

**Figure 1:**
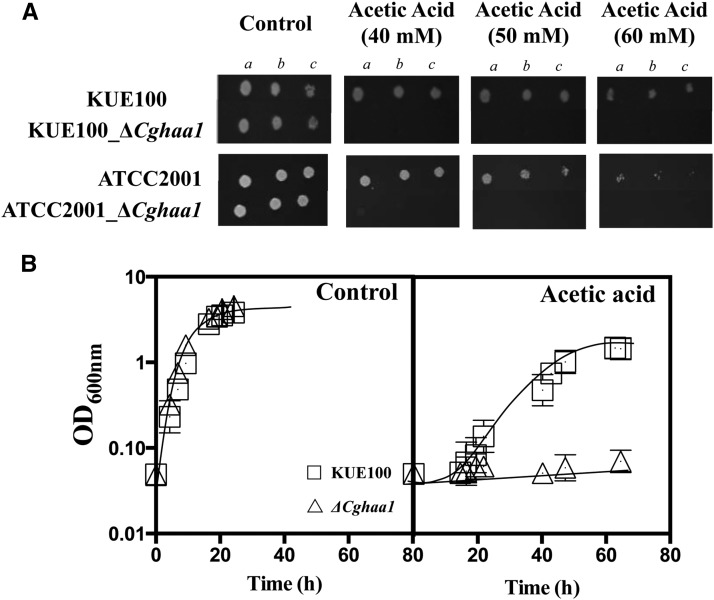
The *CgHAA1* gene is a determinant of *C. glabrata* tolerance to acetic acid. (A) Comparison of the susceptibility of wild-type *C. glabrata* KUE100 or ATCC2001 cells, or of the derived deletion mutants devoid of the *CgHAA1* gene, to inhibitory concentrations of acetic acid (at pH 4.5). Cells used to prepare the spots were cultivated until midexponential phase (as detailed in *Materials and Methods*), harvested by centrifugation, and then resuspended in water to obtain a cell suspension having an OD_600nm_ of 0.05 ± 0.005 (lane a). Lanes (b) and lane (c) are, respectively, 1:5 and 1:25 dilutions of cell suspension shown in lane (a). (B) Growth curves of the parental strain KUE100 (white squares) and of the deletion mutant Δ*CgHaa1* (white triangles) in MM (at pH 4.0; control) or in this same medium supplemented with 60 mM acetic acid. Both the results of the spot assays and of the growth curves shown are representative of at least three independent experiments that gave essentially the same result. MM, minimal medium.

Based on the evidence gathered in *S. cerevisiae* for the Haa1 gene (Fernandes *et al.* 2005; Mira *et al.* 2010a), we hypothesized that the *C. glabrata HAA1* ortholog (ORF CAGL0L09339g) would play a role in conferring tolerance to organic acids and, in particular, to acetic acid in this yeast species. Indeed, deletion of ORF CAGL0L09339g from the genome of the *C. glabrata* KUE100 strain led to a dramatic increase in susceptibility to acetic acid; the mutant strain showing no visible growth when cultivated in MM (at pH 4.5) supplemented with 40, 50, or 60 mM of acetic acid (Figure 1A). Under the same conditions, growth of the wild-type strain was not significantly affected (Figure 1A). Deletion of ORF CAGL0L09339g from the genome of the *C. glabrata* ATCC2001 strain also conferred a strong susceptibility to acetic acid (Figure 1A), demonstrating that the phenotype is independent of the genetic background of the strain used. Based on these findings, ORF CAGL0L09339g was designated as the CgHaa1 gene. The results obtained in liquid MM for the *C. glabrata* KUE100 and Δ*Cghaa1* strains are consistent with those obtained in the spot assays, making evident the high susceptibility to acetic acid of the mutant (Figure 1B). The deletion strain devoid of *CgHAA1* expression failed to grow over 80 hr of incubation in the presence of acetic acid, whereas the wild-type population resumed exponential growth following a lag phase of around 18 hr. However, in the absence of acetic acid there are no significant differences between the growth curves of wild-type and Δ*Cghaa1* cells (Figure 1, A and B). Growth curves of KUE100 and of KUE100_Δ*Cghaa1* cells were indistinguishable when MM was acidified to pH 3 using the strong acid HCl (Figure S1), clearly demonstrating that CgHaa1 is specifically required for *C. glabrata* tolerance to acetic acid, but not to low pH itself.

### Effect of CgHaa1 on C. glabrata tolerance to environmental stress

The effect of *CgHAA1* expression in *C. glabrata* tolerance to environmental stresses other than acetic acid was tested. In particular, the effect of *CgHAA1* expression in conferring protection against inhibitory concentrations of lactic and proponic acids, H_2_O_2_, NaCl, and azoles was examined (Figure S1). Under the experimental conditions used, *CgHAA1* played no role in determining *C. glabrata* susceptibility to azoles to osmotic stress, or to oxidative stress (Figure S2). The Δ*CgHaa1* mutant only showed reduced growth when cultivated in the presence of inhibitory concentrations of lactic and propionic acids (Figure S1) or during cultivation at 42° (Figure S1). These results are consistent with those obtained in a recent phenotypic profiling experiment, in which it was observed that *CgHAA1* deletion did not increase *C. glabrata* susceptibility to azoles, osmotic stress, or to caspofungin (Schwarzmuller *et al.* 2014). Overall, the results obtained indicate that CgHaa1 is not required for general stress resilience in *C. glabrata*, but is specifically required for tolerance to short-chain weak acids.

### Transcriptomic analysis of wild-type and ΔCghaa1 cells under acetic acid stress

**Figure 2:**
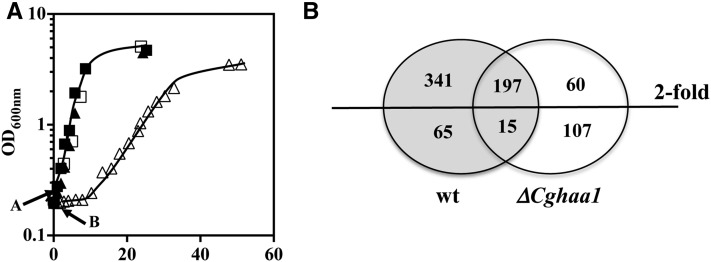
(A) Growth curves of *C. glabrata* KUE100 (squares) and of the deletion mutant Δ*Cghaa1* (triangles) in MM (pH 4.0) (closed symbols) or in this same medium supplemented with 30 mM acetic acid (pH 4) (open symbols). The arrow indicates the time of cultivation at which cell samples were harvested for the transcriptomic analysis. Growth of the cultures was followed by accompanying the increase in culture OD_600 nm_ and in the concentration of viable cells, assessed as the number of colony-forming units per milliliter of cell culture (CFU/ml). The growth curves shown are representative of at least three independent growth curves that gave rise to the same growth pattern. (B) Venn diagram summarizing the number of genes up- or downregulated (above twofold) in response to acetic acid in cells of the parental strain *C. glabrata* KUE100 and/or in the mutant Δ*Cghaa1*. wt, wild-type.

Considering the important role of CgHaa1 in *C. glabrata* tolerance to acetic acid and its predicted function as a transcriptional regulator, we used global transcriptional analysis using species-specific microarrays to identify the set of CgHaa1 target genes. KUE100 and KUE_Δ*Cghaa1* cells were harvested after 30 min of exposure to 30 mM of acetic acid (at pH 4.0). This concentration is within the range of concentrations usually found in the vaginal tract, especially when bacterial overproliferation occurs (Owen and Katz 1999; Chaudry *et al.* 2004) (Figure 2). Under the selected experimental conditions, the wild-type strain exhibited no significant lag phase whereas the Δ*Cghaa1* mutant showed no visible growth for a period of around 20 hr, after which exponential growth was resumed (Figure 2A). For this analysis, we have chosen to use a milder concentration of acetic acid, below the one used in the phenotypic assays, to avoid the induction of a wide nonspecific stress response, particularly in the highly susceptible Δ*Cghaa1* mutant, which would further complicate the identification of genes specifically regulated by CgHaa1. No significant loss of cell viability was observed in the two acid-challenged populations (not shown), which is consistent with the described fungistatic (and not fungicidal) effect of acetic acid (Mira *et al.* 2010c; Piper *et al.* 2001).

The exposure of the wild-type strain to acetic acid resulted in the statistically significant (p < 0.01) upregulation (above twofold) of 538 genes, while 80 genes were downregulated (above twofold), compared with the transcript levels registered in cells cultivated in the absence of the acid (Figure 2B and Table S1). Functional clustering of the upregulated genes revealed an enrichment (p < 0.01) of genes encoding enzymes involved in central carbon metabolism (particularly in glycolysis, the Krebs cycle, the pentose phosphate pathway, and the catabolism of glycogen and trehalose); amino acid metabolism; ion transport (in particular ammonium, iron, potassium, and calcium); and the response to oxidative or to low pH (Figure S3 and Table S1). This transcriptional response is consistent with described toxic effects of acetic acid stress in fungal cells, which include the depletion of ATP and amino acid pools, intracellular acidification, inhibition of nutrient uptake, protein denaturation, and reduction of the internal iron pool (Ullah *et al.* 2013; Mira *et al.* 2010a,b; Cottier *et al.* 2015a; Hueso *et al.* 2012). A significant number of genes related to cell wall function was also found to be upregulated in acetic acid-stressed *C. glabrata* cells, including genes involved in the synthesis of β-1,3 and β-1,6 glucans (*e.g.*, CgFks2, CgKnh1, CgKre6, and CgGas5), mannoproteins linked to the uptake of sterols (CgTir1, CgTir3,and CgYeh2) or required for the maintenance of cell wall structure (CgCwp1 and CgCwp2), and several predicted adhesins (*e.g.*, CgEpa15, CgEpa12, CAGL0M11726g, and CAGL0K10164g) (Figure S3 and Table S1).

Genes downregulated under acetic acid stress in *C. glabrata* cells are enriched in protein synthesis and in ribosomal biogenesis (Table S1), the repression of these functions being a hallmark of the response of this yeast species to environmental stress (Roetzer *et al.* 2008). Significant repression of ribosomal genes and of ribosomal RNA-encoding genes was also reported following exposure of *C. albicans* and *S. cerevisiae* to acetic acid, this being associated with the drastic reduction in the amount of ribosomal RNA in these acid-stressed cells (Cottier *et al.* 2015a; Mroczek and Kufel 2008). Transport between the ER and the Golgi is the other functional class enriched (p < 0.01) in the dataset of *C. glabrata* genes repressed under acetic acid stress (Table S1).

Exposure to 30 mM acetic acid (at pH 4) in the Δ*Cghaa1* mutant led to the upregulation of 257 genes, 197 of them being in common with those that were induced in the wild-type strain (Figure 2B and Table S2). Closer inspection of the 60 genes only found to be upregulated in the highly susceptible Δ*Cghaa1* strain showed an enrichment of genes involved in the oxidative stress response (*CgGPX2*, *CgSOD2*, *CgGRX1*, *CAGL0M13189g*, and *CgCTA1*) and in the biosynthesis of methionine and cysteine (*CgSUL2*, *CgMET3*, *CgMET10*, *CgMET17*, *CgMET3*, *CgMET15*, and *CgMET14*) (Figure S2 and Table S2). Acetic acid has been demonstrated to induce oxidative stress in yeast (Semchyshyn *et al.* 2011) and to drastically reduce the methionine pool in *Escherichia coli* (Roe *et al.* 2002). Since Δ*Cghaa1* cells accumulate more acetic acid than wild-type cells (see below), it is likely that the increased expression of the above-referred genes in the mutant background might result from a potentiated toxic effect of the acid. Also in line with this idea, 46 of the genes that were only found to be repressed in the ΔCgHaa1 mutant have a biological function related to ribosome biogenesis (Table S2) that, as noted above, appears to be repressed under acetic acid stress.

### Characterization of the CgHaa1-regulon active under acetic acid stress

The comparison of the transcriptomes of wild-type and Δ*Cghaa1* cells led to the identification of 341 genes with increased expression (above twofold) in response to acetic acid in wild-type cells but that had no detectable change in expression in the Δ*Cghaa1* mutant. Furthermore, the acid-induced transcriptional activation of 63 other genes was reduced by more than 50% in the Δ*Cghaa1* background (for example, the transcript levels of *CgTPO3* are increased ∼16-fold in acid-stressed wild-type cells but only by 2.9-fold in the Δ*Cghaa1* mutant) (Table 3 Table S3). These correspond to genes whose acid-induced transcriptional activation was partly mediated by CgHaa1. Overall, 404 genes were found to be positively regulated by CgHaa1 in response to acetic acid stress, corresponding to 75% of the total set of acid-responsive genes (Figure 2, B and C and Table S3). Subsets of the CgHaa1-activated genes are shown in Table 2 and Table 3, and the full list is available in Table S3. To confirm some of the results of the microarray analysis, the transcript levels of a selected set of genes (*CgPMP2*, *CgPMA1*, *CgTPO3*, and *CAGL0G05632g*) were compared in wild-type and in Δ*Cghaa1* cells using quantitative real-time RT-PCR. The results obtained coincided with those of the microarray analysis, although the ratios of transcriptional activation induced by acetic acid were consistently higher in the microarrays (Figure 4).

**Table 2:** C. *glabrata* genes whose acetic acid-induced transcriptional activation was fully mediated by CgHaa1

ORF	*C. glabrata* Gene	LogFC (wt AC/wt CTRL)	Function	*S. cerevisiae* Ortholog	*S. cerevisiae* Ortholog Regulated by ScHaa1?	*S. cerevisiae* Homolog Confers Resistance to Acetic Acid?
CAGL0C04323g		5,33	Ortholog(s) have α,α-trehalase activity, role in trehalose catabolic process, and cytoplasm localization	*NTH1*	Not described	No
CAGL0I07249g		4,71	Putative GTPase-activating protein involved in cell wall and cytoskeleton homeostasis; gene is upregulated in azole-resistant strain	*BAG7*	Not described	No
CAGL0K07337g		4,37	Has domain(s) with predicted ion channel activity, role in ion transport, and membrane localization	*HSP30*	Yes	No
CAGL0H02585g		4,19	Ortholog(s) have glutamate decarboxylase activity, role in cellular response to oxidative stress, glutamate catabolic process, and cytoplasm localization	*GAD1*	Yes	No
CAGL0H10076g		4,12	Has domain(s) with predicted ion channel activity, role in ion transport, and membrane localization	*YRO2*	Yes	Yes
CAGL0G06182g		3,72	No description available	*YHR131C*	Not described	No
CAGL0A01804g	*CgHXT1*	3,65	Ortholog(s) have fructose transmembrane transporter activity, pentose transmembrane transporter activity, role in glucose transport, mannose transport, and plasma membrane localization	*HXT1*	Not described	No
CAGL0K03421g		3,39	Ortholog(s) have cytosol, nucleus localization	*PGM2*	Yes	No
CAGL0L08008g		3,39	No description available	*PMP2*	Not described	Yes
CAGL0G05269g		3,27	Putative mitochondrial protein; gene is downregulated in azole-resistant strain	*FMP16*	Not described	No
CAGL0I09702g		3,24	Ortholog(s) have riboflavin transporter activity, role in riboflavin transport, and plasma membrane localization	*MCH5*	Yes	Yes
CAGL0E05148g		3,23	Ortholog(s) have α-mannosidase activity, role in oligosaccharide catabolic process, and cytosol, fungal-type vacuole membrane localization	*AMS1*	Not described	No
CAGL0G02057g		3,19	Ortholog(s) have cytoplasm, nucleus localization	*YKR075C*	Yes	No
CAGL0H04851g		3,18	Ortholog(s) have 4-nitrophenylphosphatase activity, protein serine/threonine phosphatase activity, and role in cellular protein localization, cellular sodium ion homeostasis, and protein dephosphorylation	*PPZ1*	Not described	No
CAGL0A02002g		3,16	No description available	*YOL024W*	Not described	No
CAGL0K07590g	*CgMYO3*	3,11	Putative myosin	*MYO3*	Not described	No
CAGL0I05148g	*CgDLD1*	3,07	D-lactate ferricytochrome C oxidoreductase	*DLD1*	Not described	No
CAGL0G02563g		3,06	Has domain(s) with predicted ubiquitin thiolesterase activity and role in ubiquitin-dependent protein catabolic process			
CAGL0G03179g		3,06	Has domain(s) with predicted phospholipid binding activity	*ASK10	Not described	Not tested
CAGL0A00495g	*CgPMA1*	2,98	Putative plasma membrane proton pump with a predicted role in pH homeostasis	*PMA1	Not described	Not tested
CAGL0A01870g		2,97	Has domain(s) with predicted integral to membrane localization			
CAGL0I06644g		2,94	Putative GPI-linked cell wall protein	*SPI1*	Not described	No
CAGL0M06897g		2,91	Ortholog(s) have cytoplasm localization	*YNL024C*	Yes	No
CAGL0H07469g		2,90	Putative adhesin-like protein	*ICS2*	Not described	No
CAGL0G05698g	*CgGDH2*	2,89	Ortholog(s) have glutamate dehydrogenase (NAD+) activity, role in nitrogen compound metabolic process, and cytosol, mitochondrion localization	*GDH2*	Not described	No
CAGL0A01716g		2,87	Ortholog(s) have nicotinamidase activity, role in chromatin silencing at rDNA, chromatin silencing at telomere, replicative cell aging, and cytosol, nucleus, and peroxisome localization	*PNC1*	Not described	No
CAGL0E03630g		2,84	Ortholog(s) have RNA binding activity and role in negative regulation of conjugation with cellular fusion, premeiotic DNA replication, reciprocal meiotic recombination, and sporulation resulting in formation of a cellular spore	*RIM4*	Not described	No
CAGL0J11462g		2,84	Predicted GPI-linked cell wall protein	*YNL190W*	Not described	No
CAGL0G03267g		2,80	Ortholog(s) have role in protein targeting to membrane and cytoplasm localization	*AST2*	Not described	No
CAGL0A01650g		2,80	Putative protein; gene is upregulated in azole-resistant strain	*ECL1*	Yes	No
CAGL0E01749g	*CgYPS4*	2,78	Putative aspartic protease; member of a YPS gene cluster that is required for virulence in mice; induced in response to low pH and high temperature	*YPS1*	Not described	No

The genes found to be upregulated in response to acetic acid in the wild-type and in the Δ*Cghaa1* strains were compared and those genes whose acid-induced activation was abrogated in the mutant background were selected as CgHaa1 targets. A subset of these CgHaa1-activated genes is shown in this table but the full list is shown in Table S3. The biological function indicated was based on the information available at the *Candida* Genome Database website or on the information available for the corresponding *S. cerevisiae* ortholog. Essential genes are indicated with * (predicted based on the information available for *S. cerevisiae*). Information on the involvement of the corresponding *S. cerevisiae* (Sc) orthologs in tolerance to acetic acid and their inclusion in the ScHaa1-dependent transcriptional regulatory network is also shown, based on the information available in the YEASTRACT database (Teixeira *et al.* 2014) or on published results (Mira *et al.* 2010a,b). ORF, open reading frame; AC, Acetic acid; CTRL, Control; GTPase, guanosine triphosphatase; GPI, glycosylphosphatidylinositol; NAD, nicotinamide, adenine dinucleotide; YPS, *Yersinia pseudotuberculosis*. log - log2

**Table 3:** C *glabrata* genes whose acetic acid-induced transcriptional activation was partly mediated by CgHaa1

ORF	*C. glabrata* Gene	LogFC (wt AC/wt CTRL)	LogFC (ΔCghaa1AC/ΔCghaa1 CTRL)	Function	*S. cerevisiae* Ortholog	*S. cerevisiae* Ortholog Regulated by ScHaa1?	*S. cerevisiae* Homolog Confers Resistance to Acetic Acid?
CAGL0I06182g	CgPIR2	7,65	3,10	O-mannosylated heat shock protein that is secreted and covalently attached to the cell wall via β-1,3-glucan and disulfide bridges; required for cell wall stability; induced by heat shock, oxidative stress, and nitrogen limitation	HSP150	Not described	No
CAGL0G05632g		6,95	1,41	Ortholog(s) have cytoplasm localization	*YDL218W*	Not described	No
CAGL0I10010g		6,82	3,66	v-SNARE binding protein that facilitates specific protein retrieval from a late endosome to the Golgi; modulates arginine uptake, possible role in mediating pH homeostasis between the vacuole and plasma membrane H^+^-ATPase	*BTN2*	Not described	No
CAGL0M01166g		6,44	4,20	Ortholog(s) have ferrous iron binding activity, role in mitochondrial genome maintenance, thiamine biosynthetic process, thiazole biosynthetic process, and cytosol or nucleus localization	*THI4*	Not described	No
CAGL0F08261g		6,11	2,55	Ortholog(s) have phosphopyruvate hydratase activity, role in glycolysis, regulation of vacuole fusion, nonautophagic and fungal-type vacuole, internal side of plasma membrane, mitochondrion, and phosphopyruvate hydratase complex localization	*ENO1*	Not described	
CAGL0G03883g		6,10	4,05	Disaggregase; Heat shock protein that cooperates with Ydj1p (Hsp40) and Ssa1p (Hsp70) to refold and reactivate previously denatured, aggregated proteins; responsive to stresses including: heat, ethanol, and sodium arsenite; involved in [PSI+] propagation	*HSP104*	Yes	
CAGL0G03289g	*CgSSA3*	6,10	2,09	Heat shock protein of the HSP70 family	*SSA4*	Yes	Yes
CAGL0J00451g		5,66	4,38	Putative glyceraldehyde-3-phosphate dehydrogenase; protein differentially expressed in azole resistant strain; expression downregulated in biofilm *vs.* planktonic cell culture	*TDH3*	Not described	No
CAGL0M08822g	*CgHSP78*	5,06	3,34	Ortholog(s) have ATPase activity, misfolded protein binding activity	*HSP78*	Not described	No
CAGL0E00803g		4,82	2,88	Putative small cytosolic stress-induced chaperone; gene is upregulated in azole-resistant strain	*HSP42*	Yes	No
CAGL0H03707g		4,64	2,57	Ortholog(s) have role in protein folding, translational initiation and cytosolic small ribosomal subunit, nucleus localization	*SIS1	Not described	No
CAGL0G08866g		4,38	1,96	Ortholog(s) have RNA polymerase II transcription factor binding, RNA polymerase II transcription factor binding transcription factor activity, sequence-specific DNA binding activity	*FKH2*	Yes	No
CAGL0C02321g	*CgPHM8*	4,25	2,36	Ortholog(s) have nucleotidase activity and role in pyrimidine nucleobase metabolic process	*SDT1*	Not described	No
CAGL0K10164g		4,08	2,54	Predicted GPI-linked protein; putative adhesin-like protein	*SPI1*	Yes	No
CAGL0I09724g		4,07	2,05	Unknown			No
CAGL0F04631g		4,03	1,84	No description available	*MOH1*	Not described	No
CAGL0F04631g		4,03	1,84	Protein of unknown function, has homology to kinase Snf7p; not required for growth on nonfermentable carbon sources; essential for survival in stationary phase	*MOH1*	Not described	No
CAGL0I10384g	*CgTPO3*	3,98	1,49	Predicted polyamine transporter of the major facilitator superfamily; required for azole resistance	*TPO3*	Yes	Yes
CAGL0J06050g		3,97	1,41	Has domain(s) with predicted role in cellular amino acid metabolic process	*YGP1*	Yes	No
CAGL0H00704g		3,95	2,86	Protein of unknown function; mobilized into polysomes upon a shift from a fermentable to nonfermentable carbon source; potential Cdc28p substrate	*ICY2*	Not described	Yes
CAGL0G08624g	*CgQDR2*	3,92	2,69	Drug:H^+^ antiporter of the Major Facilitator Superfamily, confers imidazole drug resistance, involved in quinidine/multidrug efflux; gene is activated by Pdr1p; upregulated in azole-resistant strain	*QDR1*	Not described	No

The genes found to be upregulated in response to acetic acid in the wild-type and in the Δ*Cghaa1* strains were compared and those genes whose acid-induced activation was reduced by more than 50% in the mutant background were selected as CgHaa1 targets. A subset of these CgHaa1-activated genes is shown in this table but the full list is shown in Table S3. The biological function indicated was based on the information available at the *Candida* Genome Database website or on the information available for the corresponding *S. cerevisiae* ortholog. Essential genes are indicated with * (predicted based on the information available for *S. cerevisiae*). Information on the involvement of the corresponding *S. cerevisiae* (Sc) orthologs in tolerance to acetic acid and their inclusion in the ScHaa1-dependent transcriptional regulatory network is also shown, based on the information available in the YEASTRACT database (Teixeira *et al.* 2014) or on published results (Mira *et al.* 2010a,b). ORF, open reading frame; AC, acetic acid; CTRL, Control; SNARE, soluble NSF attachment protein receptor; ATPase, adenosine triphosphatase; GPI, glycosylphosphatidylinositol. log-log2

**Figure 3:**
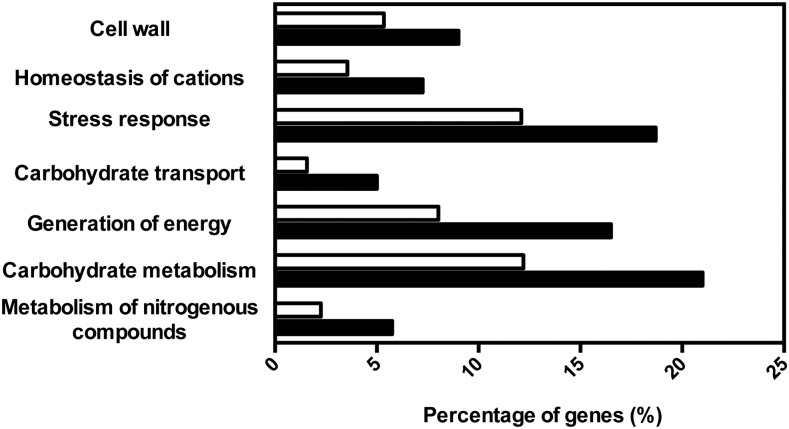
Functional clustering of CgHaa1-activated genes in response to acetic acid stress. The genes found to be upregulated in response to acetic acid stress in a CgHaa1-dependent manner were clustered according to their biological function, using the MIPS Functional Catalogue database (black bars), and the enriched functional classes (p < 0.001) were selected. The percentages shown correspond to the ratio of the number of genes included in each functional class and the total number of CgHaa1-regulated genes. The white bars represent the percentage of genes clustered in each functional class using as an input dataset the entire ORFeome of *C. glabrata* CBS138.

The functional categories “metabolism of nitrogenous compounds,” “carbohydrate metabolism,” “generation of energy,” “stress response,” “transport of carbohydrates,” “homeostasis of cations,” and “cell wall” are enriched (p < 0.001) in the dataset of CgHaa1 target genes (Figure 3). The CgHaa1 targets clustered in the carbohydrate, generation of energy, and amino acid metabolism classes include genes involved in histidine biosynthesis and in the main pathways of central carbon metabolism, including glycolysis, the glyoxylate cycle, the pentose phosphate pathway, the Krebs cycle, and the catabolism of trehalose and glycogen (Figure S3, Table 2, Table 3, Table S2, and Table S3). The stress response functional class is composed of a wide range of stress-responsive genes encoding protein chaperones (*e.g.*, *CAGL0E00803g*, *CgSSA3*, *CgFES1*, *CgSSA1*, *CgHSP31*, *CAGL0G03883g*, or *CAGL0H08195g*) or enzymes of the antioxidant response (*e.g.*, CAGL0C03850g or CAGL0K00803g) (Table S3). The CgHaa1 target genes clustered in the “homeostasis of cations” function class are involved in the transport of calcium, zinc, and iron (*e.g.*, CAGL0A00517g, CAGL0D03322g, and CAGL0F00187g). Genes involved in the control of internal pH homeostasis (proton transport) such as the essential proton pump-encoding gene *CgPMA1*, its predicted regulators *CgPMP2* and *CgHSP30*, and *CgVMA1*, encoding a subunit of the vacuolar proton pump, were also clustered in the “ion transport” functional class (Table S3). Seventy-eight of the CgHaa1 target genes were found to have a transport-related function, although this class was not considered enriched in the dataset of CgHaa1 targets (p of 0.007, above the threshold of 0.001 used). CgHaa1-activated genes grouped in this class included several transporters involved in the uptake of carbohydrates (*e.g.*, *CgHXT1* and *CgHXT4*), as well as the putative drug efflux pumps CgTpo3, CgQdr2, CAGL0M07293g, and CAGL0L10912g (Table 2, Table 3 Table S2, and Table S3).

### Effect of CgHaa1 target genes in C. glabrata tolerance to acetic acid

The strong susceptibility of the Δ*Cghaa1* mutant to acetic acid could be attributable to the lack of expression of one (or more) acid-induced gene(s) that directly contribute to tolerance to this acid. There are 35 CgHaa1 target genes whose orthologs were implicated in acetic acid tolerance in *S. cerevisiae* (Table 2, Table 3 and Table S3). It is difficult to determine the individual effect of the CgHaa1-regulated genes on tolerance to acetic acid in *C. glabrata*, not only because the dataset is large (404 genes), but also because a substantial percentage of these genes have paralogs and/or are essential, as in the case of the plasma membrane proton pump-encoding gene *CgPMA1* (Bairwa and Kaur 2011) (Table 2, Table S2, and Table S3). Therefore, we examined the protective effect against acetic acid of a set of 15 CgHaa1-regulated genes, namely, *CgSSA3*, *CgFPS1*, *CgFPS2*, *CgSUT2*, *CgMIT1*, *CgTPO3*, *CgHSP30*, *CgBAG7*, *CgYPS4*, *CgGAD1*, *CgENO2*, *CgRSB1*, *CgCRM3*, *CAGL0G05632g*, and *CAGL0E03740g*. These genes were selected based on their high level of upregulation in response to acetic acid, the dependence on CgHaa1 expression, and/or on the reported involvement of their homologs in acetic acid tolerance in *S. cerevisiae* (Table 2 and Table 3). For this, deletion mutants were constructed in the KUE100 background and their susceptibility to acetic acid (60 mM at pH 4) was compared with the parental strain. Despite their potent upregulation (by 26-, 20- and 123-fold, respectively), the deletion of *CAGL0I07249g*, *CAGL0H02585g*, and *CAGL0G05632g* genes did not increase *C. glabrata* susceptibility to acetic acid. In fact, out of the 15 mutants tested, only Δ*Cgtpo3* and Δ*Cghsp30* were found to be significantly more susceptible to acetic acid than the parental strain (Figure 5A).

### Expression of CgHaa1 and of the CgHaa1-regulated gene CgTpo3 reduces the internal accumulation of acetic acid

**Figure 4:**
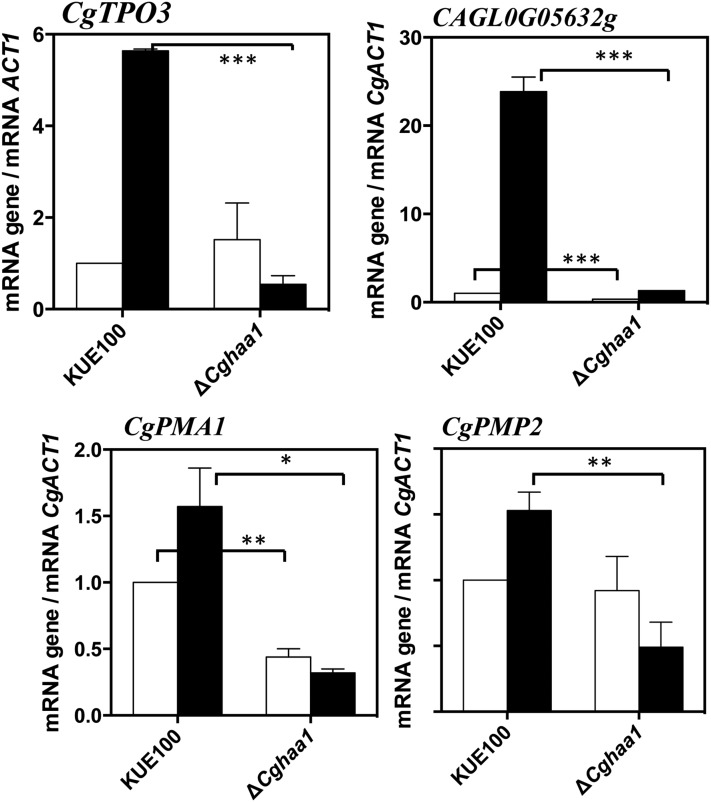
Comparison, by quantitative real-time RT-PCR, of the transcript levels of *CgTPO3*, *CgPMP2*, *CgPMA1*, and *CAGL0G05632g* genes in *C. glabrata* wild-type KUE100 and KUE100_Δ*Cghaa1* cells under acetic acid stress. Levels of mRNA of the above-referred genes/ORFs were compared in cultures of the two strains after 30 min of cultivation in MM (at pH 4.0) (white bars) or in this basal medium supplemented with 30 mM of acetic acid (dark bars). Transcript levels were normalized using as internal control the levels of *CgACT1* mRNA and the values presented are relative to those registered in unstressed wild-type cells, (which were considered to be equal to 1). The results shown are means of three independent experiments. Statistical significance of the results was assessed using ANOVA, taking into account the different replicate assays performed. *** p < 0.001, ** p < 0.01, * p < 0.05. MM, minimal medium; mRNA, messenger RNA; ORF, open reading frame; RT-PCR, reverse transcription-polymerase chain reaction.

**Figure 5:**
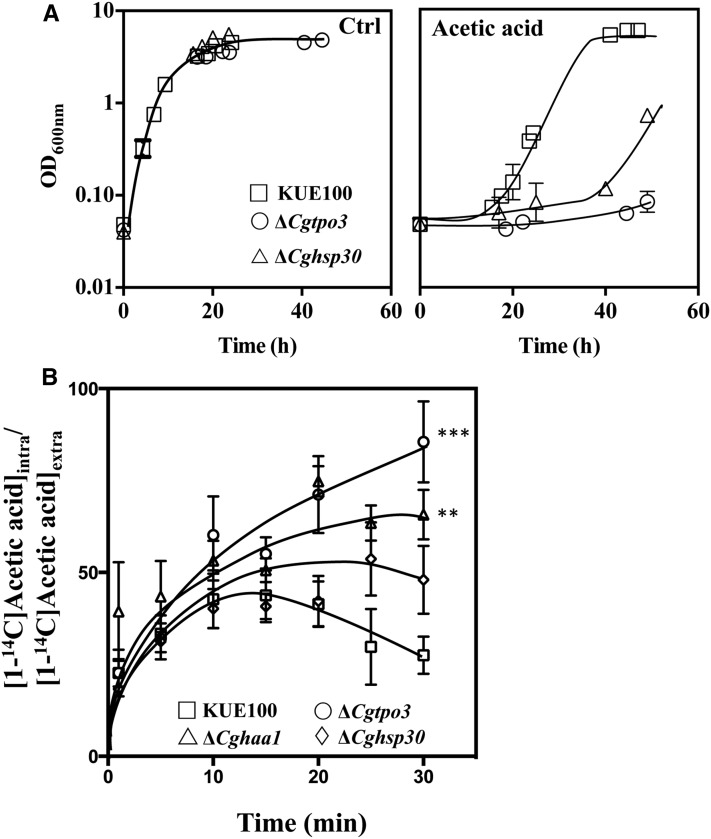
(A) The CgHaa1 target genes *CgHSP30* and *CgTPO3* are required for maximal *C. glabrata* tolerance to acetic acid. Growth curve of KUE100 (white squares) or of the derived mutants devoid of *CgTPO3* (white circles) or of the *CgHSP30* (white triangles) gene in MM (at pH 4.0) either supplemented or not with acetic acid (60 mM). The growth curves shown are representative of at least three independent experiments that gave essentially the same results. (B) The expression of *CgTPO3* and of *CgHAA1* reduces intracellular accumulation of acetic acid. Time-course representation of the accumulation ratio, A, of [1-^14^C]-acetic acid in wild-type *C. glabrata* KUE100 (white squares) or in the deletion mutants Δ*Cghaa1* (white triangles), Δ*Cgtpo3* (white circles), or Δ*Cghsp30* (white diamonds) during cultivation in MM (at pH 4) supplemented with 60 mM of cold acetic acid. The asterisks stand for the assessment of the statistical difference registered in the last time point (30 min) of the accumulation ratio registered in the different strains, based on the results of the five replicates that were performed in this assay. *** p < 0.001, ** p < 0.01. Ctrl, control; MM, minimal medium.

The strong susceptibility of the Δ*Cgtpo3* mutant to acetic acid (Figure 5), together with the upregulation of the *CgTPO3* gene registered in cells challenged with this acid (Figure 4 and Table 2), prompted us to examine the effect of this putative drug efflux pump in reducing the intracellular accumulation of acetic acid in *C. glabrata*. To test this hypothesis, the accumulation of radiolabeled acetic acid was followed during the first 30 min of incubation of KUE100 and KUE100_Δ*Cgtpo3* cells in MM supplemented with 60 mM of cold acetic acid (Figure 5B). The internal accumulation of radiolabeled acetic acid was consistently higher in the Δ*Cgtpo3* mutant than in the wild-type strain, attaining a maximum difference of about threefold after 30 min of cultivation (Figure 5B). The amount of acetic acid accumulated intracellularly in the Δ*Cghaa1* mutant was also higher (around 2.5-fold) than the levels attained in the parental strain (Figure 5B). The expression of *CgHSP30*, the other gene regulated by CgHaa1 that contributed for maximal *C. glabrata* tolerance to acetic acid, also marginally reduced the internal accumulation of acetic acid (Figure 5B).

### Under acetic acid stress, CgHaa1 expression increases the content in the plasma membrane and the activity of the proton pump CgPma1

**Figure 6:**
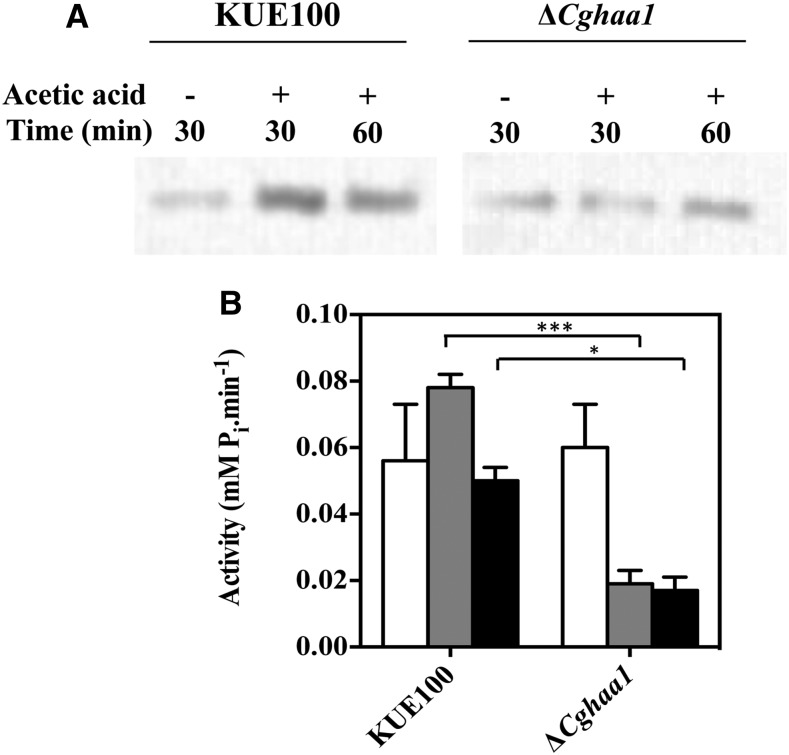
*CgHAA1* expression leads to increased content and activity of the plasma membrane H^+^-ATPase CgPma1. The content of CgPma1 present at the plasma membrane of unstressed and acetic acid-challenged wild-type and Δ*Cghaa1* cells was compared by western blot, as detailed in *Materials and Methods*. Cells of the two strains were cultivated in MM (at pH 4) (control; white bars) or in this same medium supplemented with 30 mM acetic acid. After 30 (gray bar) and 60 (black bar) min of incubation in the presence or absence of the acid, cells were harvested and plasma membrane-enriched protein fractions were obtained for quantification of CgPma1 concentration (A) or for estimation of CgPma1 activity (B). The relative values of CgPma1 shown were obtained upon densitometry of the signal obtained in the membranes used for the western blot shown in (A). Equal loading of the different protein extracts was confirmed by staining the membrane prior to signal detection. To calculate the relative abundance of CgPma1, the signal obtained in the different protein fractions was compared to the one obtained in unstressed wild-type cells, which was considered equal to 1. The results shown are means of at least three independent experiments. Statistical significance of the data shown in the different panels was assessed using ANOVA and taking into account the different replicas performed. *** p < 0.001, * p < 0.05. MM, minimal medium.

The plasma membrane proton pump Pma1 has an essential role in the control of internal pH homeostasis in yeasts, including in *C. glabrata* (Ullah *et al.* 2013; Bairwa and Kaur 2011), and was found in this work to be upregulated under acetic acid stress under the dependence of *CgHAA1* (Table 2). Additionally, the acetic acid-induced expression of *CgPMP2*, *CgHSP30*, CAGL0F03707g, and CAGL0C02893g genes, all predicted to encode regulators of CgPma1 activity, also required CgHaa1 (Table 2 and Table 3). Therefore, we hypothesized that CgHaa1 may regulate the activity and the concentration of CgPma1 present in the plasma membrane in response to acetic acid stress. To test this hypothesis, a western blot was used to compare the amount of CgPma1 present in plasma membrane-enriched protein fractions recovered from wild-type and Δ*Cghaa1* cells cultivated in the presence or absence of acetic acid (Figure 6). The results obtained confirmed that the levels of CgPma1 present in the plasma membrane of acetic acid-challenged KUE100 cells are between two and threefold higher than those attained in the Δ*Cghaa1* mutant cells (Figure 6A). The activity of CgPma1 is also higher (between three and fourfold) in KUE100 than in Δ*Cghaa1* cells (Figure 6B). In fact, upon sudden exposure to acetic acid, the activity of CgPma1 in the mutant background suffered a dramatic drop, whereas activity in the wild-type strain increased, in comparison to the values registered in control cells (Figure 6B). The lower activity of CgPma1 in the ΔCghaa1 mutant is consistent with the reduced transcript levels of the *CgPMA1* and *CgPMP2* genes registered in these cells, in comparison with those attained by parental strain cells (Figure 4).

### CgHaa1 improves adhesion and colonization of reconstituted human vaginal epithelium by C. glabrata in the presence of acetic acid

**Figure 7:**
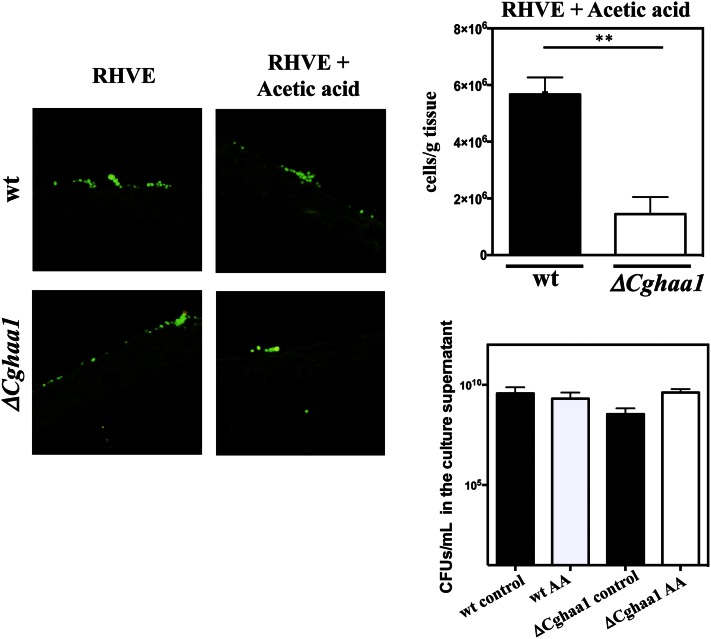
The expression of *CgHAA1* increases *C. glabrata* adhesion and colonization of reconstituted human vaginal epithelium. Wild-type KUE100 and KUE100_Δ*Cghaa1* cells were cultivated in RPMI growth medium (at pH 4), either or not supplemented with 30 mM acetic acid, in the presence of reconstituted human vaginal epithelium. After 24 hr of incubation in the presence of the tissue, yeast cells were stained using a specific PNA FISH. Scale bars correspond to 20 µm. Quantification of the number of yeast cells of the two strains that were able to colonize the tissue was performed based on quantification of *C. glabrata* genomic DNA. Viability of wild-type or Δ*Cghaa1* plakntonic cells during cultivation in the RPMI growth medium under the experimental conditions used is shown. Statistical significance of the data shown was assessed using ANOVA and taking into account the different replicas performed. ** p < 0.01. MM, minimal medium; PNA FISH, Peptide nucleic acid probe fluorescence *in situ* hybridization; RHVE, reconstituted human vaginal epithelium; RPMI, Roswell Park Memorial Institute; wt, wild-type.

Nine genes (*AWP12*, *AWP13*, *CgEPA2*, CAGL0K10164g, CAGL0H07469g, CAGL0H00110g, CAGL0F09273g, CAGL0M11726g, and CAGL0E06666g) upregulated by CgHaa1 under acetic acid stress encode predicted adhesins (Table 2, Table 3 Table S2, and Table S3). This observation, together with the previously described role of ScHaa1 in *S. cerevisiae* adherence (Malcher *et al.* 2011), led us to test whether CgHaa1 is required for adhesion and subsequent colonization of RHVE by *C. glabrata*. KUE100 and KUE100_Δ*Cghaa1* cells were cultivated for 24 hr in RPMI growth medium with or without acetic acid (30 mM at pH 4) in the presence of RHVE (Figure 7). In the absence of acetic acid, there were no significant differences in the ability of the two strains to adhere to the RHVE tissue; however, when acetic acid was present in the medium, the number of Δ*Cghaa1* cells adhered to the vaginal epithelial tissue was less than half that of the parental strain (Figure 7). Under the experimental conditions that were used, the viability of planktonic Δ*Cghaa1* cells was not significantly affected by the presence of acetic acid (Figure 7), indicating that these cells are not *a priori* less capable of colonizing the tissue by suffering a stronger toxic effect of the acid. This observation suggests that the reduced number of Δ*Cghaa1* cells recovered from the RHVE tissue may reflect a true defect in adhesive capacity of this strain, although we cannot totally exclude the possibility of also resulting from a growth defect of this strain, since growth in the epithelial layer and in planktonic conditions may differ. Notably, under the experimental conditions that were used, the presence of acetic acid in the culture medium reduced (in the range of 20–25%) the number of KUE100 and Δ*Cghaa1 C. glabrata* cells recovered from the surface of the epithelium (results not shown). Although acetic acid leads to increased expression of adhesins that could facilitate adhesion to the vaginal epithelial cells, its presence in the acidic environment also leads to other deleterious toxic effects for the yeast cells that could compromise, or at least slow down, the adhesion process.

## Discussion

Despite being known that *C. glabrata* cells are challenged with high concentrations of acetic acid during colonization of the vaginal tract, little is known about the molecular mechanisms underlying tolerance of this yeast to this organic acid. The present study is, to our knowledge, the first to examine such tolerance mechanisms at a pH similar to the one found in vaginal fluid, with an emphasis on the role played by the CgHaa1-dependent signaling system in that response. Our results show that CgHaa1 is an essential determinant of *C. glabrata* tolerance to acetic acid, and also to lactic and propionic acids, two other carboxylic acids that also challenge this yeast species in the vaginal and/or gastrointestinal tract (Yamaguchi *et al.* 2005; Hickey *et al.* 2012; Boskey *et al.* 2001). Transcriptomics revealed that CgHaa1 regulates, directly or indirectly, about 75% of the acetic acid-induced genes. The set of genes downregulated by acetic acid was only marginally affected by *CgHAA1* deletion, suggesting that CgHaa1 works mainly as a transcriptional activator, similar to its *S. cerevisiae* ortholog (Mira *et al.* 2010a). Although a substantial number of the genes upregulated under acetic acid stress are involved in the general response of *C. glabrata* cells to environmental stress (Roetzer *et al.* 2008), neither CgMsn2 nor CgMsn4, the regulators of this general response (Roetzer *et al.* 2008), were found to be required for maximal tolerance to acetic acid (unpublished results). Likewise, the expression of CgWar1, required for *C. glabrata* tolerance to propionic or sorbic acids, was also dispensable for tolerance to acetic acid (Mundy and Cormack 2009). Altogether, these observations support the idea that CgHaa1 is the central player in the control of the *C. glabrata* response and tolerance to acetic acid stress in acidic environments. Interestingly, tolerance to acetic acid in *C. albicans* also appears to be under the control of dedicated regulatory systems since Mnl1, a key player in the control of the response of this yeast species to acetic acid stress, was found to be largely dispensable for the response to other environmental stressors (Ramsdale *et al.* 2008).

**Figure 8:**
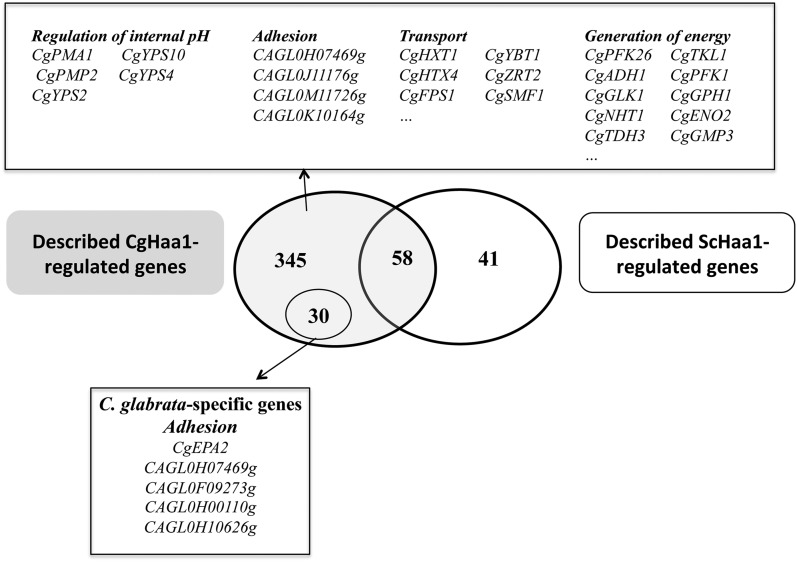
Comparison of the CgHaa1- and ScHaa1-regulons active in response to acetic acid stress. The dataset of genes found to be activated by CgHaa1 or by ScHaa1 during response of *C. glabrata* or *S. cerevisiae* to acetic acid (30 mM at pH 4) were compared using the data obtained in this study and previously published data (Mira *et al.* 2010a). The intersection of the two datasets revealed a modest overlap between the two networks and led to the identification of 14 genes that are only regulated by CgHaa1 and that are specific to *C. glabrata*. The functional classes most represented within the dataset of genes specifically regulated by CgHaa1 are indicated in the figure, alongside the names of some of the genes clustered in these functional classes.

Although 93% of the genes regulated by CgHaa1 under acetic acid stress have orthologs in *S. cerevisiae*, only 58 have been shown to be regulated by ScHaa1, and of these only 24 are regulated by ScHaa1 specifically under acetic acid stress (Figure 8 and Table S4). It is important to note that this comparison is based on data obtained from transcriptomic studies performed in identical experimental setups (*e.g.*, equivalent inhibitory concentrations of acetic acid, the same pH, and the same sampling time), a condition that is essential for an accurate comparison of regulatory networks (Lelandais *et al.* 2008). The loss and gain of binding sites in target gene promoters has been found to partly underlie differences in orthologous networks in *S. cerevisiae* and *C. glabrata* (Lelandais *et al.* 2008). ScHaa1 was recently found to recognize the HRE motif 5′-(G/C)(A/C)GG(G/C)-3′ (Mira *et al.* 2011). It is possible that CgHaa1 could recognize a DNA motif similar to HRE, considering the high similarity (58% identity at the amino acid level) of ScHaa1 and CgHaa1 at the level of the DNA binding domain. Remarkably, 54 of the genes regulated by CgHaa1 harbor a HRE motif in their promoter that is not present in the promoter region of the corresponding *S. cerevisiae* ortholog (Table S4). Differences in the recognition sequence of CgHaa1 (compared to the HRE motif), nucleosome position, promoter structure, and the interaction of CgHaa1/ScHaa1 with other transcriptional regulators may also underlie the observed differences in the genes that are under the control of the two regulatory networks, as observed in other cases (Lelandais *et al.* 2008). The identification of the genes directly regulated by CgHaa1 by chromatin immunoprecipitation will also provide an essential input to fully understand the structure of the regulatory network controlled by this transcription factor.

The modest overlap of the ScHaa1- and CgHaa1-regulons shows that the two networks have diverged, probably reflecting different control of *C. glabrata* and *S. cerevisiae* over the response to the deleterious toxic effects imposed by acetic acid. Functional clustering of the acetic acid-responsive genes regulated specifically by CgHaa1 shows enrichment of genes involved in the generation of energy, regulation of internal pH, and in cell wall function (Figure 8). In this last functional class, a significant number of adhesins were included (some of which specific of *C. glabrata*), as well as cell wall proteins involved in the uptake of sterols (*CgTIR3*) and the synthesis of β-1,3 and β-1,6 glucans (*CgGAS5*, *CAGL0F03003g*, *CAGL0G09515g*, *CAGL0H10120g*, *CAGL0H07997g*, and *CAGL0I10054g*). Although an involvement of ScHaa1 in adhesion has been shown (Malcher *et al.* 2011), so far no involvement has been reported for in the regulation of glucan synthesis, in the generation of energy, or in the regulation of internal pH homeostasis. Thus, there are likely represent specific functional features of the CgHaa1 network. This observation is interesting, considering that intracellular acidification, energy depletion, and cell wall damage are toxic effects triggered by acetic acid both in *C. glabrata* and *S. cerevisiae* cells (Ullah *et al.* 2013). The mechanisms underlying the regulation of internal pH homeostasis, particularly under stress, are still poorly studied in *C. glabrata*. Our results show that acetic acid induces expression of the plasma membrane proton pump-encoding gene *CgPMA1*, this response being fully dependent on CgHaa1. This observed increase in *CgPMA1* transcription markedly differs from what is observed to occur in *S. cerevisiae*, where no significant upregulation of the *ScPMA1* gene occurs under stress (Piper *et al.* 1997; Carmelo *et al.* 1996; Viegas *et al.* 1994). Besides *CgPMA1*, CgHaa1 also upregulated the expression of several hypothesized regulators of this pump, including CgPmp2 and CgHsp30, the latter being demonstrated herein to be required for maximal *C. glabrata* tolerance to acetic acid. The function of CgHsp30 in *C. glabrata* has not been examined previously, but its *S. cerevisiae* ortholog was implicated in the regulation of the activity and concentration of Pma1 in the plasma membrane (Chattopadhyay *et al.* 2000; Piper *et al.* 1997; Thakur 2010). Our results also show that CgHsp30 expression marginally reduces the internal accumulation of acetic acid inside *C. glabrata* cells. Under acetic acid stress, CgHaa1 also upregulated the expression of *CgYPS2*, *CgYPS4*, and *CgYPS10* genes, which belonging to a family of yapsins shown to be involved in the control of *C. glabrata* internal pH homeostasis (Bairwa and Kaur 2011).

At least 9 predicted adhesins (four of which are specific to *C. glabrata*) were found to be regulated by CgHaa1 in acetic acid-stressed cells. Consistently, CgHaa1 was found to maximize the adherence of *C. glabrata* to vaginal epithelial cells when acetic acid was present in the growth medium. Up to now, only *EPA1* and *EPA6* adhesins have been implicated in *C. glabrata* adherence to vaginal cells (Mundy and Cormack 2009), however, the studies undertaken were carried out in the absence of acetic acid, a condition that is unlikely to occur *in vivo* and that may also alter the profile of adhesins required for adhesion to the vaginal tissue. In that sense, the role of the multiple adhesins upregulated by CgHaa1 in determining *C. glabrata* adherence to vaginal tissue in the presence of acetic acid should be further investigated. Other CgHaa1-regulated genes could also contribute to maximize the adhesion of *C. glabrata* to the vaginal cells, particularly genes that are involved in the generation of energy and in cell wall biosynthesis and maintenance, as these are two physiological functions likely to play a crucial role during the adhesion process.

The protective effect of the CgHaa1-regulated gene *CgTPO3* against acetic acid was correlated with the involvement of this drug efflux pump in reducing the internal accumulation of the acid. Similarly, Δ*Cghaa1* cells were also found to accumulate more acetic acid than wild-type cells, this being consistent with the positive effect of CgHaa1 in inducing *CgTPO3* transcription in response to the acid. It is not possible with the data available to determine if CgTpo3 directly catalyzes the extrusion of acetate/acetic acid or if the observed effect is indirect, resulting from the transport of another substrate(s) whose distribution between the extracellular and intracellular environment may end up affecting acetic acid partitioning, as described for other drug efflux pumps (Sá-Correia *et al.* 2009; Costa *et al.* 2014). CgTpo3 mediates the export of clotrimazole and spermidine, the latter suggested to be its physiological substrate (Costa *et al.* 2013). *C. glabrata* cells are faced with acetate/acetic acid in the gastrointestinal and genitourinary human tracts; therefore, these molecule(s) may also be physiological substrate(s) of CgTpo3. CgTpo3 is the first transporter shown to be involved in the export of acetate/acetic acid in *C. glabrata* since this function could not be attributed to CgAqr1, recently identified as a determinant of *C. glabrata* tolerance to acetic acid (Costa *et al.* 2013).

Overall, our study shows that the response and tolerance of *C. glabrata* to acetic acid at a low pH is largely dependent on the CgHaa1 signaling system, which controls, directly or indirectly, the expression of 75% of the acetic acid-induced genes. Under acetic acid stress, CgHaa1 was found to contribute to the control of internal pH homeostasis by maximizing the expression and activity of the plasma membrane proton pump CgPma1. Through the upregulation of *CgTPO3* and, less significantly, of *CgHSP30*, CgHaa1 also contributed to reduce the internal accumulation of acetic acid. In the presence of acetic acid, CgHaa1 also maximized *C. glabrata* adhesion to vaginal epithelial cells, this being consistent with a positive effect exerted by the transcription factor in the expression of multiple adhesin-encoding genes. Considering that tolerance to organic acids and competition for adhesion sites are essential factors for competitiveness in the vaginal tract, the activity of CgHaa1 is likely to contribute significantly to the success of *C. glabrata* as a vaginal colonizer.

## Supplementary Material

Supplemental material is available online at www.g3journal.org/lookup/suppl/doi:10.1534/g3.116.034660/-/DC1.
