# Supplementary figures and images for: The CgHaa1-Regulon Mediates Response and Tolerance to Acetic Acid Stress in the Human Pathogen Candida glabrata
Source: G3 (Bethesda). 2016 Nov 4;7(1):1–18. doi: 10.1534/g3.116.034660 (PMC5217100; doi:10.1534/g3.116.034660)

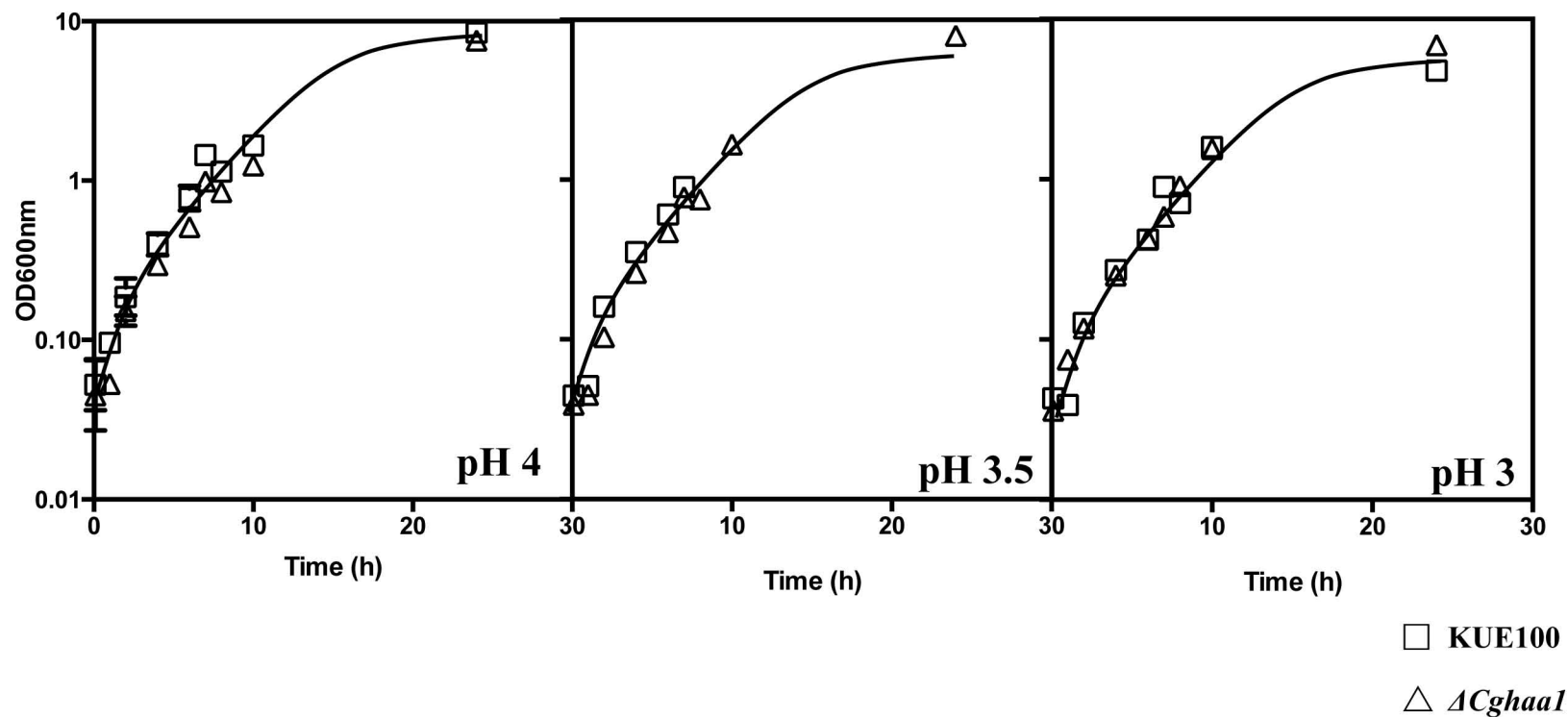

**Figure S1**

Supplement: Supplementary file 1 [file 1FigureS1.pdf]

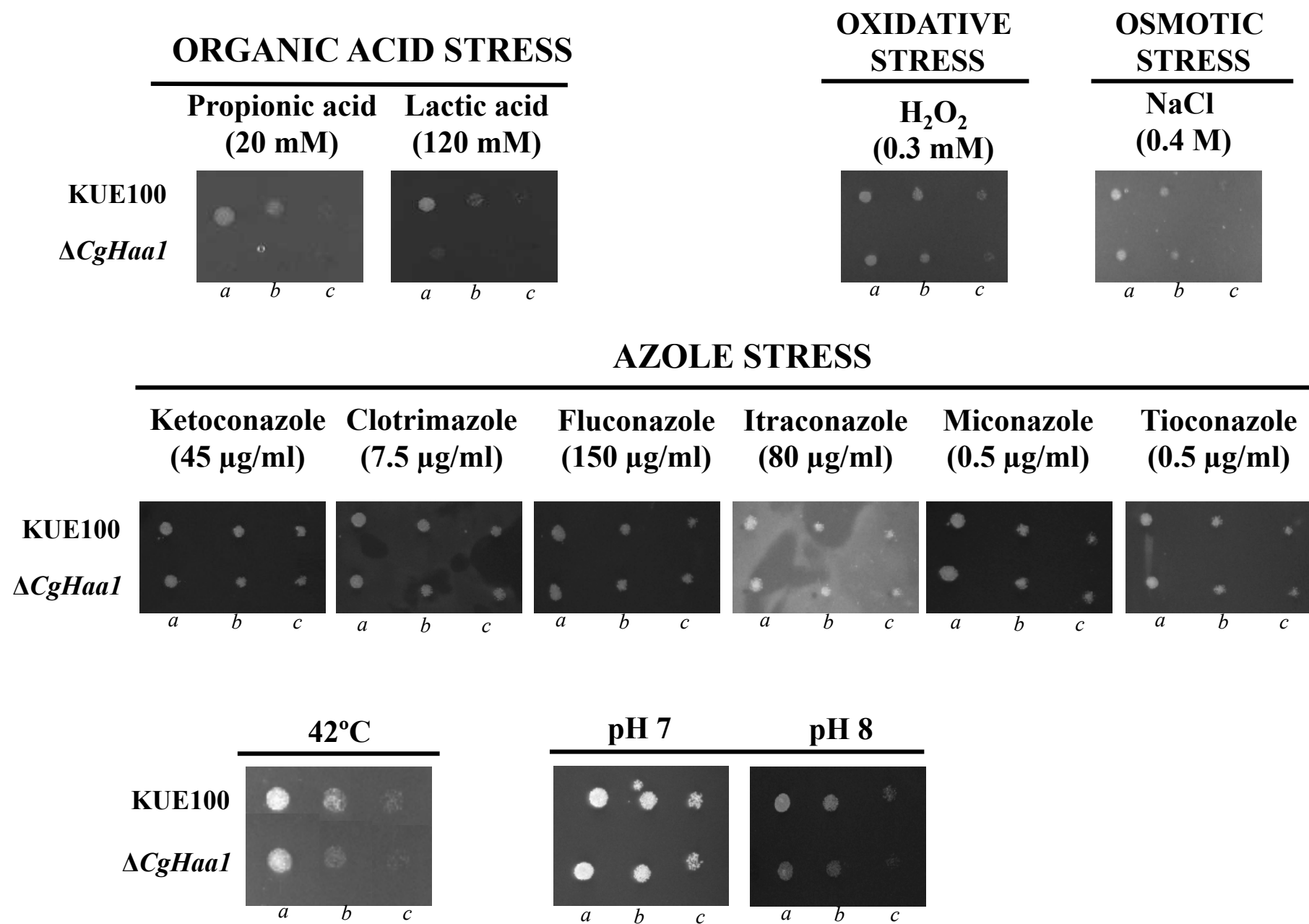

Figure S2

Supplement: Supplementary file 2 [file 1FigureS2.pdf]

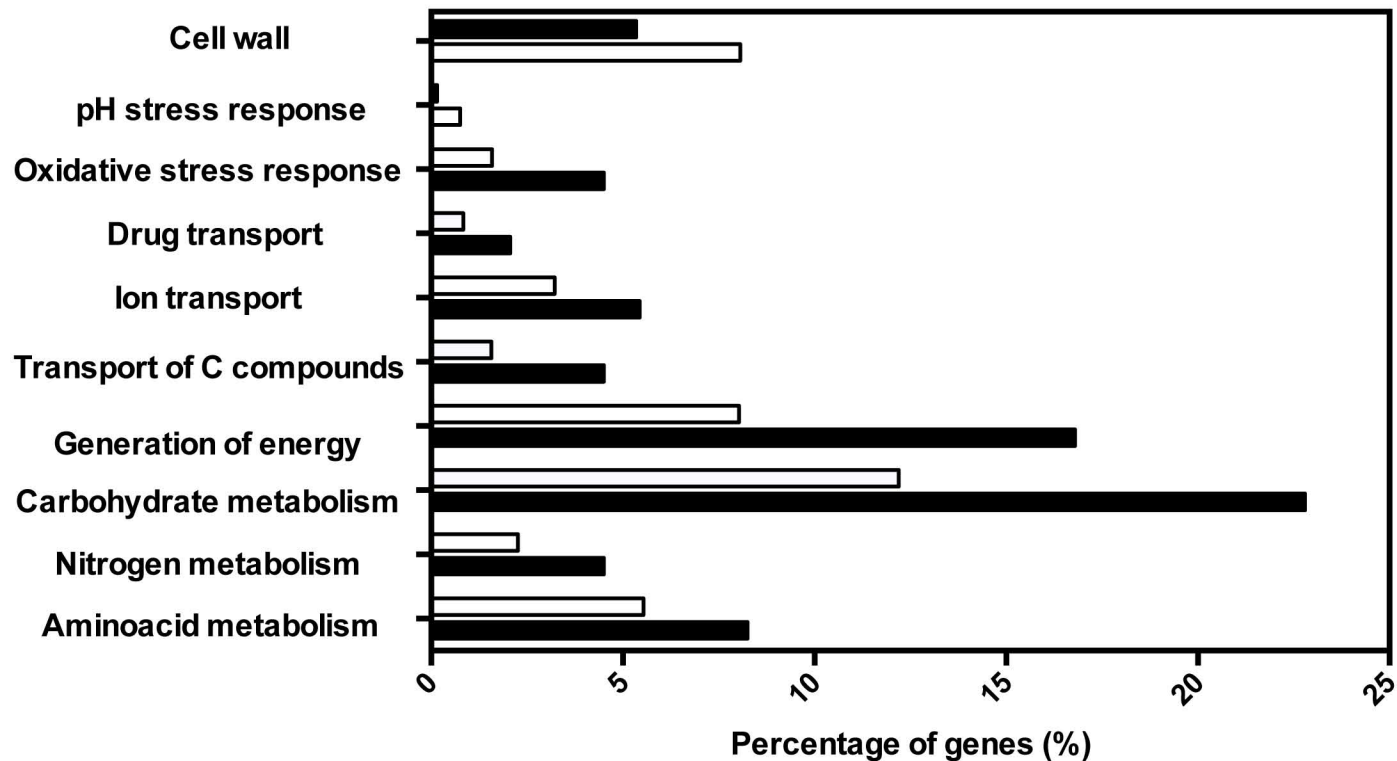

**Figure S3**

Supplement: Supplementary file 3 [file 1FigureS3.pdf]

# CARBON METABOLISM

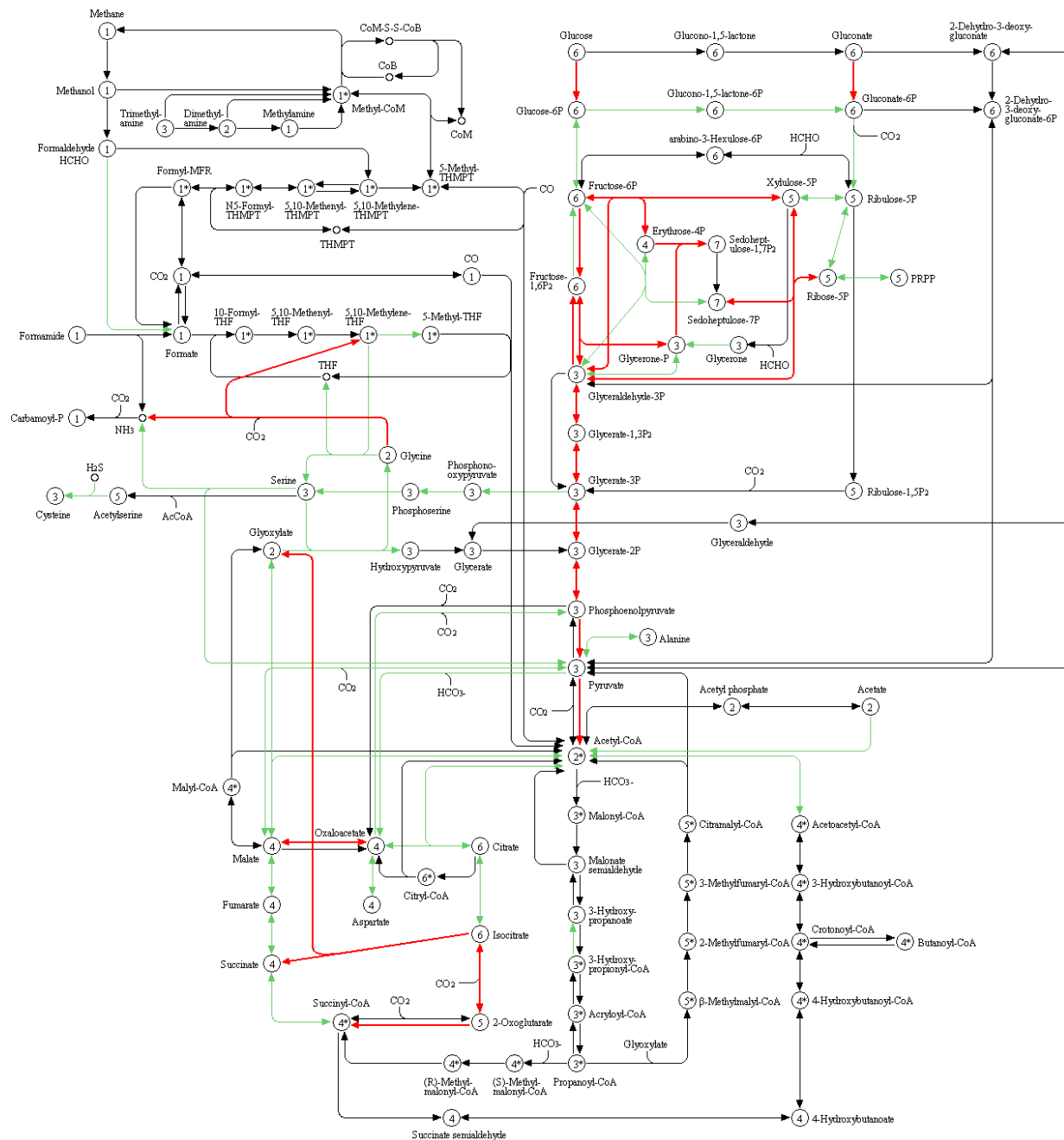

Figure S4

Supplement: Supplementary file 4 [file 1FigureS4.pdf]

## BIOSYNTHESIS OF AMINO ACIDS

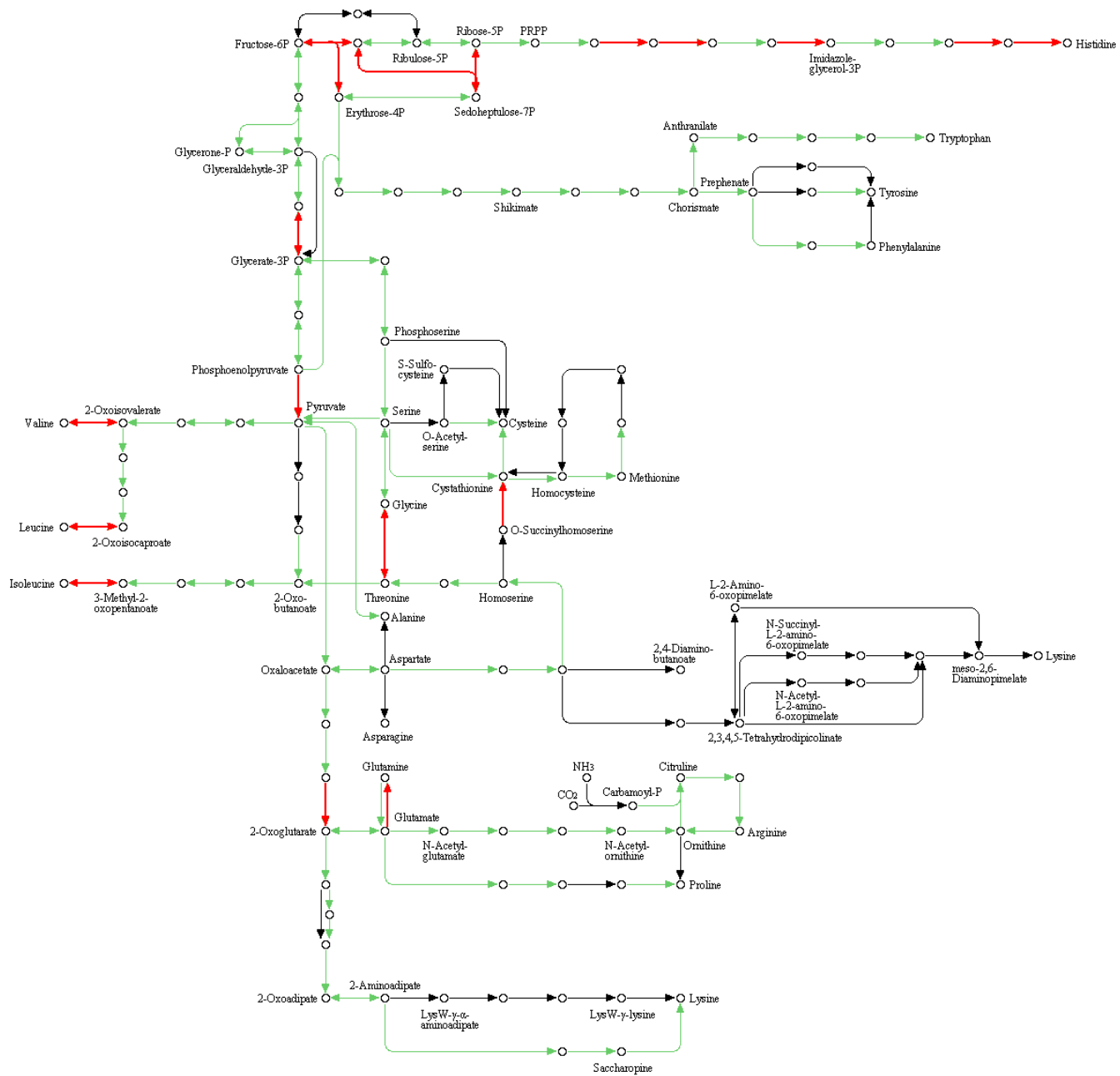

### Figure S5

Supplement: Supplementary file 5 [file 1FigureS5.pdf]
