# Supplementary material for: The CgHaa1-Regulon Mediates Response and Tolerance to Acetic Acid Stress in the Human Pathogen Candida glabrata
Source: G3 (Bethesda). 2016 Nov 4;7(1):1–18. doi: 10.1534/g3.116.034660 (PMC5217100; doi:10.1534/g3.116.034660)
Supplement: Supplementary file 6 [file 1TableS1.pdf]

Supplementary Table S1 – List of the genes found to be up- or down- regulated in response to acetic acid (30 mM, at pH 4.0) in *C. glabrata* KUE100 cells. Genes whose expression increased or decreased (above a 2-fold threshold level) in acetic acid (AC)-challenged cells, in comparison with unstressed cells (CTRL), were selected as described in materials and methods and are here listed. The biological function indicated was based on the information available at Candida Genome Database and/or in the information available for the *S. cerevisiae* orthologue

UP-REGULATED GENES

ORF	Gene name	log_2FC (wt AC/wt CTRL)	p_value	*S. cerevisiae* orthologue	Function
CAGL0G05632g		6,95	1,20E-07	*YDL218W*	Putative protein of unknown function; YDL218W transcription is regulated by Azf1p and induced by starvation and aerobic conditions; expression also induced in cells treated with the mycotoxin patulin
CAGL0I10010g		6,82	1,18E-08	*BTN2*	v-SNARE binding protein that facilitates specific protein retrieval from a late endosome to the Golgi; modulates arginine uptake, possible role in mediating pH homeostasis between the vacuole and plasma membrane H(+)-ATPase
CAGL0M01166g		6,44	2,09E-06	*THI4*	Thiazole synthase, abundant protein involved in the formation of the thiazole moiety of thiamine during thiamine biosynthesis; acts more as a co-substrate rather than an enzyme by providing the sulphur source for thiazole formation; undergoes a single turnover only; required for mitochondrial genome stability in response to DNA damaging agents
CAGL0F08261g		6,11	1,01E-07	*ENO1*	Enolase I, a phosphopyruvate hydratase that catalyzes the conversion of 2-phosphoglycerate to phosphoenolpyruvate during glycolysis and the reverse reaction during gluconeogenesis; expression is repressed in response to glucose
CAGL0G03883g		6,10	1,40E-08	*HSP104*	Disaggregase; Heat shock protein that cooperates with Ydj1p (Hsp40) and Ssa1p (Hsp70) to refold and reactivate previously denatured, aggregated proteins; responsive to stresses including: heat, ethanol, and sodium arsenite; involved in [PSI+] propagation
CAGL0G03289g	*CgSSA3*	6,10	5,22E-07	*SSA4*	Heat shock protein that is highly induced upon stress; plays a role in SRP-dependent cotranslational protein-membrane targeting and translocation; member of the HSP70 family; cytoplasmic protein that concentrates in nuclei upon starvation
CAGL0J00451g		5,66	4,15E-07	-	Putative glyceraldehyde-3-phosphate dehydrogenase
CAGL0C04323g		5,33	8,54E-07	*NTH2*	Putative neutral trehalase, required for thermotolerance and may mediate resistance to other cellular stresses
CAGL0M11902g		5,08	3,85E-08	*FUN19*	Non-essential protein of unknown function; expression induced in response to heat stress
CAGL0M08822g	*CgHSP78*	5,06	9,72E-08	*HSP78*	Oligomeric mitochondrial matrix chaperone that cooperates with Ssc1p in mitochondrial thermotolerance after heat shock; able to prevent the aggregation of misfolded proteins as well as resolubilize protein aggregates
CAGL0E00803g		4,82	4,94E-08	*HSP42*	Small heat shock protein (sHSP) with chaperone activity; forms barrel-shaped oligomers that suppress unfolded protein aggregation; involved in cytoskeleton reorganization after heat shock
CAGL0I07249g		4,71	1,01E-05	*BAG7*	Rho GTPase activating protein (RhoGAP), stimulates the intrinsic GTPase activity of Rho1p, which plays a role in actin cytoskeleton organization and control of cell wall synthesis; structurally and functionally related to Sac7p
CAGL0H03707g		4,64	1,89E-07	*SIS1*	Type II HSP40 co-chaperone that interacts with the HSP70 protein Ssa1p; not functionally redundant with Ydj1p due to due to substrate specificity; shares similarity with bacterial DnaJ proteins
CAGL0E01353g		4,55	2,67E-07	*ZRT2*	Low-affinity zinc transporter of the plasma membrane; transcription is induced under low-zinc conditions by the Zap1p transcription factor
CAGL0G08866g		4,38	1,42E-07	*FKH1*	Forkhead family transcription factor with a minor role in the expression of G2/M phase genes; negatively regulates transcriptional elongation; positive role in chromatin silencing at HML and HMR; regulates donor preference during switching
CAGL0K07337g		4,37	1,63E-06	*HSP30*	Has domain(s) with predicted ion channel activity, role in ion transport and membrane localization
CAGL0C02321g	*CgPHM8*	4,25	1,45E-07	*PHM8*	Lysophosphatidic acid (LPA) phosphatase involved in LPA hydrolysis in response to phosphate starvation; phosphatase activity is soluble and Mg2+ dependent; expression is induced by low phosphate levels and by inactivation of Pho85p
CAGL0H02585g		4,19	1,35E-06	*GAD1*	Glutamate decarboxylase, converts glutamate into gamma-aminobutyric acid (GABA) during glutamate catabolism; involved in response to oxidative stress
CAGL0H10076g		4,12	1,50E-06	*YRO2*	Protein of unknown function with similarity to archaeal rhodopsins; the authentic, non-tagged protein is detected in a phosphorylated state in highly purified mitochondria in high-throughput studies; transcriptionally regulated by Haa1p
CAGL0K10164g		4,08	1,73E-05	*SED1*	Major stress-induced structural GPI-cell wall glycoprotein in stationary-phase cells, associates with translating ribosomes, possible role in mitochondrial genome maintenance; ORF contains two distinct variable minisatellites
CAGL0I09724g		4,07	6,09E-07	-	Has domain(s) with predicted role in transmembrane transport and integral component of membrane localization
CAGL0F04631g		4,03	6,56E-06	*MOH1*	Protein of unknown function, has homology to kinase Snf7p; not required for growth on nonfermentable carbon sources; essential for survival in stationary phase
CAGL0I10384g	*CgTPO3*	3,98	2,68E-06	*TPO2*	Polyamine transport protein specific for spermine; localizes to the plasma membrane; transcription of TPO2 is regulated by Haa1p; member of the major facilitator superfamily
CAGL0J06050g		3,97	3,26E-06	*YGP1*	Cell wall-related secretory glycoprotein; induced by nutrient deprivation-associated growth arrest and upon entry into stationary phase; may be involved in adaptation prior to stationary phase entry; has similarity to Sps100p
CAGL0H00704g		3,95	5,98E-07	*ICY2*	Protein of unknown function; mobilized into polysomes upon a shift from a fermentable to nonfermentable carbon source; potential Cdc28p substrate
CAGL0G08624g	*CgQDR2*	3,92	2,06E-06	*QDR1*	Multidrug transporter of the major facilitator superfamily, required for resistance to quinidine, ketoconazole, fluconazole, and barban
CAGL0F04521g		3,84	8,29E-07	*ECM13*	Non-essential protein of unknown function; induced by treatment with 8-methoxypsoralen and UVA irradiation
CAGL0G06182g		3,72	2,98E-06	*YHR131C*	Putative protein of unknown function; GFP-fusion protein localizes to the cytoplasm; overexpression causes cell cycle delay or arrest; contains a PH domain and binds phosphatidylinositols and other lipids in a large-scale study
CAGL0L07722g	*CgPGK1*	3,69	2,65E-05	*PGK1*	3-phosphoglycerate kinase, catalyzes transfer of high-energy phosphoryl groups from the acyl phosphate of 1,3-bisphosphoglycerate to ADP to produce ATP; key enzyme in glycolysis and gluconeogenesis
CAGL0A01804g	*CgHXT1*	3,65	2,98E-06	*HXT1*	Low-affinity glucose transporter of the major facilitator superfamily, expression is induced by Hxk2p in the presence of glucose and repressed by Rgt1p when glucose is limiting

CAGL0H04279g	*MT-IIB*	3,65	2,67E-05	-	Copper-binding metallothionein, involved in sequestration of metal ions
CAGL0F04895g		3,61	4,57E-06	*GPH1*	Non-essential glycogen phosphorylase required for the mobilization of glycogen, activity is regulated by cyclic AMP-mediated phosphorylation, expression is regulated by stress-response elements and by the HOG MAP kinase pathway
CAGL0K12254g		3,60	1,98E-06	*VID24*	Peripheral membrane protein located at Vid (vacuole import and degradation) vesicles; regulates fructose-1,6-bisphosphatase (FBPase) targeting to the vacuole; promotes proteasome-dependent catabolite degradation of FBPase
CAGL0K04301g		3,47	1,20E-05	*FMP48*	Putative protein of unknown function; the authentic, non-tagged protein is detected in highly purified mitochondria in high-throughput studies; induced by treatment with 8-methoxypsoralen and UVA irradiation
CAGL0B00616g		3,46	3,39E-05	*SPS22*	Protein of unknown function, redundant with Sps2p for the organization of the beta-glucan layer of the spore wall
CAGL0E06358g	*CgGPM1*	3,46	1,18E-06	*GPM1*	Tetrameric phosphoglycerate mutase, mediates the conversion of 3-phosphoglycerate to 2-phosphoglycerate during glycolysis and the reverse reaction during gluconeogenesis
CAGL0K03421g		3,39	2,14E-05	*PGM2*	Phosphoglucomutase, catalyzes the conversion from glucose-1-phosphate to glucose-6-phosphate, which is a key step in hexose metabolism; functions as the acceptor for a Glc-phosphotransferase
CAGL0L08008g		3,39	1,40E-04	*PMP1*	Small single-membrane span proteolipid that functions as a regulatory subunit of the plasma membrane H(+)-ATPase Pma1p, forms unique helix and positively charged cytoplasmic domain that is able to specifically segregate phosphatidylserines
CAGL0H02101g		3,31	8,50E-05	*RTC3*	Protein of unknown function involved in RNA metabolism; has structural similarity to SBDS, the human protein mutated in Shwachman-Diamond Syndrome (the yeast SBDS ortholog = SDO1); null mutation suppresses cdc13-1 temperature sensitivity
CAGL0J10296g		3,30	1,21E-06	*APJ1*	Putative chaperone of the HSP40 (DNAJ) family; overexpression interferes with propagation of the [Psi+] prion; the authentic, non-tagged protein is detected in highly purified mitochondria in high-throughput studies
CAGL0G05269g		3,27	1,70E-05	*FMP16*	Putative protein of unknown function; proposed to be involved in responding to conditions of stress; the authentic, non-tagged protein is detected in highly purified mitochondria in high-throughput studies
CAGL0I09702g		3,24	2,15E-06	*MCH5*	Plasma membrane riboflavin transporter; facilitates the uptake of vitamin B2; required for FAD-dependent processes; sequence similarity to mammalian monocarboxylate permeases, however mutants are not deficient in monocarboxylate transport
CAGL0G09383g	*CgTDH3*	3,23	5,64E-06	*TDH3*	Glyceraldehyde-3-phosphate dehydrogenase, isozyme 3, involved in glycolysis and gluconeogenesis; tetramer that catalyzes the reaction of glyceraldehyde-3-phosphate to 1,3 bis-phosphoglycerate; detected in the cytoplasm and cell wall
CAGL0E05148g		3,23	3,68E-06	*AMS1*	Vacuolar alpha mannosidase, involved in free oligosaccharide (fOS) degradation; delivered to the vacuole in a novel pathway separate from the secretory pathway
CAGL0F08745g		3,22	7,88E-05	*STF2*	Protein involved in resistance to dessication stress; Stf2p exhibits antioxidant properties, and its overexpression prevents ROS accumulation and apoptosis; binds to the F0 sector of mitochondrial F1F0 ATPase in vitro and is proposed to modulate the inhibitory action of Inh1p and Stf1p
CAGL0M02167g		3,22	2,99E-06	*PRM4*	Pheromone-regulated protein proposed to be involved in mating; predicted to have 1 transmembrane segment; transcriptionally regulated by Ste12p during mating and by Cat8p during the diauxic shift
CAGL0G02057g		3,19	2,48E-05	*YKR075C*	Protein of unknown function; similar to YOR062Cp and Reg1p; expression regulated by glucose and Rgt1p; GFP-fusion protein is induced in response to the DNA-damaging agent MMS
CAGL0H04851g		3,18	4,78E-06	*PPZ1*	Serine/threonine protein phosphatase Z, isoform of Ppz2p; involved in regulation of potassium transport, which affects osmotic stability, cell cycle progression, and halotolerance
CAGL0J01595g	*CgGLM6*	3,18	4,74E-06	*YPR015C*	Putative protein of unknown function; overexpression causes a cell cycle delay or arrest
CAGL0A03102g	*CgARO10*	3,17	2,47E-06	*ARO10*	Phenylpyruvate decarboxylase, catalyzes decarboxylation of phenylpyruvate to phenylacetaldehyde, which is the first specific step in the Ehrlich pathway
CAGL0A02002g		3,16	7,20E-06	*YOL024W*	Putative protein of unknown function; predicted to have thiol-disulfide oxidoreductase active site
CAGL0H00418g		3,16	1,31E-05	-	Unknown
CAGL0I00418g		3,15	4,34E-05	*OLE1*	Delta(9) fatty acid desaturase, required for monounsaturated fatty acid synthesis and for normal distribution of mitochondria
CAGL0K04719g		3,15	2,17E-06	*YNL208W*	Protein of unknown function; may interact with ribosomes, based on co-purification experiments; authentic, non-tagged protein is detected in purified mitochondria in high-throughput studies; potential orthologs found in other fungi
CAGL0G03795g	*CgSSA1*	3,13	4,17E-05	*SSA2*	ATP binding protein involved in protein folding and vacuolar import of proteins; member of heat shock protein 70 (HSP70) family; associated with the chaperonin-containing T-complex; present in the cytoplasm, vacuolar membrane and cell wall; 98% identical with Ssa1p, but subtle differences between the two proteins provide functional specificity with respect to propagation of yeast [URE3] prions and vacuolar-mediated degradations of gluconeogenesis enzymes
CAGL0I05934g		3,12	2,01E-06	*YJL144W*	Cytoplasmic hydrophilin essential in dessication-rehydration process; expression induced by osmotic stress, starvation and during stationary phase; GFP-fusion protein is induced by the DNA-damaging agent MMS
CAGL0K07590g	*CgMYO3*	3,11	3,72E-06	*MYO3*	One of two type I myosins; localizes to actin cortical patches; deletion of MYO3 has little effect on growth, but myo3 myo5 double deletion causes severe defects in growth and actin cytoskeleton organization
CAGL0E05566g		3,11	1,28E-05	*TYE7*	Serine-rich protein that contains a basic-helix-loop-helix (bHLH) DNA binding motif; binds E-boxes of glycolytic genes and contributes to their activation; may function as a transcriptional activator in Ty1-mediated gene expression
CAGL0F01111g		3,09	1,64E-06	*OPI10*	Protein with a possible role in phospholipid biosynthesis, based on inositol-excreting phenotype of the null mutant and its suppression by exogenous choline
CAGL0I05148g	*CgDLD1*	3,07	5,89E-05	*DLD1*	D-lactate dehydrogenase, oxidizes D-lactate to pyruvate, transcription is heme-dependent, repressed by glucose, and derepressed in ethanol or lactate; located in the mitochondrial inner membrane
CAGL0G02563g		3,06	3,38E-05	*NA*	NA
CAGL0G03179g		3,06	2,72E-06	*ASK10*	Component of RNA polymerase II holoenzyme, phosphorylated in response to oxidative stress; has a role in destruction of Ssn8p; proposed to function in activation of the glycerol channel Fps1p; paralogous to Rgc1p
CAGL0I02486g	*CgENO1*	3,06	2,02E-05	*ENO2*	Enolase II, a phosphopyruvate hydratase that catalyzes the conversion of 2-phosphoglycerate to phosphoenolpyruvate during glycolysis and the reverse reaction during gluconeogenesis; expression is induced in response to glucose
CAGL0K03685g		3,05	5,72E-05	*PKR1*	V-ATPase assembly factor, functions with other V-ATPase assembly factors in the ER to efficiently assemble the V-ATPase membrane sector (V0)

CAGL0J04466g		3,04	1,97E-06	*PUN1*	Plasma membrane protein with a role in cell wall integrity; co-localizes with Sur7p in punctate membrane patches; null mutant displays decreased thermotolerance; transcription induced upon cell wall damage and metal ion stress
CAGL0C00275g	*CgHSP31*	3,04	1,33E-05	-	Putative cysteine protease
CAGL0M13651g		3,04	3,07E-06	*PRC1*	Vacuolar carboxypeptidase Y (proteinase C; CPY), broad-specificity C-terminal exopeptidase involved in non-specific protein degradation in the vacuole; member of the serine carboxypeptidase family
CAGL0C02893g		3,01	5,78E-06	*HRK1*	Protein kinase implicated in activation of the plasma membrane H(+)-ATPase Pma1p in response to glucose metabolism; plays a role in ion homeostasis
CAGL0M11660g		2,99	3,55E-06	-	Has domain(s) with predicted hydrolase activity
CAGL0M07920g	*CgPDC*	2,98	1,83E-05	*PDC1*	Major of three pyruvate decarboxylase isozymes, key enzyme in alcoholic fermentation, decarboxylates pyruvate to acetaldehyde; subject to glucose-, ethanol-, and autoregulation; involved in amino acid catabolism
CAGL0H03971g		2,98	7,55E-06	*YCP4*	Protein of unknown function, has sequence and structural similarity to flavodoxins; predicted to be palmitoylated; the authentic, non-tagged protein is detected in highly purified mitochondria in high-throughput studies
CAGL0A00495g	*CgPMA1*	2,98	8,50E-06	*PMA1*	Plasma membrane H+-ATPase, pumps protons out of the cell; major regulator of cytoplasmic pH and plasma membrane potential; P2-type ATPase; Hsp30p plays a role in Pma1p regulation; interactions with Std1p appear to propagate [GAR+]
CAGL0A01870g		2,97	1,36E-05	-	Has domain(s) with predicted integral component of membrane localization
CAGL0I06644g		2,94	8,14E-04	*SPI1*	GPI-anchored cell wall protein involved in weak acid resistance; basal expression requires Msn2p/Msn4p; expression is induced under conditions of stress and during the diauxic shift; similar to Sed1p
CAGL0I06182g	*CgPIR2*	2,94	2,06E-05	*HSP150*	O-mannosylated heat shock protein that is secreted and covalently attached to the cell wall via beta-1,3-glucan and disulfide bridges; required for cell wall stability; induced by heat shock, oxidative stress, and nitrogen limitation
CAGL0M06897g		2,91	8,50E-05	*YNL024C*	Putative methyltransferase; has seven beta-strand methyltransferase motif; green fluorescent protein (GFP)-fusion protein localizes to the cytoplasm; despite similarity to methyltransferases, null mutant does not display alterations in lysine methylation pattern
CAGL0J07568g		2,91	1,05E-05	*LAP3*	Cysteine aminopeptidase with homocysteine-thiolactonase activity; protects cells against homocysteine toxicity; has bleomycin hydrolase activity in vitro; transcription is regulated by galactose via Gal4p; orthologous to human BLMH
CAGL0H07469g		2,90	3,42E-06	*ICS2*	Protein of unknown function; null mutation does not confer any obvious defects in growth, spore germination, viability, or carbohydrate utilization
CAGL0G05698g	*CgGDH2*	2,89	1,19E-05	*GDH2*	NAD(+)-dependent glutamate dehydrogenase, degrades glutamate to ammonia and alpha-ketoglutarate; expression sensitive to nitrogen catabolite repression and intracellular ammonia levels
CAGL0M08492g	*CgPIR3*	2,88	6,55E-06	*PIR1*	O-glycosylated protein required for cell wall stability; attached to the cell wall via beta-1,3-glucan; mediates mitochondrial translocation of Apn1p; expression regulated by the cell integrity pathway and by Swi5p during the cell cycle
CAGL0A01716g		2,87	6,32E-05	*PNC1*	Nicotinamidase that converts nicotinamide to nicotinic acid as part of the NAD(+) salvage pathway, required for life span extension by calorie restriction; PNC1 expression responds to all known stimuli that extend replicative life span
CAGL0E03630g		2,84	1,52E-05	*RIM4*	Putative RNA-binding protein required for the expression of early and middle sporulation genes
CAGL0L02497g	*CgFBA1*	2,84	8,94E-06	*FBA1*	Fructose 1,6-bisphosphate aldolase, required for glycolysis and gluconeogenesis; catalyzes conversion of fructose 1,6 bisphosphate to glyceraldehyde-3-P and dihydroxyacetone-P; locates to mitochondrial outer surface upon oxidative stress
CAGL0J11462g		2,84	6,38E-06	*YNL190W*	Hydrophilin essential in dessication-rehydration process; cell wall protein; contains a putative GPI-attachment site
CAGL0G03267g		2,80	7,17E-06	*AST2*	Similar to lipid raft associated protein Ast1p; similarly to Ast1p, overexpression restores Pma1p localization to lipid rafts which is required for targeting of Pma1p to the plasma membrane; sometimes classified in the medium-chain dehydrogenase/reductases (MDRs) superfamily
CAGL0A01650g		2,80	3,76E-06	*ECL1*	Protein of unknown function, affects chronological lifespan; induced by iron homeostasis transcription factor Aft2p; multicopy suppressor of temperature sensitive hsf1 mutant; induced by treatment with 8-methoxypsoralen and UVA irradiation
CAGL0E01749g	*CgYPS4*	2,78	3,45E-05	-	Putative aspartic protease; member of a YPS gene cluster that is required for virulence in mice; induced in response to low pH and high temperature
CAGL0L06424g		2,77	1,45E-04	*CCCW12*	Predicted GPI-linked adhesin-like protein
CAGL0F06413g		2,77	2,33E-03	*FET3*	Ferro-O2-oxidoreductase required for high-affinity iron uptake and involved in mediating resistance to copper ion toxicity, belongs to class of integral membrane multicopper oxidases
CAGL0H08393g		2,75	2,62E-04	*BAP2*	High-affinity leucine permease, functions as a branched-chain amino acid permease involved in the uptake of leucine, isoleucine and valine; contains 12 predicted transmembrane domains
CAGL0D01270g		2,75	1,90E-04	-	Unknown
CAGL0M12034g	*CgPYK1*	2,72	1,08E-04	*CDC19*	Pyruvate kinase, functions as a homotetramer in glycolysis to convert phosphoenolpyruvate to pyruvate, the input for aerobic (TCA cycle) or anaerobic (glucose fermentation) respiration
CAGL0K02145g		2,71	3,82E-06	*YER130C*	Protein of unknown function; transcription is regulated by Haa1p, Sok2p and Zap1p transcriptional activators; computational analysis suggests a role as a transcription factor; C. albicans homolog (MNL1) plays a role in adaptation to stress
CAGL0L05786g		2,71	2,16E-05	*YPR013C*	Putative zinc finger protein; YPR013C is not an essential gene
CAGL0F03707g		2,71	4,31E-06	*HRK1*	Protein kinase implicated in activation of the plasma membrane H(+)-ATPase Pma1p in response to glucose metabolism; plays a role in ion homeostasis
CAGL0D03322g		2,70	6,91E-06	*IZH3*	Membrane protein involved in zinc ion homeostasis, member of the four-protein IZH family, expression induced by zinc deficiency; deletion reduces sensitivity to elevated zinc and shortens lag phase, overexpression reduces Zap1p activity
CAGL0G09603g		2,69	6,69E-05	*YOR186W*	Putative protein of unknown function; proper regulation of expression during heat stress is sphingolipid-dependent
CAGL0M12430g	*CgRHR2*	2,68	1,32E-05	*RHR2*	Constitutively expressed isoform of DL-glycerol-3-phosphatase; involved in glycerol biosynthesis, induced in response to both anaerobic and, along with the Hor2p/Gpp2p isoform, osmotic stress
CAGL0G01474g		2,66	3,57E-05	*BOP3*	Protein of unknown function, potential Cdc28p substrate; overproduction confers resistance to methylmercury
CAGL0J03080g		2,65	7,58E-04	*RGI1*	Protein of unknown function involved in energy metabolism under respiratory conditions; protein abundance is increased upon intracellular iron depletion
CAGL0K07634g	*CgGAT1*	2,65	3,38E-05	*GAT1*	Transcriptional activator of genes involved in nitrogen catabolite repression; contains a GATA-1-type zinc finger DNA-binding motif; activity and localization regulated by nitrogen limitation and Ure2p

CAGL0L08030g		2,64	1,44E-05	*SLM5*	Mitochondrial asparaginyl-tRNA synthetase
CAGL0H08844g		2,64	2,49E-04	*DDR48*	DNA damage-responsive protein, expression is increased in response to heat-shock stress or treatments that produce DNA lesions; contains multiple repeats of the amino acid sequence NNNDSYGS
CAGL0I01342g		2,63	3,53E-05	*GLY1*	Threonine aldolase, catalyzes the cleavage of L-allo-threonine and L-threonine to glycine; involved in glycine biosynthesis
CAGL0F07117g		2,63	1,47E-05	*GPG1*	Proposed gamma subunit of the heterotrimeric G protein that interacts with the receptor Gpr1p; involved in regulation of pseudohyphal growth; requires Gpb1p or Gpb2p to interact with Gpa2p; overproduction causes prion curing
CAGL0L03696g		2,62	2,36E-05	*ECM3*	Non-essential protein of unknown function; involved in signal transduction and the genotoxic response; induced rapidly in response to treatment with 8-methoxypsoralen and UVA irradiation
CAGL0J04202g	*CgHSP12*	2,61	1,24E-04	*HSP12*	Plasma membrane protein involved in maintaining membrane organization in stress conditions; induced by heat shock, oxidative stress, osmostress, stationary phase, glucose depletion, oleate and alcohol; regulated by HOG and Ras-Pka pathways
CAGL0G04081g		2,60	4,79E-05	*THI73*	Putative plasma membrane permease proposed to be involved in carboxylic acid uptake and repressed by thiamine; substrate of Dbf2p/Mob1p kinase; transcription is altered if mitochondrial dysfunction occurs
CAGL0H08327g	*CgTPI1*	2,60	9,36E-06	*TPI1*	Triose phosphate isomerase, abundant glycolytic enzyme; mRNA half-life is regulated by iron availability; transcription is controlled by activators Reb1p, Gcr1p, and Rap1p through binding sites in the 5' non-coding region; inhibition of Tpi1p activity by PEP (phosphoenolpyruvate) stimulates redox metabolism in respiring cells; E104D mutation in human TPI causes a rare autosomal disease
CAGL0C03740g		2,59	6,57E-06	*MIT1*	Transcriptional regulator of pseudohyphal growth; protein with sequence similarity to S. pombe gti1+ (gluconate transport inducer 1) and C. albicans Wor1
CAGL0B03619g		2,58	1,08E-03	*PRB1*	Vacuolar proteinase B (yscB), a serine protease of the subtilisin family; involved in protein degradation in the vacuole and required for full protein degradation during sporulation; activity inhibited by Pbi2p
CAGL0I05874g		2,57	1,18E-04	*NA*	NA
CAGL0J04026g		2,56	5,55E-06	*HER1*	Protein of unknown function required for proliferation or remodeling of the ER that is caused by overexpression of Hmg2p; may interact with ribosomes, based on co-purification experiments
CAGL0I04246g		2,56	1,07E-04	*SUT1*	Transcription factor of the Zn[II]2Cys6 family involved in sterol uptake; involved in induction of hypoxic gene expression
CAGL0E01837g	*CgYPS9*	2,56	1,38E-05	-	Putative aspartic protease; predicted GPI-anchor; member of a YPS gene cluster that is required for virulence in mice; expression induced at high temperature
CAGL0H08778g		2,55	1,07E-05	*PUF2*	Member of the PUF protein family, which is defined by the presence of Pumilio homology domains that confer RNA binding activity; preferentially binds mRNAs encoding membrane-associated proteins
CAGL0E04774g		2,55	2,25E-05	*YDR222W*	Protein of unknown function; green fluorescent protein (GFP)-fusion protein localizes to the cytoplasm in a punctate pattern
CAGL0F08371g	*CgTNA1*	2,54	1,74E-04	*TNA1*	High affinity nicotinic acid plasma membrane permease, responsible for uptake of low levels of nicotinic acid; expression of the gene increases in the absence of extracellular nicotinic acid or para-aminobenzoate (PABA)
CAGL0D01276g		2,51	8,60E-06	*OPY2*	Integral membrane protein that functions in the signaling branch of the high-osmolarity glycerol (HOG) pathway; interacts with Ste50p; overproduction blocks cell cycle arrest in the presence of mating pheromone
CAGL0M11000g		2,50	2,79E-03	*YNR034W-A*	Putative protein of unknown function; expression is regulated by Msn2p/Msn4p
CAGL0G03531g		2,49	3,59E-05	*SPR6*	Protein of unknown function, expressed during sporulation; not required for sporulation, but gene exhibits genetic interactions with other genes required for sporulation
CAGL0K09372g		2,47	9,76E-06	*MIG2*	Zinc finger transcriptional repressor; cooperates with Mig1p in glucose-induced repression of many genes including SUC2 (invertase); binds to Mig1p-binding sites in SUC2 promoter; under low glucose conditions Mig2p relocalizes to mitochondrion, where it regulates morphology, interacts with Ups1p and antagonizes mitochondrial fission factor, Dnm1p, indicative of a role in promoting mitochondrial fusion or regulating morphology
CAGL0J00715g		2,46	2,26E-05	*YHR022C*	Putative protein of unknown function; YHR022C is not an essential gene
CAGL0H01375g	*CgSUR2*	2,44	9,18E-05	*SUR2*	Sphinganine C4-hydroxylase, catalyses the conversion of sphinganine to phytosphingosine in sphingolipid biosyntheis
CAGL0I05060g		2,43	3,58E-04	*DOT6*	Protein involved in rRNA and ribosome biogenesis; binds polymerase A and C motif; subunit of the RPD3L histone deacetylase complex; similar to Tod6p; has chromatin specific SANT domain; involved in telomeric gene silencing and filamentation
CAGL0F08041g	*CgPFK1*	2,43	3,45E-05	*PFK1*	Alpha subunit of heterooctameric phosphofructokinase involved in glycolysis, indispensable for anaerobic growth, activated by fructose-2,6-bisphosphate and AMP, mutation inhibits glucose induction of cell cycle-related genes
CAGL0K03663g		2,43	4,24E-05	*YMR122W-A*	Protein of unknown function; green fluorescent protein (GFP)-fusion protein localizes to the cytoplasm and endoplasmic reticulum
CAGL0H02563g		2,42	4,65E-05	*HOR7*	Protein of unknown function; overexpression suppresses Ca2+ sensitivity of mutants lacking inositol phosphorylceramide mannosyltransferases Csg1p and Csh1p; transcription is induced under hyperosmotic stress and repressed by alpha factor
CAGL0D05566g		2,42	4,98E-05	*YEH1*	Steryl ester hydrolase, one of three gene products (Yeh1p, Yeh2p, Tgl1p) responsible for steryl ester hydrolase activity and involved in sterol homeostasis; localized to lipid particle membranes
CAGL0E01793g	*CgYPS6*	2,40	6,59E-05	*NA*	NA
CAGL0G04763g		2,38	2,54E-04	*RGS2*	Negative regulator of glucose-induced cAMP signaling; directly activates the GTPase activity of the heterotrimeric G protein alpha subunit Gpa2p
CAGL0K06853g		2,38	1,11E-04	*PCS60*	Peroxisomal protein that binds AMP and mRNA, localizes to both the peroxisomal peripheral membrane and matrix, expression is highly inducible by oleic acid, similar to E. coli long chain acyl-CoA synthetase
CAGL0E04312g		2,38	5,98E-05	*STP2*	Transcription factor, activated by proteolytic processing in response to signals from the SPS sensor system for external amino acids; activates transcription of amino acid permease genes
CAGL0H02519g		2,37	2,07E-04	*YMR253C*	Putative protein of unknown function; green fluorescent protein (GFP)-fusion protein localizes to the cytoplasm in a punctate pattern; YMR253C is not an essential gene
CAGL0M07634g		2,37	2,00E-04	*SOK2*	Nuclear protein that plays a regulatory role in the cyclic AMP (cAMP)-dependent protein kinase (PKA) signal transduction pathway; negatively regulates pseudohyphal differentiation; homologous to several transcription factors
CAGL0M00748g	*CgECM7*	2,36	3,02E-05	*ECM7*	Non-essential putative integral membrane protein with a role in calcium uptake; mutant has cell wall defects and Ca+ uptake deficiencies; transcription is induced under conditions of zinc deficiency

CAGL0G08844g		2,35	2,30E-04	*ASG1*	Zinc cluster protein proposed to function as a transcriptional regulator involved in the stress response; null mutants have a respiratory deficiency, calcofluor white sensitivity and slightly increased cycloheximide resistance
CAGL0B01875g		2,33	2,22E-04	*COX26*	Putative protein of unknown function; copurifies with respiratory chain supercomplexes composed of Complex III (ubiquinol-cytochrome c reductase) and Complex IV (cytochrome c oxidase)
CAGL0E01881g	*CgYPS11*	2,32	5,58E-05	-	Putative aspartic protease; member of a YPS gene cluster that is required for virulence in mice; induced in response to low pH and high temperature
CAGL0F08217g		2,30	6,42E-04	*YGR250C*	Putative RNA binding protein; localizes to stress granules induced by glucose deprivation; interacts with Rbg1p in a two-hybrid
CAGL0J10846g		2,30	1,49E-05	*PCL5*	Cyclin, interacts with and phosphorylated by Pho85p cyclin-dependent kinase (Cdk), induced by Gcn4p at level of transcription, specifically required for Gcn4p degradation, may be sensor of cellular protein biosynthetic capacity
CAGL0J06028g	*CgMEP2*	2,27	5,81E-03	*MEP2*	Ammonium permease involved in regulation of pseudohyphal growth; belongs to a ubiquitous family of cytoplasmic membrane proteins that transport only ammonium (NH4+); expression is under the nitrogen catabolite repression regulation
CAGL0D04026g		2,27	1,36E-04	*UGA1*	Gamma-aminobutyrate (GABA) transaminase (4-aminobutyrate aminotransferase) involved in the 4-aminobutyrate and glutamate degradation pathways; required for normal oxidative stress tolerance and nitrogen utilization
CAGL0L06072g		2,26	5,51E-05	*YER130C*	Protein of unknown function; transcription is regulated by Haa1p, Sok2p and Zap1p transcriptional activators; computational analysis suggests a role as a transcription factor; C. albicans homolog (MNL1) plays a role in adaptation to stress
CAGL0E05654g		2,24	3,25E-05	*PGC1*	Phosphatidyl Glycerol phospholipase C; regulates the phosphatidylglycerol (PG) content via a phospholipase C-type degradation mechanism; contains glycerophosphodiester phosphodiesterase motifs
CAGL0J09900g		2,23	2,74E-03	*POR1*	Mitochondrial porin (voltage-dependent anion channel), outer membrane protein required for the maintenance of mitochondrial osmotic stability and mitochondrial membrane permeability; phosphorylated
CAGL0D05082g		2,21	6,76E-05	*UBI4*	Ubiquitin, becomes conjugated to proteins, marking them for selective degradation via the ubiquitin-26S proteasome system; essential for the cellular stress response; encoded as a polyubiquitin precursor comprised of 5 head-to-tail repeats
CAGL0K07458g		2,20	8,61E-05	*YPK1*	Serine/threonine protein kinase; phosphorylates and downregulates flippase activator Fpk1p; also inactivates Orm1p and Orm2p (inhibitors of serine:palmitoyl-coenzyme A transferase) by phosphorylation in response to compromised sphingolipid synthesis; mutations affect receptor-mediated endocytosis and sphingolipid-mediated and cell integrity signaling pathways; homolog of mammalian kinase SGK
CAGL0K07007g		2,20	4,98E-04	*YBR238C*	Mitochondrial membrane protein with similarity to Rmd9p; not required for respiratory growth but causes a synthetic respiratory defect in combination with rmd9 mutations; transcriptionally up-regulated by TOR; deletion increases life span
CAGL0K02651g		2,19	3,01E-05	*YHL008C*	Putative protein of unknown function, may be involved in the uptake of chloride ions; does not appear to be involved in monocarboxylic acid transport; green fluorescent protein (GFP)-fusion protein localizes to the vacuole
CAGL0H08888g		2,18	1,81E-04	*FLC3*	Putative FAD transporter, similar to Flc1p and Flc2p; localized to the ER
CAGL0J10494g		2,18	1,26E-04	*APT2*	Apparent pseudogene, not transcribed or translated under normal conditions; encodes a protein with similarity to adenine phosphoribosyltransferase, but artificially expressed protein exhibits no enzymatic activity
CAGL0F03641g		2,18	2,33E-03	*NA*	NA
CAGL0A02046g		2,17	1,25E-04	*ROD1*	Membrane protein that binds the ubiquitin ligase Rsp5p via its 2 PY motifs; overexpression confers resistance to the GST substrate o-dinitrobenzene,zinc, and calcium; proposed to regulate the endocytosis of plasma membrane proteins
CAGL0F04917g		2,17	1,39E-04	*PIG1*	Putative targeting subunit for the type-1 protein phosphatase Glc7p that tethers it to the Gsy2p glycogen synthase
CAGL0I09108g		2,17	2,01E-05	-	Unknown
CAGL0C05071g		2,15	3,52E-05	*THI20*	Trifunctional enzyme of thiamine biosynthesis, degradation and salvage; has hydroxymethylpyrimidine (HMP) kinase, HMP-phosphate (HMP-P) kinase and thiaminase activities; member of a gene family with THI21 and THI22; HMP and HMP-P kinase activity redundant with Thi21p
CAGL0F04807g		2,15	1,17E-04	*OM45*	Protein of unknown function, major constituent of the mitochondrial outer membrane; located on the outer (cytosolic) face of the outer membrane
CAGL0I01100g	*CgGCY1*	2,15	1,44E-04	*GCY1*	Putative NADP(+) coupled glycerol dehydrogenase, proposed to be involved in an alternative pathway for glycerol catabolism; also has mRNA binding activity; member of the aldo-keto reductase (AKR) family
CAGL0J11242g		2,15	1,89E-04	*RHO5*	Non-essential small GTPase of the Rho/Rac subfamily of Ras-like proteins, likely involved in protein kinase C (Pkc1p)-dependent signal transduction pathway that controls cell integrity
CAGL0L11154g		2,14	3,34E-05	*NTE1*	Serine esterase, homolog of human neuropathy target esterase (NTE); Nte1p-mediated phosphatidylcholine turnover influences transcription factor Opi1p localization, affecting transcriptional regulation of phospholipid biosynthesis genes
CAGL0L01837g	*CgVPS24*	2,14	2,03E-05	*VPS24*	One of four subunits of the endosomal sorting complex required for transport III (ESCRT-III); forms an ESCRT-III subcomplex with Did4p; involved in the sorting of transmembrane proteins into the multivesicular body (MVB) pathway
CAGL0K12144g	*CgFES1*	2,13	4,76E-05	*FES1*	Hsp70 (Ssa1p) nucleotide exchange factor, cytosolic homolog of Sil1p, which is the nucleotide exchange factor for BiP (Kar2p) in the endoplasmic reticulum
CAGL0C02541g		2,13	7,55E-05	*BDF1*	Protein involved in transcription initiation at TATA-containing promoters; associates with the basal transcription factor TFIID; contains two bromodomains; corresponds to the C-terminal region of mammalian TAF1; redundant with Bdf2p
CAGL0B00902g	*CgHIS4*	2,13	1,03E-04	*HIS4*	Multifunctional enzyme containing phosphoribosyl-ATP pyrophosphatase, phosphoribosyl-AMP cyclohydrolase, and histidinol dehydrogenase activities; catalyzes the second, third, ninth and tenth steps in histidine biosynthesis
CAGL0H00110g		2,13	1,57E-04	-	Adhesin-like protein with internal repeats; predicted GPI-anchor
CAGL0M03179g		2,13	4,90E-05	*YKR018C*	Putative protein of unknown function; green fluorescent protein (GFP)-fusion protein localizes to the cytoplasm and nucleus
CAGL0G07183g		2,13	2,52E-04	-	Unknown
CAGL0K09702g		2,12	3,00E-04	*YNL134C*	Putative protein of unknown function with similarity to dehydrogenases from other model organisms; green fluorescent protein (GFP)-fusion protein localizes to both the cytoplasm and nucleus and is induced by the DNA-damaging agent MMS
CAGL0K12100g		2,12	2,39E-05	*HEM13*	Coproporphyrinogen III oxidase, an oxygen requiring enzyme that catalyzes the sixth step in the heme biosynthetic pathway; transcription is repressed by oxygen and heme (via Rox1p and Hap1p)

CAGL0L00495g	*CgHSC82*	2,11	1,11E-04	*HSC82*	Cytoplasmic chaperone of the Hsp90 family, redundant in function and nearly identical with Hsp82p, and together they are essential; expressed constitutively at 10-fold higher basal levels than HSP82 and induced 2-3 fold by heat shock
CAGL0D04422g		2,11	9,35E-05	*YPL279C*	Putative protein of unknown function
CAGL0G10219g	*CgAWP12*	2,11	2,63E-04	-	Adhesin-like protein with 5 tandem repeats
CAGL0I09086g		2,11	2,89E-04	*ESBP6*	Protein with similarity to monocarboxylate permeases, appears not to be involved in transport of monocarboxylates such as lactate, pyruvate or acetate across the plasma membrane
CAGL0F07359g		2,10	3,28E-05	*YGL117W*	Putative protein of unknown function
CAGL0E06292g		2,10	1,63E-04	*YHR022C*	Putative protein of unknown function; YHR022C is not an essential gene
CAGL0L05236g	*CgMDH1*	2,08	2,22E-03	*MDH1*	Mitochondrial malate dehydrogenase, catalyzes interconversion of malate and oxaloacetate; involved in the tricarboxylic acid (TCA) cycle; phosphorylated
CAGL0G02849g		2,08	3,58E-04	*UIP4*	Protein that interacts with Ulp1p, a Ubl (ubiquitin-like protein)-specific protease for Smt3p protein conjugates; detected in a phosphorylated state in the mitochondrial outer membrane; also detected in ER and nuclear envelope
CAGL0E06424g		2,06	2,00E-03	*MCR1*	Mitochondrial NADH-cytochrome b5 reductase, involved in ergosterol biosynthesis
CAGL0H08261g		2,05	7,44E-05	*YOR019W*	Protein of unknown function that may interact with ribosomes, based on co-purification experiments
CAGL0M12320g		2,05	1,34E-04	*FLC2*	Putative FAD transporter; required for uptake of FAD into endoplasmic reticulum; involved in cell wall maintenance
CAGL0I06204g	*CgPIR1*	2,05	4,70E-03	*YJL160C*	Putative protein of unknown function; member of the PIR (proteins with internal repeats) family of cell wall proteins; non-essential gene that is required for sporulation; mRNA is weakly cell cycle regulated, peaking in mitosis
CAGL0K10428g		2,05	2,37E-04	*IGD1*	Cytoplasmic protein that inhibits Gdb1p glycogen debranching activity; required for normal intracellular accumulation of glycogen; phosphorylated in vivo; expression increases during wine fermentation
CAGL0L12012g		2,04	1,32E-04	*TMT1*	Trans-aconitate methyltransferase, cytosolic enzyme that catalyzes the methyl esterification of 3-isopropylmalate, an intermediate of the leucine biosynthetic pathway, and trans-aconitate, which inhibits the citric acid cycle
CAGL0L10758g	*CgPFK2*	2,03	1,65E-04	*PFK2*	Beta subunit of heterooctameric phosphofructokinase involved in glycolysis, indispensable for anaerobic growth, activated by fructose-2,6-bisphosphate and AMP, mutation inhibits glucose induction of cell cycle-related genes
CAGL0K07546g	*CgPMU2*	2,03	1,19E-04	-	Putative phosphate starvation inducible acid phosphatase
CAGL0E06072g		2,02	2,55E-04	*YMR181C*	Protein of unknown function; mRNA transcribed as part of a bicistronic transcript with a predicted transcriptional repressor RGM1/YMR182C; mRNA is destroyed by nonsense-mediated decay (NMD); YMR181C is not an essential gene
CAGL0J11770g	*CgPLB1*	2,02	4,32E-05	*PLB1*	Phospholipase B (lysophospholipase) involved in lipid metabolism, required for deacylation of phosphatidylcholine and phosphatidylethanolamine but not phosphatidylinositol
CAGL0G02937g		2,01	1,05E-03	-	Ortholog(s) have pyruvate decarboxylase activity and role in L-phenylalanine catabolic process
CAGL0D01298g	*CgTKL1*	2,01	4,37E-03	*TKL1*	Transketolase, similar to Tkl2p; catalyzes conversion of xylulose-5-phosphate and ribose-5-phosphate to sedoheptulose-7-phosphate and glyceraldehyde-3-phosphate in the pentose phosphate pathway; needed for synthesis of aromatic amino acids
CAGL0I06743g		2,00	3,37E-03	*FTR1*	High affinity iron permease involved in the transport of iron across the plasma membrane; forms complex with Fet3p; expression is regulated by iron
CAGL0M07315g		2,00	2,34E-04	*SUR1*	Probable catalytic subunit of a mannosylinositol phosphorylceramide (MIPC) synthase, forms a complex with probable regulatory subunit Csg2p; function in sphingolipid biosynthesis is overlapping with that of Csh1p
CAGL0J05060g		1,98	8,24E-05	*ZAP1*	Zinc-regulated transcription factor; binds to zinc-responsive promoters to induce transcription of certain genes in presence of zinc, represses other genes in low zinc; regulates its own transcription; contains seven zinc-finger domains
CAGL0G09042g		1,98	7,17E-05	*AFT2*	Iron-regulated transcriptional activator; activates genes involved in intracellular iron use and required for iron homeostasis and resistance to oxidative stress; similar to Aft1p
CAGL0E05588g		1,97	7,53E-05	*REV1*	Deoxycytidyl transferase; involved in repair of abasic sites and adducted guanines in damaged DNA by translesion synthesis (TLS); forms a complex with the subunits of DNA polymerase zeta, Rev3p and Rev7p;
CAGL0K04169g		1,97	7,12E-05	*KSS1*	Mitogen-activated protein kinase (MAPK) involved in signal transduction pathways that control filamentous growth and pheromone response; the KSS1 gene is nonfunctional in S288C strains and functional in W303 strains
CAGL0K01155g		1,97	1,55E-03	*YGR079W*	Putative protein of unknown function; YGR079W is not an essential gene
CAGL0E05456g		1,97	2,10E-04	*YOR338W*	Putative protein of unknown function; YOR338W transcription is regulated by Azf1p and its transcript is a specific target of the G protein effector Scp160p; identified as being required for sporulation in a high-throughput mutant screen
CAGL0M06347g		1,97	9,00E-05	*YPC1*	Alkaline ceramidase that also has reverse (CoA-independent) ceramide synthase activity, catalyzes both breakdown and synthesis of phytoceramide; overexpression confers fumonisin B1 resistance
CAGL0L06974g		1,96	8,98E-04	*YDL086W*	Putative protein of unknown function; the authentic, non-tagged protein is detected in highly purified mitochondria in high-throughput studies; YDL086W is not an essential gene
CAGL0J09262g		1,95	3,12E-04	*STF1*	Protein involved in regulation of the mitochondrial F1F0-ATP synthase; Stf1p and Stf2p act as stabilizing factors that enhance inhibitory action of the Inh1p protein
CAGL0F02167g		1,94	2,18E-04	*MSH4*	Protein involved in meiotic recombination, required for normal levels of crossing over, colocalizes with Zip2p to discrete foci on meiotic chromosomes, has homology to bacterial MutS protein
CAGL0I01276g		1,93	3,33E-03	*YHR112C*	Protein of unknown function; localizes to the cytoplasm and nucleus; overexpression affects protein trafficking through the endocytic pathway
CAGL0I05390g		1,93	2,26E-04	*SKS1*	Putative serine/threonine protein kinase; involved in the adaptation to low concentrations of glucose independent of the SNF3 regulated pathway
CAGL0I07843g	*CgADH1*	1,93	2,18E-03	*ADH1*	Alcohol dehydrogenase, fermentative isozyme active as homo- or heterotetramers; required for the reduction of acetaldehyde to ethanol, the last step in the glycolytic pathway
CAGL0B02475g	*CgPHO84*	1,93	7,93E-04	*PHO84*	High-affinity inorganic phosphate (Pi) transporter and low-affinity manganese transporter; regulated by Pho4p and Spt7p; mutation confers resistance to arsenate; exit from the ER during maturation requires Pho86p; cells overexpressing Pho84p accumulate heavy metals but do not develop symptoms of metal toxicity
CAGL0K07183g		1,92	5,69E-03	-	Unknown

CAGL0K11858g		1,92	3,35E-04	*PST2*	Protein with similarity to members of a family of flavodoxin-like proteins; induced by oxidative stress in a Yap1p dependent manner; the authentic, non-tagged protein is detected in highly purified mitochondria in high-throughput studies
CAGL0I00550g		1,91	1,16E-03	*YLR297W*	Putative protein of unknown function; green fluorescent protein (GFP)-fusion protein localizes to the vacuole; YLR297W is not an essential gene; induced by treatment with 8-methoxypsoralen and UVA irradiation
CAGL0H08305g		1,91	3,48E-04	*DET1*	Acid phosphatase involved in the non-vesicular transport of sterols in both directions between the endoplasmic reticulum and plasma membrane; deletion confers sensitivity to nickel
CAGL0B03069g	*CgTAL1*	1,90	8,89E-03	*TAL1*	Transaldolase, enzyme in the non-oxidative pentose phosphate pathway; converts sedoheptulose 7-phosphate and glyceraldehyde 3-phosphate to erythrose 4-phosphate and fructose 6-phosphate
CAGL0F03399g	*CgSCS7*	1,88	1,44E-04	*SCS7*	Sphingolipid alpha-hydroxylase, functions in the alpha-hydroxylation of sphingolipid-associated very long chain fatty acids, has both cytochrome b5-like and hydroxylase/desaturase domains, not essential for growth
CAGL0H08195g		1,88	3,14E-04	*STI1*	Hsp90 cochaperone, interacts with the Ssa group of the cytosolic Hsp70 chaperones and activates Ssa1p ATPase activity; interacts with Hsp90 chaperones and inhibits their ATPase activity; homolog of mammalian Hop
CAGL0L03267g	*CgGAP1*	1,87	6,74E-03	*GAP1*	General amino acid permease; Gap1p senses the presence of amino acid substrates to regulate localization to the plasma membrane when needed
CAGL0L07480g		1,87	1,23E-04	*NRG2*	Transcriptional repressor that mediates glucose repression and negatively regulates filamentous growth; has similarity to Nrg1p
CAGL0I07821g		1,87	1,14E-04	*DUF1*	Ubiquitin-binding protein of unknown function; contains one WD40 repeat in a beta-propeller fold; green fluorescent protein (GFP)-fusion protein localizes to the cytoplasm; homolog of human WDR48/UAF1, which is involved in regulating the Fanconi anemia pathway; deletion mutant is sensitive to various chemicals including phenanthroline, sanguinarine, and nordihydroguaiaretic acid
CAGL0M03135g		1,87	1,75E-04	*ALY2*	Alpha arrestin that controls nutrient-mediated intracellular sorting of permease Gap1p; interacts with AP-1 subunit Apl4p; phosphorylated by Npr1p and also by cyclin-CDK complex Pcl7p-Pho85p; promotes endocytosis of plasma membrane proteins
CAGL0B00990g		1,86	2,15E-04	*FRM2*	Type II nitroreductase, using NADH as reductant; mutants are defective in fatty acid mediated repression of genes involved in fatty acid biosynthesis indicative of a role in lipid signaling; involved in the oxidative stress response; transcription induction by cadmium and selenite indicates a possible role in the metal stress response; expression induced in cells treated with the mycotoxin patulin
CAGL0C00319g		1,85	2,95E-04	*CPS1*	Vacuolar carboxypeptidase yscS; expression is induced under low-nitrogen conditions
CAGL0H02959g	*CgTOS8*	1,85	1,20E-03	*TOS8*	Homeodomain-containing protein and putative transcription factor found associated with chromatin; target of SBF transcription factor; induced during meiosis and under cell-damaging conditions; similar to Cup9p transcription factor
CAGL0L10582g		1,85	7,84E-04	*YMR196W*	Putative protein of unknown function; green fluorescent protein (GFP)-fusion protein localizes to the cytoplasm; YMR196W is not an essential gene
CAGL0F00605g	*CgGLK1*	1,85	6,46E-03	*EMI2*	Aldohexose specific glucokinase
CAGL0E06116g		1,84	3,37E-04	*RGM1*	Putative zinc finger DNA binding transcription factor; contains two N-terminal C2H2 zinc fingers and C-terminal proline rich domain; overproduction impairs cell growth and induces expression of genes involved in monosaccharide catabolism and aldehyde metabolism; deletion decreases expression of Y-prime telomeric elements; regulates expression of subtelomeric COS genes in conjunction with RNA binding protein Pub1p; localized to X elements within subtelomeric regions by ChIP
CAGL0K07678g		1,84	2,77E-04	-	Unknown
CAGL0K05357g	*CgGLN1*	1,84	1,63E-03	*GLN1*	Glutamine synthetase (GS), synthesizes glutamine from glutamate and ammonia; with Glt1p, forms the secondary pathway for glutamate biosynthesis from ammonia; expression regulated by nitrogen source and by amino acid limitation
CAGL0H04037g		1,84	1,60E-03	*GAC1*	Regulatory subunit for Glc7p type-1 protein phosphatase (PP1), tethers Glc7p to Gsy2p glycogen synthase, binds Hsf1p heat shock transcription factor, required for induction of some HSF-regulated genes under heat shock
CAGL0B04675g		1,83	9,99E-05	*DOM34*	Protein that, with binding partner Hbs1p, facilitates ribosomal subunit dissociation when translation is stalled; required for RNA cleavage in no-go decay, but reports conflict on endonuclease activity; Pelota ortholog
CAGL0I08613g	*CgDUR3*	1,83	8,74E-05	-	Putative plasma membrane polyamine transporter
CAGL0F06369g		1,83	2,60E-04	*LHS1*	Molecular chaperone of the endoplasmic reticulum lumen, involved in polypeptide translocation and folding; nucleotide exchange factor for the ER lumenal Hsp70 chaperone Kar2p; regulated by the unfolded protein response pathway
CAGL0J04268g		1,83	8,46E-05	*ACH1*	Protein with CoA transferase activity, particularly for CoASH transfer from succinyl-CoA to acetate; has minor acetyl-CoA-hydrolase activity; phosphorylated; required for acetate utilization and for diploid pseudohyphal growth
CAGL0F09207g	*CgBAT1*	1,82	9,68E-05	*BAT1*	Mitochondrial branched-chain amino acid (BCAA) aminotransferase, preferentially involved in BCAA biosynthesis; homolog of murine ECA39; highly expressed during logarithmic phase and repressed during stationary phase
CAGL0G07271g		1,82	1,57E-03	*TSA1*	Thioredoxin peroxidase, acts as both a ribosome-associated and free cytoplasmic antioxidant; self-associates to form a high-molecular weight chaperone complex under oxidative stress; deletion results in mutator phenotype
CAGL0K01727g	*CgRPN4*	1,82	1,26E-04	*RPN4*	Transcription factor that stimulates expression of proteasome genes; Rpn4p levels are in turn regulated by the 26S proteasome in a negative feedback control mechanism; RPN4 is transcriptionally regulated by various stress responses
CAGL0B03685g		1,82	1,59E-04	*YCP4*	Protein of unknown function, has sequence and structural similarity to flavodoxins; predicted to be palmitoylated; the authentic, non-tagged protein is detected in highly purified mitochondria in high-throughput studies
CAGL0L10186g		1,80	7,40E-05	*YOR052C*	Nuclear protein of unknown function; expression induced by nitrogen limitation in a GLN3, GAT1-independent manner and by weak acid; transcriptionally regulated by Rpn4p along with proteasome subunit genes; putative ortholog of human AIRAP, which stimulates proteasome activity in response to arsenic
CAGL0M05995g		1,80	1,59E-04	*PET10*	Protein of unknown function that co-purifies with lipid particles; expression pattern suggests a role in respiratory growth; computational analysis of large-scale protein-protein interaction data suggests a role in ATP/ADP exchange
CAGL0A01782g	*CgHXT4*	1,80	9,72E-04	*HXT4*	High-affinity glucose transporter of the major facilitator superfamily, expression is induced by low levels of glucose and repressed by high levels of glucose
CAGL0G05830g		1,79	1,27E-04	*CRP1*	Protein that binds to cruciform DNA structures
CAGL0K07205g		1,79	3,61E-04	*YGL117W*	Putative protein of unknown function
CAGL0L07920g		1,78	4,89E-04	*PET18*	Protein of unknown function, has weak similarity to proteins involved in thiamin metabolism; expression is induced in the absence of thiamin

CAGL0E03674g	*CgTPO1_1*	1,78	1,40E-04	*TPO1*	Polyamine transporter that recognizes spermine, putrescine, and spermidine; catalyzes uptake of polyamines at alkaline pH and excretion at acidic pH; phosphorylation enhances activity and sorting to the plasma membrane
CAGL0H02387g		1,78	1,38E-04	*TPS3*	Regulatory subunit of trehalose-6-phosphate synthase/phosphatase complex, which synthesizes the storage carbohydrate trehalose; expression is induced by stress conditions and repressed by the Ras-cAMP pathway
CAGL0G02101g	*CgECM4*	1,77	1,19E-04	*ECM4*	Omega class glutathione transferase; not essential; similar to Ygr154cp; green fluorescent protein (GFP)-fusion protein localizes to the cytoplasm
CAGL0K02101g		1,76	1,17E-04	*PMD1*	Protein with an N-terminal kelch-like domain, putative negative regulator of early meiotic gene expression; required, with Mds3p, for growth under alkaline conditions
CAGL0F03267g		1,76	1,01E-04	*YHR080C*	Protein of unknown function that may interact with ribosomes, based on co-purification experiments; the authentic, non-tagged protein is detected in highly purified mitochondria in high-throughput studies
CAGL0F09273g		1,75	1,92E-04	-	Putative adhesin-like protein
CAGL0B00264g	*HMLALPHA2*	1,75	7,35E-04	*HMLALPHA2*	Silenced copy of ALPHA2 at HML; homeobox-domain protein that associates with Mcm1p in haploid cells to repress a-specific gene expression and interacts with a1p in diploid cells to repress haploid-specific gene expression
CAGL0K03707g		1,75	7,43E-04	*YMR124W*	Protein of unknown function; GFP-fusion protein localizes to the cell periphery, cytoplasm, bud, and bud neck; interacts with Crm1p in two-hybrid assay; YMR124W is not an essential gene; predicted to have a role in organelle organization
CAGL0K01683g	*CgGPD1*	1,74	4,43E-04	*GPD1*	NAD-dependent glycerol-3-phosphate dehydrogenase, key enzyme of glycerol synthesis, essential for growth under osmotic stress; expression regulated by high-osmolarity glycerol response pathway; homolog of Gpd2p
CAGL0B03817g		1,74	1,53E-03	*MHO1*	Protein of unknown function; inhibits haploid invasive growth when overexpressed; synthetically lethal with phospholipase C (PLC1); expression induced by mild heat-stress on a non-fermentable carbon source, upon entry into stationary phase and upon nitrogen deprivation; repressed by inosine and choline in an Opi1p-dependent manner; highly conserved from bacteria to human; Memo, the human homolog, is an ErbB2 interacting protein with an essential function in cell motility
CAGL0G09977g	*CgGDB1*	1,74	4,65E-04	*GDB1*	Glycogen debranching enzyme containing glucanotranferase and alpha-1,6-amyloglucosidase activities, required for glycogen degradation; phosphorylated in mitochondria; activity is inhibited by Igd1p
CAGL0K05665g		1,73	1,02E-04	-	Has domain(s) with predicted catalytic activity, pyridoxal phosphate binding activity
CAGL0B04917g		1,73	5,50E-04	*IDP2*	Cytosolic NADP-specific isocitrate dehydrogenase, catalyzes oxidation of isocitrate to alpha-ketoglutarate; levels are elevated during growth on non-fermentable carbon sources and reduced during growth on glucose
CAGL0M03245g		1,73	3,05E-04	*YEL020C*	Protein of unknown function with low sequence identity to Pdc1p; mRNA identified as translated by ribosome profiling data
CAGL0M04807g	*CgSNF2*	1,73	1,79E-04	*SNF2*	Catalytic subunit of the SWI/SNF chromatin remodeling complex involved in transcriptional regulation; contains DNA-stimulated ATPase activity; functions interdependently in transcriptional activation with Snf5p and Snf6p
CAGL0M06765g		1,73	3,14E-04	*SIW14*	Tyrosine phosphatase that plays a role in actin filament organization and endocytosis; localized to the cytoplasm
CAGL0G08888g		1,73	9,56E-05	*CSM2*	Component of the Shu complex, which promotes error-free DNA repair; Shu complex mediates inhibition of Srs2p function; structural analysis reveals a similar DNA-binding region in both Psy3p and Csm2p and that both regions work together to form a single DNA binding site; required for accurate chromosome segregation during meiosis
CAGL0G06842g		1,72	1,34E-04	*BBC1*	Protein possibly involved in assembly of actin patches; interacts with an actin assembly factor Las17p and with the SH3 domains of Type I myosins Myo3p and Myo5p; localized predominantly to cortical actin patches
CAGL0J08074g		1,72	1,97E-04	*PDR17*	Phosphatidylinositol transfer protein (PITP), downregulates Plb1p-mediated turnover of phosphatidylcholine, found in the cytosol and microsomes, homologous to Pdr16p, deletion affects phospholipid composition
CAGL0E05610g		1,72	4,61E-03	*PYK2*	Pyruvate kinase that appears to be modulated by phosphorylation; PYK2 transcription is repressed by glucose, and Pyk2p may be active under low glycolytic flux
CAGL0J03960g		1,71	2,23E-04	*WTM2*	Transcriptional modulator involved in regulation of meiosis, silencing, and expression of RNR genes; involved in response to replication stress; contains WD repeats
CAGL0J08316g		1,71	5,43E-03	*MET2*	L-homoserine-O-acetyltransferase, catalyzes the conversion of homoserine to O-acetyl homoserine which is the first step of the methionine biosynthetic pathway
CAGL0J07502g		1,71	3,75E-04	*YNL234W*	Protein of unknown function with similarity to globins; has a functional heme-binding domain; mutant has aneuploidy tolerance; transcription induced by stress conditions; may be involved in glucose signaling or metabolism; regulated by Rgt1
CAGL0C01551g		1,70	3,49E-04	*CUP9*	Homeodomain-containing transcriptional repressor of PTR2, which encodes a major peptide transporter; imported peptides activate ubiquitin-dependent proteolysis, resulting in degradation of Cup9p and de-repression of PTR2 transcription
CAGL0F07821g		1,70	2,22E-04	*HUL5*	Multiubiquitin chain assembly factor (E4); proteasome processivity factor that elongates polyUb chains on substrates, opposing Ubp6p, a branched polyubiquitin protease; required for retrograde transport of misfolded proteins during ERAD
CAGL0H05379g		1,69	4,13E-04	*GCR1*	Transcriptional activator of genes involved in glycolysis; DNA-binding protein that interacts and functions with the transcriptional activator Gcr2p
CAGL0G01540g	*CgNCE103*	1,69	2,42E-03	*NCE103*	Carbonic anhydrase; metalloenzyme that catalyzes CO2 hydration to bicarbonate, which is an important metabolic substrate, and protons; not expressed under conditions of high CO2, such as inside a growing colony, but transcription is induced in response to low CO2 levels, such as on the colony surface in ambient air; poorly transcribed under aerobic conditions and at an undetectable level under anaerobic conditions; mutation affects non-classical protein export pathway
CAGL0H10164g		1,69	1,45E-03	*MUM2*	Cytoplasmic protein essential for meiotic DNA replication and sporulation; interacts with Orc2p, which is a component of the origin recognition complex
CAGL0M02915g		1,69	5,89E-04	*ATG36*	Pex3p interacting protein, required for pexophagy; interacts with Atg8p and Atg11p; mRNA is weakly cell cycle regulated, peaking in G2 phase; YJL185C is a non-essential gene
CAGL0G09449g		1,69	1,46E-04	*CRH1*	Chitin transglycosylase that functions in the transfer of chitin to beta(1-6) and beta(1-3) glucans in the cell wall; similar and functionally redundant to Utr2; localizes to sites of polarized growth; expression induced by cell wall stress
CAGL0L05016g		1,68	4,26E-04	*STB6*	Protein that binds Sin3p in a two-hybrid assay
CAGL0L06446g		1,68	2,12E-03	*YDR132C*	Putative protein of unknown function
CAGL0B01265g	*MATALPHA2*	1,68	1,37E-03	*MATALPHA2*	Homeobox-domain protein that, with Mcm1p, represses a-specific genes in haploids; acts with A1p to repress transcription of haploid-specific genes in diploids; one of two genes encoded by the MATalpha mating type cassette
CAGL0E04548g		1,67	4,65E-04	*YOR020W-A*	Putative protein of unknown function, conserved in A. gossypii; the authentic, non-tagged protein is detected in highly purified mitochondria in high-throughput studies

CAGL0H10626g	*CgAWP13*	1,67	4,53E-04	*NA*	NA
CAGL0L06006g		1,67	2,54E-04	*ATG1*	Protein ser/thr kinase required for vesicle formation in autophagy and the cytoplasm-to-vacuole targeting (Cvt) pathway; structurally required for phagophore assembly site formation; during autophagy forms a complex with Atg13p and Atg17p
CAGL0J11176g		1,67	1,60E-04	*TDA7*	Cell cycle-regulated gene of unknown function, promoter bound by Fkh2p; null mutant is sensitive to expression of the top1-T722A allele
CAGL0M01716g		1,66	6,80E-03	*TEC1*	Transcription factor targeting filamentation genes and Ty1 expression; Ste12p activation of most filamentation gene promoters depends on Tec1p and Tec1p transcriptional activity is dependent on its association with Ste12p; binds to TCS elements upstream of filamentation genes, which are regulated by Tec1p/Ste12p/Dig1p complex; competes with Dig2p for binding to Ste12p/Dig1p; positive regulator of chronological life span; TEA/ATTS DNA-binding domain family member
CAGL0K08338g		1,65	1,16E-03	-	Unknown
CAGL0L06776g		1,65	6,12E-04	*GAT2*	Protein containing GATA family zinc finger motifs; similar to Gln3p and Dal80p; expression repressed by leucine
CAGL0D04752g		1,65	1,90E-04	*YPR127W*	Protein of unknown function, differentially expressed during alcoholic fermentation; expression activated by transcription factor YRM1/YOR172W; green fluorescent protein (GFP)-fusion protein localizes to both the cytoplasm and the nucleus
CAGL0C02365g		1,64	1,65E-03	-	Unknown
CAGL0I05698g		1,64	1,45E-04	*PFK2*	Beta subunit of heterooctameric phosphofructokinase involved in glycolysis, indispensable for anaerobic growth, activated by fructose-2,6-bisphosphate and AMP, mutation inhibits glucose induction of cell cycle-related genes
CAGL0C03597g	*CgABP1*	1,64	4,05E-04	*ABP1*	Actin-binding protein of the cortical actin cytoskeleton, important for activation of the Arp2/3 complex that plays a key role actin in cytoskeleton organization; phosphorylation within its PRR (Proline-Rich Region), mediated by Cdc28p and Pho85p, protects Abp1p from proteolysis mediated by its own PEST sequences
CAGL0F02101g		1,64	2,81E-04	*BLM10*	Proteasome activator; binds the core proteasome and stimulates proteasome-mediated protein degradation by inducing gate opening; required for resistance to bleomycin, may be involved in protecting against oxidative damage; similar to mammalian PA200
CAGL0L11990g		1,63	1,07E-03	*GRX4*	Hydroperoxide and superoxide-radical responsive glutathione-dependent oxidoreductase; monothiol glutaredoxin subfamily member along with Grx3p and Grx5p; protects cells from oxidative damage; mutant has increased aneuploidy tolerance; transcription is regulated by Yap5
CAGL0F03003g		1,62	1,02E-03	*HKR1*	Mucin family member that functions as an osmosensor in the Sho1p-mediated HOG pathway with Msb2p; proposed to be a negative regulator of filamentous growth; mutant displays defects in beta-1,3 glucan synthesis and bud site selection
CAGL0B00594g		1,62	2,85E-04	*YCL048W-A*	Putative protein of unknown function
CAGL0L02453g		1,61	6,66E-04	*MIT1*	Transcriptional regulator of pseudohyphal growth; protein with sequence similarity to S. pombe gti1+ (gluconate transport inducer 1) and C. albicans Wor1
CAGL0M10439g		1,61	2,27E-03	*NTH1*	Neutral trehalase, degrades trehalose; required for thermotolerance and may mediate resistance to other cellular stresses; may be phosphorylated by Cdc28p
CAGL0E03025g		1,60	2,02E-04	*ECL1*	Protein of unknown function, affects chronological lifespan; induced by iron homeostasis transcription factor Aft2p; multicopy suppressor of temperature sensitive hsf1 mutant; induced by treatment with 8-methoxypsoralen and UVA irradiation
CAGL0F07601g	*CgCWP1_1*	1,59	1,65E-03	*CWP2*	Covalently linked cell wall mannoprotein, major constituent of the cell wall; plays a role in stabilizing the cell wall; involved in low pH resistance; precursor is GPI-anchored
CAGL0G08712g		1,59	7,42E-04	*KGD1*	Component of the mitochondrial alpha-ketoglutarate dehydrogenase complex, which catalyzes a key step in the tricarboxylic acid (TCA) cycle, the oxidative decarboxylation of alpha-ketoglutarate to form succinyl-CoA
CAGL0H10120g		1,59	2,03E-03	*YBR056W*	Putative cytoplasmic protein of unknown function
CAGL0M01364g	*CgCBF3D*	1,58	6,01E-03	*SKP1*	Evolutionarily conserved kinetochore protein that is part of multiple protein complexes, including the SCF ubiquitin ligase complex, the CBF3 complex that binds centromeric DNA, and the RAVE complex that regulates assembly of the V-ATPase
CAGL0L03157g		1,58	9,40E-04	*DAL80*	Negative regulator of genes in multiple nitrogen degradation pathways; expression is regulated by nitrogen levels and by Gln3p; member of the GATA-binding family, forms homodimers and heterodimers with Deh1p
CAGL0F00187g		1,57	9,49E-04	*FET4*	Low-affinity Fe(II) transporter of the plasma membrane
CAGL0C05027g		1,57	2,72E-04	*YAT1*	Outer mitochondrial carnitine acetyltransferase, minor ethanol-inducible enzyme involved in transport of activated acyl groups from the cytoplasm into the mitochondrial matrix; phosphorylated
CAGL0E05170g	*CgGRE2*	1,57	1,95E-03	-	Putative methylglyoxal reductase (NADPH-dependent)
CAGL0F07579g	*CgCWP1_2*	1,57	1,66E-03	*CWP1*	Cell wall mannoprotein that localizes specifically to birth scars of daughter cells, linked to a beta-1,3- and beta-1,6-glucan heteropolymer through a phosphodiester bond; required for propionic acid resistance
CAGL0E01529g		1,56	4,61E-04	*PFK27*	6-phosphofructo-2-kinase, catalyzes synthesis of fructose-2,6-bisphosphate; inhibited by phosphoenolpyruvate and sn-glycerol 3-phosphate, expression induced by glucose and sucrose, transcriptional regulation involves protein kinase A
CAGL0M04191g	*CgYPS1*	1,56	2,19E-03	*YPS1*	Aspartic protease, member of the yapsin family of proteases involved in cell wall growth and maintenance; attached to the plasma membrane via a glycosylphosphatidylinositol (GPI) anchor
CAGL0I09009g		1,55	1,08E-03	*HIS2*	Histidinolphosphatase, catalyzes the eighth step in histidine biosynthesis; mutations cause histidine auxotrophy and sensitivity to Cu, Co, and Ni salts; transcription is regulated by general amino acid control
CAGL0J01870g		1,55	3,05E-04	*PMR1*	High affinity Ca2+/Mn2+ P-type ATPase required for Ca2+ and Mn2+ transport into Golgi; involved in Ca2+ dependent protein sorting and processing; mutations in human homolog ATP2C1 cause acantholytic skin condition Hailey-Hailey disease
CAGL0E05808g	*CgTHI6*	1,54	5,72E-04	*THI6*	Bifunctional enzyme with thiamine-phosphate pyrophosphorylase and 4-methyl-5-beta-hydroxyethylthiazole kinase activities, required for thiamine biosynthesis; GFP-fusion protein localizes to the cytoplasm in a punctate pattern
CAGL0F04125g		1,54	2,46E-03	*YBL029W*	Non-essential protein of unknown function
CAGL0I10747g		1,54	4,48E-04	*MEP1*	Ammonium permease; belongs to a ubiquitous family of cytoplasmic membrane proteins that transport only ammonium (NH4+); expression is under the nitrogen catabolite repression regulation
CAGL0H02541g		1,53	1,09E-03	*YMR252C*	Putative protein of unknown function; green fluorescent protein (GFP)-fusion protein localizes to mitochondria; YMR252C is not an essential gene
CAGL0L08074g		1,53	4,84E-04	*SYP1*	Protein of unknown function that is involved in endocytic site formation; may regulate assembly and disassembly of the septin ring; colocalizes and interacts with septin subunits; potential role in actin cytoskeletal organization

CAGL0B02860g		1,53	2,78E-03	*ATG33*	Mitochondrial mitophagy-specific protein; required primarily for mitophagy induced at the post-log phase; not required for other types of selective autophagy or macroautophagy; conserved within fungi, but not in higher eukaryotes
CAGL0M00132g	*CgEPA12*	1,52	1,58E-03	-	Putative adhesin-like cell wall protein
CAGL0C03113g		1,52	7,59E-04	*DCS1*	Non-essential hydrolase involved in mRNA decapping; activates Xrn1p; may function in a feedback mechanism to regulate deadenylation, contains pyrophosphatase activity and a HIT (histidine triad) motif; interacts with neutral trehalase Nth1p; required for growth on glycerol medium
CAGL0L10912g		1,51	8,34E-04	*TPO4*	Polyamine transport protein, recognizes spermine, putrescine, and spermidine; localizes to the plasma membrane; member of the major facilitator superfamily
CAGL0E03740g		1,51	5,76E-04	*YHL026C*	Putative protein of unknown function; transcriptionally regulated by Upc2p via an upstream sterol response element; YHL026C is not an essential gene; in 2005 the start site was moved 141 nt upstream (see Locus History)
CAGL0L03916g		1,50	1,12E-03	*AZF1*	Zinc-finger transcription factor, involved in induction of CLN3 transcription in response to glucose; genetic and physical interactions indicate a possible role in mitochondrial transcription or genome maintenance
CAGL0A03212g		1,50	1,07E-03	*ATO3*	Plasma membrane protein, regulation pattern suggests a possible role in export of ammonia from the cell; phosphorylated in mitochondria; member of the TC 9.B.33 YaaH family of putative transporters
CAGL0B01595g		1,49	5,42E-04	-	Unknown
CAGL0M10153g		1,49	1,53E-03	*STE20*	Cdc42p-activated signal transducing kinase of the PAK (p21-activated kinase) family; involved in pheromone response, pseudohyphal/invasive growth, vacuole inheritance, down-regulation of sterol uptake; GBB motif binds Ste4p
CAGL0F02519g		1,48	3,99E-04	*YJL206C*	Putative protein of unknown function; similar to transcriptional regulators from the Zn[2]-Cys[6] binuclear cluster protein family; mRNA is weakly cell cycle regulated, peaking in S phase; induced rapidly upon MMS treatment
CAGL0B04895g		1,48	1,81E-03	*RFX1*	Major transcriptional repressor of DNA-damage-regulated genes, recruits repressors Tup1p and Cyc8p to their promoters; involved in DNA damage and replication checkpoint pathway; similar to a family of mammalian DNA binding RFX1-4 proteins
CAGL0K12078g		1,48	4,33E-04	*NRG1*	Transcriptional repressor that recruits the Cyc8p-Tup1p complex to promoters; mediates glucose repression and negatively regulates a variety of processes including filamentous growth and alkaline pH response
CAGL0L09273g		1,48	7,20E-04	*ICL2*	2-methylisocitrate lyase of the mitochondrial matrix, functions in the methylcitrate cycle to catalyze the conversion of 2-methylisocitrate to succinate and pyruvate; ICL2 transcription is repressed by glucose and induced by ethanol
CAGL0A02145g		1,47	2,80E-03	*YSC84*	Actin-binding protein involved in bundling of actin filaments and endocytosis of actin cortical patches; activity stimulated by Las17p; contains SH3 domain similar to Rvs167p
CAGL0D06424g	*CgACO1*	1,47	5,10E-04	*ACO1*	Aconitase, required for the tricarboxylic acid (TCA) cycle and also independently required for mitochondrial genome maintenance; phosphorylated; component of the mitochondrial nucleoid; mutation leads to glutamate auxotrophy
CAGL0K10824g		1,47	9,43E-04	*YLR149C*	Putative protein of unknown function; overexpression causes a cell cycle delay or arrest; null mutation results in a decrease in plasma membrane electron transport; YLR149C is not an essential gene
CAGL0C04213g		1,47	6,20E-04	*RCR1*	Protein of the ER membrane involved in cell wall chitin deposition; may function in the endosomal-vacuolar trafficking pathway, helping determine whether plasma membrane proteins are degraded or routed to the plasma membrane
CAGL0C04587g		1,47	1,41E-03	*YJR098C*	Putative protein of unknown function; the authentic, non-tagged protein is detected in highly purified mitochondria in high-throughput studies
CAGL0D02948g	*CgKAR2*	1,46	1,18E-03	*KAR2*	ATPase involved in protein import into the ER, also acts as a chaperone to mediate protein folding in the ER and may play a role in ER export of soluble proteins; regulates the unfolded protein response via interaction with Ire1p
CAGL0E00319g		1,46	1,84E-03	-	Unknown
CAGL0H07997g	*CgKNH1*	1,46	9,47E-04	*KNH1*	Protein with similarity to Kre9p, which is involved in cell wall beta 1,6-glucan synthesis; overproduction suppresses growth defects of a kre9 null mutant; required for propionic acid resistance
CAGL0H09966g		1,46	9,24E-04	*FMP23*	Putative protein of unknown function; proposed to be involved in iron or copper homeostasis; the authentic, non-tagged protein is detected in highly purified mitochondria in high-throughput studies
CAGL0I04180g	*CgAMT1*	1,46	1,30E-03	*CUP2*	Copper-binding transcription factor; activates transcription of the metallothionein genes CUP1-1 and CUP1-2 in response to elevated copper concentrations
CAGL0E04004g		1,46	5,01E-03	*MUP3*	Low affinity methionine permease, similar to Mup1p
CAGL0H10054g		1,46	3,46E-03	*YBR053C*	Putative protein of unknown function; induced by cell wall perturbation
CAGL0M03157g		1,46	4,12E-03	*IRS4*	EH domain-containing protein involved in regulating phosphatidylinositol 4,5-bisphosphate levels and autophagy; Irs4p and Tax4p bind and activate the PtdIns phosphatase Inp51p; Irs4p and Tax4p are involved in localizing Atg17p to the PAS
CAGL0B02431g		1,45	1,71E-03	*NDI1*	NADH:ubiquinone oxidoreductase, transfers electrons from NADH to ubiquinone in the respiratory chain but does not pump protons, in contrast to the higher eukaryotic multisubunit respiratory complex I; phosphorylated; homolog of human AMID
CAGL0A03718g		1,45	8,46E-04	*CHC1*	Clathrin heavy chain, subunit of the major coat protein involved in intracellular protein transport and endocytosis; two heavy chains form the clathrin triskelion structural component; the light chain (CLC1) is thought to regulate function
CAGL0K08624g		1,45	1,19E-03	*HAP4*	Subunit of the heme-activated, glucose-repressed Hap2p/3p/4p/5p CCAAT-binding complex, a transcriptional activator and global regulator of respiratory gene expression; provides the principal activation function of the complex
CAGL0M09449g	*CgCRH1*	1,44	5,78E-04	*ECM21*	Protein involved in regulating the endocytosis of plasma membrane proteins; identified as a substrate for ubiquitination by Rsp5p and deubiquitination by Ubp2p; promoter contains several Gcn4p binding elements
CAGL0I02530g		1,43	9,86E-04	*FMO1*	Flavin-containing monooxygenase, localized to the cytoplasmic face of the ER membrane; catalyzes oxidation of biological thiols to maintain the ER redox buffer ratio for correct folding of disulfide-bonded proteins
CAGL0I05522g		1,43	4,19E-03	*UBP9*	Ubiquitin carboxyl-terminal hydrolase, ubiquitin-specific protease that cleaves ubiquitin-protein fusions
CAGL0G09295g		1,43	8,15E-04	*FYV8*	Protein of unknown function, required for survival upon exposure to K1 killer toxin
CAGL0M02211g	*CgPEP4*	1,43	9,74E-03	*PEP4*	Vacuolar aspartyl protease (proteinase A), required for the posttranslational precursor maturation of vacuolar proteinases; important for protein turnover after oxidative damage; synthesized as a zymogen, self-activates

	CAGL0L01177g	*CgFRSD1*	1,43	1,88E-03	*YEL047C*	Soluble fumarate reductase, required with isoenzyme Osm1p for anaerobic growth; may interact with ribosomes, based on co-purification experiments; authentic, non-tagged protein is detected in purified mitochondria in high-throughput studies
	CAGL0H08173g		1,43	7,93E-03	-	Unknown
	CAGL0J11968g	*CgEPA15*	1,42	1,15E-03	-	Putative adhesin-like cell wall protein
	CAGL0A03872g		1,42	3,66E-03	*ENT2*	Epsin-like protein required for endocytosis and actin patch assembly and functionally redundant with Ent1p; contains clathrin-binding motif at C-terminus
	CAGL0G09515g		1,42	4,42E-04	*SPR1*	Sporulation-specific exo-1,3-beta-glucanase; contributes to ascospore thermoresistance
	CAGL0C03267g		1,42	7,06E-04	*FPS1*	Plasma membrane channel, member of major intrinsic protein (MIP) family; involved in efflux of glycerol and in uptake of acetic acid and the trivalent metalloids arsenite and antimonite; phosphorylated by Hog1p MAPK under acetate stress
	CAGL0I08305g		1,42	8,14E-04	*YAT2*	Carnitine acetyltransferase; has similarity to Yat1p, which is a carnitine acetyltransferase associated with the mitochondrial outer membrane
	CAGL0M07612g		1,41	1,91E-03	*FMS1*	Polyamine oxidase, converts spermine to spermidine, which is required for the essential hypusination modification of translation factor eIF-5A; also involved in pantothenic acid biosynthesis
	CAGL0L03135g	*MFalpha*	1,41	4,99E-04	*SPO14*	Phospholipase D; catalyzes the hydrolysis of phosphatidylcholine, producing choline and phosphatidic acid; involved in Sec14p-independent secretion; required for meiosis and spore formation; differently regulated in secretion and meiosis; participates in transcription initiation and/or early elongation of specific genes; interacts with foot domain of RNA polymerase II; deletion results in abnormal CTD-Ser5 phosphorylation of RNA polymerase II at specific promoter regions
2,66	CAGL0J03058g		1,41	7,25E-04	*ICL1*	Isocitrate lyase, catalyzes the formation of succinate and glyoxylate from isocitrate, a key reaction of the glyoxylate cycle; expression of ICL1 is induced by growth on ethanol and repressed by growth on glucose
	CAGL0F01793g	*CgERG3*	1,41	1,37E-03	*ERG3*	C-5 sterol desaturase, glycoprotein that catalyzes the introduction of a C-5(6) double bond into episterol, a precursor in ergosterol biosynthesis; mutants are viable, but cannot grow on non-fermentable carbon sources; substrate of the HRD ubiquitin ligase
	CAGL0H05445g	*CgPGI1*	1,41	4,72E-03	*PGI1*	Glycolytic enzyme phosphoglucose isomerase, catalyzes the interconversion of glucose-6-phosphate and fructose-6-phosphate; required for cell cycle progression and completion of the gluconeogenic events of sporulation
	CAGL0M06963g		1,41	3,61E-04	*SOL2*	Protein with a possible role in tRNA export; shows similarity to 6-phosphogluconolactonase non-catalytic domains but does not exhibit this enzymatic activity; homologous to Sol1p, Sol3p, and Sol4p
	CAGL0M02101g		1,41	3,70E-04	*PET20*	Mitochondrial protein, required for respiratory growth under some conditions and for stability of the mitochondrial genome
	CAGL0H04213g		1,41	1,01E-03	*TDA9*	DNA-binding protein, putative transcription factor; green fluorescent protein (GFP)-fusion protein localizes to the nucleus; null mutant is sensitive to expression of the top1-T722A allele; not an essential gene
	CAGL0L00583g		1,40	3,81E-03	*USV1*	Putative transcription factor containing a C2H2 zinc finger; mutation affects transcriptional regulation of genes involved in growth on non-fermentable carbon sources, response to salt stress and cell wall biosynthesis
	CAGL0G06006g		1,40	2,73E-03	*YHR138C*	Putative protein of unknown function; has similarity to Pbi2p; double null mutant lacking Pbi2p and Yhr138p exhibits highly fragmented vacuoles
	CAGL0L08426g	*CgSUE1*	1,39	5,90E-04	*SUE1*	Mitochondrial protein required for degradation of unstable forms of cytochrome c
	CAGL0K01705g		1,39	4,38E-04	*GPM2*	Homolog of Gpm1p phosphoglycerate mutase, which converts 3-phosphoglycerate to 2-phosphoglycerate in glycolysis; may be non-functional derivative of a gene duplication event
	CAGL0F04829g		1,39	5,82E-04	*PIN3*	Protein that induces appearance of [PIN+] prion when overproduced
	CAGL0H02893g		1,39	5,14E-04	*YJL070C*	Protein of unknown function; some similarity to AMP deaminases but lacks key catalytic residues and does not rescue purine nucleotide metabolic defect of quadruple aah1 ade8 amd1 his1 mutant; may regulate purine nucleotide homeostasis as overexpression in an AMD1 strain grown in adenine results in greatly reduced GDP and GTP intracellular levels; the authentic, non-tagged protein is detected in highly purified mitochondria in high-throughput studies; not an essential gene
	CAGL0G05357g		1,39	1,06E-03	*YNL200C*	NADHX epimerase; catalyzes isomerization of (R)- and (S)-NADHX; homologous to AIBP in mammals and the N- terminal domain of YjeF in E.coli; enzyme is widespread in eukaryotes, prokaryotes and archaea; the authentic, non-tagged protein is detected in highly purified mitochondria in high-throughput studies
	CAGL0D04510g		1,39	6,63E-04	*YPR117W*	Putative protein of unknown function
	CAGL0I07227g		1,38	6,48E-04	*IDH2*	Subunit of mitochondrial NAD(+)-dependent isocitrate dehydrogenase, which catalyzes the oxidation of isocitrate to alpha-ketoglutarate in the TCA cycle; phosphorylated
	CAGL0G06798g		1,38	6,06E-04	*YJR005C-A*	Putative protein of unknown function, originally identified as a syntenic homolog of an <i>Ashbya gossypii</i> gene
	CAGL0B00704g	*CgPDI1*	1,38	7,58E-03	*PDI1*	Protein disulfide isomerase; multifunctional protein resident in the endoplasmic reticulum lumen, essential for the formation of disulfide bonds in secretory and cell-surface proteins, unscrambles non-native disulfide bonds; forms a complex with Mnl1p that has exomannosidase activity, processing unfolded protein-bound Man8GlcNAc2 oligosaccharides to Man7GlcNAc2 which promotes degradation in the unfolded protein response
	CAGL0G02673g		1,38	3,98E-04	*IDH1*	Subunit of mitochondrial NAD(+)-dependent isocitrate dehydrogenase, which catalyzes the oxidation of isocitrate to alpha-ketoglutarate in the TCA cycle
	CAGL0M04763g		1,37	2,03E-03	*YOR289W*	Putative protein of unknown function; transcription induced by the unfolded protein response; green fluorescent protein (GFP)-fusion protein localizes to both the cytoplasm and the nucleus
	CAGL0M10571g		1,37	4,91E-04	*ARE2*	Acyl-CoA:sterol acyltransferase, isozyme of Are1p; endoplasmic reticulum enzyme that contributes the major sterol esterification activity in the presence of oxygen
	CAGL0B03421g		1,37	1,13E-03	*HAP1*	Zinc finger transcription factor involved in the complex regulation of gene expression in response to levels of heme and oxygen; the S288C sequence differs from other strain backgrounds due to a Ty1 insertion in the carboxy terminus
	CAGL0D01980g		1,37	5,62E-04	*TGL1*	Steryl ester hydrolase, one of three gene products (Yeh1p, Yeh2p, Tgl1p) responsible for steryl ester hydrolase activity and involved in sterol homeostasis; localized to lipid particle membranes
	CAGL0G05962g		1,37	9,68E-04	*YHR140W*	Putative integral membrane protein of unknown function
	CAGL0C03872g	*CgTIR3*	1,36	1,45E-03	*TIR3*	Cell wall mannoprotein of the Srp1p/Tip1p family of serine-alanine-rich proteins; expressed under anaerobic conditions and required for anaerobic growth
	CAGL0C03289g	*CgYBT1*	1,35	3,83E-03	*YBT1*	Transporter of the ATP-binding cassette (ABC) family involved in bile acid transport; similar to mammalian bile transporters
	CAGL0L09383g		1,35	6,83E-03	*SUT2*	Putative transcription factor; multicopy suppressor of mutations that cause low activity of the cAMP/protein kinase A pathway; highly similar to Sut1p
	CAGL0E02651g		1,35	1,97E-03	*YSP3*	Putative precursor to the subtilisin-like protease III

CAGL0C01595g		1,35	1,85E-03	*HIS7*	Imidazole glycerol phosphate synthase (glutamine amidotransferase:cyclase), catalyzes the fifth step of histidine biosynthesis and also produces 5-aminoimidazole-4-carboxamide ribotide (AICAR), a purine precursor
CAGL0F04785g		1,35	7,08E-03	*VHS2*	Cytoplasmic protein of unknown function; identified as a high-copy suppressor of the synthetic lethality of a sis2 sit4 double mutant, suggesting a role in G1/S phase progression; similar to Mlf3p
CAGL0J07876g		1,34	7,45E-04	*RTC4*	Protein of unknown function; null mutation suppresses cdc13-1 temperature sensitivity; (GFP)-fusion protein localizes to both the cytoplasm and the nucleus
CAGL0G06050g		1,34	2,54E-03	-	Unknown
CAGL0A04081g		1,34	4,25E-03	*YLR194C*	Structural constituent of the cell wall attached to the plasma membrane by a GPI-anchor; expression is upregulated in response to cell wall stress
CAGL0M12947g	*CgPUP1*	1,34	2,01E-03	-	Mitochondria-localized protein
CAGL0G00858g		1,33	9,95E-04	*MID2*	O-glycosylated plasma membrane protein that acts as a sensor for cell wall integrity signaling and activates the pathway; interacts with Rom2p, a guanine nucleotide exchange factor for Rho1p, and with cell integrity pathway protein Zeo1p
CAGL0L04598g		1,33	8,18E-04	*DCS2*	Non-essential, stress induced regulatory protein; modulates m7G-oligoribonucleotide metabolism; inhibits Dcs1p; regulated by Msn2p, Msn4p, and the Ras-cAMP-cAPK signaling pathway; mutant has increased aneuploidy tolerance
CAGL0J02948g	*CgFCY2*	1,33	2,69E-03	*FCY22*	Putative purine-cytosine permease, very similar to Fcy2p but cannot substitute for its function
CAGL0L12892g		1,33	6,37E-04	*SFK1*	Plasma membrane protein that may act together with or upstream of Stt4p to generate normal levels of the essential phospholipid PI4P, at least partially mediates proper localization of Stt4p to the plasma membrane
CAGL0L10362g		1,33	1,47E-03	*YOR062C*	Protein of unknown function; similar to YKR075Cp and Reg1p; expression regulated by glucose and Rgt1p; GFP-fusion protein is induced in response to the DNA-damaging agent MMS
CAGL0J04004g		1,33	1,30E-03	*YOR228C*	Protein of unknown function, localized to the mitochondrial outer membrane
CAGL0L09251g		1,32	1,18E-03	*HAL1*	Cytoplasmic protein involved in halotolerance; decreases intracellular Na+ (via Ena1p) and increases intracellular K+ by decreasing efflux; expression repressed by Ssn6p-Tup1p and Sko1p and induced by NaCl, KCl, and sorbitol through Gcn4p
CAGL0K11297g		1,31	4,01E-03	*YDR248C*	Putative protein of unknown function; sequence similarity to bacterial and human gluconokinase; green fluorescent protein (GFP)-fusion protein localizes to the cytoplasm; upregulated by deletion of the RNAP-II associated factor, PAF1
CAGL0L07744g		1,31	7,76E-04	*ADP1*	Putative ATP-dependent permease of the ABC transporter family of proteins
CAGL0A01243g	*CgGIT1*	1,30	5,87E-03	-	Unknown
CAGL0I05676g		1,30	3,52E-03	*PRP2*	RNA-dependent ATPase in the DEAH-box family, required for activation of the spliceosome before the first transesterification step in RNA splicing; orthologous to human protein DHX16
CAGL0M04675g		1,30	6,55E-03	*RDL1*	Protein of unknown function containing a rhodanese-like domain; localized to the mitochondrial outer membrane
CAGL0J08613g		1,29	7,40E-04	*YVC1*	Vacuolar cation channel, mediates release of Ca(2+) from the vacuole in response to hyperosmotic shock
CAGL0C01771g		1,29	3,00E-03	*YBR241C*	Putative transporter, member of the sugar porter family; green fluorescent protein (GFP)-fusion protein localizes to the vacuolar membrane; YBR241C is not an essential gene
CAGL0E01969g		1,29	1,65E-03	*SMF1*	Divalent metal ion transporter with a broad specificity for di-valent and tri-valent metals; post-translationally regulated by levels of metal ions; member of the Nramp family of metal transport proteins
CAGL0L01287g	*CgUTR4*	1,29	9,04E-04	*UTR4*	Protein with sequence similarity to 2,3-diketo-5-methylthiopentyl-1-phosphate enolase-phosphatases, involved in methionine salvage; found in both the cytoplasm and nucleus
CAGL0L10043g		1,29	1,39E-03	*STD1*	Protein involved in control of glucose-regulated gene expression; interacts with kinase Snf1p, glucose sensors Snf3p and Rgt2p, TATA-binding Spt15p; regulator of transcription factor Rgt1p; interactions with Pma1p appear to propagate [GAR+]
CAGL0H09944g		1,29	4,05E-03	*ZTA1*	NADPH-dependent quinone reductase, GFP-tagged protein localizes to the cytoplasm and nucleus; has similarity to E. coli quinone oxidoreductase and to human zeta-crystallin
CAGL0G01100g		1,29	8,66E-04	*YLR345W*	Similar to 6-phosphofructo-2-kinase/fructose-2,6-bisphosphatase enzymes responsible for the metabolism of fructoso-2,6-bisphosphate; mRNA expression is repressed by the Rfx1p-Tup1p-Ssn6p repressor complex; YLR345W is not an essential gene
CAGL0H03135g		1,29	7,87E-03	*MF(ALPHA)2*	Mating pheromone alpha-factor, made by alpha cells; interacts with mating type a cells to induce cell cycle arrest and other responses leading to mating; also encoded by MF(ALPHA)1, which is more highly expressed than MF(ALPHA)2
CAGL0J11308g		1,28	1,82E-03	*NPR1*	Protein kinase that stabilizes several plasma membrane amino acid transporters by antagonizing their ubiquitin-mediated degradation; phosphorylates Aly2p; negatively regulates Ldb19p-mediated endocytosis through phosphorylation of Ldb19p, which prevents its association with the plasma membrane; Npr1p activity is negatively regulated via phosphorylation by the TOR complex
CAGL0H07667g		1,28	1,84E-03	*PDE1*	Low-affinity cyclic AMP phosphodiesterase, controls glucose and intracellular acidification-induced cAMP signaling, target of the cAMP-protein kinase A (PKA) pathway; glucose induces transcription and inhibits translation
CAGL0I10648g	*CgASN2*	1,28	1,48E-03	*ASN2*	Asparagine synthetase, isozyme of Asn1p; catalyzes the synthesis of L-asparagine from L-aspartate in the asparagine biosynthetic pathway
CAGL0A01045g		1,28	2,58E-03	*FUM1*	Fumarase, converts fumaric acid to L-malic acid in the TCA cycle; cytosolic and mitochondrial distribution determined by the N-terminal targeting sequence, protein conformation, and status of glyoxylate shunt; phosphorylated in mitochondria
CAGL0H09130g		1,28	2,12E-03	*MNN4*	Putative positive regulator of mannosylphosphate transferase (Mnn6p), involved in mannosylphosphorylation of N-linked oligosaccharides; expression increases in late-logarithmic and stationary growth phases
CAGL0J01699g		1,28	3,66E-03	*YPR010C-A*	Putative protein of unknown function; conserved among Saccharomyces sensu stricto species
CAGL0K00803g	*CgTRX2*	1,28	1,96E-03	*TRX1*	Cytoplasmic thioredoxin isoenzyme of the thioredoxin system which protects cells against oxidative and reductive stress, forms LMA1 complex with Pbi2p, acts as a cofactor for Tsa1p, required for ER-Golgi transport and vacuole inheritance
CAGL0F04609g		1,27	1,41E-03	*EDE1*	Endocytic protein; involved in a network of interactions with other endocytic proteins, binds membranes in a ubiquitin-dependent manner, may also bind ubiquitinated membrane-associated proteins; interacts with Cmk2 and functions upstream of CMK2 in regulating non-apoptotic cell death
CAGL0L05654g		1,27	3,58E-03	*TRK1*	Component of the Trk1p-Trk2p potassium transport system; 180 kDa high affinity potassium transporter; phosphorylated in vivo and interacts physically with the phosphatase Ppz1p, suggesting Trk1p acitivy is regulated by phosphorylation

CAGL0C05533g		1,27	1,39E-03	*AIM6*	Putative protein of unknown function, required for respiratory growth; YDL237W is not an essential gene
CAGL0M11726g		1,27	7,91E-03	*CCW12*	Putative GPI-linked cell wall adhesin-like protein;
CAGL0M07293g		1,27	7,46E-03	*PDR12*	Plasma membrane ATP-binding cassette (ABC) transporter, weak-acid-inducible multidrug transporter required for weak organic acid resistance; induced by sorbate and benzoate and regulated by War1p; mutants exhibit sorbate hypersensitivity
CAGL0E02321g		1,26	7,85E-04	*PLB3*	Phospholipase B (lysophospholipase) involved in phospholipid metabolism; hydrolyzes phosphatidylinositol and phosphatidylserine and displays transacylase activity in vitro
CAGL0F05687g		1,26	1,89E-03	*YDR186C*	Putative protein of unknown function; may interact with ribosomes, based on co-purification experiments; green fluorescent protein (GFP)-fusion protein localizes to the cytoplasm
CAGL0I05082g		1,26	1,25E-03	*PIN4*	Protein involved in G2/M phase progression and response to DNA damage, interacts with Rad53p; contains an RNA recognition motif, a nuclear localization signal, and several SQ/TQ cluster domains; hyperphosphorylated in response to DNA damage
CAGL0I11011g		1,25	8,75E-03	*NA*	NA
CAGL0C04191g		1,25	4,46E-03	*UGA2*	Succinate semialdehyde dehydrogenase involved in the utilization of gamma-aminobutyrate (GABA) as a nitrogen source; part of the 4-aminobutyrate and glutamate degradation pathways; localized to the cytoplasm
CAGL0C01397g		1,24	2,13E-03	*PFK26*	6-phosphofructo-2-kinase, inhibited by phosphoenolpyruvate and sn-glycerol 3-phosphate; has negligible fructose-2,6-bisphosphatase activity; transcriptional regulation involves protein kinase A
CAGL0J00803g		1,24	3,20E-03	*MHP1*	Microtubule-associated protein involved in assembly and stabilization of microtubules; overproduction results in cell cycle arrest at G2 phase; similar to Drosophila protein MAP and to mammalian MAP4 proteins
CAGL0L06864g		1,24	1,56E-03	*SIP5*	Protein of unknown function; interacts with both the Reg1p/Glc7p phosphatase and the Snf1p kinase
CAGL0I03872g		1,24	1,22E-03	*RGT2*	Plasma membrane high glucose sensor that regulates glucose transport; contains 12 predicted transmembrane segments and a long C-terminal tail required for induction of hexose transporters; highly similar to Snf3p
CAGL0L06248g		1,24	8,46E-04	*YBR085C-A*	Putative protein of unknown function; green fluorescent protein (GFP)-fusion protein localizes to the cytoplasm and to the nucleus
CAGL0K10604g		1,24	4,48E-03	*CMK1*	Calmodulin-dependent protein kinase; may play a role in stress response, many Ca++/calmodulin dependent phosphorylation substrates demonstrated in vitro, amino acid sequence similar to Cmk2p and mammalian Cam Kinase II
CAGL0J03256g		1,24	1,05E-03	*PTP3*	Phosphotyrosine-specific protein phosphatase involved in the inactivation of mitogen-activated protein kinase (MAPK) during osmolarity sensing; dephosporylates Hog1p MAPK and regulates its localization; localized to the cytoplasm
CAGL0L09933g		1,23	4,45E-03	*CUE5*	Protein containing a CUE domain that binds ubiquitin, which may facilitate intramolecular monoubiquitination; green fluorescent protein (GFP)-fusion protein localizes to the cytoplasm in a punctate pattern
CAGL0C04785g		1,23	5,13E-03	*YJR115W*	Putative protein of unknown function
CAGL0D00990g		1,23	9,76E-04	*YDL057W*	Putative protein of unknown function; YDL057W is not an essential gene
CAGL0K01133g		1,23	2,21E-03	*TWF1*	Twinfilin, highly conserved actin monomer-sequestering protein involved in regulation of the cortical actin cytoskeleton, composed of two cofilin-like regions, localizes actin monomers to sites of rapid filament assembly
CAGL0H09614g		1,23	5,89E-03	*TIR1*	Cell wall mannoprotein of the Srp1p/Tip1p family of serine-alanine-rich proteins; expression is downregulated at acidic pH and induced by cold shock and anaerobiosis; abundance is increased in cells cultured without shaking
CAGL0K04257g		1,23	1,01E-03	*RME1*	Zinc finger protein involved in control of meiosis; prevents meiosis by repressing IME1 expression and promotes mitosis by activating CLN2 expression; directly repressed by a1-alpha2 regulator; mediates cell type control of sporulation
CAGL0L09108g		1,22	1,53E-03	*PDH1*	Mitochondrial protein that participates in respiration, induced by diauxic shift; homologous to E. coli PrpD, may take part in the conversion of 2-methylcitrate to 2-methylisocitrate
CAGL0E06666g	*CgEPA2*	1,22	2,14E-03	-	Epithelial adhesion protein
CAGL0H02695g		1,21	1,29E-03	*GLG1*	Self-glucosylating initiator of glycogen synthesis, also glucosylates n-dodecyl-beta-D-maltoside; similar to mammalian glycogenin
CAGL0I03476g		1,21	8,12E-03	*DHH1*	Cytoplasmic DExD/H-box helicase, stimulates mRNA decapping; coordinates distinct steps in mRNA function and decay, interacts with both the decapping and deadenylase complexes, may have a role in mRNA export and translation; C-terminus of Dhh1p interacts with Ngr1p and promotes POR1, but not EDC1 mRNA decay
CAGL0D01100g		1,21	3,25E-03	*PFK26*	6-phosphofructo-2-kinase, inhibited by phosphoenolpyruvate and sn-glycerol 3-phosphate; has negligible fructose-2,6-bisphosphatase activity; transcriptional regulation involves protein kinase A
CAGL0I08943g		1,21	4,43E-03	*PES4*	Poly(A) binding protein, suppressor of DNA polymerase epsilon mutation, similar to Mip6p
CAGL0H09592g		1,21	3,55E-03	-	Putative GPI-linked cell wall protein
CAGL0B02755g		1,21	1,49E-03	*YLR361C-A*	Putative protein of unknown function
CAGL0F01947g	*CgIRC15*	1,21	2,92E-03	*IRC15*	Microtubule associated protein; regulates microtubule dynamics; required for accurate meiotic chromosome segregation; null mutant displays large budded cells due to delayed mitotic progression, increased levels of spontaneous Rad52 foci
CAGL0M09108g		1,20	4,31E-03	*JSN1*	Member of the Puf family of RNA-binding proteins, interacts with mRNAs encoding membrane-associated proteins; involved in localizing the Arp2/3 complex to mitochondria; overexpression causes increased sensitivity to benomyl
CAGL0M05401g		1,20	8,32E-03	*YBR201C-A*	Putative protein of unknown function
CAGL0I01980g		1,20	1,92E-03	*YSP1*	Mitochondrial protein with a potential role in promoting mitochondrial fragmentation during programmed cell death in response to high levels of alpha-factor mating pheromone or the drug amiodarone
CAGL0L06358g		1,20	1,33E-03	*TMS1*	Vacuolar membrane protein of unknown function that is conserved in mammals; predicted to contain eleven transmembrane helices; interacts with Pdr5p, a protein involved in multidrug resistance
CAGL0K12716g		1,20	5,27E-03	*YFL040W*	Putative transporter, member of the sugar porter family; YFL040W is not an essential gene
CAGL0H01177g		1,20	2,70E-03	*DPP1*	Diacylglycerol pyrophosphate (DGPP) phosphatase, zinc-regulated vacuolar membrane-associated lipid phosphatase, dephosphorylates DGPP to phosphatidate (PA) and Pi, then PA to diacylglycerol; involved in lipid signaling and cell metabolism
CAGL0E01859g	*CgYPS10*	1,19	1,93E-03	-	Putative aspartic protease

CAGL0L12056g	*CgBMH1*	1,19	1,25E-03	*BMH1*	14-3-3 protein, major isoform; controls proteome at post-transcriptional level, binds proteins and DNA, involved in regulation of many processes including exocytosis, vesicle transport, Ras/MAPK signaling, and rapamycin-sensitive signaling
CAGL0K04675g		1,19	1,74E-03	-	Unknown
CAGL0E01419g	*CgYPS2*	1,18	1,81E-03	*MKC7*	GPI-anchored aspartyl protease, member of the yapsin family of proteases involved in cell wall growth and maintenance; shares functions with Yap3p and Kex2p
CAGL0L10142g	*CgRSB1*	1,18	2,64E-03	*RSB1*	Suppressor of sphingoid long chain base (LCB) sensitivity of an LCB-lyase mutation; putative integral membrane transporter or flippase that may transport LCBs from the cytoplasmic side toward the extracytoplasmic side of the membrane
CAGL0H01287g		1,18	2,68E-03	*SSD1*	Translational repressor with a role in polar growth and wall integrity; regulated by Cbk1p phosphorylation to effect bud-specific translational control and localization of specific mRNAs; interacts with TOR pathway components; contains a functional N-terminal nuclear localization sequence and nucleocytoplasmic shuttling appears to be critical to Ssd1p function
CAGL0K04037g	*CgFKS2*	1,18	6,33E-03	*GSC2*	Catalytic subunit of 1,3-beta-glucan synthase, involved in formation of the inner layer of the spore wall; activity positively regulated by Rho1p and negatively by Smk1p; has similarity to an alternate catalytic subunit, Fks1p (Gsc1p)
CAGL0M08800g	*CgYAP6*	1,17	4,24E-03	*YAP6*	Basic leucine zipper (bZIP) transcription factor; physically interacts with the Tup1-Cyc8 complex and recruits Tup1p to its targets; overexpression increases sodium and lithium tolerance; computational analysis suggests a role in regulation of expression of genes involved in carbohydrate metabolism
CAGL0M06457g		1,17	2,49E-03	*GDT1*	Putative protein of unknown function; expression is reduced in a gcr1 null mutant; GFP-fusion protein localizes to the vacuole; expression pattern and physical interactions suggest a possible role in ribosome biogenesis
CAGL0J01892g	*CgPAN1*	1,17	3,39E-03	*PAN1*	Part of actin cytoskeleton-regulatory complex Pan1p-Sla1p-End3p, associates with actin patches on the cell cortex; promotes protein-protein interactions essential for endocytosis; binds to and activates the Arp2/3 complex in vitro; previously thought to be a subunit of poly(A) ribonuclease
CAGL0L06094g	*CgSTR3*	1,17	6,84E-03	*STR3*	Peroxisomal cystathionine beta-lyase, converts cystathionine into homocysteine; may be redox regulated by Gto1p
CAGL0L09691g		1,17	1,59E-03	*PUT3*	Transcriptional activator of proline utilization genes, constitutively binds PUT1 and PUT2 promoter sequences as a dimer and undergoes a conformational change to form the active state; differentially phosphorylated in the presence of different nitrogen sources; has a Zn(2)-Cys(6) binuclear cluster domain
CAGL0E06380g		1,16	5,59E-03	*YKL151C*	NADHX dehydratase; converts (S)-NADHX to NADH in an ATP-dependent manner; homologous to Carkd in mammals, and the C-terminal domain of YjeF in E.coli; enzyme is widespread in eukaryotes, prokaryotes and archaea; YKL151C promoter contains STREs (stress response elements) and expression is induced by heat shock or methyl methanesulfonate; green fluorescent protein (GFP)-fusion protein localizes to the cytoplasm
CAGL0C03850g		1,16	4,79E-03	*DOT5*	Nuclear thiol peroxidase which functions as an alkyl-hydroperoxide reductase during post-diauxic growth
CAGL0E02255g		1,16	2,89E-03	*ZEO1*	Peripheral membrane protein of the plasma membrane that interacts with Mid2p; regulates the cell integrity pathway mediated by Pkc1p and Slt2p; the authentic protein is detected in a phosphorylated state in highly purified mitochondria
CAGL0K12760g		1,16	1,65E-03	*YFL042C*	Putative protein of unknown function; YFL042C is not an essential gene
CAGL0C05137g	*CgGPD2*	1,15	2,09E-03	*GPD2*	NAD-dependent glycerol 3-phosphate dehydrogenase, homolog of Gpd1p, expression is controlled by an oxygen-independent signaling pathway required to regulate metabolism under anoxic conditions; located in cytosol and mitochondria
CAGL0L08140g		1,15	1,85E-03	*BPH1*	PProtein homologous to human Chediak-Higashi syndrome and murine Beige proteins, which are implicated in disease syndromes due to defective lysosomal trafficking; mutant phenotype and genetic interactions suggest a role in protein sorting
CAGL0H02497g		1,15	2,14E-03	*GFD1*	Coiled-coiled protein of unknown function, identified as a high-copy suppressor of a dbp5 mutation
CAGL0G05093g		1,15	3,81E-03	*YDR061W*	Protein with similarity to ATP-binding cassette (ABC) transporter family members; lacks predicted membrane-spanning regions; transcriptionally activated by Yrm1p along with genes involved in multidrug resistance
CAGL0D01474g		1,14	1,80E-03	*AIM3*	Protein interacting with Rvs167p; null mutant is viable and displays elevated frequency of mitochondrial genome loss
CAGL0I03168g		1,14	3,78E-03	*YEL023C*	Putative protein of unknown function; expression is increased greatly during sporulation; YEL023C is not an essential gene
CAGL0J01331g		1,14	4,47E-03	-	Unknown
CAGL0L02937g	*CgHIS3*	1,14	1,41E-03	*HIS3*	Imidazoleglycerol-phosphate dehydratase, catalyzes the sixth step in histidine biosynthesis; mutations cause histidine auxotrophy and sensitivity to Cu, Co, and Ni salts; transcription is regulated by general amino acid control via Gcn4p
CAGL0C01243g		1,13	4,80E-03	*HIS5*	Histidinol-phosphate aminotransferase, catalyzes the seventh step in histidine biosynthesis; responsive to general control of amino acid biosynthesis; mutations cause histidine auxotrophy and sensitivity to Cu, Co, and Ni salts
CAGL0I02200g	*CgSOL3*	1,13	9,52E-03	*SOL3*	6-phosphogluconolactonase, catalyzes the second step of the pentose phosphate pathway; weak multicopy suppressor of los1-1 mutation; homologous to Sol2p and Sol1p
CAGL0A00539g		1,13	1,94E-03	*COG7*	Component of the conserved oligomeric Golgi complex (Cog1p through Cog8p), a cytosolic tethering complex that functions in protein trafficking to mediate fusion of transport vesicles to Golgi compartments
CAGL0I07887g		1,13	1,55E-03	*ATG19*	Receptor protein for the cytoplasm-to-vacuole targeting (Cvt) pathway; delivers cargo proteins aminopeptidase I (Ape1p) and alpha-mannosidase (Ams1p) to the phagophore assembly site for packaging into Cvt vesicles
CAGL0C04939g		1,12	6,81E-03	*YJR107W*	Putative protein of unknown function; has sequence or structural similarity to lipases
CAGL0D00286g	*CgBMT1*	1,12	1,68E-03	-	Beta mannosyltransferase
CAGL0I02046g		1,12	2,66E-03	-	Unknown
CAGL0L00803g		1,12	4,22E-03	*PIG2*	Putative type-1 protein phosphatase targeting subunit that tethers Glc7p type-1 protein phosphatase to Gsy2p glycogen synthase
CAGL0M02629g		1,11	5,93E-03	*ISU1*	Conserved protein of the mitochondrial matrix, performs a scaffolding function during assembly of iron-sulfur clusters, interacts physically and functionally with yeast frataxin (Yfh1p); isu1 isu2 double mutant is inviable
CAGL0L00957g		1,11	3,58E-03	*CAJ1*	Nuclear type II J heat shock protein of the E. coli dnaJ family, contains a leucine zipper-like motif, binds to non-native substrates for presentation to Ssa3p, may function during protein translocation, assembly and disassembly
CAGL0H09218g	*CgSDT1*	1,11	1,64E-03	*SDT1*	Pyrimidine nucleotidase; overexpression suppresses the 6-AU sensitivity of transcription elongation factor S-II, as well as resistance to other pyrimidine derivatives
CAGL0K10736g	*CgCYB2*	1,10	3,34E-03	*CYB2*	Cytochrome b2 (L-lactate cytochrome-c oxidoreductase), component of the mitochondrial intermembrane space, required for lactate utilization; expression is repressed by glucose and anaerobic conditions
CAGL0M00550g		1,10	2,00E-03	*STR2*	Cystathionine gamma-synthase, converts cysteine into cystathionine

CAGL0L07810g		1,10	4,06E-03	*SAT4*	Ser/Thr protein kinase involved in salt tolerance; funtions in regulation of Trk1p-Trk2p potassium transporter; partially redundant with Hal5p; has similarity to Npr1p
CAGL0J03916g		1,10	2,21E-03	*HES1*	Protein implicated in the regulation of ergosterol biosynthesis; one of a seven member gene family with a common essential function and non-essential unique functions; similar to human oxysterol binding protein (OSBP)
CAGL0H03311g		1,10	3,78E-03	*YGL081W*	Putative protein of unknown function; non-essential gene; interacts genetically with CHS5, a gene involved in chitin biosynthesis
CAGL0C02519g		1,10	1,83E-03	*MIG3*	Probable transcriptional repressor involved in response to toxic agents such as hydroxyurea that inhibit ribonucleotide reductase; phosphorylation by Snf1p or the Mec1p pathway inactivates Mig3p, allowing induction of damage response genes
CAGL0G02893g		1,09	2,14E-03	*POS5*	Mitochondrial NADH kinase, phosphorylates NADH; also phosphorylates NAD(+) with lower specificity; required for the response to oxidative stress
CAGL0L02607g		1,09	3,40E-03	*YHR202W*	Putative protein of unknown function; green fluorescent protein (GFP)-fusion protein localizes to the vacuole, while HA-tagged protein is found in the soluble fraction, suggesting cytoplasmic localization
CAGL0K09790g		1,09	5,71E-03	*SRV2*	CAP (cyclase-associated protein) subunit of adenylyl cyclase complex; N-terminus binds adenylyl cyclase and facilitates activation by RAS; C-terminus binds ADP-actin monomers, facilitating regulation of actin dynamics and cell morphogenesis
CAGL0L11440g		1,09	3,20E-03	*TCB3*	Lipid-binding ER protein, enriched at ER-plasma membrane contact sites; localized to the bud via specific mRNA transport; non-tagged protein detected in a phosphorylated state in mitochondria; C-termini of Tcb1p, Tcb2p and Tcb3p interact
CAGL0I01496g		1,09	8,26E-03	*SSC1*	Hsp70 family ATPase, constituent of the import motor component of the Translocase of the Inner Mitochondrial membrane (TIM23 complex); involved in protein translocation and folding; subunit of SceI endonuclease
CAGL0E02035g		1,09	8,22E-03	*MCH4*	Protein with similarity to mammalian monocarboxylate permeases, which are involved in transport of monocarboxylic acids across the plasma membrane; mutant is not deficient in monocarboxylate transport
CAGL0M08206g		1,09	3,64E-03	*YJL171C*	GPI-anchored cell wall protein of unknown function; induced in response to cell wall damaging agents and by mutations in genes involved in cell wall biogenesis; sequence similarity to YBR162C/TOS1, a covalently bound cell wall protein
CAGL0K05687g		1,08	5,93E-03	-	Unknown
CAGL0A02299g		1,08	5,28E-03	-	Unknown
CAGL0M13783g		1,08	2,82E-03	*UBP15*	Ubiquitin-specific protease involved in protein deubiquitination; catalytic activity regulated by an N-terminal TRAF-like domain and and C-terminal sequences; physically interacts with anaphase-promoting complex/cyclosome (APC/C) activator, Cdh1p
CAGL0M08552g		1,08	2,29E-03	*PMP3*	Small plasma membrane protein related to a family of plant polypeptides that are overexpressed under high salt concentration or low temperature, not essential for viability, deletion causes hyperpolarization of the plasma membrane potential
CAGL0G08646g		1,08	3,18E-03	*POG1*	Nuclear chromatin-associated protein of unknown function; overexpression promotes recovery from pheromone induced arrest and suppresses the stress sensitivity caused by a mutation in the E3 ubiquitin ligase Rsp5p; binds upstream of BAR1 and cell cycle-related genes; potential Cdc28p substrate; SBF regulated
CAGL0G01738g		1,08	2,90E-03	*PIL1*	Primary component of eisosomes, which are large immobile cell cortex structures associated with endocytosis; null mutants show activation of Pkc1p/Ypk1p stress resistance pathways; detected in phosphorylated state in mitochondria; member of the BAR domain family
CAGL0A00517g		1,08	2,58E-03	*PMC1*	Vacuolar Ca2+ ATPase involved in depleting cytosol of Ca2+ ions; prevents growth inhibition by activation of calcineurin in the presence of elevated concentrations of calcium; similar to mammalian PMCA1a
CAGL0F01419g	*CgAUS1*	1,07	6,12E-03	*AUS1*	Plasma membrane sterol transporter of the ATP-binding cassette family; required, along with Pdr11p, for uptake of exogenous sterols and their incorporation into the plasma membrane; activity is stimulated by phosphatidylserine; sterol uptake is required for anaerobic growth because sterol biosynthesis requires oxygen
CAGL0I10626g		1,07	2,77E-03	*YGR125W*	Putative protein of unknown function; deletion mutant has decreased rapamycin resistance but normal wormannin resistance; green fluorescent protein (GFP)-fusion protein localizes to the vacuole
CAGL0M08602g		1,07	6,74E-03	*CCC2*	Cu(+2)-transporting P-type ATPase, required for export of copper from the cytosol into an extracytosolic compartment; has similarity to human proteins involved in Menkes and Wilsons diseases
CAGL0J09416g		1,07	2,21E-03	*SNA4*	Protein of unknown function, localized to the vacuolar outer membrane; predicted to be palmitoylated
CAGL0M07568g		1,06	3,18E-03	*UBC7*	Ubiquitin conjugating enzyme, involved in the ER-associated protein degradation pathway; requires Cue1p for recruitment to the ER membrane; proposed to be involved in chromatin assembly
CAGL0C03509g		1,06	3,78E-03	*FPK1*	Ser/Thr protein kinase that regulates the putative phospholipid translocases Lem3p-Dnf1p/Dnf2p; phosphorylates and inhibits upstream inhibitory kinase, Ypk1p; localizes to the cytoplasm, early endosome/TGN compartments, and plasma membrane
CAGL0K04367g		1,06	2,24E-03	*MUP1*	High affinity methionine permease, integral membrane protein with 13 putative membrane-spanning regions; also involved in cysteine uptake
CAGL0I02552g	*CgSTB5*	1,06	4,89E-03	*STB5*	Transcription factor, involved in regulating multidrug resistance and oxidative stress response; forms a heterodimer with Pdr1p; contains a Zn(II)2Cys6 zinc finger domain that interacts with a pleiotropic drug resistance element in vitro
CAGL0M13365g		1,05	3,61E-03	*SSP1*	Protein involved in the control of meiotic nuclear division and coordination of meiosis with spore formation; transcription is induced midway through meiosis
CAGL0L08712g		1,05	5,54E-03	*YPL014W*	Putative protein of unknown function; green fluorescent protein (GFP)-fusion protein localizes to the cytoplasm and to the nucleus
CAGL0M10549g		1,05	5,29E-03	*RCF2*	Cytochrome c oxidase subunit; has a role in assembly of respiratory supercomplexes; similar to Rcf1p, and either Rcf1p or Rcf2p is required for late-stage assembly of the Cox12p and Cox13p subunits and for cytochrome c oxidase activity; associates with the cytochrome c oxidase - cytochrome bc1 supercomplex; null mutant accumulates reactive oxygen species; member of the conserved hypoxia induced gene family; C. elegans homolog is functional in yeast
CAGL0F06831g		1,05	3,28E-03	*MGA2*	ER membrane protein involved in regulation of OLE1 transcription, acts with homolog Spt23p; inactive ER form dimerizes and one subunit is then activated by ubiquitin/proteasome-dependent processing followed by nuclear targeting
CAGL0K12694g	*CgACT1*	1,04	7,91E-03	*ACT1*	Actin, structural protein involved in cell polarization, endocytosis, and other cytoskeletal functions
CAGL0B00242g		1,04	9,46E-03	*HMLALPHA1*	Silenced copy of ALPHA1 at HML, encoding a transcriptional coactivator involved in the regulation of mating-type alpha-specific gene expression
CAGL0A02024g		1,04	3,00E-03	*LAG2*	Protein that negatively regulates the SCF E3-ubiquitin ligase by interacting with and preventing neddyation of the cullin subunit, Cdc53p; longevity determinant that is preferentially expressed in young cells; similar to mammalian Cand1
CAGL0L00649g		1,04	2,83E-03	*ACS1*	Acetyl-coA synthetase isoform which, along with Acs2p, is the nuclear source of acetyl-coA for histone acetylation; expressed during growth on nonfermentable carbon sources and under aerobic conditions

CAGL0F07777g		1,04	6,07E-03	*ALD3*	Cytoplasmic aldehyde dehydrogenase, involved in beta-alanine synthesis; uses NAD+ as the preferred coenzyme; very similar to Ald2p; expression is induced by stress and repressed by glucose
CAGL0F02387g	*CgPHO87*	1,04	8,27E-03	*PHO90*	Low-affinity phosphate transporter; deletion of pho84, pho87, pho89, pho90, and pho91 causes synthetic lethality; transcription independent of Pi and Pho4p activity; overexpression results in vigorous growth
CAGL0I03960g	*CgVMA1*	1,03	5,14E-03	*VMA1*	Subunit A of the eight-subunit V1 peripheral membrane domain of the vacuolar H+-ATPase; protein precursor undergoes self-catalyzed splicing to yield the extein Tfp1p and the intein Vde (PI-SceI), which is a site-specific endonuclease
CAGL0D02134g		1,03	3,94E-03	*YKL133C*	Putative protein of unknown function; has similarity to Mgr3p, but unlike MGR3, is not required for growth of cells lacking the mitochondrial genome (null mutation does not confer a petite-negative phenotype)
CAGL0E00891g		1,03	5,63E-03	*STB3*	Ribosomal RNA processing element (RRPE)-binding protein involved in the glucose-induced transition from quiescence to growth; restricted to nucleus in quiescent cells, released into cytoplasm after glucose repletion; binds Sin3p
CAGL0K04477g		1,03	3,03E-03	*ERG25*	C-4 methyl sterol oxidase, catalyzes the first of three steps required to remove two C-4 methyl groups from an intermediate in ergosterol biosynthesis; mutants accumulate the sterol intermediate 4,4-dimethylzymosterol
CAGL0M11484g		1,03	5,85E-03	*ARO1*	Pentafunctional arom protein, catalyzes steps 2 through 6 in the biosynthesis of chorismate, which is a precursor to aromatic amino acids
CAGL0J01529g		1,02	2,93E-03	*RCO1*	Essential subunit of the histone deacetylase Rpd3S complex; interacts with Eaf3p
CAGL0L08338g		1,02	8,50E-03	*TPM2*	Minor isoform of tropomyosin, binds to and stabilizes actin cables and filaments, which direct polarized cell growth and the distribution of several organelles; appears to have distinct and also overlapping functions with Tpm1p
CAGL0I10054g		1,02	3,68E-03	*SKN1*	Protein involved in sphingolipid biosynthesis; type II membrane protein with similarity to Kre6p
CAGL0A03344g		1,02	3,47E-03	*ASI3*	Putative integral membrane E3 ubiquitin ligase; acts with Asi1p and Asi2p to ensure the fidelity of SPS-sensor signalling by maintaining the dormant repressed state of gene expression in the absence of inducing signals
CAGL0L03982g		1,01	7,18E-03	*MLS1*	Malate synthase, enzyme of the glyoxylate cycle, involved in utilization of non-fermentable carbon sources; expression is subject to carbon catabolite repression; localizes in peroxisomes during growth in oleic acid medium
CAGL0J08349g		1,01	4,50E-03	*CAF120*	Part of the evolutionarily-conserved CCR4-NOT transcriptional regulatory complex involved in controlling mRNA initiation, elongation, and degradation
CAGL0E03652g		1,00	9,80E-03	*ISA1*	Protein required for maturation of mitochondrial [4Fe-4S] proteins; functions in a complex with Isa2p and possibly Iba57p; isa1 deletion causes loss of mitochondrial DNA and respiratory deficiency; depletion reduces growth on nonfermentable carbon sources; functional ortholog of bacterial A-type ISC proteins
CAGL0K04873g		1,00	5,05E-03	*YDL233W*	Putative protein of unknown function; green fluorescent protein (GFP)-fusion protein localizes to the cytoplasm and nucleus; YDL233W is not an essential gene
CAGL0F01287g	*CgGAS5*	1,00	6,74E-03	*GAS5*	1,3-beta-glucanosyltransferase, has similarity to Gas1p; localizes to the cell wall
CAGL0D06512g		1,00	5,16E-03	*SDC25*	ANNOTATED BY YGOB - skipping over frameshift in strain S288c

DOWN-REGULATED GENES

ORF	Gene	log_2FC (wt AC/wt CTRL)	p_value	*S. cerevisiae* orthologue	Function
CAGL0I01232g		-1,01	5,26E-03	*ERP5*	Protein with similarity to Emp24p and Erv25p, member of the p24 family involved in ER to Golgi transport
CAGL0H05643g		-1,02	9,58E-03	*RPS6A*	Protein component of the small (40S) ribosomal subunit; identical to Rps6Bp and has similarity to rat S6 ribosomal protein
CAGL0A00979g		-1,03	6,06E-03	*RPL38*	Protein component of the large (60S) ribosomal subunit, has similarity to rat L38 ribosomal protein
CAGL0I06985g		-1,03	5,79E-03	*FRA2*	Protein involved in negative regulation of transcription of iron regulon; forms an iron independent complex with Fra2p, Grx3p, and Grx4p; null mutant fails to repress iron regulon and is sensitive to nickel
CAGL0K08954g		-1,04	3,18E-03	*CDC31*	Calcium-binding component of the spindle pole body (SPB) half-bridge, required for SPB duplication in mitosis and meiosis II; homolog of mammalian centrin; binds multiubiquitinated proteins and is involved in proteasomal protein degradation
CAGL0G00682g		-1,04	6,90E-03	*ERP1*	Protein that forms a heterotrimeric complex with Erp2p, Emp24p, and Erv25p; member, along with Emp24p and Erv25p, of the p24 family involved in ER to Golgi transport and localized to COPII-coated vesicles
CAGL0K07414g		-1,05	6,43E-03	-	Ortholog(s) have cell surface, cytosolic large ribosomal subunit
CAGL0J10164g	*CgRPL16A*	-1,05	8,64E-03	*RPL16B*	N-terminally acetylated protein component of the large (60S) ribosomal subunit, binds to 5.8 S rRNA; has similarity to Rpl16Ap, E. coli L13 and rat L13a ribosomal proteins; transcriptionally regulated by Rap1p
CAGL0L13156g		-1,05	5,85E-03	*RFU1*	Protein that inhibits Doa4p deubiquitinating activity; contributes to ubiquitin homeostasis by regulating the conversion of free ubiquitin chains to ubiquitin monomers by Doa4p; GFP-fusion protein localizes to endosomes
CAGL0E06028g		-1,05	3,79E-03	*ALG5*	UDP-glucose:dolichyl-phosphate glucosyltransferase, involved in asparagine-linked glycosylation in the endoplasmic reticulum
CAGL0L04840g		-1,05	9,35E-03	*RPS23A*	Ribosomal protein 28 (rp28) of the small (40S) ribosomal subunit, required for translational accuracy; nearly identical to Rps23Bp and similar to E. coli S12 and rat S23 ribosomal proteins; deletion of both RPS23A and RPS23B is lethal
CAGL0G06490g		-1,06	3,87E-03	*RPS7B*	Protein component of the small (40S) ribosomal subunit, nearly identical to Rps7Ap; interacts with Kti11p; deletion causes hypersensitivity to zymocin; has similarity to rat S7 and Xenopus S8 ribosomal proteins
CAGL0G08734g		-1,06	5,64E-03	*RPL9B*	Protein component of the large (60S) ribosomal subunit, nearly identical to Rpl9Ap and has similarity to E. coli L6 and rat L9 ribosomal proteins
CAGL0J06952g		-1,06	2,95E-03	*IDI1*	Isopentenyl diphosphate:dimethylallyl diphosphate isomerase (IPP isomerase), catalyzes an essential activation step in the isoprenoid biosynthetic pathway; required for viability
CAGL0L13222g		-1,06	4,21E-03	*SIC1*	Cyclin-dependent kinase inhibitor (CKI); inhibitor of Cdc28-Clb kinase complexes that controls G1/S phase transition, preventing premature S phase and ensuring genomic integrity; phosphorylation targets Sic1p for SCF(CDC4)-dependent turnover; functional homolog of mammalian Kip1
CAGL0C02761g		-1,06	4,14E-03	*ERP2*	Protein that forms a heterotrimeric complex with Erp1p, Emp24p, and Erv25p; member, along with Emp24p and Erv25p, of the p24 family involved in ER to Golgi transport and localized to COPII-coated vesicles

CAGL0K09240g		-1,07	2,56E-03	*TAH1*	Component of the conserved R2TP complex (Rvb1-Rvb2-Tah1-Pih1); contains a single TPR domain with at least two TPR motifs; R2TP complex interacts with Hsp90 (Hsp82p and Hsc82p) to mediate assembly large protein complexes such as box C/D snoRNPs and RNA polymerase II
CAGL0J02354g		-1,07	6,21E-03	*RPL2B*	Protein component of the large (60S) ribosomal subunit, identical to Rpl2Ap and has similarity to E. coli L2 and rat L8 ribosomal proteins; expression is upregulated at low temperatures
CAGL0F02937g		-1,07	8,07E-03	*RPL12B*	Protein component of the large (60S) ribosomal subunit, nearly identical to Rpl12Ap; rpl12a rpl12b double mutant exhibits slow growth and slow translation; has similarity to E. coli L11 and rat L12 ribosomal proteins
CAGL0L01243g		-1,08	3,10E-03	*GDA1*	Guanosine diphosphatase located in the Golgi, involved in the transport of GDP-mannose into the Golgi lumen by converting GDP to GMP after mannose is transferred its substrate
CAGL0M08118g		-1,09	3,65E-03	*RPL17A*	Protein component of the large (60S) ribosomal subunit, nearly identical to Rpl17Bp and has similarity to E. coli L22 and rat L17 ribosomal proteins; copurifies with the Dam1 complex (aka DASH complex)
CAGL0D05742g		-1,09	3,90E-03	*OST1*	Alpha subunit of the oligosaccharyltransferase complex of the ER lumen, which catalyzes asparagine-linked glycosylation of newly synthesized proteins
CAGL0E03938g		-1,09	1,27E-02	*RPL8A*	Ribosomal protein L4 of the large (60S) ribosomal subunit, nearly identical to Rpl8Bp and has similarity to rat L7a ribosomal protein; mutation results in decreased amounts of free 60S subunits
CAGL0B01203g		-1,09	9,04E-03	*RPL37A*	Protein component of the large (60S) ribosomal subunit, has similarity to Rpl37Bp and to rat L37 ribosomal protein
CAGL0F01683g		-1,10	6,55E-03	*RPL22A*	Protein component of the large (60S) ribosomal subunit; exhibits similarity to Rpl22Bp and to rat L22 ribosomal protein; required for the oxidative stress response in yeast
CAGL0M07161g	*CgEGD2*	-1,10	4,85E-03	*EGD2*	Alpha subunit of the heteromeric nascent polypeptide-associated complex (NAC) involved in protein sorting and translocation, associated with cytoplasmic ribosomes
CAGL0L08118g		-1,10	7,60E-03	*RPS14A*	Ribosomal protein 59 of the small subunit, required for ribosome assembly and 20S pre-rRNA processing; mutations confer cryptopleurine resistance; nearly identical to Rps14Bp and similar to E. coli S11 and rat S14 ribosomal proteins
CAGL0L01969g		-1,10	7,34E-03	*YKL033W-A*	Putative protein of unknown function; similar to uncharacterized proteins from other fungi
CAGL0K04587g		-1,11	5,29E-03	*RPS22B*	Protein component of the small (40S) ribosomal subunit; nearly identical to Rps22Ap and has similarity to E. coli S8 and rat S15a ribosomal proteins
CAGL0L13200g		-1,11	2,50E-03	*BOS1*	v-SNARE (vesicle specific SNAP receptor), localized to the endoplasmic reticulum membrane and necessary for vesicular transport from the ER to the Golgi
CAGL0J00737g		-1,11	7,90E-03	*RPS27B*	Protein component of the small (40S) ribosomal subunit; nearly identical to Rps27Ap and has similarity to rat S27 ribosomal protein
CAGL0K02739g		-1,13	2,25E-03	*LAG1*	Ceramide synthase component, involved in synthesis of ceramide from C26(acyl)-coenzyme A and dihydrosphingosine or phytosphingosine, functionally equivalent to Lac1p
CAGL0B03927g		-1,13	7,16E-03	*TMA22*	Protein of unknown function; associates with ribosomes and has a putative RNA binding domain; interacts with Tma20p; similar to human GRAP and human DRP1, which interacts with human Tma20p homolog MCT-1
CAGL0G01826g		-1,14	5,20E-03	*RPL11A*	Protein of the large 60S ribosomal subunit, nearly identical to Rpl11Bp but expressed at twice the level; involved in ribosomal assembly; depletion causes degradation of 60S proteins and RNA; similar to E. coli L5 and rat L11
CAGL0L12540g		-1,14	1,69E-03	*EGD1*	Subunit beta1 of the nascent polypeptide-associated complex (NAC) involved in protein targeting, associated with cytoplasmic ribosomes; enhances DNA binding of the Gal4p activator; homolog of human BTF3b
CAGL0E04554g		-1,15	5,30E-03	-	Unknown
CAGL0K00605g		-1,15	1,68E-03	*CDC6*	Essential ATP-binding protein required for DNA replication, component of the pre-replicative complex (pre-RC) which requires ORC to associate with chromatin and is in turn required for Mcm2-7p DNA association; homologous to S. pombe Cdc18p
CAGL0M02365g		-1,16	5,87E-03	*PPT2*	Phosphopantetheine:protein transferase (PPTase), activates mitochondrial acyl carrier protein (Acp1p) by phosphopantetheinylation
CAGL0I00814g		-1,16	9,89E-03	*RPP1A*	Ribosomal stalk protein P1 alpha, involved in the interaction between translational elongation factors and the ribosome; accumulation of P1 in the cytoplasm is regulated by phosphorylation and interaction with the P2 stalk component
CAGL0I07557g		-1,17	7,28E-03	*RIB3*	3,4-dihydroxy-2-butanone-4-phosphate synthase (DHBP synthase), required for riboflavin biosynthesis from ribulose-5-phosphate, also has an unrelated function in mitochondrial respiration
CAGL0E04994g		-1,17	5,05E-03	*RPL9A*	Protein component of the large (60S) ribosomal subunit, nearly identical to Rpl9Bp and has similarity to E. coli L6 and rat L9 ribosomal proteins
CAGL0H08976g		-1,17	5,84E-03	*RPL1B*	N-terminally acetylated protein component of the large (60S) ribosomal subunit, nearly identical to Rpl1Ap and has similarity to E. coli L1 and rat L10a ribosomal proteins; rpl1a rpl1b double null mutation is lethal
CAGL0J07898g		-1,17	2,75E-03	*GIS2*	Translational activator for mRNAs with internal ribosome entry sites; associates with polysomes and binds to a specific subset of mRNAs; ortholog of human ZNF9/CNBP, a gene involved in myotonic dystrophy type 2
CAGL0L06886g		-1,18	1,04E-02	*RPL13A*	Protein component of the large (60S) ribosomal subunit, nearly identical to Rpl13Bp; not essential for viability; has similarity to rat L13 ribosomal protein
CAGL0K10906g		-1,18	4,10E-03	*RPL36A*	N-terminally acetylated protein component of the large (60S) ribosomal subunit, nearly identical to Rpl36Bp and has similarity to rat L36 ribosomal protein; binds to 5.8 S rRNA
CAGL0M10241g		-1,18	4,10E-03	*RPL14A*	N-terminally acetylated protein component of the large (60S) ribosomal subunit, nearly identical to Rpl14Bp and has similarity to rat L14 ribosomal protein; rpl14a csh5 double null mutant exhibits synthetic slow growth
CAGL0M06501g		-1,19	3,80E-03	*RPS9B*	Protein component of the small (40S) ribosomal subunit; nearly identical to Rps9Ap and has similarity to E. coli S4 and rat S9 ribosomal proteins
CAGL0L10978g		-1,19	1,84E-03	*CAF20*	Phosphoprotein of the mRNA cap-binding complex involved in translational control, repressor of cap-dependent translation initiation, competes with eIF4G for binding to eIF4E
CAGL0K03135g		-1,20	4,57E-03	*RPS20*	Protein component of the small (40S) ribosomal subunit; overproduction suppresses mutations affecting RNA polymerase III-dependent transcription; has similarity to E. coli S10 and rat S20 ribosomal proteins
CAGL0D00616g		-1,20	4,54E-03	*RPL31A*	Protein component of the large (60S) ribosomal subunit, nearly identical to Rpl31Bp and has similarity to rat L31 ribosomal protein; associates with the karyopherin Sxm1p; loss of both Rpl31p and Rpl39p confers lethality
CAGL0J07238g	*CgRPS12*	-1,21	2,55E-03	*RPS12*	Protein component of the small (40S) ribosomal subunit; has similarity to rat ribosomal protein S12

CAGL0G09130g		-1,23	1,68E-03	*RPL7B*	Protein component of the large (60S) ribosomal subunit, nearly identical to Rpl7Ap and has similarity to E. coli L30 and rat L7 ribosomal proteins; contains a conserved C-terminal Nucleic acid Binding Domain (NDB2)
CAGL0K06149g		-1,25	2,93E-03	*RPS17B*	Ribosomal protein 51 (rp51) of the small (40s) subunit; nearly identical to Rps17Ap and has similarity to rat S17 ribosomal protein
CAGL0M13563g		-1,26	5,53E-03	*GOT1*	Homodimeric protein that is packaged into COPII vesicles and cycles between the ER and Golgi; involved in secretory transport but not directly required for aspects of transport assayed in vitro; may influence membrane composition
CAGL0A01562g		-1,26	8,39E-03	*RPL24A*	Ribosomal protein L30 of the large (60S) ribosomal subunit, nearly identical to Rpl24Bp and has similarity to rat L24 ribosomal protein; not essential for translation but may be required for normal translation rate
CAGL0K07436g		-1,27	3,74E-03	*YHM2*	Carrier protein that exports citrate from and imports oxoglutarate into the mitochondrion, causing net export of NADPH reducing equivalents; also associates with mt nucleoids and has a role in replication and segregation of the mt genome
CAGL0C04983g	*CgADO1*	-1,28	6,84E-03	*ADO1*	Adenosine kinase, required for the utilization of S-adenosylmethionine (AdoMet); may be involved in recycling adenosine produced through the methyl cycle
CAGL0H05511g		-1,28	1,69E-03	*RPS9A*	Protein component of the small (40S) ribosomal subunit; nearly identical to Rps9Bp and has similarity to E. coli S4 and rat S9 ribosomal proteins
CAGL0L08114g		-1,29	1,31E-03	*RPS22A*	Protein component of the small (40S) ribosomal subunit; nearly identical to Rps22Bp and has similarity to E. coli S8 and rat S15a ribosomal proteins
CAGL0E02013g		-1,30	6,37E-03	*RPL18A*	Protein component of the large (60S) ribosomal subunit, identical to Rpl18Bp and has similarity to rat L18 ribosomal protein; intron of RPL18A pre-mRNA forms stem-loop structures that are a target for Rnt1p cleavage leading to degradation
CAGL0M02497g		-1,31	1,97E-03	*RPL33A*	N-terminally acetylated ribosomal protein L37 of the large (60S) ribosomal subunit, nearly identical to Rpl33Bp and has similarity to rat L35a; rpl33a null mutant exhibits slow growth while rpl33a rpl33b double null mutant is inviable
CAGL0K06281g		-1,32	6,33E-04	*GUK1*	Guanylate kinase, converts GMP to GDP; required for growth and mannose outer chain elongation of cell wall N-linked glycoproteins
CAGL0L01551g		-1,34	2,18E-03	*SUR7*	Plasma membrane protein that localizes to furrow-like invaginations (MCC patches); component of eisosomes; associated with endocytosis, along with Pil1p and Lsp1p; sporulation and plasma membrane sphingolipid content are altered in mutants
CAGL0L08184g	*CgFEN1*	-1,34	1,85E-03	*FEN1*	Fatty acid elongase, involved in sphingolipid biosynthesis; acts on fatty acids of up to 24 carbons in length; mutations have regulatory effects on 1,3-beta-glucan synthase, vacuolar ATPase, and the secretory pathway
CAGL0G07227g		-1,34	2,48E-03	*RPS18B*	Protein component of the small (40S) ribosomal subunit; nearly identical to Rps18Ap and has similarity to E. coli S13 and rat S18 ribosomal proteins
CAGL0L09581g		-1,37	2,54E-03	*DUT1*	deoxyuridine triphosphate diphosphatase (dUTPase); catalyzes hydrolysis of dUTP to dUMP and PPi, thereby preventing incorporation of uracil into DNA during replication; critical for the maintenance of genetic stability; also has diphosphatase activity on deoxyinosine triphosphate
CAGL0G03575g		-1,37	4,55E-03	*RPL23B*	Protein component of the large (60S) ribosomal subunit, identical to Rpl23Ap and has similarity to E. coli L14 and rat L23 ribosomal proteins
CAGL0I04004g		-1,37	3,13E-03	*YDL183C*	Mitochondrial inner-membrane protein thought to be involved in the formation of an active mitochondrial K+/H+ exchanger (KHE) system; non-essential gene
CAGL0J03234g		-1,39	2,85E-03	*RPS24A*	Protein component of the small (40S) ribosomal subunit; identical to Rps24Bp and has similarity to rat S24 ribosomal protein
CAGL0J06374g		-1,43	1,67E-03	*RPP1B*	Ribosomal protein P1 beta, component of the ribosomal stalk, which is involved in interaction of translational elongation factors with ribosome; accumulation is regulated by phosphorylation and interaction with the P2 stalk component
CAGL0D01606g		-1,44	2,29E-03	*YMC1*	Mitochondrial protein, putative inner membrane transporter with a role in oleate metabolism and glutamate biosynthesis; member of the mitochondrial carrier (MCF) family; has similarity with Ymc2p
CAGL0B02794g		-1,45	1,21E-03	*ADE13*	Adenylosuccinate lyase, catalyzes two steps in the 'de novo' purine nucleotide biosynthetic pathway; expression is repressed by adenine and activated by Bas1p and Pho2p; mutations in human ortholog ADSL cause adenylosuccinase deficiency
CAGL0L12364g		-1,47	1,68E-03	*ERG10*	Acetyl-CoA C-acetyltransferase (acetoacetyl-CoA thiolase), cytosolic enzyme that transfers an acetyl group from one acetyl-CoA molecule to another, forming acetoacetyl-CoA; involved in the first step in mevalonate biosynthesis
CAGL0K06061g		-1,48	6,47E-04	*RPS28B*	Protein component of the small (40S) ribosomal subunit; nearly identical to Rps28Ap and has similarity to rat S28 ribosomal protein
CAGL0D01562g	*CgFCY1*	-1,49	1,30E-03	*FCY1*	Cytosine deaminase, zinc metalloenzyme that catalyzes the hydrolytic deamination of cytosine to uracil; of biomedical interest because it also catalyzes the deamination of 5-fluorocytosine (5FC) to form anticancer drug 5-fluorouracil (5FU)
CAGL0A00627g		-1,53	7,63E-04	*ERP6*	Protein with similarity to Emp24p and Erv25p, member of the p24 family involved in ER to Golgi transport; the authentic, non-tagged protein is detected in highly purified mitochondria in high-throughput studies
CAGL0B01122g	*CgSAM1*	-1,54	1,02E-03	*SAM1*	S-adenosylmethionine synthetase, catalyzes transfer of the adenosyl group of ATP to the sulfur atom of methionine; one of two differentially regulated isozymes (Sam1p and Sam2p)
CAGL0D01716g		-1,63	1,36E-03	*POL30*	Proliferating cell nuclear antigen (PCNA), functions as the sliding clamp for DNA polymerase delta; may function as a docking site for other proteins required for mitotic and meiotic chromosomal DNA replication and for DNA repair
CAGL0K06259g	*CgTSA2*	-1,96	1,34E-02	*TSA2*	Stress inducible cytoplasmic thioredoxin peroxidase; cooperates with Tsa1p in the removal of reactive oxygen, nitrogen and sulfur species using thioredoxin as hydrogen donor; deletion enhances the mutator phenotype of tsa1 mutants
CAGL0H05137g		-2,62	4,01E-05	*ALD6*	Cytosolic aldehyde dehydrogenase, activated by Mg2+ and utilizes NADP+ as the preferred coenzyme; required for conversion of acetaldehyde to acetate; constitutively expressed; locates to the mitochondrial outer surface upon oxidative stress
