# Supplementary material for: The CgHaa1-Regulon Mediates Response and Tolerance to Acetic Acid Stress in the Human Pathogen Candida glabrata
Source: G3 (Bethesda). 2016 Nov 4;7(1):1–18. doi: 10.1534/g3.116.034660 (PMC5217100; doi:10.1534/g3.116.034660)
Supplement: Supplementary file 7 [file 1TableS2.pdf]

Supplementary Table S2 – List of the genes found to be up- or down- regulated in response to acetic acid (30 mM, at pH 4.0) in *C. glabrata* KUE100_ΔCgHaa1 cells. Genes whose expression increased or decreased (above a 2-fold threshold level) in acetic acid-challenged cells, in comparison with unstressed cells, were selected and are here listed. Only genes exhibiting similar degree of variance in the three replica samples performed were considered to be differently expressed in response to acetic acid, as detailed in materials and methods. The biological function indicated is based on the information available at Candida Genome Database.

UP-REGULATED GENES

ORF	Gene name	log_2FC (Δ*Cghaa1* AC/ Δ*Cghaa1* CTRL)	p_value	*S. cerevisiae* orthologue	Function
CAGL0M11902g		4,75	3,43E-06	*FUN19*	Non-essential protein of unknown function; expression induced in response to heat stress
CAGL0J00451g		4,38	6,70E-03	-	Putative glyceraldehyde-3-phosphate dehydrogenase
CAGL0M01166g		4,20	1,48E-03	*THI4*	Thiazole synthase, abundant protein involved in the formation of the thiazole moiety of thiamine during thiamine biosynthesis; acts more as a co-substrate rather than an enzyme by providing the sulphur source for thiazole formation; undergoes a single turnover only; required for mitochondrial genome stability in response to DNA damaging agents
CAGL0E01353g		4,09	4,36E-06	*ZRT2*	Low-affinity zinc transporter of the plasma membrane; transcription is induced under low-zinc conditions by the Zap1p transcription factor
CAGL0G03883g		4,05	3,78E-07	*HSP104*	Disaggregase; Heat shock protein that cooperates with Ydj1p (Hsp40) and Ssa1p (Hsp70) to refold and reactivate previously denatured, aggregated proteins; responsive to stresses including: heat, ethanol, and sodium arsenite; involved in [PSI+] propagation
CAGL0I10010g		3,66	4,76E-03	*BTN2*	v-SNARE binding protein that facilitates specific protein retrieval from a late endosome to the Golgi; modulates arginine uptake, possible role in mediating pH homeostasis between the vacuole and plasma membrane H(+)-ATPase
CAGL0B00616g		3,58	3,55E-04	*SPS22*	Protein of unknown function, redundant with Sps2p for the organization of the beta-glucan layer of the spore wall
CAGL0M08822g	*CgHSP78*	3,34	3,89E-05	*HSP78*	Oligomeric mitochondrial matrix chaperone that cooperates with Ssc1p in mitochondrial thermotolerance after heat shock; able to prevent the aggregation of misfolded proteins as well as resolubilize protein aggregates
CAGL0M11660g		3,06	5,40E-04	-	Has domain(s) with predicted hydrolase activity
CAGL0K03685g		3,00	3,82E-05	*PKR1*	V-ATPase assembly factor, functions with other V-ATPase assembly factors in the ER to efficiently assemble the V-ATPase membrane sector (V0)
CAGL0H08151g		2,99	2,20E-05	-	Unknown
CAGL0H08173g		2,94	6,67E-05	-	Unknown
CAGL0E00803g		2,88	1,12E-04	*HSP42*	Small heat shock protein (sHSP) with chaperone activity; forms barrel-shaped oligomers that suppress unfolded protein aggregation; involved in cytoskeleton reorganization after heat shock
CAGL0H00704g		2,86	2,73E-04	*ICY2*	Protein of unknown function; mobilized into polysomes upon a shift from a fermentable to nonfermentable carbon source; potential Cdc28p substrate
CAGL0D01276g		2,77	7,29E-03	*OPY2*	Integral membrane protein that functions in the signaling branch of the high-osmolarity glycerol (HOG) pathway; interacts with Ste50p; overproduction blocks cell cycle arrest in the presence of mating pheromone
CAGL0H04279g	*MT-IIB*	2,73	8,15E-06	-	Copper-binding metallothionein
CAGL0H08844g		2,71	1,30E-05	*DDR48*	DNA damage-responsive protein, expression is increased in response to heat-shock stress or treatments that produce DNA lesions; contains multiple repeats of the amino acid sequence NNNDSYGS
CAGL0K12254g		2,71	1,10E-04	*VID24*	Peripheral membrane protein located at Vid (vacuole import and degradation) vesicles; regulates fructose-1,6-bisphosphatase (FBPase) targeting to the vacuole; promotes proteasome-dependent catabolite degradation of FBPase
CAGL0J04202g	*CgHSP12*	2,70	1,65E-03	*HSP12*	Plasma membrane protein involved in maintaining membrane organization in stress conditions; induced by heat shock, oxidative stress, osmostress, stationary phase, glucose depletion, oleate and alcohol; regulated by HOG and Ras-Pka pathways
CAGL0G08624g	*CgQDR2*	2,69	1,08E-05	*QDR1*	Multidrug transporter of the major facilitator superfamily, required for resistance to quinidine, ketoconazole, fluconazole, and barban
CAGL0E06358g	*CgGPM1*	2,58	3,23E-06	*GPM1*	Tetrameric phosphoglycerate mutase, mediates the conversion of 3-phosphoglycerate to 2-phosphoglycerate during glycolysis and the reverse reaction during gluconeogenesis
CAGL0H03707g		2,57	5,12E-05	*SIS1*	Type II HSP40 co-chaperone that interacts with the HSP70 protein Ssa1p; not functionally redundant with Ydj1p due to due to substrate specificity; shares similarity with bacterial DnaJ proteins
CAGL0K08338g		2,55	3,42E-05	-	Unknown
CAGL0F08261g		2,55	9,61E-04	*ENO1*	Enolase I, a phosphopyruvate hydratase that catalyzes the conversion of 2-phosphoglycerate to phosphoenolpyruvate during glycolysis and the reverse reaction during gluconeogenesis; expression is repressed in response to glucose
CAGL0J01595g	*CgGLM6*	2,55	9,50E-05	*YPR015C*	Putative protein of unknown function; overexpression causes a cell cycle delay or arrest
CAGL0K10164g		2,54	1,24E-05	*SED1*	Major stress-induced structural GPI-cell wall glycoprotein in stationary-phase cells, associates with translating ribosomes, possible role in mitochondrial genome maintenance; ORF contains two distinct variable minisatellites
CAGL0M12320g		2,53	2,66E-04	*FLC2*	Putative FAD transporter; required for uptake of FAD into endoplasmic reticulum; involved in cell wall maintenance
CAGL0J07568g		2,50	5,11E-02	*LAP3*	Cysteine aminopeptidase with homocysteine-thiolactonase activity; protects cells against homocysteine toxicity; has bleomycin hydrolase activity in vitro; transcription is regulated by galactose via Gal4p; orthologous to human BLMH
CAGL0L06446g		2,48	2,30E-04	*YDR132C*	Putative protein of unknown function
CAGL0M07920g	*CgPDC*	2,46	1,99E-05	*PDC1*	Major of three pyruvate decarboxylase isozymes, key enzyme in alcoholic fermentation, decarboxylates pyruvate to acetaldehyde; subject to glucose-, ethanol-, and autoregulation; involved in amino acid catabolism
CAGL0D04752g		2,46	4,55E-05	*YPR127W*	Protein of unknown function, differentially expressed during alcoholic fermentation; expression activated by transcription factor YRM1/YOR172W; green fluorescent protein (GFP)-fusion protein localizes to both the cytoplasm and the nucleus
CAGL0G09383g	*CgTDH3*	2,42	5,67E-05	*TDH3*	Glyceraldehyde-3-phosphate dehydrogenase, isozyme 3, involved in glycolysis and gluconeogenesis; tetramer that catalyzes the reaction of glyceraldehyde-3-phosphate to 1,3 bis-phosphoglycerate; detected in the cytoplasm and cell wall
CAGL0D01270g		2,41	1,93E-05	-	Unknown
CAGL0I05874g		2,39	1,89E-05	-	Haloacid dehalogenase-like hydrolase

CAGL0F06413g		2,39	4,66E-02	*FET3*	Ferro-O2-oxidoreductase required for high-affinity iron uptake and involved in mediating resistance to copper ion toxicity, belongs to class of integral membrane multicopper oxidases
CAGL0M12430g	*CgRHR2*	2,37	7,17E-04	*RHR2*	Constitutively expressed isoform of DL-glycerol-3-phosphatase; involved in glycerol biosynthesis, induced in response to both anaerobic and, along with the Hor2p/Gpp2p isoform, osmotic stress
CAGL0C02321g	*CgPHM8*	2,36	2,21E-04	*PHM8*	Lysophosphatidic acid (LPA) phosphatase involved in LPA hydrolysis in response to phosphate starvation; phosphatase activity is soluble and Mg2+ dependent; expression is induced by low phosphate levels and by inactivation of Pho85p
CAGL0K11858g		2,34	3,17E-05	*PST2*	Protein with similarity to members of a family of flavodoxin-like proteins; induced by oxidative stress in a Yap1p dependent manner; the authentic, non-tagged protein is detected in highly purified mitochondria in high-throughput studies
CAGL0L07722g	*CgPGK1*	2,34	6,39E-06	*PGK1*	3-phosphoglycerate kinase, catalyzes transfer of high-energy phosphoryl groups from the acyl phosphate of 1,3-bisphosphoglycerate to ADP to produce ATP; key enzyme in glycolysis and gluconeogenesis
CAGL0E04312g		2,28	3,53E-05	*STP2*	Transcription factor, activated by proteolytic processing in response to signals from the SPS sensor system for external amino acids; activates transcription of amino acid permease genes
CAGL0K09702g		2,27	3,05E-03	*YNL134C*	Putative protein of unknown function with similarity to dehydrogenases from other model organisms; green fluorescent protein (GFP)-fusion protein localizes to both the cytoplasm and nucleus and is induced by the DNA-damaging agent MMS
CAGL0L07480g		2,24	9,75E-05	*NRG2*	Transcriptional repressor that mediates glucose repression and negatively regulates filamentous growth; has similarity to Nrg1p
CAGL0J10494g		2,21	4,24E-05	*APT2*	Apparent pseudogene, not transcribed or translated under normal conditions; encodes a protein with similarity to adenine phosphoribosyltransferase, but artificially expressed protein exhibits no enzymatic activity
CAGL0A02046g		2,20	2,00E-02	*ROD1*	Membrane protein that binds the ubiquitin ligase Rsp5p via its 2 PY motifs; overexpression confers resistance to the GST substrate o-dinitrobenzene,zinc, and calcium; proposed to regulate the endocytosis of plasma membrane proteins
CAGL0E01881g	*CgYPS11*	2,19	6,06E-04	-	Putative aspartic protease
CAGL0K10868g	*CgCTA1*	2,18	7,92E-04	*CTA1*	Catalase A, breaks down hydrogen peroxide in the peroxisomal matrix formed by acyl-CoA oxidase (Pox1p) during fatty acid beta-oxidation
CAGL0B03069g	*CgTAL1*	2,17	8,48E-05	*TAL1*	Transaldolase, enzyme in the non-oxidative pentose phosphate pathway; converts sedoheptulose 7-phosphate and glyceraldehyde 3-phosphate to erythrose 4-phosphate and fructose 6-phosphate
CAGL0E01793g	*CgYPS6*	2,14	1,30E-04	-	Putative aspartic protease
CAGL0I02486g	*CgENO1*	2,14	1,44E-05	*ENO2*	Enolase II, a phosphopyruvate hydratase that catalyzes the conversion of 2-phosphoglycerate to phosphoenolpyruvate during glycolysis and the reverse reaction during gluconeogenesis; expression is induced in response to glucose
CAGL0D05082g		2,12	1,05E-04	*UBI4*	Ubiquitin, becomes conjugated to proteins, marking them for selective degradation via the ubiquitin-26S proteasome system; essential for the cellular stress response; encoded as a polyubiquitin precursor comprised of 5 head-to-tail repeats
CAGL0E01837g	*CgYPS9*	2,12	3,49E-05	-	Putative aspartic protease
CAGL0G03289g	*CgSSA3*	2,09	4,69E-03	*SSA4*	Heat shock protein that is highly induced upon stress; plays a role in SRP-dependent cotranslational protein-membrane targeting and translocation; member of the HSP70 family; cytoplasmic protein that concentrates in nuclei upon starvation
CAGL0M04191g	*CgYPS1*	2,07	1,50E-04	*YPS1*	Aspartic protease, member of the yapsin family of proteases involved in cell wall growth and maintenance; attached to the plasma membrane via a glycosylphosphatidylinositol (GPI) anchor
CAGL0C00275g	*CgHSP31*	2,05	7,64E-02	*HSP31*	Putative cysteine protease
CAGL0I09724g		2,05	9,94E-05	-	Has domain(s) with predicted role in transmembrane transport
CAGL0K05813g		2,04	4,86E-03	*GRX2*	Cytoplasmic glutaredoxin, thioltransferase, glutathione-dependent disulfide oxidoreductase involved in maintaining redox state of target proteins, also exhibits glutathione peroxidase activity, expression induced in response to stress
CAGL0I00418g		2,00	6,28E-03	*OLE1*	Delta(9) fatty acid desaturase, required for monounsaturated fatty acid synthesis and for normal distribution of mitochondria
CAGL0K03663g		1,99	1,07E-04	*YMR122W-A*	Protein of unknown function; green fluorescent protein (GFP)-fusion protein localizes to the cytoplasm and endoplasmic reticulum
CAGL0K03707g		1,98	8,31E-04	*YMR124W*	Protein of unknown function; GFP-fusion protein localizes to the cell periphery, cytoplasm, bud, and bud neck; interacts with Crm1p in two-hybrid assay; YMR124W is not an essential gene; predicted to have a role in organelle organization
CAGL0B00990g	*CgHBN1*	1,98	1,27E-04	*FRM2*	Type II nitroreductase, using NADH as reductant; mutants are defective in fatty acid mediated repression of genes involved in fatty acid biosynthesis indicative of a role in lipid signaling; involved in the oxidative stress response; transcription induction by cadmium and selenite indicates a possible role in the metal stress response; expression induced in cells treated with the mycotoxin patulin
CAGL0G08866g		1,96	2,22E-03	*FKH1*	Forkhead family transcription factor with a minor role in the expression of G2/M phase genes; negatively regulates transcriptional elongation; positive role in chromatin silencing at HML and HMR; regulates donor preference during switching
CAGL0I05934g		1,95	1,27E-02	*YJL144W*	Cytoplasmic hydrophilin essential in dessication-rehydration process; expression induced by osmotic stress, starvation and during stationary phase; GFP-fusion protein is induced by the DNA-damaging agent MMS
CAGL0H02101g		1,95	7,68E-02	*RTC3*	Protein of unknown function involved in RNA metabolism; has structural similarity to SBDS, the human protein mutated in Shwachman-Diamond Syndrome (the yeast SBDS ortholog = SDO1); null mutation suppresses cdc13-1 temperature sensitivity
CAGL0F08371g	*CgTNA1*	1,94	3,18E-05	*TNA1*	High affinity nicotinic acid plasma membrane permease, responsible for uptake of low levels of nicotinic acid; expression of the gene increases in the absence of extracellular nicotinic acid or para-aminobenzoate (PABA)
CAGL0H02563g		1,93	9,08E-04	*HOR7*	Protein of unknown function; overexpression suppresses Ca2+ sensitivity of mutants lacking inositol phosphorylceramide mannosyltransferases Csg1p and Csh1p; transcription is induced under hyperosmotic stress and repressed by alpha factor
CAGL0E06424g		1,90	2,67E-03	*MCR1*	Mitochondrial NADH-cytochrome b5 reductase, involved in ergosterol biosynthesis
CAGL0L10362g		1,90	1,14E-04	*YOR062C*	Protein of unknown function; similar to YKR075Cp and Reg1p; expression regulated by glucose and Rgt1p; GFP-fusion protein is induced in response to the DNA-damaging agent MMS
CAGL0G01540g	*CgNCE103*	1,89	1,72E-03	*NCE103*	Carbonic anhydrase; metalloenzyme that catalyzes CO2 hydration to bicarbonate, which is an important metabolic substrate, and protons; not expressed under conditions of high CO2, such as inside a growing colony, but transcription is induced in response to low CO2 levels, such as on the colony surface in ambient air; poorly transcribed under aerobic conditions and at an undetectable level under anaerobic conditions;
CAGL0G04763g		1,89	7,03E-03	*RGS2*	Negative regulator of glucose-induced cAMP signaling; directly activates the GTPase activity of the heterotrimeric G protein alpha subunit Gpa2p
CAGL0D06402g	*CgMET15*	1,89	1,70E-04	*MET17*	Methionine and cysteine synthase (O-acetyl homoserine-O-acetyl serine sulfhydrylase), required for sulfur amino acid synthesis
CAGL0F01111g		1,88	3,69E-03	*OPI10*	Protein with a possible role in phospholipid biosynthesis, based on inositol-excreting phenotype of the null mutant and its suppression by exogenous choline
CAGL0H02959g	*CgTOS8*	1,88	4,26E-04	*TOS8*	Homeodomain-containing protein and putative transcription factor found associated with chromatin; target of SBF transcription factor; induced during meiosis and under cell-damaging conditions; similar to Cup9p transcription factor
CAGL0I01122g	*CgGRE3*	1,86	8,57E-05	*GRE3*	Aldose reductase involved in methylglyoxal, d-xylose, arabinose, and galactose metabolism; stress induced (osmotic, ionic, oxidative, heat shock, starvation and heavy metals); regulated by the HOG pathway
CAGL0J11242g		1,85	3,82E-03	*RHO5*	Non-essential small GTPase of the Rho/Rac subfamily of Ras-like proteins, likely involved in protein kinase C (Pkc1p)-dependent signal transduction pathway that controls cell integrity
CAGL0G03795g	*CgSSA1*	1,84	6,21E-04	*SSA2*	ATP binding protein involved in protein folding and vacuolar import of proteins; member of heat shock protein 70 (HSP70) family; associated with the chaperonin-containing T-complex; present in the cytoplasm, vacuolar membrane and cell wall; 98% identical with Ssa1p, but subtle differences between the two proteins provide functional specificity with respect to propagation of yeast [URE3] prions and vacuolar-mediated

CAGL0H08327g	*CgTPI1*	1,84	4,80E-05	*TPI1*	Triose phosphate isomerase, abundant glycolytic enzyme; mRNA half-life is regulated by iron availability; transcription is controlled by activators Reb1p, Gcr1p, and Rap1p through binding sites in the 5' non-coding region; inhibition of Tpi1p activity by PEP (phosphoenolpyruvate) stimulates redox metabolism in respiring cells; E104D mutation in human TPI causes a rare autosomal disease
CAGL0F04631g		1,84	2,30E-04	*MOH1*	Protein of unknown function, has homology to kinase Snf7p; not required for growth on nonfermentable carbon sources; essential for survival in stationary phase
CAGL0M02167g		1,83	5,99E-03	*PRM4*	Pheromone-regulated protein proposed to be involved in mating; predicted to have 1 transmembrane segment; transcriptionally regulated by Ste12p during mating and by Cat8p during the diauxic shift
CAGL0H03971g		1,82	1,13E-03	*YCP4*	Protein of unknown function, has sequence and structural similarity to flavodoxins; predicted to be palmitoylated; the authentic, non-tagged protein is detected in highly purified mitochondria in high-throughput studies
CAGL0K00803g	*CgTRX2*	1,82	2,75E-04	*TRX1*	Cytoplasmic thioredoxin isoenzyme of the thioredoxin system which protects cells against oxidative and reductive stress, forms LMA1 complex with Pbi2p, acts as a cofactor for Tsa1p, required for ER-Golgi transport and vacuole inheritance
CAGL0G07271g		1,80	7,45E-04	*TSA1*	Thioredoxin peroxidase, acts as both a ribosome-associated and free cytoplasmic antioxidant; self-associates to form a high-molecular weight chaperone complex under oxidative stress; deletion results in mutator phenotype
CAGL0K04169g		1,80	9,45E-03	*KSS1*	Mitogen-activated protein kinase (MAPK) involved in signal transduction pathways that control filamentous growth and pheromone response; the KSS1 gene is nonfunctional in S288C strains and functional in W303 strains
CAGL0B03619g		1,80	6,01E-03	*PRB1*	Vacuolar proteinase B (yscB), a serine protease of the subtilisin family; involved in protein degradation in the vacuole and required for full protein degradation during sporulation; activity inhibited by Pbi2p
CAGL0F07579g	*CgCWP1_2*	1,80	7,84E-05	*CWP1*	Cell wall mannoprotein that localizes specifically to birth scars of daughter cells, linked to a beta-1,3- and beta-1,6-glucan heteropolymer through a phosphodiester bond; required for propionic acid resistance
CAGL0M13343g	*CgGND1*	1,79	1,04E-03	*GND1*	6-phosphogluconate dehydrogenase (decarboxylating), catalyzes an NADPH regenerating reaction in the pentose phosphate pathway; required for growth on D-glucono-delta-lactone and adaptation to oxidative stress
CAGL0F07601g	*CgCWP1_1*	1,78	1,64E-04	*CWP2*	Covalently linked cell wall mannoprotein, major constituent of the cell wall; plays a role in stabilizing the cell wall; involved in low pH resistance; precursor is GPI-anchored
CAGL0I05390g		1,78	1,32E-03	*SKS1*	Putative serine/threonine protein kinase; involved in the adaptation to low concentrations of glucose independent of the SNF3 regulated pathway
CAGL0G09449g	*CgCRH1*	1,77	4,72E-05	*CRH1*	Chitin transglycosylase that functions in the transfer of chitin to beta(1-6) and beta(1-3) glucans in the cell wall; similar and functionally redundant to Utr2; localizes to sites of polarized growth; expression induced by cell wall stress
CAGL0M07315g		1,77	1,45E-02	*SUR1*	Probable catalytic subunit of a mannosylinositol phosphorylceramide (MIPC) synthase, forms a complex with probable regulatory subunit Csg2p; function in sphingolipid biosynthesis is overlapping with that of Csh1p
CAGL0I06182g	*CgPIR2*	1,76	7,80E-05	*HSP150*	O-mannosylated heat shock protein that is secreted and covalently attached to the cell wall via beta-1,3-glucan and disulfide bridges; required for cell wall stability; induced by heat shock, oxidative stress, and nitrogen limitation
CAGL0B03685g		1,75	2,64E-03	*YCP4*	Protein of unknown function, has sequence and structural similarity to flavodoxins; predicted to be palmitoylated; the authentic, non-tagged protein is detected in highly purified mitochondria in high-throughput studies
CAGL0M12034g	*CgPYK1*	1,75	1,68E-04	*CDC19*	Pyruvate kinase, functions as a homotetramer in glycolysis to convert phosphoenolpyruvate to pyruvate, the input for aerobic (TCA cycle) or anaerobic (glucose fermentation) respiration
CAGL0L09383g		1,75	3,89E-04	*SUT2*	Putative transcription factor; multicopy suppressor of mutations that cause low activity of the cAMP/protein kinase A pathway; highly similar to Sut1p
CAGL0E05654g		1,74	3,96E-03	*PGC1*	Phosphatidyl Glycerol phospholipase C; regulates the phosphatidylglycerol (PG) content via a phospholipase C-type degradation mechanism; contains glycerophosphodiester phosphodiesterase motifs
CAGL0J10846g		1,74	3,43E-05	*PCL5*	Cyclin, interacts with and phosphorylated by Pho85p cyclin-dependent kinase (Cdk), induced by Gcn4p at level of transcription, specifically required for Gcn4p degradation, may be sensor of cellular protein biosynthetic capacity
CAGL0I01100g	*CgGCY1*	1,74	2,85E-03	*GCY1*	Putative NADP(+) coupled glycerol dehydrogenase, proposed to be involved in an alternative pathway for glycerol catabolism; also has mRNA binding activity; member of the aldo-keto reductase (AKR) family
CAGL0A01221g		1,74	8,26E-05	-	Has domain(s) with predicted transporter activity
CAGL0M13189g	*CgMSN4*	1,71	6,54E-04	*MSN4*	Transcriptional activator related to Msn2p; activated in stress conditions, which results in translocation from the cytoplasm to the nucleus; binds DNA at stress response elements of responsive genes, inducing gene expression
CAGL0F08745g		1,70	2,00E-03	*STF2*	Protein involved in resistance to dessication stress; Stf2p exhibits antioxidant properties, and its overexpression prevents ROS accumulation and apoptosis; binds to the F0 sector of mitochondrial F1F0 ATPase in vitro and is proposed to modulate the inhibitory action of Inh1p and Stf1p
CAGL0J07612g	*CgZWF1*	1,70	1,01E-04	*ZWF1*	Glucose-6-phosphate dehydrogenase (G6PD), catalyzes the first step of the pentose phosphate pathway; involved in adapting to oxidatve stress; homolog of the human G6PD which is deficient in patients with hemolytic anemia
CAGL0E05170g	*CgGRE2*	1,70	3,52E-04	-	Putative methylglyoxal reductase (NADPH-dependent)
CAGL0B03839g	*CgMET3*	1,69	7,22E-04	*MET3*	ATP sulfurylase, catalyzes the primary step of intracellular sulfate activation, essential for assimilatory reduction of sulfate to sulfide, involved in methionine metabolism
CAGL0F06369g		1,68	1,92E-03	*LHS1*	Molecular chaperone of the endoplasmic reticulum lumen, involved in polypeptide translocation and folding; nucleotide exchange factor for the ER lumenal Hsp70 chaperone Kar2p; regulated by the unfolded protein response pathway
CAGL0J04466g		1,68	5,83E-03	*PUN1*	Plasma membrane protein with a role in cell wall integrity; co-localizes with Sur7p in punctate membrane patches; null mutant displays decreased thermotolerance; transcription induced upon cell wall damage and metal ion stress
CAGL0M07634g		1,66	5,76E-05	*SOK2*	Nuclear protein that plays a regulatory role in the cyclic AMP (cAMP)-dependent protein kinase (PKA) signal transduction pathway; negatively regulates pseudohyphal differentiation; homologous to several transcription factors
CAGL0M08492g	*CgPIR3*	1,66	5,48E-05	*PIR1*	O-glycosylated protein required for cell wall stability; attached to the cell wall via beta-1,3-glucan; mediates mitochondrial translocation of Apn1p; expression regulated by the cell integrity pathway and by Swi5p during the cell cycle
CAGL0A03102g	*CgARO10*	1,66	8,80E-03	*ARO10*	Phenylpyruvate decarboxylase, catalyzes decarboxylation of phenylpyruvate to phenylacetaldehyde, which is the first specific step in the Ehrlich pathway
CAGL0E04356g		1,65	4,87E-04	*SOD2*	Mitochondrial manganese superoxide dismutase, protects cells against oxygen toxicity; phosphorylated
CAGL0M13651g		1,62	3,40E-02	*PRC1*	Vacuolar carboxypeptidase Y (proteinase C; CPY), broad-specificity C-terminal exopeptidase involved in non-specific protein degradation in the vacuole; member of the serine carboxypeptidase family
CAGL0E01177g	*CgCPR1*	1,62	3,07E-04	*CPR1*	Cytoplasmic peptidyl-prolyl cis-trans isomerase (cyclophilin), catalyzes the cis-trans isomerization of peptide bonds N-terminal to proline residues; binds the drug cyclosporin A
CAGL0G06006g		1,62	4,76E-04	*YHR138C*	Putative protein of unknown function; has similarity to Pbi2p; double null mutant lacking Pbi2p and Yhr138p exhibits highly fragmented vacuoles
CAGL0L00495g	*CgHSC82*	1,61	7,62E-04	*HSC82*	Cytoplasmic chaperone of the Hsp90 family, redundant in function and nearly identical with Hsp82p, and together they are essential; expressed constitutively at 10-fold higher basal levels than HSP82 and induced 2-3 fold by heat shock
CAGL0L02497g	*CgFBA1*	1,59	1,33E-04	*FBA1*	Fructose 1,6-bisphosphate aldolase, required for glycolysis and gluconeogenesis; catalyzes conversion of fructose 1,6 bisphosphate to glyceraldehyde-3-P and dihydroxyacetone-P; locates to mitochondrial outer surface upon oxidative stress
CAGL0M12386g		1,59	4,80E-03	*MMF1*	Mitochondrial protein required for transamination of isoleucine but not of valine or leucine; may regulate specificity of branched-chain transaminases Bat1p and Bat2p; interacts genetically with mitochondrial ribosomal protein genes
CAGL0D01298g	*CgTKL1*	1,59	4,10E-03	*TKL1*	Transketolase, similar to Tkl2p; catalyzes conversion of xylulose-5-phosphate and ribose-5-phosphate to sedoheptulose-7-phosphate and glyceraldehyde-3-phosphate in the pentose phosphate pathway; needed for synthesis of aromatic amino acids
CAGL0L06974g		1,57	1,08E-03	*YDL086W*	Putative protein of unknown function; the authentic, non-tagged protein is detected in highly purified mitochondria in high-throughput studies; YDL086W is not an essential gene

CAGL0L06072g		1,56	3,02E-04	*YER130C*	Protein of unknown function; transcription is regulated by Haa1p, Sok2p and Zap1p transcriptional activators; computational analysis suggests a role as a transcription factor; C. albicans homolog (MNL1) plays a role in adaptation to stress
CAGL0D05280g		1,56	1,56E-03	*MET10*	Subunit alpha of assimilatory sulfite reductase, which converts sulfite into sulfide
CAGL0B00704g	*CgPDI1*	1,56	1,47E-04	*PDI1*	Protein disulfide isomerase; multifunctional protein resident in the endoplasmic reticulum lumen, essential for the formation of disulfide bonds in secretory and cell-surface proteins, unscrambles non-native disulfide bonds; forms a complex with Mnl1p that has exomannosidase activity, processing unfolded protein-bound Man8GlcNAc2 oligosaccharides to Man7GlcNAc2 which promotes degradation in the unfolded protein
CAGL0M05995g		1,55	4,65E-04	*PET10*	Protein of unknown function that co-purifies with lipid particles; expression pattern suggests a role in respiratory growth; computational analysis of large-scale protein-protein interaction data suggests a role in ATP/ADP exchange
CAGL0M00374g		1,55	3,61E-04	*MET5*	Sulfite reductase beta subunit, involved in amino acid biosynthesis, transcription repressed by methionine
CAGL0F07557g		1,55	3,69E-03	*YJU2*	Essential protein required for pre-mRNA splicing; associates transiently with the spliceosomal NTC (nineteen complex) and acts after Prp2p to promote the first catalytic reaction of splicing
CAGL0H08393g		1,55	1,16E-03	*BAP2*	High-affinity leucine permease, functions as a branched-chain amino acid permease involved in the uptake of leucine, isoleucine and valine; contains 12 predicted transmembrane domains
CAGL0I07821g		1,52	3,27E-04	*DUF1*	Ubiquitin-binding protein of unknown function; contains one WD40 repeat in a beta-propeller fold; green fluorescent protein (GFP)-fusion protein localizes to the cytoplasm; homolog of human WDR48/UAF1, which is involved in regulating the Fanconi anemia pathway; deletion mutant is sensitive to various chemicals including phenanthroline, sanguinarine, and nordihydroguaiaretic acid
CAGL0I06743g		1,52	1,36E-04	*FTR1*	High affinity iron permease involved in the transport of iron across the plasma membrane; forms complex with Fet3p; expression is regulated by iron
CAGL0L10186g		1,51	6,61E-02	*YOR052C*	Nuclear protein of unknown function; expression induced by nitrogen limitation in a GLN3, GAT1-independent manner and by weak acid; transcriptionally regulated by Rpn4p along with proteasome subunit genes; putative ortholog of human AIRAP, which stimulates proteasome activity in response to arsenic
CAGL0C02541g		1,51	9,38E-04	*BDF1*	Protein involved in transcription initiation at TATA-containing promoters; associates with the basal transcription factor TFIID; contains two bromodomains; corresponds to the C-terminal region of mammalian TAF1; redundant with Bdf2p
CAGL0C04785g		1,51	1,56E-03	*YJR115W*	Putative protein of unknown function
CAGL0I10384g	*CgTPO3*	1,49	3,53E-04	*TPO2*	Polyamine transport protein specific for spermine; localizes to the plasma membrane; transcription of TPO2 is regulated by Haa1p; member of the major facilitator superfamily
CAGL0J01870g		1,48	1,06E-03	*PMR1*	High affinity Ca2+/Mn2+ P-type ATPase required for Ca2+ and Mn2+ transport into Golgi; involved in Ca2+ dependent protein sorting and processing; mutations in human homolog ATP2C1 cause acantholytic skin condition Hailey-Hailey disease
CAGL0K01727g	*CgRPN4*	1,47	7,14E-04	*RPN4*	Transcription factor that stimulates expression of proteasome genes; Rpn4p levels are in turn regulated by the 26S proteasome in a negative feedback control mechanism; RPN4 is transcriptionally regulated by various stress responses
CAGL0F04807g		1,47	2,66E-03	*OM45*	Protein of unknown function, major constituent of the mitochondrial outer membrane; located on the outer (cytosolic) face of the outer membrane
CAGL0D05434g		1,45	1,08E-03	-	Has domain(s) with predicted sequence-specific DNA binding activity
CAGL0K04301g		1,45	9,69E-04	*FMP48*	Putative protein of unknown function; the authentic, non-tagged protein is detected in highly purified mitochondria in high-throughput studies; induced by treatment with 8-methoxypsoralen and UVA irradiation
CAGL0F04895g		1,45	7,85E-03	*GPH1*	Non-essential glycogen phosphorylase required for the mobilization of glycogen, activity is regulated by cyclic AMP-mediated phosphorylation, expression is regulated by stress-response elements and by the HOG MAP kinase pathway
CAGL0K04125g		1,45	2,48E-04	*ACB1*	Acyl-CoA-binding protein, transports newly synthesized acyl-CoA esters from fatty acid synthetase (Fas1p-Fas2p) to acyl-CoA-consuming processes; subject to starvation-induced, Grh1p-mediated unconventional secretion
CAGL0J11968g	*CgEPA15*	1,44	2,57E-04	*FLO5*	Putative adhesin-like cell wall protein
CAGL0K00715g	*CgRTA1*	1,44	2,07E-04	*YLR046C*	Putative membrane protein; member of the fungal lipid-translocating exporter (LTE) family of proteins; transcription is activated by paralogous transcription factors Yrm1p and Yrr1p along with genes involved in multidrug resistance
CAGL0H09130g		1,43	7,21E-04	*MNN4*	Putative positive regulator of mannosylphosphate transferase (Mnn6p), involved in mannosylphosphorylation of N-linked oligosaccharides; expression increases in late-logarithmic and stationary growth phases
CAGL0J09900g		1,43	4,22E-04	*POR1*	Mitochondrial porin (voltage-dependent anion channel), outer membrane protein required for the maintenance of mitochondrial osmotic stability and mitochondrial membrane permeability; phosphorylated
CAGL0I02200g	*CgSOL3*	1,42	3,94E-03	*SOL3*	6-phosphogluconolactonase, catalyzes the second step of the pentose phosphate pathway; weak multicopy suppressor of los1-1 mutation; homologous to Sol2p and Sol1p
CAGL0D06424g	*CgACO1*	1,42	9,34E-04	*ACO1*	Aconitase, required for the tricarboxylic acid (TCA) cycle and also independently required for mitochondrial genome maintenance; phosphorylated; component of the mitochondrial nucleoid; mutation leads to glutamate auxotrophy
CAGL0G05632g		1,42	4,21E-03	*YDL218W*	Putative protein of unknown function; YDL218W transcription is regulated by Azf1p and induced by starvation and aerobic conditions; expression also induced in cells treated with the mycotoxin patulin
CAGL0L06424g		1,42	6,33E-03	*YDR134C*	ANNOTATED BY YGOB -
CAGL0M02211g	*CgPEP4*	1,41	3,34E-03	*PEP4*	Vacuolar aspartyl protease (proteinase A), required for the posttranslational precursor maturation of vacuolar proteinases; important for protein turnover after oxidative damage; synthesized as a zymogen, self-activates
CAGL0H09856g		1,41	1,14E-02	*HHT1*	Histone H3, core histone protein required for chromatin assembly, part of heterochromatin-mediated telomeric and HM silencing; one of two identical histone H3 proteins (see HHT2); regulated by acetylation, methylation, and phosphorylation
CAGL0J06050g		1,41	1,70E-04	*YGP1*	Cell wall-related secretory glycoprotein; induced by nutrient deprivation-associated growth arrest and upon entry into stationary phase; may be involved in adaptation prior to stationary phase entry; has similarity to Sps100p
CAGL0I02508g		1,41	8,21E-03	*CTR2*	Putative low-affinity copper transporter of the vacuolar membrane; mutation confers resistance to toxic copper concentrations, while overexpression confers resistance to copper starvation
CAGL0A03212g		1,41	4,98E-04	*ATO3*	Plasma membrane protein, regulation pattern suggests a possible role in export of ammonia from the cell; phosphorylated in mitochondria; member of the TC 9.B.33 YaaH family of putative transporters
CAGL0E05566g		1,41	2,19E-03	*TYE7*	Serine-rich protein that contains a basic-helix-loop-helix (bHLH) DNA binding motif; binds E-boxes of glycolytic genes and contributes to their activation; may function as a transcriptional activator in Ty1-mediated gene expression
CAGL0K09130g		1,41	8,28E-03	*SRL1*	Mannoprotein that exhibits a tight association with the cell wall, required for cell wall stability in the absence of GPI-anchored mannoproteins; has a high serine-threonine content; expression is induced in cell wall mutants
CAGL0J10296g		1,40	5,22E-02	*APJ1*	Putative chaperone of the HSP40 (DNAJ) family; overexpression interferes with propagation of the [Psi+] prion; the authentic, non-tagged protein is detected in highly purified mitochondria in high-throughput studies
CAGL0J03960g		1,40	2,78E-04	*WTM2*	Transcriptional modulator involved in regulation of meiosis, silencing, and expression of RNR genes; involved in response to replication stress; contains WD repeats
CAGL0L07920g		1,40	8,14E-03	*PET18*	Protein of unknown function, has weak similarity to proteins involved in thiamin metabolism; expression is induced in the absence of thiamin
CAGL0E05456g		1,40	6,21E-04	*YOR338W*	Putative protein of unknown function; YOR338W transcription is regulated by Azf1p and its transcript is a specific target of the G protein effector Scp160p; identified as being required for sporulation in a high-throughput mutant screen
CAGL0L02937g	*CgHIS3*	1,38	5,86E-04	*HIS3*	Imidazoleglycerol-phosphate dehydratase, catalyzes the sixth step in histidine biosynthesis; mutations cause histidine auxotrophy and sensitivity to Cu, Co, and Ni salts; transcription is regulated by general amino acid control via Gcn4p
CAGL0J08316g		1,38	9,99E-03	*MET2*	L-homoserine-O-acetyltransferase, catalyzes the conversion of homoserine to O-acetyl homoserine which is the first step of the methionine biosynthetic pathway

CAGL0A02299g		1,38	4,07E-04	-	Unknown
CAGL0H07667g		1,37	5,81E-03	*PDE1*	Low-affinity cyclic AMP phosphodiesterase, controls glucose and intracellular acidification-induced cAMP signaling, target of the cAMP-protein kinase A (PKA) pathway; glucose induces transcription and inhibits translation
CAGL0L01837g	*CgVPS24*	1,37	5,25E-03	*VPS24*	One of four subunits of the endosomal sorting complex required for transport III (ESCRT-III); forms an ESCRT-III subcomplex with Did4p; involved in the sorting of transmembrane proteins into the multivesicular body (MVB) pathway
CAGL0D04092g	*CgDOG2*	1,36	6,89E-03	-	2-deoxyglucose-6-phosphate phosphatase
CAGL0A04081g		1,36	1,77E-03	*YLR194C*	Structural constituent of the cell wall attached to the plasma membrane by a GPI-anchor; expression is upregulated in response to cell wall stress
CAGL0I01078g	*CgPFY1*	1,36	3,79E-04	*PFY1*	Profilin, binds actin, phosphatidylinositol 4,5-bisphosphate, and polyproline regions; involved in cytoskeleton organization; required for normal timing of actin polymerization in response to thermal stress
CAGL0C01793g		1,34	6,75E-03	*OM14*	Integral mitochondrial outer membrane protein; abundance is decreased in cells grown in glucose relative to other carbon sources; appears to contain 3 alpha-helical transmembrane segments; ORF encodes a 97-basepair intron
CAGL0H01375g	*CgSUR2*	1,34	9,84E-04	*SUR2*	Sphinganine C4-hydroxylase, catalyses the conversion of sphinganine to phytosphingosine in sphingolipid biosyntheis
CAGL0M02101g		1,33	5,10E-04	*PET20*	Mitochondrial protein, required for respiratory growth under some conditions and for stability of the mitochondrial genome
CAGL0L08426g	*CgSUE1*	1,32	4,26E-03	*SUE1*	Mitochondrial protein required for degradation of unstable forms of cytochrome c
CAGL0I04554g	*CgGRX7*	1,31	1,18E-03	*GRX7*	Cis-golgi localized monothiol glutaredoxin; more similar in activity to dithiol than other monothiol glutaredoxins; involved in the oxidative stress response; does not bind metal ions; functional overlap with GRX6
CAGL0H09834g		1,31	3,98E-04	*HHF1*	Histone H4, core histone protein required for chromatin assembly and chromosome function; one of two identical histone proteins (see also HHF2); contributes to telomeric silencing; N-terminal domain involved in maintaining genomic integrity
CAGL0C04136g		1,30	5,00E-04	*HHF1*	Histone H4, core histone protein required for chromatin assembly and chromosome function; one of two identical histone proteins (see also HHF2); contributes to telomeric silencing; N-terminal domain involved in maintaining genomic integrity
CAGL0F04125g		1,29	5,57E-04	*YBL029W*	Non-essential protein of unknown function
CAGL0K07007g		1,29	4,55E-03	*YBR238C*	Mitochondrial membrane protein with similarity to Rmd9p; not required for respiratory growth but causes a synthetic respiratory defect in combination with rmd9 mutations; transcriptionally up-regulated by TOR; deletion increases life span
CAGL0L12342g		1,29	4,80E-04	*SUL2*	High affinity sulfate permease; sulfate uptake is mediated by specific sulfate transporters Sul1p and Sul2p, which control the concentration of endogenous activated sulfate intermediates
CAGL0H05445g	*CgPGI1*	1,29	4,81E-04	*PGI1*	Glycolytic enzyme phosphoglucose isomerase, catalyzes the interconversion of glucose-6-phosphate and fructose-6-phosphate; required for cell cycle progression and completion of the gluconeogenic events of sporulation
CAGL0G09977g	*CgGDB1*	1,28	7,11E-03	*GDB1*	Glycogen debranching enzyme containing glucanotranferase and alpha-1,6-amyloglucosidase activities, required for glycogen degradation; phosphorylated in mitochondria; activity is inhibited by Igd1p
CAGL0C04587g		1,28	4,03E-04	*YJR098C*	Putative protein of unknown function; the authentic, non-tagged protein is detected in highly purified mitochondria in high-throughput studies
CAGL0F07359g		1,27	8,90E-04	*YGL117W*	Putative protein of unknown function
CAGL0J01331g		1,27	3,37E-03	-	Unknown
CAGL0C04114g	*CgHHT2*	1,26	2,74E-03	*HHT1*	Histone H3, core histone protein required for chromatin assembly, part of heterochromatin-mediated telomeric and HM silencing; one of two identical histone H3 proteins (see HHT2); regulated by acetylation, methylation, and phosphorylation
CAGL0D02618g	*CgPEX11*	1,26	6,22E-03	*PEX11*	Peroxisomal membrane protein required for medium-chain fatty acid oxidation and peroxisome proliferation, possibly by inducing membrane curvature; localization regulated by phosphorylation; transcription regulated by Adr1p and Pip2p-Oaf1p
CAGL0M00132g	*CgEPA12*	1,26	2,88E-02	*FLO1*	Putative adhesin-like cell wall protein
CAGL0H00418g		1,26	4,22E-03	-	Unknown
CAGL0G05049g		1,24	2,61E-03	*AIM7*	Protein that interacts with Arp2/3 complex to stimulate actin filament debranching and inhibit actin nucleation; has similarity to Cof1p and also to human glia maturation factor (GMF); null mutant displays elevated mitochondrial genome loss
CAGL0G02937g		1,24	9,86E-03	-	Ortholog(s) have pyruvate decarboxylase activity
CAGL0B00594g		1,24	2,26E-03	*YCL048W-A*	Putative protein of unknown function
CAGL0F08041g	*CgPFK1*	1,23	5,56E-04	*PFK1*	Alpha subunit of heterooctameric phosphofructokinase involved in glycolysis, indispensable for anaerobic growth, activated by fructose-2,6-bisphosphate and AMP, mutation inhibits glucose induction of cell cycle-related genes
CAGL0A03718g		1,23	1,70E-03	*CHC1*	Clathrin heavy chain, subunit of the major coat protein involved in intracellular protein transport and endocytosis; two heavy chains form the clathrin triskelion structural component; the light chain (CLC1) is thought to regulate function
CAGL0B02755g		1,23	6,93E-03	*YLR361C-A*	Putative protein of unknown function
CAGL0E01243g		1,23	1,09E-03	*CTH1*	Member of the CCCH zinc finger family; has similarity to mammalian Tis11 protein, which activates transcription and also has a role in mRNA degradation; may function with Tis11p in iron homeostasis
CAGL0C02893g		1,22	1,21E-03	*HRK1*	Protein kinase implicated in activation of the plasma membrane H(+)-ATPase Pma1p in response to glucose metabolism; plays a role in ion homeostasis
CAGL0I08305g		1,22	8,44E-03	*YAT2*	Carnitine acetyltransferase; has similarity to Yat1p, which is a carnitine acetyltransferase associated with the mitochondrial outer membrane
CAGL0L06776g		1,22	3,27E-04	*GAT2*	Protein containing GATA family zinc finger motifs; similar to Gln3p and Dal80p; expression repressed by leucine
CAGL0M04675g		1,22	3,52E-03	*RDL1*	Protein of unknown function containing a rhodanese-like domain; localized to the mitochondrial outer membrane
CAGL0I08613g	*CgDUR3*	1,22	3,06E-03	*DUR3*	Putative plasma membrane polyamine transporter
CAGL0E03674g	*CgTPO1_2*	1,22	9,53E-04	*TPO1*	Polyamine transporter that recognizes spermine, putrescine, and spermidine; catalyzes uptake of polyamines at alkaline pH and excretion at acidic pH; phosphorylation enhances activity and sorting to the plasma membrane
CAGL0E03652g		1,21	9,26E-04	*ISA1*	Protein required for maturation of mitochondrial [4Fe-4S] proteins; functions in a complex with Isa2p and possibly Iba57p; isa1 deletion causes loss of mitochondrial DNA and respiratory deficiency; depletion reduces growth on nonfermentable carbon sources; functional ortholog of bacterial A-type ISC proteins
CAGL0K03069g		1,21	4,94E-03	*IRC21*	Putative protein of unknown function; may be involved in resistance to carboplatin and cisplatin; null mutant displays increase in spontaneous Rad52p foci; contains a lipid-binding domain and binds cardiolipin in a large-scale study
CAGL0C02739g		1,21	2,88E-03	*FUN14*	Mitochondrial protein of unknown function

CAGL0G01870g		1,20	1,97E-03	-	-
CAGL0I06204g	*CgPIR1*	1,20	3,04E-03	*YJL160C*	Putative protein of unknown function; member of the PIR (proteins with internal repeats) family of cell wall proteins; non-essential gene that is required for sporulation; mRNA is weakly cell cycle regulated, peaking in mitosis
CAGL0K04191g		1,19	1,07E-03	*BUD9*	Protein involved in bud-site selection; mutant has increased aneuploidy tolerance; diploid mutants display a unipolar budding pattern instead of the wild-type bipolar pattern, and bud at the distal pole
CAGL0F04521g		1,19	1,42E-03	*ECM13*	Non-essential protein of unknown function; induced by treatment with 8-methoxypsoralen and UVA irradiation
CAGL0I02574g		1,18	2,21E-03	*OYE2*	Conserved NADPH oxidoreductase containing flavin mononucleotide (FMN), homologous to Oye3p with different ligand binding and catalytic properties; may be involved in sterol metabolism, oxidative stress response, and programmed cell death
CAGL0C03597g	*CgABP1*	1,18	2,77E-03	*ABP1*	Actin-binding protein of the cortical actin cytoskeleton, important for activation of the Arp2/3 complex that plays a key role actin in cytoskeleton organization; phosphorylation within its PRR (Proline-Rich Region), mediated by Cdc28p and Pho85p, protects Abp1p from proteolysis mediated by its own PEST sequences
CAGL0G09273g		1,17	8,75E-03	*SNG1*	Protein involved in resistance to nitrosoguanidine (MNNG) and 6-azauracil (6-AU); expression is regulated by transcription factors involved in multidrug resistance
CAGL0G05830g		1,17	2,06E-03	*CRP1*	Protein that binds to cruciform DNA structures
CAGL0G08844g		1,16	4,65E-03	*ASG1*	Zinc cluster protein proposed to function as a transcriptional regulator involved in the stress response; null mutants have a respiratory deficiency, calcofluor white sensitivity and slightly increased cycloheximide resistance
CAGL0I08591g		1,16	2,25E-03	*YER158C*	Protein of unknown function, has similarity to Afr1p; potentially phosphorylated by Cdc28p
CAGL0J11176g		1,16	6,84E-03	*TDA7*	Cell cycle-regulated gene of unknown function, promoter bound by Fkh2p; null mutant is sensitive to expression of the top1-T722A allele
CAGL0F04917g		1,15	3,22E-03	*PIG1*	Putative targeting subunit for the type-1 protein phosphatase Glc7p that tethers it to the Gsy2p glycogen synthase
CAGL0M06655g		1,15	7,91E-04	*HHT2*	Histone H3, core histone protein required for chromatin assembly, part of heterochromatin-mediated telomeric and HM silencing; one of two identical histone H3 proteins (see HHT1); regulated by acetylation, methylation, and phosphorylation
CAGL0G02849g		1,15	9,30E-03	*UIP4*	Protein that interacts with Ulp1p, a Ubl (ubiquitin-like protein)-specific protease for Smt3p protein conjugates; detected in a phosphorylated state in the mitochondrial outer membrane; also detected in ER and nuclear envelope
CAGL0I10516g		1,14	7,09E-04	*YGR130C*	Component of the eisosome with unknown function; GFP-fusion protein localizes to the cytoplasm; specifically phosphorylated in vitro by mammalian diphosphoinositol pentakisphosphate (IP7)
CAGL0B01100g		1,14	3,08E-03	*TFS1*	Protein that interacts with and inhibits carboxypeptidase Y and Ira2p; phosphatidylethanolamine-binding protein (PEBP) family member; targets to vacuolar membranes during stationary phase; acetylated by NatB N-terminal acetyltransferase
CAGL0E01969g		1,14	4,94E-03	*SMF1*	Divalent metal ion transporter with a broad specificity for di-valent and tri-valent metals; post-translationally regulated by levels of metal ions; member of the Nramp family of metal transport proteins
CAGL0F06017g	*CgLYS7*	1,14	1,45E-03	*CCS1*	Copper chaperone for superoxide dismutase Sod1p, involved in oxidative stress protection; Met-X-Cys-X2-Cys motif within the N-terminal portion is involved in insertion of copper into Sod1p under conditions of copper deprivation
CAGL0M08206g		1,13	2,32E-03	*YJL171C*	GPI-anchored cell wall protein of unknown function; induced in response to cell wall damaging agents and by mutations in genes involved in cell wall biogenesis; sequence similarity to YBR162C/TOS1, a covalently bound cell wall protein
CAGL0M06237g		1,13	8,69E-03	*EHT1*	Acyl-coenzymeA:ethanol O-acyltransferase that plays a minor role in medium-chain fatty acid ethyl ester biosynthesis; possesses short-chain esterase activity; localizes to lipid particles and the mitochondrial outer membrane
CAGL0H03993g		1,13	2,78E-03	*CIT1*	Citrate synthase, catalyzes the condensation of acetyl coenzyme A and oxaloacetate to form citrate; the rate-limiting enzyme of the TCA cycle; nuclear encoded mitochondrial protein
CAGL0L11154g		1,13	2,99E-03	*NTE1*	Serine esterase, homolog of human neuropathy target esterase (NTE); Nte1p-mediated phosphatidylcholine turnover influences transcription factor Opi1p localization, affecting transcriptional regulation of phospholipid biosynthesis genes
CAGL0G09042g		1,13	7,85E-02	*AFT2*	Iron-regulated transcriptional activator; activates genes involved in intracellular iron use and required for iron homeostasis and resistance to oxidative stress; similar to Aft1p
CAGL0B04917g		1,12	4,79E-03	*IDP2*	Cytosolic NADP-specific isocitrate dehydrogenase, catalyzes oxidation of isocitrate to alpha-ketoglutarate; levels are elevated during growth on non-fermentable carbon sources and reduced during growth on glucose
CAGL0L01177g	*CgFRDS1*	1,12	6,56E-04	*YEL047C*	Soluble fumarate reductase, required with isoenzyme Osm1p for anaerobic growth; may interact with ribosomes, based on co-purification experiments; authentic, non-tagged protein is detected in purified mitochondria in high-throughput studies
CAGL0J11770g	*CgPLB1*	1,11	2,35E-03	*PLB1*	Phospholipase B (lysophospholipase) involved in lipid metabolism, required for deacylation of phosphatidylcholine and phosphatidylethanolamine but not phosphatidylinositol
CAGL0J11110g		1,11	1,65E-03	*YJR003C*	Putative protein of unknown function; detected in highly purified mitochondria in high-throughput studies; predicted to be involved in ribosome biogenesis
CAGL0K05203g		1,11	4,33E-03	*YOP1*	Membrane protein that interacts with Yip1p to mediate membrane traffic; interacts with Sey1p to maintain ER morphology; overexpression leads to cell death and accumulation of internal cell membranes
CAGL0G02673g		1,11	6,59E-04	*IDH1*	Subunit of mitochondrial NAD(+)-dependent isocitrate dehydrogenase, which catalyzes the oxidation of isocitrate to alpha-ketoglutarate in the TCA cycle
CAGL0M11000g		1,10	2,23E-03	*YNR034W-A*	Putative protein of unknown function; expression is regulated by Msn2p/Msn4p
CAGL0G03531g		1,10	6,87E-03	*SPR6*	Protein of unknown function, expressed during sporulation; not required for sporulation, but gene exhibits genetic interactions with other genes required for sporulation
CAGL0J04268g		1,10	5,31E-03	*ACH1*	Protein with CoA transferase activity, particularly for CoASH transfer from succinyl-CoA to acetate; has minor acetyl-CoA-hydrolase activity; phosphorylated; required for acetate utilization and for diploid pseudohyphal growth
CAGL0J06380g		1,09	3,50E-03	*INH1*	Protein that inhibits ATP hydrolysis by the F1F0-ATP synthase; inhibitory function is enhanced by stabilizing proteins Stf1p and Stf2p; has similarity to Stf1p; has a calmodulin-binding motif and binds calmodulin in vitro
CAGL0G00286g	*CgGAS1*	1,09	1,45E-03	*GAS1*	Beta-1,3-glucanosyltransferase, required for cell wall assembly and also has a role in transcriptional silencing; localizes to the cell surface via a glycosylphosphatidylinositol (GPI) anchor; also found at the nuclear periphery
CAGL0J04004g		1,09	7,63E-02	*YOR228C*	Protein of unknown function, localized to the mitochondrial outer membrane
CAGL0K04719g		1,09	2,28E-03	*YNL208W*	Protein of unknown function; may interact with ribosomes, based on co-purification experiments; authentic, non-tagged protein is detected in purified mitochondria in high-throughput studies; potential orthologs found in other fungi
CAGL0C01705g	*CgGPX2*	1,08	6,24E-03	*GPX2*	Phospholipid hydroperoxide glutathione peroxidase induced by glucose starvation that protects cells from phospholipid hydroperoxides and nonphospholipid peroxides during oxidative stress
CAGL0G06842g		1,08	6,00E-03	*BBC1*	Protein possibly involved in assembly of actin patches; interacts with an actin assembly factor Las17p and with the SH3 domains of Type I myosins Myo3p and Myo5p; localized predominantly to cortical actin patches
CAGL0G05544g		1,08	9,76E-03	*HBT1*	Substrate of the Hub1p ubiquitin-like protein that localizes to the shmoo tip (mating projection); mutants are defective for mating projection formation, thereby implicating Hbt1p in polarized cell morphogenesis
CAGL0M06677g		1,08	7,02E-02	*HHF2*	Histone H4, core histone protein required for chromatin assembly and chromosome function; one of two identical histone proteins (see also HHF1); contributes to telomeric silencing; N-terminal domain involved in maintaining genomic integrity
CAGL0J03058g		1,08	5,35E-03	*ICL1*	Isocitrate lyase, catalyzes the formation of succinate and glyoxylate from isocitrate, a key reaction of the glyoxylate cycle; expression of ICL1 is induced by growth on ethanol and repressed by growth on glucose

CAGL0B02145g		1,07	2,95E-03	*TOS2*	Protein involved in localization of Cdc24p to the site of bud growth; may act as a membrane anchor; localizes to the bud neck and bud tip; potentially phosphorylated by Cdc28p
CAGL0G08712g		1,07	1,19E-03	*KGD1*	Component of the mitochondrial alpha-ketoglutarate dehydrogenase complex, which catalyzes a key step in the tricarboxylic acid (TCA) cycle, the oxidative decarboxylation of alpha-ketoglutarate to form succinyl-CoA
CAGL0L02321g		1,07	2,25E-03	*MET14*	Adenylylsulfate kinase, required for sulfate assimilation and involved in methionine metabolism
CAGL0K07634g	*CgGAT1*	1,07	4,61E-03	*GAT1*	Transcriptional activator of genes involved in nitrogen catabolite repression; contains a GATA-1-type zinc finger DNA-binding motif; activity and localization regulated by nitrogen limitation and Ure2p
CAGL0C03113g		1,07	3,06E-03	*DCS1*	Non-essential hydrolase involved in mRNA decapping; activates Xrn1p; may function in a feedback mechanism to regulate deadenylation, contains pyrophosphatase activity and a HIT (histidine triad) motif; interacts with neutral trehalase Nth1p; required for growth on glycerol medium
CAGL0E04774g		1,05	4,11E-03	*YDR222W*	Protein of unknown function; green fluorescent protein (GFP)-fusion protein localizes to the cytoplasm in a punctate pattern
CAGL0F07821g		1,05	2,55E-03	*HUL5*	Multiubiquitin chain assembly factor (E4); proteasome processivity factor that elongates polyUb chains on substrates, opposing Ubp6p, a branched polyubiquitin protease; required for retrograde transport of misfolded proteins during ERAD
CAGL0L09933g		1,05	4,11E-03	*CUE5*	Protein containing a CUE domain that binds ubiquitin, which may facilitate intramolecular monoubiquitination; green fluorescent protein (GFP)-fusion protein localizes to the cytoplasm in a punctate pattern
CAGL0B02860g		1,05	3,01E-03	*ATG33*	Mitochondrial mitophagy-specific protein; required primarily for mitophagy induced at the post-log phase; not required for other types of selective autophagy or macroautophagy; conserved within fungi, but not in higher eukaryotes
CAGL0C02673g		1,04	3,83E-03	*ART10*	Protein of unknown function that contains 2 PY motifs and is ubiquinated by Rsp5p; overexpression confers resistance to arsenite; green fluorescent protein (GFP)-fusion protein localizes it to the cytoplasm; non-essential gene
CAGL0K04037g	*CgFKS2*	1,04	1,24E-03	*GSC2*	Catalytic subunit of 1,3-beta-glucan synthase, involved in formation of the inner layer of the spore wall; activity positively regulated by Rho1p and negatively by Smk1p; has similarity to an alternate catalytic subunit, Fks1p (Gsc1p)
CAGL0F03267g		1,03	9,42E-03	*YHR080C*	Protein of unknown function that may interact with ribosomes, based on co-purification experiments; the authentic, non-tagged protein is detected in highly purified mitochondria in high-throughput studies
CAGL0G10219g	*CgAWP12*	1,02	2,28E-03	-	Adhesin-like protein with 5 tandem repeats
CAGL0H10032g	*CgPST2*	1,02	8,59E-03	*RFS1*	Protein of unknown function; member of a flavodoxin-like fold protein family that includes Pst2p and Ycp4p; green fluorescent protein (GFP)-fusion protein localizes to the cytoplasm in a punctate pattern
CAGL0L06270g	*CgBHM1_B*	1,01	2,75E-03	*BMH2*	14-3-3 protein, minor isoform; controls proteome at post-transcriptional level, binds proteins and DNA, involved in regulation of many processes including exocytosis, vesicle transport, Ras/MAPK signaling, and rapamycin-sensitive signaling
CAGL0I01342g		1,00	2,57E-03	*GLY1*	Threonine aldolase, catalyzes the cleavage of L-allo-threonine and L-threonine to glycine; involved in glycine biosynthesis
CAGL0K04213g		1,00	6,28E-03	*YGR042W*	Putative protein of unknown function; involved in maintenance of proper telomere length; green fluorescent protein (GFP)-fusion protein localizes to both the cytoplasm and the nucleus
CAGL0J00539g	*CgSLT2*	1,00	1,97E-01	*SLT2*	Serine/threonine MAP kinase; involved in regulating maintenance of cell wall integrity, progression through the cell cycle, and nuclear mRNA retention in heat shock; required for mitophagy and pexophagy; affects recruitment of mitochondria to the phagophore assembly site (PAS); regulated by the PKC1-mediated signaling pathway

DOWN-REGULATED GENES

ORF	Gene Name	log_2FC (Δ*Cghaa1* AC/ Δ*Cghaa1* CTRL)	p_value	*S. cerevisiae* orthologue	Function
CAGL0H02409g		-1,00	3,10E-03	*TIF11*	Translation initiation factor eIF1A, essential protein that forms a complex with Sui1p (eIF1) and the 40S ribosomal subunit and scans for the start codon; C-terminus associates with Fun12p (eIF5B); N terminus interacts with eIF2 and eIF3
CAGL0H04521g		-1,01	9,04E-04	*RPL32*	Protein component of the large (60S) ribosomal subunit, has similarity to rat L32 ribosomal protein; overexpression disrupts telomeric silencing
CAGL0G00154g		-1,01	1,93E-03	*ZUO1*	Ribosome-associated chaperone, functions in ribosome biogenesis and, in partnership with Ssz1p and SSb1/2, as a chaperone for nascent polypeptide chains; contains a DnaJ domain and functions as a J-protein partner for Ssb1p and Ssb2p
CAGL0E05764g		-1,01	4,07E-03	*PUS1*	tRNA:pseudouridine synthase, introduces pseudouridines at positions 26-28, 34-36, 65, and 67 of tRNA; nuclear protein that appears to be involved in tRNA export; also acts on U2 snRNA
CAGL0H00462g		-1,01	9,88E-03	*RPS5*	Protein component of the small (40S) ribosomal subunit, the least basic of the non-acidic ribosomal proteins; phosphorylated in vivo; essential for viability; has similarity to E. coli S7 and rat S5 ribosomal proteins
CAGL0F04983g		-1,01	4,52E-03	*DBP9*	DEAD-box protein required for 27S rRNA processing; exhibits DNA, RNA and DNA/RNA helicase activities; ATPase activity shows preference for DNA over RNA; DNA helicase activity abolished by mutation in RNA-binding domain
CAGL0J00957g		-1,01	6,51E-03	*RLP24*	Essential protein with similarity to Rpl24Ap and Rpl24Bp, associated with pre-60S ribosomal subunits and required for ribosomal large subunit biogenesis
CAGL0L05566g		-1,02	2,66E-03	*GCD14*	Subunit of tRNA (1-methyladenosine) methyltransferase, with Gcd10p, required for the modification of the adenine at position 58 in tRNAs, especially tRNAi-Met; first identified as a negative regulator of GCN4 expression
CAGL0A02090g		-1,02	9,66E-03	*YHR020W*	Protein of unknown function that may interact with ribosomes, based on co-purification experiments; has similarity to proline-tRNA ligase; YHR020W is an essential gene
CAGL0K03795g		-1,02	8,02E-03	*ECM16*	Essential DEAH-box ATP-dependent RNA helicase specific to the U3 snoRNP, predominantly nucleolar in distribution, required for 18S rRNA synthesis
CAGL0L11638g		-1,02	1,96E-03	*ESF1*	Nucleolar protein involved in pre-rRNA processing; depletion causes severely decreased 18S rRNA levels
CAGL0H00957g		-1,03	2,78E-03	*SUI3*	Beta subunit of the translation initiation factor eIF2, involved in the identification of the start codon; proposed to be involved in mRNA binding
CAGL0F01749g		-1,03	1,12E-03	*SHM2*	Cytosolic serine hydroxymethyltransferase, converts serine to glycine plus 5,10 methylenetetrahydrofolate; major isoform involved in generating precursors for purine, pyrimidine, amino acid, and lipid biosynthesis
CAGL0K12804g		-1,03	3,35E-03	*BUD20*	Protein involved in bud-site selection; diploid mutants display a random budding pattern instead of the wild-type bipolar pattern
CAGL0D02706g		-1,04	2,69E-03	*RIX1*	Essential component of the Rix1 complex (Rix1p, Ipi1p, Ipi3p) that is required for processing of ITS2 sequences from 35S pre-rRNA; Rix1 complex associates with Mdn1p in pre-60S ribosomal particles
CAGL0E05500g		-1,04	1,32E-02	*RPA190*	RNA polymerase I largest subunit A190
CAGL0I02398g		-1,04	7,70E-03	*NMD3*	Protein involved in nuclear export of the large ribosomal subunit; acts as a Crm1p-dependent adapter protein for export of nascent ribosomal subunits through the nuclear pore complex
CAGL0L12364g		-1,04	1,74E-03	*ERG10*	Acetyl-CoA C-acetyltransferase (acetoacetyl-CoA thiolase), cytosolic enzyme that transfers an acetyl group from one acetyl-CoA molecule to another, forming acetoacetyl-CoA; involved in the first step in mevalonate biosynthesis
CAGL0B01881g		-1,05	9,59E-03	*TRM1*	tRNA methyltransferase; two forms of the protein are made by alternative translation starts; localizes to both the nucleus and mitochondrion to produce the modified base N2,N2-dimethylguanosine in tRNAs in both compartments

CAGL0M01430g		-1,05	4,85E-03	*UTP4*	Subunit of U3-containing 90S preribosome and Small Subunit (SSU) processome complexes involved in production of 18S rRNA and assembly of small ribosomal subunit; member of t-Utp subcomplex involved with transcription of 35S rRNA transcript
CAGL0B00792g		-1,05	2,18E-03	*SRO9*	Cytoplasmic RNA-binding protein that associates with translating ribosomes; involved in heme regulation of Hap1p as a component of the HMC complex, also involved in the organization of actin filaments; contains a La motif
CAGL0K06567g		-1,05	3,00E-03	*RPL27B*	Protein component of the large (60S) ribosomal subunit, nearly identical to Rpl27Ap and has similarity to rat L27 ribosomal protein
CAGL0B04125g		-1,05	5,51E-03	*RPC40*	RNA polymerase subunit AC40, common to RNA polymerase I and III
CAGL0E03938g		-1,06	2,49E-03	*RPL8A*	Ribosomal protein L4 of the large (60S) ribosomal subunit, nearly identical to Rpl8Bp and has similarity to rat L7a ribosomal protein; mutation results in decreased amounts of free 60S subunits
CAGL0F02937g		-1,06	6,80E-03	*RPL12B*	Protein component of the large (60S) ribosomal subunit, nearly identical to Rpl12Ap; rpl12a rpl12b double mutant exhibits slow growth and slow translation; has similarity to E. coli L11 and rat L12 ribosomal proteins
CAGL0B01232g	CgEMG1	-1,06	9,92E-03	*EMG1*	Member of the alpha/beta knot fold methyltransferase superfamily; required for maturation of 18S rRNA and for 40S ribosome production; interacts with RNA and with S-adenosylmethionine; associates with spindle/microtubules; forms homodimers; human ortholog is mutated in Bowen-Conradi syndrome
CAGL0M04829g		-1,06	8,57E-03	*RRP5*	RNA binding protein with preference for single stranded tracts of U's involved in synthesis of both 18S and 5.8S rRNAs; component of both the ribosomal small subunit (SSU) processosome and the 90S preribosome
CAGL0H03773g		-1,07	8,22E-03	*RLP7*	Nucleolar protein with similarity to large ribosomal subunit L7 proteins; constituent of 66S pre-ribosomal particles; plays an essential role in processing of precursors to the large ribosomal subunit RNAs
CAGL0A00979g		-1,07	5,96E-04	*RPL38*	Protein component of the large (60S) ribosomal subunit, has similarity to rat L38 ribosomal protein
CAGL0I03080g	CgURA3	-1,09	3,73E-03	*URA3*	Orotidine-5'-phosphate (OMP) decarboxylase, catalyzes the sixth enzymatic step in the de novo biosynthesis of pyrimidines, converting OMP into uridine monophosphate (UMP); converts 5-FOA into 5-fluorouracil, a toxic compound
CAGL0I08327g		-1,09	2,52E-03	*GCD11*	Gamma subunit of the translation initiation factor eIF2, involved in the identification of the start codon; binds GTP when forming the ternary complex with GTP and tRNAi-Met
CAGL0K04499g		-1,09	6,22E-03	*ADE6*	Formylglycinamidine-ribonucleotide (FGAM)-synthetase, catalyzes a step in the 'de novo' purine nucleotide biosynthetic pathway
CAGL0L04950g		-1,09	4,83E-03	*ERB1*	Constituent of 66S pre-ribosomal particles, forms a complex with Nop7p and Ytm1p that is required for maturation of the large ribosomal subunit; required for maturation of the 25S and 5.8S ribosomal RNAs; homologous to mammalian Bop1
CAGL0K02541g		-1,10	2,21E-03	*PRS3*	5-phospho-ribosyl-1(alpha)-pyrophosphate synthetase, synthesizes PRPP, which is required for nucleotide, histidine, and tryptophan biosynthesis; one of five related enzymes, which are active as heteromultimeric complexes
CAGL0F02541g		-1,10	4,61E-02	*UTP5*	Subunit of U3-containing Small Subunit (SSU) processome complex involved in production of 18S rRNA and assembly of small ribosomal subunit
CAGL0F01045g		-1,10	3,81E-03	*RPS15*	Protein component of the small (40S) ribosomal subunit; has similarity to E. coli S19 and rat S15 ribosomal proteins
CAGL0D03718g	CgTIF5	-1,10	1,86E-03	*TIF5*	Translation initiation factor eIF5; functions both as a GTPase-activating protein to mediate hydrolysis of ribosome-bound GTP and as a GDP dissociation inhibitor to prevent recycling of eIF2
CAGL0K01991g		-1,11	3,93E-03	*NCL1*	S-adenosyl-L-methionine-dependent tRNA: m5C-methyltransferase, methylates cytosine to m5C at several positions in tRNAs and intron-containing pre-tRNAs; increases proportion of tRNALeu(CAA) with m5C at wobble position in response to hydrogen peroxide, causing selective translation of mRNA from genes enriched in TTG codon; loss of NCL1 confers hypersensitivity to oxidative stress; similar to Nop2p and human
CAGL0J11286g		-1,11	4,68E-03	*IPI3*	Essential component of the Rix1 complex (Rix1p, Ipi1p, Ipi3p) that is required for processing of ITS2 sequences from 35S pre-rRNA; highly conserved and contains WD40 motifs; Rix1 complex associates with Mdn1p in pre-60S ribosomal particles
CAGL0L00341g		-1,11	2,84E-03	*EBP2*	Required for 25S rRNA maturation and 60S ribosomal subunit assembly; localizes to the nucleolus and in foci along nuclear periphery; constituent of 66S pre-ribosomal particles; cooperates with Rrs1p and Mps3p to mediate telomere clustering by binding Sir4p, but is not involved in telomere tethering
CAGL0E04994g		-1,12	6,77E-04	*RPL9A*	Protein component of the large (60S) ribosomal subunit, nearly identical to Rpl9Bp and has similarity to E. coli L6 and rat L9 ribosomal proteins
CAGL0L03806g		-1,12	2,80E-03	*NOP15*	Constituent of 66S pre-ribosomal particles, involved in 60S ribosomal subunit biogenesis; localizes to both nucleolus and cytoplasm
CAGL0L01551g		-1,13	9,99E-04	*SUR7*	Plasma membrane protein that localizes to furrow-like invaginations (MCC patches); component of eisosomes; associated with endocytosis, along with Pil1p and Lsp1p; sporulation and plasma membrane sphingolipid content are altered in mutants
CAGL0M13893g		-1,14	1,85E-01	*NIP1*	eIF3c subunit of the eukaryotic translation initiation factor 3 (eIF3), involved in the assembly of preinitiation complex and start codon selection
CAGL0G01991g		-1,14	1,67E-03	*NOB1*	Essential nuclear protein involved in proteasome maturation and synthesis of 40S ribosomal subunits; required for cleavage of the 20S pre-rRNA to generate the mature 18S rRNA
CAGL0J03124g		-1,14	1,19E-03	*ARG5,6*	Acetylglutamate kinase and N-acetyl-gamma-glutamyl-phosphate reductase; N-acetyl-L-glutamate kinase (NAGK) catalyzes the 2nd and N-acetyl-gamma-glutamyl-phosphate reductase (NAGSA), the 3rd step in arginine biosynthesis; synthesized as a precursor which is processed in the mitochondrion to yield mature NAGK and NAGSA; enzymes form a metabolon complex with Arg2p; NAGK C-terminal domain stabilizes the
CAGL0K01551g		-1,15	2,53E-03	*DBP10*	Putative ATP-dependent RNA helicase of the DEAD-box protein family, constituent of 66S pre-ribosomal particles; essential protein involved in ribosome biogenesis
CAGL0F02563g	CgHPT1	-1,15	4,90E-03	*HPT1*	Dimeric hypoxanthine-guanine phosphoribosyltransferase, catalyzes the transfer of the phosphoribosyl portion of 5-phosphoribosyl-alpha-1-pyrophosphate to a purine base (either guanine or hypoxanthine) to form pyrophosphate and a purine nucleotide (either guanosine monophosphate or inosine monophosphate); mutations in the human homolog HPRT1 can cause Lesch-Nyhan syndrome and Kelley-Seegmiller syndrome
CAGL0B02794g		-1,15	1,77E-03	*ADE13*	Adenylosuccinate lyase, catalyzes two steps in the 'de novo' purine nucleotide biosynthetic pathway; expression is repressed by adenine and activated by Bas1p and Pho2p; mutations in human ortholog ADSL cause adenylosuccinase deficiency
CAGL0I08393g	CgTIF3	-1,15	1,69E-03	*TIF3*	Translation initiation factor eIF-4B, has RNA annealing activity; contains an RNA recognition motif and binds to single-stranded RNA
CAGL0J10032g		-1,15	3,46E-03	*NOP2*	Probable RNA m(5)C methyltransferase, essential for processing and maturation of 27S pre-rRNA and large ribosomal subunit biogenesis; localized to the nucleolus; constituent of 66S pre-ribosomal particles
CAGL0D00880g		-1,15	8,03E-03	*TSR1*	Protein required for processing of 20S pre-rRNA in the cytoplasm; associates with pre-40S ribosomal particles; inhibits the premature association of 60S subunits with assembling 40S subunits in the cytoplasm; similar to Bms1p
CAGL0D00550g		-1,16	1,98E-03	*PRS1*	5-phospho-ribosyl-1(alpha)-pyrophosphate synthetase, synthesizes PRPP, which is required for nucleotide, histidine, and tryptophan biosynthesis; one of five related enzymes, which are active as heteromultimeric complexes
CAGL0G02409g		-1,16	2,69E-03	*SRP40*	Nucleolar, serine-rich protein with a role in preribosome assembly or transport; may function as a chaperone of small nucleolar ribonucleoprotein particles (snoRNPs); immunologically and structurally to rat Nopp140
CAGL0L06952g	CgTIF34	-1,16	2,99E-03	*TIF34*	eIF3i subunit of the core complex of translation initiation factor 3 (eIF3), which is essential for translation; stimulates rate of ribosomal scanning during translation reinitiation
CAGL0E02673g		-1,16	2,10E-03	*UTP23*	Component of the small subunit processome, involved in 40S ribosomal subunit biogenesis; interacts with snR30 and is required for dissociation of snR30 from large pre-ribosomal particles; has homology to PINc domain protein Fcf1p, although the PINc domain of Utp23p is not required for function; essential protein
CAGL0J06374g		-1,16	7,11E-04	*RPP1B*	Ribosomal protein P1 beta, component of the ribosomal stalk, which is involved in interaction of translational elongation factors with ribosome; accumulation is regulated by phosphorylation and interaction with the P2 stalk component
CAGL0B01507g	CgARG8	-1,16	5,17E-03	*ARG8*	Acetylornithine aminotransferase, catalyzes the fourth step in the biosynthesis of the arginine precursor ornithine
CAGL0B00286g		-1,16	6,43E-03	*CHA1*	Catabolic L-serine (L-threonine) deaminase, catalyzes the degradation of both L-serine and L-threonine; required to use serine or threonine as the sole nitrogen source, transcriptionally induced by serine and threonine
CAGL0K09966g	CgPRT1	-1,17	7,06E-04	*PRT1*	eIF3b subunit of the core complex of translation initiation factor 3 (eIF3), essential for translation; part of a subcomplex (Prt1p-Rpg1p-Nip1p) that stimulates binding of mRNA and tRNA(i)Met to ribosomes

CAGL0J07238g	CgRPS12	-1,18	5,29E-03	*RPS12*	Protein component of the small (40S) ribosomal subunit; has similarity to rat ribosomal protein S12
CAGL0K02233g		-1,18	7,63E-03	*NSA2*	Protein constituent of 66S pre-ribosomal particles, contributes to processing of the 27S pre-rRNA
CAGL0E02013g		-1,19	5,68E-04	*RPL18A*	Protein component of the large (60S) ribosomal subunit, identical to Rpl18Bp and has similarity to rat L18 ribosomal protein; intron of RPL18A pre-mRNA forms stem-loop structures that are a target for Rnt1p cleavage leading to degradation
CAGL0M11616g		-1,21	1,12E-03	*MDN1*	Huge dynein-related AAA-type ATPase (midasin), forms extended pre-60S particle with the Rix1 complex (Rix1p-Ipi1p-Ipi3p); acts in removal of ribosomal biogenesis factors at successive steps of pre-60S assembly and export from nucleus
CAGL0H02937g		-1,21	1,21E-03	*UTP18*	Possible U3 snoRNP protein involved in maturation of pre-18S rRNA, based on computational analysis of large-scale protein-protein interaction data
CAGL0J03476g		-1,21	7,73E-03	*RSA4*	WD-repeat protein involved in ribosome biogenesis; may interact with ribosomes; required for maturation and efficient intra-nuclear transport or pre-60S ribosomal subunits, localizes to the nucleolus
CAGL0K09042g		-1,21	1,59E-03	*TMA16*	Protein of unknown function that associates with ribosomes
CAGL0M12881g		-1,21	8,18E-04	*NA*	NA
CAGL0H09724g		-1,22	1,12E-03	*NUG1*	GTPase that associates with nuclear 60S pre-ribosomes, required for export of 60S ribosomal subunits from the nucleus
CAGL0M03487g		-1,22	7,57E-03	*RPC34*	RNA polymerase III subunit C34; interacts with TFIIIB70 and is a key determinant in pol III recruitment by the preinitiation complex
CAGL0M05775g		-1,23	6,47E-03	*NIP7*	Nucleolar protein required for 60S ribosome subunit biogenesis, constituent of 66S pre-ribosomal particles; physically interacts with Nop8p and the exosome subunit Rrp43p
CAGL0L06314g		-1,23	2,08E-03	*ARX1*	Shuttling pre-60S factor; involved in the biogenesis of ribosomal large subunit biogenesis; interacts directly with Alb1; responsible for Tif6 recycling defects in absence of Rei1; associated with the ribosomal export complex
CAGL0G07843g		-1,24	1,17E-02	*NOP7*	Component of several different pre-ribosomal particles; forms a complex with Ytm1p and Erb1p that is required for maturation of the large ribosomal subunit; required for exit from G₀ and the initiation of cell proliferation
CAGL0D01562g	CgFCY1	-1,26	5,96E-03	*FCY1*	Cytosine deaminase, zinc metalloenzyme that catalyzes the hydrolytic deamination of cytosine to uracil; of biomedical interest because it also catalyzes the deamination of 5-fluorocytosine (5FC) to form anticancer drug 5-fluorouracil (5FU)
CAGL0E03289g		-1,26	1,33E-03	*TIF4631*	Translation initiation factor eIF4G, subunit of the mRNA cap-binding protein complex (eIF4F) that also contains eIF4E (Cdc33p); interacts with Pab1p and with eIF4A (Tif1p); also has a role in biogenesis of the large ribosomal subunit
CAGL0M13519g		-1,26	1,28E-03	*HAS1*	ATP-dependent RNA helicase; localizes to both the nuclear periphery and nucleolus; highly enriched in nuclear pore complex fractions; constituent of 66S pre-ribosomal particles
CAGL0L03846g		-1,26	9,42E-03	*DBP2*	Essential ATP-dependent RNA helicase of the DEAD-box protein family, involved in nonsense-mediated mRNA decay and rRNA processing
CAGL0K11880g		-1,27	4,49E-04	*MRH1*	Protein that localizes primarily to the plasma membrane, also found at the nuclear envelope; the authentic, non-tagged protein is detected in mitochondria in a phosphorylated state; has similarity to Hsp30p and Yro2p
CAGL0K10362g		-1,27	7,29E-02	*ORT1*	Ornithine transporter of the mitochondrial inner membrane, exports ornithine from mitochondria as part of arginine biosynthesis; human ortholog is associated with hyperammonaemia-hyperornithinaemia-homocitrullinuria (HHH) syndrome
CAGL0I08987g		-1,27	2,72E-03	*ARG4*	Argininosuccinate lyase, catalyzes the final step in the arginine biosynthesis pathway
CAGL0I06490g		-1,28	1,32E-03	*JIP5*	Essential protein required for biogenesis of the large ribosomal subunit; interacts with proteins involved in RNA processing, ribosome biogenesis, ubiquitination and demethylation; similar to WDR55, a human WD repeat protein
CAGL0A04015g		-1,29	2,63E-04	*NOP56*	Essential evolutionarily-conserved nucleolar protein component of the box C/D snoRNP complexes that direct 2'-O-methylation of pre-rRNA during its maturation; overexpression causes spindle orientation defects
CAGL0C03369g		-1,30	7,37E-04	*NOG2*	Putative GTPase that associates with pre-60S ribosomal subunits in the nucleolus and is required for their nuclear export and maturation
CAGL0I03234g		-1,30	1,78E-03	*SNU13*	RNA binding protein, part of U3 snoRNP involved in rRNA processing, part of U4/U6-U5 tri-snRNP involved in mRNA splicing, similar to human 15.5K protein
CAGL0C02343g		-1,30	5,49E-04	*ARB1*	ATPase of the ATP-binding cassette (ABC) family involved in 40S and 60S ribosome biogenesis, has similarity to Gcn20p; shuttles from nucleus to cytoplasm, physically interacts with Tif6p, Lsg1p
CAGL0L08756g		-1,30	1,97E-03	*RRP12*	Protein required for export of the ribosomal subunits; associates with the RNA components of the pre-ribosomes; has a role in nuclear import in association with Pse1p; contains HEAT-repeats
CAGL0H03685g		-1,30	4,97E-04	*CBF5*	Pseudouridine synthase catalytic subunit of box H/ACA small nucleolar ribonucleoprotein particles (snoRNPs), acts on both large and small rRNAs and on snRNA U2; mutations in human ortholog dyskerin cause the disorder dyskeratosis congenita
CAGL0E00869g		-1,31	3,38E-04	*STM1*	Protein required for optimal translation under nutrient stress; perturbs association of Yef3p with ribosomes; involved in TOR signaling; binds G4 quadruplex and purine motif triplex nucleic acid; helps maintain telomere structure
CAGL0K06809g		-1,32	6,10E-03	*YBR220C*	Putative protein of unknown function; YBR220C is not an essential gene
CAGL0E05874g		-1,32	6,14E-04	*BMS1*	GTPase required for synthesis of 40S ribosomal subunits and for processing the 35S pre-rRNA at sites A0, A1, and A2; interacts with Rcl1p, which stimulates its GTPase and U3 snoRNA binding activities; has similarity to Tsr1p
CAGL0H04763g		-1,32	4,76E-03	*NPL3*	RNA-binding protein that promotes elongation, regulates termination, and carries poly(A) mRNA from nucleus to cytoplasm; has a role in repressing translation initiation by binding eIF4G; required for pre-mRNA splicing; dissociation from mRNAs promoted by Mtr10p; phosphorylated by Sky1p in the cytoplasm
CAGL0J10010g		-1,33	9,34E-03	*GCD10*	Subunit of tRNA (1-methyladenosine) methyltransferase with Gcd14p, required for the modification of the adenine at position 58 in tRNAs, especially tRNAi-Met; first identified as a negative regulator of GCN4 expression
CAGL0F08129g	CgSDA1	-1,33	2,77E-03	*SDA1*	Highly conserved nuclear protein required for actin cytoskeleton organization and passage through Start, plays a critical role in G1 events, binds Nap1p, also involved in 60S ribosome biogenesis
CAGL0J09790g		-1,34	2,40E-03	*GGC1*	Mitochondrial GTP/GDP transporter, essential for mitochondrial genome maintenance; has a role in mitochondrial iron transport; member of the mitochondrial carrier family
CAGL0K09614g		-1,34	2,05E-03	*KRE33*	Essential protein, required for biogenesis of the small ribosomal subunit; heterozygous mutant shows haploinsufficiency in K1 killer toxin resistance
CAGL0M11110g		-1,34	1,96E-03	*RRP1*	Essential evolutionarily conserved nucleolar protein necessary for biogenesis of 60S ribosomal subunits and processing of pre-rRNAs to mature rRNAs, associated with several distinct 66S pre-ribosomal particles
CAGL0J07898g		-1,35	2,28E-04	*GIS2*	Translational activator for mRNAs with internal ribosome entry sites; associates with polysomes and binds to a specific subset of mRNAs; ortholog of human ZNF9/CNBP, a gene involved in myotonic dystrophy type 2
CAGL0I07975g		-1,35	6,94E-04	*BRX1*	Nucleolar protein, constituent of 66S pre-ribosomal particles; depletion leads to defects in rRNA processing and a block in the assembly of large ribosomal subunits; possesses a sigma(70)-like RNA-binding motif
CAGL0H05709g		-1,36	2,02E-03	*NOG1*	Putative GTPase that associates with free 60S ribosomal subunits in the nucleolus and is required for 60S ribosomal subunit biogenesis; constituent of 66S pre-ribosomal particles; member of the ODN family of nucleolar G-proteins
CAGL0L11484g		-1,36	4,55E-04	*FPR3*	Nucleolar peptidyl-prolyl cis-trans isomerase (PPIase); FK506 binding protein; phosphorylated by casein kinase II (Cka1p-Cka2p-Ckb1p-Ckb2p) and dephosphorylated by Ptp1p
CAGL0L05500g		-1,37	2,49E-03	*ALB1*	Shuttling pre-60S factor; involved in the biogenesis of ribosomal large subunit; interacts directly with Arx1p; responsible for Tif6p recycling defects in absence of Rei1p
CAGL0E00517g		-1,40	8,64E-04	*YCR087C-A*	Putative protein of unknown function; green fluorescent protein (GFP)-fusion protein localizes to the nucleolus; YCR087C-A is not an essential gene
CAGL0J01265g		-1,41	4,10E-03	*UTP15*	Nucleolar protein, component of the small subunit (SSU) processome containing the U3 snoRNA that is involved in processing of pre-18S rRNA
CAGL0L10516g		-1,42	7,18E-04	*RPF2*	Essential protein involved in the processing of pre-rRNA and the assembly of the 60S ribosomal subunit; interacts with ribosomal protein L11; localizes predominantly to the nucleolus; constituent of 66S pre-ribosomal particles
CAGL0C05115g	CgARG1	-1,44	5,31E-04	*ARG1*	Arginosuccinate synthetase, catalyzes the formation of L-argininosuccinate from citrulline and L-aspartate in the arginine biosynthesis pathway; potential Cdc28p substrate
CAGL0L08114g		-1,46	1,49E-03	*RPS22A*	Protein component of the small (40S) ribosomal subunit; nearly identical to Rps22Bp and has similarity to E. coli S8 and rat S15a ribosomal proteins
CAGL0G03443g		-1,46	1,98E-04	*KAP123*	Karyopherin beta, mediates nuclear import of ribosomal proteins prior to assembly into ribosomes and import of histones H3 and H4; localizes to the nuclear pore, nucleus, and cytoplasm; exhibits genetic interactions with RAI1
CAGL0C03355g		-1,49	6,71E-03	*ESF2*	Essential nucleolar protein involved in pre-18S rRNA processing; binds to RNA and stimulates ATPase activity of Dbp8; involved in assembly of the small subunit (SSU) processome
CAGL0G07106g	CgAPT1	-1,51	2,44E-03	*APT1*	Adenine phosphoribosyltransferase, catalyzes the formation of AMP from adenine and 5-phosphoribosylpyrophosphate; involved in the salvage pathway of purine nucleotide biosynthesis
CAGL0A04037g		-1,54	1,37E-03	*PWP1*	Protein with WD-40 repeats involved in rRNA processing; associates with trans-acting ribosome biogenesis factors; similar to beta-transducin superfamily
CAGL0J00473g		-1,56	3,97E-04	*CIC1*	Essential protein that interacts with proteasome components and has a potential role in proteasome substrate specificity; also copurifies with 66S pre-ribosomal particles

CAGL0K01859g		-1,60	4,74E-04	*NOP1*	Nucleolar protein, component of the small subunit processome complex, which is required for processing of pre-18S rRNA; has similarity to mammalian fibrillarin
CAGL0B01122g	CgSAM1	-1,60	2,43E-04	*SAM1*	S-adenosylmethionine synthetase, catalyzes transfer of the adenosyl group of ATP to the sulfur atom of methionine; one of two differentially regulated isozymes (Sam1p and Sam2p)
CAGL0I09790g		-1,62	7,75E-05	*NOP58*	Protein involved in pre-rRNA processing, 18S rRNA synthesis, and snoRNA synthesis; component of the small subunit processome complex, which is required for processing of pre-18S rRNA
CAGL0M10197g		-1,63	1,43E-04	*MRT4*	Protein involved in mRNA turnover and ribosome assembly, localizes to the nucleolus
CAGL0F03927g		-1,66	1,09E-02	*GUA1*	GMP synthase, highly conserved enzyme that catalyzes the second step in the biosynthesis of GMP from inosine 5'-phosphate (IMP); transcription is not subject to regulation by guanine but is negatively regulated by nutrient starvation; reduction-of-function mutation gua1-G388D causes changes in cellular guanine nucleotide pools, defects in general protein synthesis, and impaired translation of GCN4 mRNA
CAGL0G07535g		-1,66	2,71E-04	*RRS1*	Essential protein that binds ribosomal protein L11; required for nuclear export of the 60S pre-ribosomal subunit during ribosome biogenesis; localizes to the nucleolus and in foci along nuclear periphery; cooperates with Ebp2p and Mps3p to mediate telomere clustering by binding Sir4p, but is not involved in telomere tethering; mouse homolog shows altered expression in Huntington's disease model mice
CAGL0H02057g		-1,74	2,13E-03	*GAR1*	Protein component of the H/ACA snoRNP pseudouridylase complex, involved in the modification and cleavage of the 18S pre-rRNA
CAGL0E03245g		-1,74	3,45E-04	*NSR1*	Nucleolar protein that binds nuclear localization sequences, required for pre-rRNA processing and ribosome biogenesis
CAGL0H09064g	CgFUR1	-1,76	2,03E-04	*FUR1*	Uracil phosphoribosyltransferase, synthesizes UMP from uracil; involved in the pyrimidine salvage pathway
CAGL0I08547g		-1,80	8,75E-04	*YER156C*	Putative protein of unknown function; interacts with Hsp82p and copurifies with Ipl1p; expression is copper responsive and downregulated in strains deleted for MAC1, a copper-responsive transcription factor; similarity to mammalian MYG1
CAGL0H05137g		-1,86	1,90E-04	*ALD6*	Cytosolic aldehyde dehydrogenase, activated by Mg2+ and utilizes NADP+ as the preferred coenzyme; required for conversion of acetaldehyde to acetate; constitutively expressed; locates to the mitochondrial outer surface upon oxidative stress
