# Supplementary material for: The CgHaa1-Regulon Mediates Response and Tolerance to Acetic Acid Stress in the Human Pathogen Candida glabrata
Source: G3 (Bethesda). 2016 Nov 4;7(1):1–18. doi: 10.1534/g3.116.034660 (PMC5217100; doi:10.1534/g3.116.034660)
Supplement: Supplementary file 8 [file 1TableS3.pdf]

Supplementary Table S3 – List of the genes found to be up-regulated by CgHaa1 in response to acetic acid (30 mM, at pH 4.0). The dataset of genes regulated by CgHaa1 under acetic acid stress was selected to include: i) genes that were up-regulated (by more than 2-fold) in response to acetic acid in the KUE100 strain, but not in the ΔCgHaa1 mutant; ii) genes whose acid-induced transcriptional activation in the DCgHaa1 background was at least two-fold lower than the one registered in cells of the parental strain. The biological function indicated was based on the information available at Candida Genome Database. Information on involvement in tolerance to acetic acid of corresponding S. cerevisiae orthologues and their eventual inclusion in the ScHaa1-dependent transcriptional regulatory network is also shown, based on the information available on the YEASTRACT database (Teixeira et al., 2014).

Genes whose acetic acid-induced transcriptional activation was fully dependent of CgHaa1

ORF	*C. glabrata* Gene	logFC (wt AC/ wt CTRL)	logFC (ΔCghaa1AC/ ΔCghaa1 CTRL)	Function	*S. cerevisiae* orthologue	*S. cerevisiae* orthologue regulated by ScHaa1?	*S. cerevisiae* homologue confers resistance to acetic acid?	*C. glabrata* promoter harbors an HRE motif
CAGL0C04323g	-	5,33	-	Ortholog(s) have alpha,alpha-trehalase activity, role in trehalose catabolic process and cytoplasm localization	*NTH1*	Not described	No	No
CAGL0I07249g	-	4,71	-	Putative GTPase-activating protein involved in cell wall and cytoskeleton homeostasis; gene is upregulated in azole-resistant strain	*BAG7*	Not described	No	Yes
CAGL0K07337g	-	4,37	-	Has domain(s) with predicted ion channel activity, role in ion transport and membrane localization	*HSP30*	Yes	No	Yes
CAGL0H02585g	-	4,19	-	Ortholog(s) have glutamate decarboxylase activity, role in cellular response to oxidative stress, glutamate catabolic process and cytoplasm localization	*GAD1*	Yes	No	Yes
CAGL0H10076g	-	4,12	-	Has domain(s) with predicted ion channel activity, role in ion transport and membrane localization	*YRO2*	Yes	Yes	Yes
CAGL0G06182g	-	3,72	-	No Description Available	*YHR131C*	Not described	No	No
CAGL0A01804g	*CgHXT1*	3,65	-	Ortholog(s) have fructose transmembrane transporter activity, pentose transmembrane transporter activity, role in glucose transport, mannose transport and plasma membrane localization	*HXT1*	Not described	No	No
CAGL0K03421g	-	3,39	-	Ortholog(s) have cytosol, nucleus localization	*PGM2*	Yes	No	Yes
CAGL0L08008g	-	3,39	-	No Description Available	*PMP2*	Not described	Yes	No
CAGL0G05269g	-	3,27	-	Putative mitochondrial protein; gene is downregulated in azole-resistant strain	*FMP16*	Not described	No	No
CAGL0I09702g	-	3,24	-	Ortholog(s) have riboflavin transporter activity, role in riboflavin transport and plasma membrane localization	*MCH5*	Yes	Yes	No
CAGL0E05148g	*CgDLD1*	3,23	-	Ortholog(s) have alpha-mannosidase activity, role in oligosaccharide catabolic process and cytosol, fungal-type vacuole membrane localization	*AMS1*	Not described	No	No
CAGL0G02057g	-	3,19	-	Ortholog(s) have cytoplasm, nucleus localization	*YKR075C*	Yes	No	Yes
CAGL0H04851g	-	3,18	-	Ortholog(s) have 4-nitrophenylphosphatase activity, protein serine/threonine phosphatase activity and role in cellular protein localization, cellular sodium ion homeostasis, protein dephosphorylation	*PPZ1*	Not described	No	No
CAGL0A02002g	-	3,16	-	No Description Available	*YOL024W*	Not described	No	No
CAGL0K07590g	*CgMYO3*	3,11	-	Putative myosin	*MYO3*	Not described	No	No
CAGL0I05148g	*CgDLD1*	3,07	-	D-lactate ferricytochrome C oxidoreductase	*DLD1*	Not described	No	No
CAGL0G02563g	-	3,06	-	Has domain(s) with predicted ubiquitin thiolesterase activity and role in ubiquitin-dependent protein catabolic process	-	-	-	No
CAGL0G03179g	-	3,06	-	Has domain(s) with predicted phospholipid binding activity	**ASK10*	Not described	Not tested	Yes
CAGL0A00495g	*CgPMA1*	2,98	-	Putative plasma membrane proton pump with a predicted role in pH homeostasis	**PMA1*	Not described	Not tested	Yes
CAGL0A01870g	-	2,97	-	Has domain(s) with predicted integral to membrane localization	-	-	-	No
CAGL0I06644g	-	2,94	-	Putative GPI-linked cell wall protein	*SPI1*	Not described	No	No
CAGL0M06897g	-	2,91	-	Ortholog(s) have cytoplasm localization	*YNL024C*	Yes	No	Yes
CAGL0H07469g	-	2,90	-	Putative adhesin-like protein	*ICS2*	Not described	No	No
CAGL0G05698g	*CgGDH2*	2,89	-	Ortholog(s) have glutamate dehydrogenase (NAD+) activity, role in nitrogen compound metabolic process and cytosol, mitochondrion localization	*GDH2*	Not described	No	Yes
CAGL0A01716g	-	2,87	-	Ortholog(s) have nicotinamidase activity, role in chromatin silencing at rDNA, chromatin silencing at telomere, replicative cell aging and cytosol, nucleus, peroxisome localization	*PNC1*	Not described	No	No
CAGL0E03630g	-	2,84	-	Ortholog(s) have RNA binding activity and role in negative regulation of conjugation with cellular fusion, premeiotic DNA replication, reciprocal meiotic recombination, sporulation resulting in formation of a cellular spore	*RIM4*	Not described	No	No
CAGL0J11462g	-	2,84	-	Predicted GPI-linked cell wall protein	*YNL190W*	Not described	No	Yes
CAGL0G03267g	-	2,80	-	Ortholog(s) have role in protein targeting to membrane and cytoplasm localization	*AST2*	Not described	No	No
CAGL0A01650g	-	2,80	-	Putative protein; gene is upregulated in azole-resistant strain	*ECL1*	Yes	No	Yes

CAGL0E01749g	*CgYPS4*	2,78	-	Putative aspartic protease; member of a YPS gene cluster that is required for virulence in mice; induced in response to low pH and high temperature	*YPS1*	Not described	No	No
CAGL0K02145g	-	2,71	-	Has domain(s) with predicted nucleic acid binding activity	*YER130C*	Not described	No	Yes
CAGL0L05786g	-	2,71	-	Ortholog(s) have sequence-specific DNA binding activity	*YPR013C*	Not described	No	No
CAGL0F03707g	-	2,71	-	Ortholog(s) have protein kinase activity, role in cellular ion homeostasis, protein phosphorylation and cytoplasm localization	*HRK1*	Yes	Yes	Yes
CAGL0D03322g	-	2,70	-	Ortholog(s) have role in cellular zinc ion homeostasis and endoplasmic reticulum localization	*IZH3*	Yes	No	Yes
CAGL0G09603g	-	2,69	-	Putative protein; gene is upregulated in azole-resistant strain	*YOR186W*	-	No	Yes
CAGL0G01474g	-	2,66	-	Ortholog(s) have role in cellular response to methylmercury and cytoplasm, nucleus localization	*BOP3*	Not described	No	Yes
CAGL0J03080g	-	2,65	-	No Description Available	*RGI1*	Yes	No	Yes
CAGL0L08030g	-	2,64	-	Ortholog(s) have asparagine-tRNA ligase activity, role in mitochondrial asparaginyl-tRNA aminoacylation and mitochondrion localization	*SLM5*	Not described	No	No
CAGL0F07117g	-	2,63	-	Putative subunit of the heterotrimeric G protein; gene is upregulated in azole-resistant strain	*GPG1*	Yes	No	Yes
CAGL0L03696g	-	2,62	-	Has domain(s) with predicted role in transmembrane transport and integral to membrane localization	*ECM3*	Not described	No	Yes
CAGL0G04081g	-	2,60	-	Ortholog(s) have Golgi apparatus, cell division site, cell tip, endoplasmic reticulum localization	*THI73*	Not described	No	Yes
CAGL0C03740g	-	2,59	-	Ortholog(s) have sequence-specific DNA binding, sequence-specific DNA binding transcription factor activity	*MIT1*	Not described	Yes	Yes
CAGL0J04026g	-	2,56	-	No Description Available	*HER1*	Not described	No	No
CAGL0I04246g	-	2,56	-	Putative transcription factor involved in sterol uptake; gene is upregulated in azole-resistant strain	*SUT2*	Not described	No	No
CAGL0H08778g	-	2,55	-	Ortholog(s) have mRNA binding activity, role in nuclear-transcribed mRNA catabolic process, deadenylation-dependent decay and cytoplasm localization	*PUF2*	Not described	No	Yes
CAGL0K09372g	-	2,47	-	Ortholog(s) have RNA polymerase II core promoter proximal region sequence-specific DNA binding, more	*MIG2*	Not described	No	Yes
CAGL0J00715g	-	2,46	-	Has domain(s) with predicted GTP binding, GTPase activity, role in GTP catabolic process, protein transport, signal transduction, small GTPase mediated signal transduction and intracellular, membrane localization	*YHR022C*	Not described	No	Yes
CAGL0I05060g	-	2,43	-	Has domain(s) with predicted DNA binding, chromatin binding activity	*DOT6*	Not described	No	No
CAGL0D05566g	-	2,42	-	Ortholog(s) have sterol esterase activity, role in cell wall mannoprotein biosynthetic process, sterol metabolic process and integral to membrane, plasma membrane localization	*YEH2*	Not described	Yes	Yes
CAGL0K06853g	-	2,38	-	Ortholog(s) have mRNA binding activity, role in cellular response to cadmium ion, detoxification of cadmium ion and cytosol, peroxisomal matrix, peroxisomal membrane localization	*PCS60*	Not described	No	No
CAGL0H02519g	-	2,37	-	Ortholog(s) have cytoplasm localization	*YMR253C*	Not described	No	Yes
CAGL0M00748g	*CgECM7*	2,36	-	Putative integral membrane protein required for high-affinity Ca2+ influx	*ECM7*	Not described	No	Yes
CAGL0B01875g	-	2,33	-	Ortholog(s) have mitochondrial inner membrane localization	*COX26*	Not described	No	Yes
CAGL0F08217g	-	2,30	-	Ortholog(s) have cytoplasmic stress granule localization	*YGR250C*	Yes	No	No
CAGL0J06028g	*CgMEP2*	2,27	-	Ortholog(s) have high affinity secondary active ammonium transmembrane transporter activity, methylammonium transmembrane transporter activity	*MEP2*	Not described	No	Yes
CAGL0D04026g	-	2,27	-	Ortholog(s) have 4-aminobutyrate transaminase activity, pyridoxal phosphate binding activity, role in gamma-aminobutyric acid catabolic process, glutamate metabolic process and cytosol localization	*UGA1*	Not described	No	No
CAGL0K07458g	-	2,20	-	Has domain(s) with predicted ATP binding, protein kinase activity, protein serine/threonine kinase activity, protein tyrosine kinase activity, transferase activity, transferring phosphorus-containing groups activity	*YPK1*	Not described	No	Yes
CAGL0K02651g	-	2,19	-	Ortholog(s) have role in chloride transport and fungal-type vacuole localization	*YHL008C*	Not described	No	Yes
CAGL0H08888g	-	2,18	-	Ortholog(s) have FAD transmembrane transporter activity, role in FAD transport, fungal-type cell wall biogenesis, protein folding and cellular bud neck, endoplasmic reticulum, fungal-type vacuole localization	*FLC3*	Not described		Yes
CAGL0F03641g	-	2,18	-	Has domain(s) with predicted membrane localization	*NA*	-	-	Yes
CAGL0I09108g	-	2,18	-	Has domain(s) with predicted role in transmembrane transport and integral to membrane localization	*NA*	-	-	Yes
CAGL0C05071g	-	2,15	-	Ortholog(s) have hydroxymethylpyrimidine kinase activity, phosphomethylpyrimidine kinase activity, thiaminase activity, role in thiamine biosynthetic process, thiamine catabolic process and cytosol localization	*THI20*	Yes	No	No

CAGL0K12144g	*CgFES1*	2,13	-	Putative Hsp70p nucleotide exchange factor; protein abundance decreased in ace2 mutant cells	*FES1*	Not described	No	Yes
CAGL0H00110g	-	2,13	-	Adhesin-like protein with internal repeats; predicted GPI-anchor; likely a C-terminal fragment of a single ORF with CAGL0H00132g	-	-	-	Yes
CAGL0M03179g	-	2,13	-	Ortholog(s) have cytosol, nucleus localization	*YKR018C*	Not described	No	Yes
CAGL0G07183g	-	2,13	-	Ortholog(s) have cytoplasm localization	*NA*	-	-	Yes
CAGL0B00902g	*CgHIS4*	2,13	-	Phosphoribosyl-AMP cyclohydrolase; phosphoribosyl-ATP pyrophosphatase; histidinol dehydrogenase	*HIS4*	Not described	No	Yes
CAGL0K12100g	-	2,12	-	Putative coproporphyrinogen III oxidase; protein differentially expressed in azole resistant strain	**HEM13*	Not described	No	Yes
CAGL0D04422g	-	2,11	-	Ortholog(s) have endoplasmic reticulum localization	*YPL279C*	Not described	No	Yes
CAGL0I09086g	-	2,11	-	Ortholog(s) have endoplasmic reticulum, mitochondrion localization	*ESBP6*	Not described	No	Yes
CAGL0E06292g	-	2,10	-	Has domain(s) with predicted GTP binding, GTPase activity, role in GTP catabolic process, protein transport, signal transduction, small GTPase mediated signal transduction and intracellular, membrane localization	*YHR022C*	Not described	No	No
CAGL0L05236g	*CgMDH1*	2,08	-	Malate dehydrogenase	*MDH1*	Not described	No	Yes
CAGL0H08261g	-	2,05	-	No Description Available	*YOR019W*	Not described	No	Yes
CAGL0K10428g	-	2,05	-	No Description Available	*IGD1*	Yes	No	No
CAGL0L12012g	-	2,04	-	Ortholog(s) have trans-aconitate 3-methyltransferase activity and cytosol localization	*TMT1*	Not described	No	No
CAGL0L10758g	*CgPFK2*	2,03	-	Putative 6-phosphofructokinase, beta subunit; protein abundance increased in ace2 mutant cells	*PFK2*	Not described	Yes	Yes
CAGL0K07546g	*CgPMU2*	2,03	-	Putative phosphate starvation inducible acid phosphatase; contains a phosphomutase-like domain; functionally complements a S. cerevisiae pho5 mutant; transcript abundance during phosphate starvation regulated by Pho4p	-	-	-	No
CAGL0E06072g	-	2,02	-	Ortholog(s) have cytoplasm localization	*YMR181C*	-	No	Yes
CAGL0J05060g	-	1,98	-	Ortholog(s) have RNA polymerase II regulatory region sequence-specific DNA binding, sequence-specific DNA binding RNA polymerase II transcription factor activity, zinc ion binding activity	*ZAP1*	Not described	No	Yes
CAGL0E05588g	-	1,97	-	Ortholog(s) have DNA-directed DNA polymerase activity, deoxycytidyl transferase activity and role in error-free translesion synthesis, error-prone translesion synthesis	*REV1*	Not described	No	Yes
CAGL0K01155g	-	1,97	-	No Description Available	*YGR079W*	Not described	No	Yes
CAGL0M06347g	-	1,97	-	Ortholog(s) have dihydroceramidase activity, role in ceramide biosynthetic process, ceramide catabolic process and endoplasmic reticulum, fungal-type vacuole membrane localization	*YPC1*	Yes	No	Yes
CAGL0J09262g	-	1,95	-	Ortholog(s) have role in negative regulation of ATPase activity and mitochondrion localization	*STF1*	Not described	No	No
CAGL0F02167g	-	1,94	-	Ortholog(s) have DNA binding activity, role in reciprocal meiotic recombination and nuclear chromosome localization	*MSH4*	Not described	No	No
CAGL0I01276g	-	1,93	-	Ortholog(s) have cytoplasm, nucleus localization	*YHR112C*	Not described	No	No
CAGL0I07843g	*CgADH1*	1,93	-	Putative alcohol dehydrogenase isoenzyme III; increased protein abundance in azole resistant strain	*ADH1*	Not described	No	Yes
CAGL0B02475g	*CgPHO84*	1,93	-	Ortholog(s) have inorganic phosphate transmembrane transporter activity, manganese ion transmembrane transporter activity and role in manganese ion transport, phosphate ion transport, polyphosphate metabolic process	*PHO84*	Not described	No	Yes
CAGL0K07183g	-	1,92	-	Unknown	-	-	-	No
CAGL0H08305g	-	1,91	-	No Description Available	*DET1*	Not described	No	No
CAGL0I00550g	-	1,91	-	Ortholog(s) have fungal-type vacuole membrane, nucleus localization	*YLR297W*	Yes	No	Yes
CAGL0F03399g	*CgSCS7*	1,88	-	Ortholog(s) have fatty acid alpha-hydroxylase activity, role in cellular response to cadmium ion, detoxification of cadmium ion, mannosyl-inositol phosphorylceramide metabolic process and endoplasmic reticulum, membrane localization	*SCS7*	Not described	Yes	Yes
CAGL0H08195g	-	1,88	-	Ortholog(s) have ATPase inhibitor activity, Hsp70 protein binding, Hsp90 protein binding, mRNA binding activity, role in protein folding and cytosol, nucleus localization	*STI1*	Not described	No	Yes
CAGL0M03135g	-	1,87	-	Ortholog(s) have cell division site, cell tip, cytosol localization	*ALY2*	Not described	No	No
CAGL0L03267g	*CgGAP1*	1,87	-	Ortholog(s) have L-proline transmembrane transporter activity, polyamine transmembrane transporter activity and role in amino acid transport, polyamine transport, regulation of nitrogen utilization	*GAP1*	Not described	No	Yes
CAGL0C00319g	-	1,85	-	Ortholog(s) have carboxypeptidase activity, role in nitrogen compound metabolic process, proteolysis involved in cellular protein catabolic process and fungal-type vacuole lumen localization	*CPS1*	Not described	No	No
CAGL0L10582g	-	1,85	-	Ortholog(s) have cytoplasm localization	*YMR196W*	Not described	No	Yes
CAGL0F00605g	*CgGLK1*	1,85	-	Aldohexose specific glucokinase	*EMI2*	Not described	No	Yes

CAGL0K07678g	-	1,84	-	Unknown	-	-	-	Yes
CAGL0K05357g	*CgGLN1*	1,84	-	Ortholog(s) have glutamate-ammonia ligase activity, role in cellular response to nitrogen starvation, glutamine biosynthetic process, nitrogen utilization and cytosol, nucleus localization	**GLN1*	Yes	No	Yes
CAGL0E06116g	-	1,84	-	Has domain(s) with predicted nucleic acid binding activity	*RGM1*	-	No	Yes
CAGL0H04037g	-	1,84	-	Ortholog(s) have heat shock protein binding activity, role in glycogen metabolic process, meiosis, mitotic spindle assembly checkpoint, response to heat and protein phosphatase type 1 complex localization	*GAC1*	Not described	No	No
CAGL0B04675g	-	1,83	-	No Description Available	*YCL001W-B*	Not described	No	Yes
CAGL0F09207g	*CgBAT1*	1,82	-	Has domain(s) with predicted branched-chain-amino-acid transaminase activity, catalytic activity and role in branched-chain amino acid metabolic process, metabolic process	*BAT1*	Not described	No	No
CAGL0A01782g	*CgHXT4*	1,80	-	Ortholog(s) have glucose transmembrane transporter activity, pentose transmembrane transporter activity and plasma membrane localization	*HXT4*	Not described	No	Yes
CAGL0K07205g	-	1,79	-	No Description Available	*YGL117W*	Not described	No	Yes
CAGL0H02387g	-	1,78	-	Putative trehalose-6-phosphate synthase/phosphatase subunit; gene is upregulated in azole-resistant strain	*TPS3*	Not described	No	Yes
CAGL0G02101g	*CgECM4*	1,77	-	Putative omega class glutathione transferase; gene is downregulated in azole-resistant strain	*ECM4*	Not described	No	Yes
CAGL0K02101g	-	1,76	-	Ortholog(s) have role in TOR signaling cascade, cellular response to chemical stimulus, cellular response to heat, cellular response to pH and chlamydospore formation, more	*PMD1*	-	No	No
CAGL0B00264g	*CgMTLalpha2*	1,75	-	Homeobox-domain protein alpha2	*HMLalpha2*	Not described	No	No
CAGL0F09273g	-	1,75	-	Putative adhesin-like protein	-	-	.	No
CAGL0K01683g	*CgGPD1*	1,74	-	Putative cytoplasmic glycerol-3-phosphate dehydrogenase (NAD+); protein abundance increased in ace2 mutant cells	*GPD1*	Not described	No	No
CAGL0B03817g	-	1,74	-	Ortholog(s) have cytosol, nucleus localization	*MHO1*	Not described	No	No
CAGL0K05665g	-	1,73	-	Has domain(s) with predicted catalytic activity, pyridoxal phosphate binding activity	-	Not described	-	Yes
CAGL0M03245g	-	1,73	-	Ortholog(s) have cytosol localization	*YEL020C*	Not described	No	No
CAGL0M04807g	*CgSNF2*	1,73	-	Component of the chromatin remodelling Swi/Snf complex; involved in regulation of biofilm formation	*SNF2*	Not described	No	No
CAGL0G08888g	-	1,73	-	Ortholog(s) have role in maintenance of rDNA, meiotic chromosome segregation, recombinational repair and Shu complex, cytoplasm, nucleus localization	*CSM2*	Not described	No	No
CAGL0M06765g	-	1,73	-	Ortholog(s) have role in actin filament organization, endocytosis, response to stress and cytosol, nucleus localization	*SIW14*	Not described	Yes	Yes
CAGL0E05610g	-	1,72	-	Ortholog(s) have role in cellular response to nitrogen starvation, negative regulation of G0 to G1 transition and cytosol localization	*PYK2*	-	No	Yes
CAGL0J08074g	-	1,72	-	Ortholog(s) have phosphatidylinositol transporter activity	*PDR17*	Yes	No	No
CAGL0J07502g	-	1,71	-	Putative protein similar to globins with a heme-binding domain; gene is upregulated in azole-resistant strain	*YNL234W*	Not described	No	No
CAGL0C01551g	-	1,70	-	Has domain(s) with predicted DNA binding, sequence-specific DNA binding, sequence-specific DNA binding transcription factor activity and role in regulation of transcription, DNA-dependent	*CUP9*	Not described	No	Yes
CAGL0H10164g	-	1,69	-	Ortholog(s) have role in premeiotic DNA replication and cytoplasm localization	*MUM2*	Not described	No	No
CAGL0M02915g	-	1,69	-	Ortholog(s) have role in peroxisome degradation and peroxisome localization	*ATG36*	Not described	No	Yes
CAGL0H05379g	-	1,69	-	Ortholog(s) have RNA polymerase II core promoter proximal region sequence-specific DNA binding, more	**GCR1*	Yes	No	No
CAGL0L05016g	-	1,68	-	Ortholog(s) have Sin3-type complex localization	*STB2*	Not described	No	No
CAGL0B01265g	*CgMATalpha2*	1,68	-	Mating-type regulatory protein alpha2; expressed in all MTLalpha strains and not in MTLa strains	*MATalpha2*	Not described	No	No
CAGL0H10626g	*AWP13*	1,67	-	Predicted cell wall adhesin with a predicted role in adhesion; predicted GPI anchor; contains tandem repeats	-	-	-	Yes
CAGL0L06006g	-	1,67	-	Ortholog(s) have protein serine/threonine kinase activity	**ATG1*	Not described	No	No
CAGL0E04548g	-	1,67	-	Ortholog(s) have mitochondrion localization	*YOR020W-A*	Not described	No	No
CAGL0M01716g		1,66		Transcription factor targeting filamentation genes and Ty1 expression; Ste12p activation of most filamentation gene promoters depends on Tec1p and Tec1p transcriptional activity is dependent on its association with Ste12p; binds to TCS elements upstream of filamentation genes, which are regulated by Tec1p/Ste12p/Dig1p complex; competes with Dig2p for binding to Ste12p/Dig1p; positive regulator of chronological life span; TEA/ATTS DNA-binding domain family member	*TEC1*	Not described	No	Yes
CAGL0C02365g	-	1,64	-	Unknown	-	-	-	Yes

CAGL0I05698g	-	1,64	-	Ortholog(s) have 6-phosphofructokinase activity, mRNA binding activity, role in glycolysis, proton transport and 6-phosphofructokinase complex, mitochondrion localization	*PFK2*	Not described	Yes	Yes
CAGL0F02101g	-	1,64	-	No Description Available	*BLM10*	Not described	No	No
CAGL0L11990g	-	1,63	-	Ortholog(s) have disulfide oxidoreductase activity, role in actin cytoskeleton organization, cellular iron ion homeostasis, cellular response to oxidative stress and nucleus localization	*GRX4*	Not described	No	Yes
CAGL0F03003g	-	1,62	-	Ortholog(s) have osmosensor activity and role in (1->3)-beta-D-glucan biosynthetic process, cellular bud site selection, fungal-type cell wall organization, hyperosmotic response, osmosensory signaling pathway via Sho1 osmosensor	**HKR1*	Not described	No	Yes
CAGL0M10439g	-	1,61	-	Ortholog(s) have alpha,alpha-trehalase activity, calcium ion binding activity	*NTH1*	Not described	No	No
CAGL0L02453g	-	1,61	-	No Description Available	*MIT1*	Not described	Yes	No
CAGL0E03025g	-	1,60	-	No Description Available	*ECL1*	Not described	No	Yes
CAGL0H10120g	-	1,59	-	Ortholog(s) have glucan endo-1,6-beta-glucosidase activity and cytosol, mitochondrial intermembrane space, nucleus localization	*YBR056W*	Not described	No	Yes
CAGL0L03157g	-	1,58	-	Ortholog(s) have sequence-specific DNA binding, sequence-specific DNA binding RNA polymerase II transcription factor activity	*DAL80*	Not described	No	Yes
CAGL0M01364g	CgCBF3D	1,58		Evolutionarily conserved kinetochore protein that is part of multiple protein complexes, including the SCF ubiquitin ligase complex, the CBF3 complex that binds centromeric DNA, and the RAVE complex that regulates assembly of the V-ATPase	*SKP1*	Not described	No	Yes
CAGL0F00187g	-	1,57	-	Ortholog(s) have copper ion transmembrane transporter activity, iron ion transmembrane transporter activity	*FET4*	Not described	No	Yes
CAGL0C05027g	-	1,57	-	Has domain(s) with predicted transferase activity, transferring acyl groups activity	*YAT1*	Not described	No	Yes
CAGL0E01529g	-	1,56	-	Ortholog(s) have role in fructose 2,6-bisphosphate metabolic process, regulation of glycolysis	*PFK27*	Yes	No	Yes
CAGL0I09009g	-	1,55	-	Ortholog(s) have histidinol-phosphatase activity, role in histidine biosynthetic process and cytosol, nucleus localization	*HIS2*	Not described	No	No
CAGL0E05808g	*CgTHI6*	1,54	-	Bifunctional enzyme of thiamine biosynthesis, with thiamine-phosphate pyrophosphorylase and 4-methyl-5-beta-hydroxyethylthiazole kinase activities; active as a homohexamer	*THI6*	Not described	No	Yes
CAGL0I10747g	-	1,54	-	Ortholog(s) have ammonium transmembrane transporter activity, role in ammonium transport, nitrogen utilization and plasma membrane localization	*MEP3*	Not described	No	Yes
CAGL0H02541g	-	1,53	-	Ortholog(s) have mitochondrion localization	*YMR252C*	-	No	No
CAGL0L08074g	-	1,53	-	No Description Available	*SYP1*	Not described	No	No
CAGL0L10912g	-	1,51	-	Ortholog(s) have spermidine transmembrane transporter activity, spermine transmembrane transporter activity, role in spermidine transport, spermine transport and fungal-type vacuole membrane, plasma membrane localization	*TPO4*	Not described	No	Yes
CAGL0E03740g	-	1,51	-	No Description Available	*YHL026C*	Not described	No	Yes
CAGL0L03916g	-	1,50	-	Has domain(s) with predicted nucleic acid binding activity	*AZF1*	Not described	No	No
CAGL0B01595g	-	1,49	-	Unknown	-	Not described	No	Yes
CAGL0M10153g	-	1,49	-	Ortholog(s) have MAP kinase kinase kinase kinase activity, histone serine kinase activity	*STE20*	Not described	No	No
CAGL0F02519g	-	1,48	-	Has domain(s) with predicted DNA binding, sequence-specific DNA binding RNA polymerase II transcription factor activity, zinc ion binding activity and role in regulation of transcription, DNA-dependent, transcription, DNA-dependent	*YJL206C*	Not described	No	No
CAGL0B04895g	-	1,48	-	Ortholog(s) have RNA polymerase II core promoter proximal region sequence-specific DNA binding activity	*RFX1*	Not described	No	No
CAGL0K12078g	-	1,48	-	Has domain(s) with predicted nucleic acid binding activity	*NRG1*	Yes	Yes	No
CAGL0L09273g	-	1,48	-	Ortholog(s) have methylisocitrate lyase activity, role in propionate catabolic process, 2-methylcitrate cycle and mitochondrial matrix localization	*ICL2*	Not described	No	No
CAGL0A02145g	-	1,47	-	Ortholog(s) have actin filament binding activity, role in actin cortical patch localization, actin filament bundle assembly, endocytosis and actin cortical patch localization	*YSC84*	Not described	No	No
CAGL0C04213g	-	1,47	-	Ortholog(s) have role in vesicle-mediated transport and fungal-type vacuole, vesicle localization	*RCR1*	Not described	No	No
CAGL0K10824g	-	1,47	-	No Description Available	*YLR149C*	Not described	No	Yes
CAGL0H07997g	*CgKNH1*	1,46	-	Protein involved in cell wall beta 1,6-glucan synthesis, similar to Kre9p,	*KNH1*	Not described	No	No
CAGL0H09966g	-	1,46	-	Ortholog(s) have mitochondrion localization	*FMP23*	Not described	No	No

CAGL0I04180g	*CgAMT1*	1,46	-	Metal-activated transcription factor; binds promoters of metallothionein genes; autoregulates its own expression; gene is downregulated in azole-resistant strain	**CUP2*	Not described	No	Yes
CAGL0E04004g	-	1,46	-	Ortholog(s) have L-methionine transmembrane transporter activity and role in amino acid transport	*MUP3*	Not described	No	Yes
CAGL0M03157g	-	1,46	-	Ortholog(s) have role in autophagy, cellular response to starvation, chromatin silencing at rDNA, fungal-type cell wall organization, inositol lipid-mediated signaling and mitochondrion, pre-autophagosomal structure localization	*IRS4*	Not described	No	No
CAGL0D02948g	*CgKAR2*	1,46	-	Protein with a predicted role in nuclear fusion	**KAR2*	Not described	No	Yes
CAGL0H10054g	-	1,46	-	No Description Available	*YBR053C*	Not described	No	Yes
CAGL0E00319g		1,46		Unknown	-	Not described	-	No
CAGL0B02431g	-	1,45	-	Ortholog(s) have NADH dehydrogenase (ubiquinone) activity and role in NADH oxidation, chronological cell aging, mitochondrial electron transport, NADH to ubiquinone, positive regulation of apoptotic process	*NDI1*	Not described	No	Yes
CAGL0K08624g	-	1,45	-	Ortholog(s) have RNA polymerase II activating transcription factor binding, more	*HAP4*	Not described	No	Yes
CAGL0M09449g	-	1,44	-	No Description Available	*ECM21*	Not described	No	No
CAGL0I05522g	-	1,43	-	Has domain(s) with predicted ubiquitin thiolesterase activity and role in ubiquitin-dependent protein catabolic process	*UBP9*	Not described	No	Yes
CAGL0G09295g	-	1,43	-	Ortholog(s) have cytoplasm localization	*FYV8*	Not described	No	No
CAGL0I02530g	-	1,43	-	Ortholog(s) have N,N-dimethylaniline monooxygenase activity, role in protein folding and endoplasmic reticulum membrane, mitochondrion localization	*FMO1*	Not described	No	No
CAGL0G09515g	-	1,42	-	Ortholog(s) have glucan exo-1,3-beta-glucosidase activity, role in ascospore formation and ascospore wall localization	*SPR1*	Not described	No	No
CAGL0C03267g	*CgFPS1*	1,42	-	Glycerol transporter; 1 of 2 Fps1 orthologs in C. glabrata; double fps1/fps2 mutant accumulates glycerol, has constitutive cell wall stress, is hypersensitive to caspofungin in vitro and in vivo	*FPS1*	Not described	Yes	Yes
CAGL0A03872g	-	1,42	-	Ortholog(s) have phosphatidylinositol-4,5-bisphosphate binding activity, role in actin cortical patch assembly, actin filament organization, endocytosis and actin cortical patch, mating projection tip localization	*ENT2*	Not described	No	No
CAGL0M07612g	-	1,41	-	Ortholog(s) have polyamine oxidase activity, role in pantothenate biosynthetic process, polyamine catabolic process and cytoplasm localization	*FMS1*	Not described	No	Yes
CAGL0L03135g	-	1,41	-	Putative phospholipase D; gene is upregulated in azole-resistant strain	*SPO14*	Not described	No	Yes
CAGL0M06963g	-	1,41	-	Ortholog(s) have role in tRNA export from nucleus and cytoplasm, nucleus localization	*SOL1*	Not described	No	Yes
CAGL0H04213g	-	1,41	-	Ortholog(s) have sequence-specific DNA binding activity and nucleus localization	*TDA9*	Not described	No	No
CAGL0F01793g	*CgERG3*	1,41	-	Delta 5,6 sterol desaturase; C-5 sterol desaturase; predicted transmembrane domain and endolasmic reticulum (ER) binding motif; gene used for molecular typing of C. glabrata strain isolates	*ERG3*	Not described	Yes	No
CAGL0L00583g	-	1,40	-	Ortholog(s) have sequence-specific DNA binding, sequence-specific DNA binding transcription factor activity	*USV1*	Not described	No	Yes
CAGL0K01705g	-	1,39	-	Ortholog(s) have cytoplasm localization	*GPM2*	Not described	No	No
CAGL0F04829g	-	1,39	-	Ortholog(s) have role in negative regulation of Arp2/3 complex-mediated actin nucleation and actin cortical patch, nucleus localization	*PIN3*	Not described	No	Yes
CAGL0H02893g	-	1,39	-	Ortholog(s) have mitochondrion localization	*YJL070C*	Not described	No	No
CAGL0G05357g	-	1,39	-	Ortholog(s) have NADHX epimerase activity, role in nicotinamide nucleotide metabolic process and cytosol, mitochondrion, nucleus localization	*YNL200C*	Yes	Yes	No
CAGL0D04510g	-	1,39	-	No Description Available	*YPR117W*	Not described	No	Yes
CAGL0I07227g	-	1,38	-	Putative isocitrate dehydrogenase	*IDH2*	Not described	No	Yes
CAGL0G06798g	-	1,38	-	No Description Available	*YJR005C-A*	Not described	No	Yes
CAGL0M04763g	-	1,37	-	Ortholog(s) have role in cellular response to drug and cytosol, nucleus localization	*YOR289W*	Yes	No	Yes
CAGL0M10571g	-	1,37	-	Ortholog(s) have ergosterol O-acyltransferase activity, role in ergosterol metabolic process and endoplasmic reticulum localization	*ARE2*	Not described	No	Yes
CAGL0D01980g	-	1,37	-	Ortholog(s) have sterol esterase activity, role in cellular lipid metabolic process, sterol metabolic process and integral to membrane, lipid particle localization	*TGL1*	Not described	No	No
CAGL0G05962g	-	1,37	-	Ortholog(s) have endoplasmic reticulum localization	*YHR140W*	Not described	No	Yes
CAGL0B03421g	-	1,37	-	Has domain(s) with predicted DNA binding, sequence-specific DNA binding RNA polymerase II transcription factor activity, zinc ion binding activity and role in regulation of transcription, DNA-dependent, transcription, DNA-dependent	*HAP1*	Not described	No	Yes

CAGL0C03872g	*CgTIR3*	1,36	-	Putative GPI-linked cell wall protein involved in sterol uptake	*TIR3*	Not described	No	Yes
CAGL0C03289g	*CgYBT1*	1,35	-	Putative ABC transporter involved in bile acid transport; gene is upregulated in azole-resistant strain	*YBT1*	Not described	No	Yes
CAGL0E02651g	-	1,35	-	Has domain(s) with predicted identical protein binding, serine-type endopeptidase activity and role in negative regulation of catalytic activity, proteolysis	*YSP3*	Not described	No	No
CAGL0C01595g	-	1,35	-	Ortholog(s) have imidazoleglycerol-phosphate synthase activity, role in histidine biosynthetic process and cytosol, nucleus localization	*HIS7*	Not described	No	No
CAGL0F04785g		1,35		Cytoplasmic protein of unknown function; identified as a high-copy suppressor of the synthetic lethality of a sis2 sit4 double mutant, suggesting a role in G1/S phase progression; similar to Mlf3p	*VHS2*	Not described	No	Yes
CAGL0J07876g	-	1,34	-	Ortholog(s) have cytoplasm, nucleus localization	*RTC4*	Yes	No	No
CAGL0M12947g	*CgPUP1*	1,34	-	Mitochondria-localized protein; gene is upregulated in azole-resistant strain	-	-	-	No
CAGL0G06050g	-	1,34	-	Unknown	-	-	-	Yes
CAGL0L04598g	-	1,33	-	Ortholog(s) have RNA 7-methylguanosine cap binding, m7G(5')pppN diphosphatase activity, role in deadenylation-dependent decapping of nuclear-transcribed mRNA and cytoplasmic mRNA processing body, cytosol, nucleus localization	*DCS2*	Not described	No	Yes
CAGL0J02948g	*CgFCY2*	1,33	-	Purine-cytosine transporter	*FCY22*	Not described	No	Yes
CAGL0G00858g	-	1,33	-	No Description Available	*MID2*	Not described	No	Yes
CAGL0L12892g	-	1,33	-	Ortholog(s) have role in actin cytoskeleton organization, inositol lipid-mediated signaling, vacuole organization and plasma membrane localization	*SFK1*	Not described	No	No
CAGL0L09251g	-	1,32	-	Ortholog(s) have role in positive regulation of transcription from RNA polymerase II promoter, response to salt stress and cytoplasm localization	*HAL1*	Not described	No	Yes
CAGL0K11297g	-	1,31	-	Ortholog(s) have gluconokinase activity, role in D-gluconate metabolic process and cytosol, nucleus localization	*YDR248C*	Not described	No	Yes
CAGL0L07744g	-	1,31	-	Ortholog(s) have endoplasmic reticulum localization	*ADP1*	Not described	No	Yes
CAGL0I05676g		1,30		RNA-dependent ATPase in the DEAH-box family, required for activation of the spliceosome before the first transesterification step in RNA splicing; orthologous to human protein DHX16	*PRP2*	Yes	No	Yes
CAGL0J08613g	-	1,29	-	Ortholog(s) have calcium activated cation channel activity, calcium channel activity, potassium channel activity, sodium channel activity, voltage-gated ion channel activity and role in cellular cation homeostasis	*YVC1*	Not described	No	Yes
CAGL0L01287g	*CgUTR4*	1,29	-	Haloacid dehalogenase-like hydrolase	*UTR4*	Not described	No	No
CAGL0L10043g	-	1,29	-	Ortholog(s) have protein kinase activator activity	*STD1*	Yes	No	Yes
CAGL0H09944g	-	1,29	-	Ortholog(s) have 2-alkenal reductase [NAD(P)] activity, AU-rich element binding, NADPH:quinone reductase activity, role in cellular response to oxidative stress and cytosol, nucleus localization	*ZTA1*	Not described	No	No
CAGL0G01100g	-	1,29	-	Ortholog(s) have cytosol localization	*YLR345W*	Not described	No	Yes
CAGL0C01771g	-	1,29	-	Ortholog(s) have role in protein targeting to vacuole and mitochondrion localization	*YBR241C*	Not described	Yes	No
CAGL0H03135g	MFalpha	1,29		Mating pheromone alpha-factor, made by alpha cells; interacts with mating type a cells to induce cell cycle arrest and other responses leading to mating; also encoded by MF(ALPHA)1, which is more highly expressed than MF(ALPHA)2	*MF(ALPHA)2*	Not described	No	No
CAGL0J11308g	-	1,28	-	Has domain(s) with predicted ATP binding, protein kinase activity, protein tyrosine kinase activity, transferase activity, transferring phosphorus-containing groups activity and role in protein phosphorylation	*NPR1*	Not described	No	Yes
CAGL0I10648g	*CgASN2*	1,28	-	Putative asparagine synthetase; protein abundance increased in ace2 mutant cells	*ASN2*	Not described	No	No
CAGL0J01699g	-	1,28	-	No Description Available	*YPR010C-A*	Not described	No	Yes
CAGL0A01045g		1,28		Fumarase, converts fumaric acid to L-malic acid in the TCA cycle; cytosolic and mitochondrial distribution determined by the N-terminal targeting sequence, protein conformation, and status of glyoxylate shunt; phosphorylated in mitochondria	*FUM1*	Not described	Yes	Yes
CAGL0K00803g	CgTRX2	1,28		Cytoplasmic thioredoxin isoenzyme of the thioredoxin system which protects cells against oxidative and reductive stress, forms LMA1 complex with Pbi2p, acts as a cofactor for Tsa1p, required for ER-Golgi transport and vacuole inheritance	*TRX1*	Yes	No	Yes

CAGL0F04609g	-	1,27	-	Ortholog(s) have ubiquitin binding activity, role in endocytosis, endoplasmic reticulum unfolded protein response and actin cortical patch, cellular bud neck, cellular bud tip, mating projection tip localization	*EDE1*	Not described	No	No
CAGL0L05654g	-	1,27	-	Ortholog(s) have chloride transmembrane transporter activity, potassium ion transmembrane transporter activity	*TRK2*	Not described	No	Yes
CAGL0C05533g	-	1,27	-	Has domain(s) with predicted phosphoric diester hydrolase activity and role in lipid metabolic process	*AIM6*	Not described	No	Yes
CAGL0M07293g	-	1,27	-	Putative ABC transporter of weak organic acids; gene is downregulated in azole-resistant strain	*PDR12*	Yes	No	No
CAGL0M11726g		1,27		Putative GPI-linked cell wall adhesin-like protein;	*CCW12*	Yes	No	Yes
CAGL0E02321g	-	1,26	-	Putative phospholipase B; predicted GPI-anchor	*PLB3*	Yes	No	No
CAGL0F05687g	-	1,26	-	Ortholog(s) have cytoplasm, ribosome localization	*YDR186C*	Yes	No	No
CAGL0I05082g	-	1,26	-	Ortholog(s) have role in DNA damage checkpoint, G2/M transition of mitotic cell cycle, cellular response to biotic stimulus and cellular response to starvation, more	*PIN4*	Yes	No	No
CAGL0C04191g		1,25		Succinate semialdehyde dehydrogenase involved in the utilization of gamma-aminobutyrate (GABA) as a nitrogen source; part of the 4-aminobutyrate and glutamate degradation pathways; localized to the cytoplasm	*UGA2*	Not described	No	Yes
CAGL0I11011g		1,25		Putative adhesin	-	Not described	-	No
CAGL0C01397g	-	1,24	-	Has domain(s) with predicted 6-phosphofructo-2-kinase activity, ATP binding, catalytic activity and role in fructose 2,6-bisphosphate metabolic process, fructose metabolic process	*PFK26*	Not described	No	Yes
CAGL0J00803g	-	1,24	-	Ortholog(s) have structural constituent of cytoskeleton activity	*MHP1*	Not described	No	Yes
CAGL0L06864g	-	1,24	-	Putative protein related to ECM3; gene is downregulated in azole-resistant strain	*SIP5*	Yes	No	No
CAGL0I03872g	-	1,24	-	Has domain(s) with predicted substrate-specific transmembrane transporter activity, transmembrane transporter activity, role in transmembrane transport and integral to membrane, membrane localization	*RGT2*	Not described	No	No
CAGL0L06248g	-	1,24	-	Ortholog(s) have cytoplasm, nucleus localization	*YBR085C-A*	Not described	No	Yes
CAGL0J03256g	-	1,24	-	Ortholog(s) have protein tyrosine phosphatase activity	*PTP3*	Not described	No	Yes
CAGL0K10604g		1,24		Calmodulin-dependent protein kinase; may play a role in stress response, many Ca++/calmodulin dependent phosphorylation substrates demonstrated in vitro, amino acid sequence similar to Cmk2p and mammalian Cam Kinase II	*CMK1*	Not described	No	Yes
CAGL0K01133g	-	1,23	-	Ortholog(s) have actin monomer binding activity, role in sequestering of actin monomers and cell division site, cell tip, mating projection tip localization	*TWF1*	Not described	No	Yes
CAGL0K04257g	-	1,23	-	Ortholog(s) have ATPase activity, coupled to transmembrane movement of ions, RNA polymerase II core promoter proximal region sequence-specific DNA binding, sequence-specific DNA binding RNA polymerase II transcription factor activity	*RME1*	Not described	No	Yes
CAGL0D00990g		1,23		Putative protein of unknown function; YDL057W is not an essential gene	*YDL057W*	Not described	No	Yes
CAGL0H09614g		1,23		Cell wall mannoprotein of the Srp1p/Tip1p family of serine-alanine-rich proteins; expression is downregulated at acidic pH and induced by cold shock and anaerobiosis; abundance is increased in cells cultured without shaking	*TIR1*	Not described	No	No
CAGL0L09108g	-	1,22	-	Ortholog(s) have role in propionate metabolic process and mitochondrial outer membrane localization	*PDH1*	Yes	No	No
CAGL0E06666g	*CgEPA2*	1,22	-	Epithelial adhesion protein; predicted GPI-anchor; adhesin-like protein	*FLO1*	Yes	-	Yes
CAGL0H02695g	-	1,21	-	Ortholog(s) have glycogenin glucosyltransferase activity and role in glycogen biosynthetic process	*GLG1*	Not described	No	Yes
CAGL0D01100g	-	1,21	-	Ortholog(s) have 6-phosphofructo-2-kinase activity and role in fructose 2,6-bisphosphate metabolic process	*PFK26*	Not described	No	No
CAGL0H09592g	-	1,21	-	Putative GPI-linked cell wall protein	-	-	-	Yes
CAGL0F01947g	*CgIRC15*	1,21	-	Ortholog(s) have microtubule binding activity	*IRC15*	Not described	No	No
CAGL0I03476g		1,21		Cytoplasmic DExD/H-box helicase, stimulates mRNA decapping; coordinates distinct steps in mRNA function and decay, interacts with both the decapping and deadenylase complexes, may have a role in mRNA export and translation; C-terminus of Dhh1p interacts with Ngr1p and promotes POR1, but not EDC1 mRNA decay	*DHH1*	Not described	No	Yes
CAGL0I08943g		1,21		Poly(A) binding protein, suppressor of DNA polymerase epsilon mutation, similar to Mip6p	*PES4*	Not described	No	Yes

CAGL0M09108g	-	1,20	-	Has domain(s) with predicted RNA binding, nucleic acid binding, nucleotide binding activity	*PUF2*	Not described	No	Yes
CAGL0I01980g	-	1,20	-	Putative activator of transcription; gene is upregulated in azole-resistant strain	*YSP1*	Not described	Yes	Yes
CAGL0K12716g	-	1,20	-	Has domain(s) with predicted substrate-specific transmembrane transporter activity, transmembrane transporter activity, role in transmembrane transport and integral to membrane, membrane localization	*YFL040W*	Not described	No	No
CAGL0H01177g	-	1,20	-	Ortholog(s) have diacylglycerol diphosphate phosphatase activity, phosphatidate phosphatase activity and role in farnesol biosynthetic process, phospholipid metabolic process	*DPP1*	Not described	No	Yes
CAGL0L06358g		1,20		Vacuolar membrane protein of unknown function that is conserved in mammals; predicted to contain eleven transmembrane helices; interacts with Pdr5p, a protein involved in multidrug resistance	*TMS1*	Not described	No	No
CAGL0M05401g		1,20		Putative protein of unknown function	*YBR201C-A*	Not described	No	Yes
CAGL0E01859g	*CgYPS10*	1,19	-	Putative aspartic protease; predicted GPI-anchor; member of a YPS gene cluster that is required for virulence in mice; induced in response to low pH and high temperature	-	-	-	Yes
CAGL0L12056g	*CgBMH1(A)*	1,19	-	No Description Available	*BMH1*	Not described	No	Yes
CAGL0K04675g	-	1,19	-	Unknown	-	-	-	No
CAGL0L10142g	*CgRSB1*	1,18	-	Putative sphingolipid flippase; gene is upregulated in azole-resistant strain	*RSB1*	Not described	No	Yes
CAGL0H01287g	-	1,18	-	Ortholog(s) have mRNA 5'-UTR binding, translation repressor activity, nucleic acid binding activity	**SSD1*	Not described	No	Yes
CAGL0E01419g	*CgYPS2*	1,18	-	Putative aspartic protease; predicted GPI-anchor; member of a YPS gene cluster that is required for virulence in mice; induced in response to low pH and high temperature	*MCK7*	-	-	Yes
CAGL0J01892g	*CgPAN1*	1,17	-	Ortholog(s) have protein binding, bridging activity and role in actin cortical patch assembly, axial cellular bud site selection, bipolar cellular bud site selection, endocytosis, unidimensional cell growth	**PAN1*	Not described	No	Yes
CAGL0L09691g	-	1,17	-	Ortholog(s) have RNA polymerase II core promoter proximal region sequence-specific DNA binding, more	*PUT3*	Not described	No	Yes
CAGL0M08800g	*CgYAP6*	1,17	-	bZIP domain-containing protein	*CIN5*	Not described	No	No
CAGL0L06094g	CgSTR3	1,17		Peroxisomal cystathionine beta-lyase, converts cystathionine into homocysteine; may be redox regulated by Gto1p	*STR3*	Not described	No	Yes
CAGL0M06457g		1,17		Putative protein of unknown function; expression is reduced in a gcr1 null mutant; GFP-fusion protein localizes to the vacuole; expression pattern and physical interactions suggest a possible role in ribosome biogenesis	*GDT1*	Not described	Yes	Yes
CAGL0E06380g	-	1,16	-	Ortholog(s) have ATP-dependent NAD(P)H-hydrate dehydratase activity, role in nicotinamide nucleotide metabolic process and cytosol localization	*YKL151C*	Yes	No	Yes
CAGL0K12760g	-	1,16	-	Ortholog(s) have cytoplasm localization	*YFL042C*	Not described	No	Yes
CAGL0C03850g		1,16		Nuclear thiol peroxidase which functions as an alkyl-hydroperoxide reductase during post-diauxic growth	*DOT5*	Not described	Yes	No
CAGL0E02255g		1,16		Peripheral membrane protein of the plasma membrane that interacts with Mid2p; regulates the cell integrity pathway mediated by Pkc1p and Slt2p; the authentic protein is detected in a phosphorylated state in highly purified mitochondria	*ZEO1*	Not described	No	No
CAGL0G05093g	-	1,15	-	Ortholog(s) have mitochondrion localization	*YDR061W*	Not described	No	No
CAGL0C05137g	CgGPD2	1,15		NAD-dependent glycerol 3-phosphate dehydrogenase, homolog of Gpd1p, expression is controlled by an oxygen-independent signaling pathway required to regulate metabolism under anoxic conditions; located in cytosol and mitochondria	*GPD2*	Not described	No	Yes
CAGL0H02497g		1,15		Coiled-coiled protein of unknown function, identified as a high-copy suppressor of a dbp5 mutation	*GFD1*	Not described	No	Yes
CAGL0L08140g		1,15		PProtein homologous to human Chediak-Higashi syndrome and murine Beige proteins, which are implicated in disease syndromes due to defective lysosomal trafficking; mutant phenotype and genetic interactions suggest a role in protein sorting	*BPH1*	Not described	Yes	Yes
CAGL0I03168g	-	1,14	-	No Description Available	*YEL023C*	Not described	No	No
CAGL0D01474g	-	1,14	-	Ortholog(s) have role in actin cortical patch assembly and actin cortical patch, membrane raft localization	*AIM3*	Not described	No	No

CAGL0A00539g	-	1,13	-	Ortholog(s) have role in CVT pathway, intra-Golgi vesicle-mediated transport and Golgi transport complex, mitochondrion localization	*COG7*	Not described	No	No
CAGL0I07887g	-	1,13	-	Ortholog(s) have protein binding, bridging activity and role in CVT pathway, ER-associated protein catabolic process, protein complex localization, protein processing, vesicle organization	*ATG19*	Not described	No	No
CAGL0C01243g		1,13		Histidinol-phosphate aminotransferase, catalyzes the seventh step in histidine biosynthesis; responsive to general control of amino acid biosynthesis; mutations cause histidine auxotrophy and sensitivity to Cu, Co, and Ni salts	*HIS5*	Not described	No	Yes
CAGL0C01243g		1,13		Histidinol-phosphate aminotransferase, catalyzes the seventh step in histidine biosynthesis; responsive to general control of amino acid biosynthesis; mutations cause histidine auxotrophy and sensitivity to Cu, Co, and Ni salts	*HIS5*	Not described	No	Yes
CAGL0D00286g	*CgBMT1*	1,12	-	Beta mannosyltransferase	-	-	-	No
CAGL0C04939g		1,12		Putative protein of unknown function; has sequence or structural similarity to lipases	*YJR107W*	Not described	No	No
CAGL0I02046g		1,12		Unknown	-	Not described	-	No
CAGL0L00957g	-	1,11	-	No Description Available	*CAJ1*	Not described	No	Yes
CAGL0H09218g	CgSDT1	1,11		Pyrimidine nucleotidase; overexpression suppresses the 6-AU sensitivity of transcription elongation factor S-II, as well as resistance to other pyrimidine derivatives	*SDT1*	Not described	No	Yes
CAGL0M02629g		1,11		Conserved protein of the mitochondrial matrix, performs a scaffolding function during assembly of iron-sulfur clusters, interacts physically and functionally with yeast frataxin (Yfh1p); isu1 isu2 double mutant is inviable	*ISU1*	Not described	No	Yes
CAGL0M00550g	-	1,10	-	Ortholog(s) have cytosol, nucleus localization	*STR2*	Not described	No	No
CAGL0C02519g	-	1,10	-	Has domain(s) with predicted nucleic acid binding activity	*MIG3*	Not described	No	Yes
CAGL0L07810g	-	1,10	-	Ortholog(s) have protein kinase activity	*SAT4*	Not described	No	No
CAGL0H03311g		1,10		Putative protein of unknown function; non-essential gene; interacts genetically with CHS5, a gene involved in chitin biosynthesis	*YGL081W*	Not described	No	Yes
CAGL0J03916g		1,10		Protein implicated in the regulation of ergosterol biosynthesis; one of a seven member gene family with a common essential function and non-essential unique functions; similar to human oxysterol binding protein (OSBP)	*HES1*	Not described	No	Yes
CAGL0K10736g	CgCYB2	1,10		Cytochrome b2 (L-lactate cytochrome-c oxidoreductase), component of the mitochondrial intermembrane space, required for lactate utilization; expression is repressed by glucose and anaerobic conditions	*CYB2*	Not described	No	No
CAGL0G02893g	-	1,09	-	Ortholog(s) have NADH kinase activity, role in NADP biosynthetic process, cellular response to oxidative stress and mitochondrial matrix localization	*POS5*	Not described	Yes	No
CAGL0E02035g	-	1,09	-	Ortholog(s) have fungal-type vacuole membrane localization	*MCH4*	Yes	No	Yes
CAGL0L11440g	-	1,09	-	Ortholog(s) have lipid binding activity and role in endoplasmic reticulum membrane organization, regulation of phosphatidylinositol dephosphorylation	*TCB3*	Not described	No	No
CAGL0I01496g		1,09		Hsp70 family ATPase, constituent of the import motor component of the Translocase of the Inner Mitochondrial membrane (TIM23 complex); involved in protein translocation and folding; subunit of SceI endonuclease	*SSC1*	Not described	No	Yes
CAGL0K09790g		1,09		CAP (cyclase-associated protein) subunit of adenylyl cyclase complex; N-terminus binds adenylyl cyclase and facilitates activation by RAS; C-terminus binds ADP-actin monomers, facilitating regulation of actin dynamics and cell morphogenesis	*SRV2*	Not described	No	Yes
CAGL0L02607g		1,09		Putative protein of unknown function; green fluorescent protein (GFP)-fusion protein localizes to the vacuole, while HA-tagged protein is found in the soluble fraction, suggesting cytoplasmic localization	*YHR202W*	Not described	No	No
CAGL0M08552g	-	1,08	-	Ortholog(s) have role in cation transport, regulation of membrane potential and plasma membrane localization	*PMP3*	Yes	No	Yes
CAGL0G01738g	-	1,08	-	Ortholog(s) have lipid binding activity and role in eisosome assembly, endocytosis, negative regulation of protein kinase activity, protein localization, response to heat	*PIL1*	Not described	No	Yes

CAGL0A00517g	-	1,08	-	Vacuolar Ca2+ ATPase involved in depleting cytosol of Ca2+ ions; prevents growth inhibition by activation of calcineurin in the presence of elevated concentrations of calcium; similar to mammalian PMCA1a	*PMC1*	Not described	No	Yes
CAGL0G08646g		1,08		Nuclear chromatin-associated protein of unknown function; overexpression promotes recovery from pheromone induced arrest and suppresses the stress sensitivity caused by a mutation in the E3 ubiquitin ligase Rsp5p; binds upstream of BAR1 and cell cycle-related genes; potential Cdc28p substrate; SBF regulated	*POG1*	Not described	No	No
CAGL0K05687g		1,08			-	Not described	-	Yes
CAGL0M13783g		1,08		Ubiquitin-specific protease involved in protein deubiquitination; catalytic activity regulated by an N-terminal TRAF-like domain and and C-terminal sequences; physically interacts with anaphase-promoting complex/cyclosome (APC/C) activator, Cdh1p	*UBP15*	Not described	Yes	No
CAGL0I10626g	-	1,07	-	Ortholog(s) have Golgi apparatus, endoplasmic reticulum, fungal-type vacuole membrane localization	*YGR125W*	Not described	No	No
CAGL0F01419g	CgAUS1	1,07		Plasma membrane sterol transporter of the ATP-binding cassette family; required, along with Pdr11p, for uptake of exogenous sterols and their incorporation into the plasma membrane; activity is stimulated by phosphatidylserine; sterol uptake is required for anaerobic growth because sterol biosynthesis requires oxygen	*AUS1*	Not described	No	Yes
CAGL0J09416g		1,07		Protein of unknown function, localized to the vacuolar outer membrane; predicted to be palmitoylated	*SNA4*	Not described	No	Yes
CAGL0M08602g		1,07		Cu(+2)-transporting P-type ATPase, required for export of copper from the cytosol into an extracytosolic compartment; has similarity to human proteins involved in Menkes and Wilsons diseases	*CCC2*	Not described	No	No
CAGL0M07568g	-	1,06	-	Ortholog(s) have ubiquitin-protein ligase activity	*UBC7*	Not described	No	No
CAGL0K04367g	-	1,06	-	Has domain(s) with predicted amino acid transmembrane transporter activity, role in amino acid transmembrane transport and membrane localization	*MUP1*	Not described	No	Yes
CAGL0C03509g		1,06		Ser/Thr protein kinase that regulates the putative phospholipid translocases Lem3p-Dnf1p/Dnf2p; phosphorylates and inhibits upstream inhibitory kinase, Ypk1p; localizes to the cytoplasm, early endosome/TGN compartments, and plasma membrane	*FPK1*	Not described	Yes	Yes
CAGL0I02552g		1,06		Transcription factor, involved in regulating multidrug resistance and oxidative stress response; forms a heterodimer with Pdr1p; contains a Zn(II)2Cys6 zinc finger domain that interacts with a pleiotropic drug resistance element in vitro	*STB5*	Not described	Yes	Yes
CAGL0L08712g	-	1,05	-	Ortholog(s) have cytoplasm, nucleus localization	*YPL014W*	Yes	No	Yes
CAGL0F06831g		1,05		ER membrane protein involved in regulation of OLE1 transcription, acts with homolog Spt23p; inactive ER form dimerizes and one subunit is then activated by ubiquitin/proteasome-dependent processing followed by nuclear targeting	*MGA2*	Not described	No	No
CAGL0M10549g		1,05		Cytochrome c oxidase subunit; has a role in assembly of respiratory supercomplexes; similar to Rcf1p, and either Rcf1p or Rcf2p is required for late-stage assembly of the Cox12p and Cox13p subunits and for cytochrome c oxidase activity; associates with the cytochrome c oxidase - cytochrome bc1 supercomplex; null mutant accumulates reactive oxygen species; member of the conserved hypoxia induced gene family; C. elegans homolog is functional in yeast	*RCF2*	Not described	No	Yes
CAGL0M13365g		1,05		Protein involved in the control of meiotic nuclear division and coordination of meiosis with spore formation; transcription is induced midway through meiosis	*SSP1*	Not described	No	No
CAGL0A02024g		1,04		Protein that negatively regulates the SCF E3-ubiquitin ligase by interacting with and preventing neddyation of the cullin subunit, Cdc53p; longevity determinant that is preferentially expressed in young cells; similar to mammalian Cand1	*LAG2*	Yes	No	No

CAGL0B00242g		1,04		Silenced copy of ALPHA1 at HML, encoding a transcriptional coactivator involved in the regulation of mating-type alpha-specific gene expression	*HMLALPHA1*	Not described	No	Yes
CAGL0F02387g	CgPHO87	1,04		Low-affinity phosphate transporter; deletion of pho84, pho87, pho89, pho90, and pho91 causes synthetic lethality; transcription independent of Pi and Pho4p activity; overexpression results in vigorous growth	*PHO90*	Not described	No	Yes
CAGL0F07777g		1,04		Cytoplasmic aldehyde dehydrogenase, involved in beta-alanine synthesis; uses NAD+ as the preferred coenzyme; very similar to Ald2p; expression is induced by stress and repressed by glucose	*ALD3*	Not described	No	No
CAGL0K12694g		1,04		Actin, structural protein involved in cell polarization, endocytosis, and other cytoskeletal functions	*ACT1*	Not described	No	No
CAGL0L00649g		1,04		Acetyl-coA synthetase isoform which, along with Acs2p, is the nuclear source of acetyl-coA for histone acetylation; expressed during growth on nonfermentable carbon sources and under aerobic conditions	*ACS1*	Not described	No	Yes
CAGL0D02134g		1,03		Putative protein of unknown function; has similarity to Mgr3p, but unlike MGR3, is not required for growth of cells lacking the mitochondrial genome (null mutation does not confer a petite-negative phenotype)	*YKL133C*	Not described	No	Yes
CAGL0E00891g		1,03		Ribosomal RNA processing element (RRPE)-binding protein involved in the glucose-induced transition from quiescence to growth; restricted to nucleus in quiescent cells, released into cytoplasm after glucose repletion; binds Sin3p	*STB3*	Not described	No	No
CAGL0I03960g	CgVMA1	1,03		Subunit A of the eight-subunit V1 peripheral membrane domain of the vacuolar H+-ATPase; protein precursor undergoes self-catalyzed splicing to yield the extein Tfp1p and the intein Vde (PI-SceI), which is a site-specific endonuclease	*VMA1*	Not described	No	Yes
CAGL0K04477g		1,03		C-4 methyl sterol oxidase, catalyzes the first of three steps required to remove two C-4 methyl groups from an intermediate in ergosterol biosynthesis; mutants accumulate the sterol intermediate 4,4-dimethylzymosterol	*ERG25*	Not described	No	No
CAGL0M11484g		1,03		Pentafunctional arom protein, catalyzes steps 2 through 6 in the biosynthesis of chorismate, which is a precursor to aromatic amino acids	*ARO1*	Not described	Yes	Yes
CAGL0J01529g	-	1,02	-	Ortholog(s) have role in histone deacetylation, negative regulation of antisense RNA transcription, regulation of DNA-dependent DNA replication initiation, transcription elongation from RNA polymerase II promoter	*RCO1*	Not described	No	No
CAGL0A03344g		1,02		Putative integral membrane E3 ubiquitin ligase; acts with Asi1p and Asi2p to ensure the fidelity of SPS-sensor signalling by maintaining the dormant repressed state of gene expression in the absence of inducing signals	*ASI3*	Not described	No	Yes
CAGL0I10054g		1,02		Protein involved in sphingolipid biosynthesis; type II membrane protein with similarity to Kre6p	*SKN1*	Not described	No	Yes
CAGL0L08338g		1,02		Minor isoform of tropomyosin, binds to and stabilizes actin cables and filaments, which direct polarized cell growth and the distribution of several organelles; appears to have distinct and also overlapping functions with Tpm1p	*TPM2*	Not described	No	Yes
CAGL0J08349g		1,01		Part of the evolutionarily-conserved CCR4-NOT transcriptional regulatory complex involved in controlling mRNA initiation, elongation, and degradation	*CAF120*	Not described	No	No
CAGL0L03982g		1,01		Malate synthase, enzyme of the glyoxylate cycle, involved in utilization of non-fermentable carbon sources; expression is subject to carbon catabolite repression; localizes in peroxisomes during growth in oleic acid medium	*MLS1*	Not described	No	Yes
CAGL0D06512g		1,00			*SDC25*	Not described	No	Yes
CAGL0F01287g	CgGAS5	1,00		1,3-beta-glucanosyltransferase, has similarity to Gas1p; localizes to the cell wall	*GAS5*	Not described	No	Yes
CAGL0K04873g		1,00		Putative protein of unknown function; green fluorescent protein (GFP)-fusion protein localizes to the cytoplasm and nucleus; YDL233W is not an essential gene	*YDL233W*	Not described	No	Yes

Genes whose acetic acid-induced transcriptional activation was partly dependent of CgHaa1

ORF	*C. glabrata* gene			Function	*S. cerevisiae* orthologue	*S. cerevisiae* orthologue regulated by ScHaa1?	*S. cerevisiae* homologue confers resistance to acetic acid?	*C. glabrata* promoter harbors an HRE motif
CAGL0I06182g	*CgPIR2*	7,65	3,10	O-mannosylated heat shock protein that is secreted and covalently attached to the cell wall via beta-1,3-glucan and disulfide bridges; required for cell wall stability; induced by heat shock, oxidative stress, and nitrogen limitation	*HSP150*	Not described	No	Yes
CAGL0G05632g	-	6,95	1,41	Ortholog(s) have cytoplasm localization	*YDL218W*	Not described	No	No
CAGL0I10010g	-	6,82	3,66	v-SNARE binding protein that facilitates specific protein retrieval from a late endosome to the Golgi; modulates arginine uptake, possible role in mediating pH homeostasis between the vacuole and plasma membrane H(+)-ATPase	*BTN2*	Not described	No	Yes
CAGL0M01166g	-	6,44	4,20	Ortholog(s) have ferrous iron binding activity, role in mitochondrial genome maintenance, thiamine biosynthetic process, thiazole biosynthetic process and cytosol, nucleus localization	*THI4*	Not described	No	No
CAGL0F08261g	-	6,11	2,55	Ortholog(s) have phosphopyruvate hydratase activity, role in glycolysis, regulation of vacuole fusion, non-autophagic and fungal-type vacuole, internal side of plasma membrane, mitochondrion, phosphopyruvate hydratase complex localization	*ENO1*	Not described		No
CAGL0G03883g	-	6,10	4,05	Disaggregase; Heat shock protein that cooperates with Ydj1p (Hsp40) and Ssa1p (Hsp70) to refold and reactivate previously denatured, aggregated proteins; responsive to stresses including: heat, ethanol, and sodium arsenite; involved in [PSI+] propagation	*HSP104*	Yes		No
CAGL0G03289g	*CgSSA3*	6,10	2,09	Heat shock protein of the HSP70 family	*SSA4*	Yes	Yes	No
CAGL0J00451g	-	5,66	4,38	Putative glyceraldehyde-3-phosphate dehydrogenase; protein differentially expressed in azole resistant strain; expression downregulated in biofilm vs planktonic cell culture	*TDH3*	Not described	No	No
CAGL0M08822g	*CgHSP78*	5,06	3,34	Ortholog(s) have ATPase activity, misfolded protein binding activity	*HSP78*	Not described	No	Yes
7,36 CAGL0E00803g	-	4,82	2,88	Putative small cytosolic stress-induced chaperone; gene is upregulated in azole-resistant strain	*HSP42*	Yes	No	Yes
CAGL0H03707g	-	4,64	2,57	Ortholog(s) have role in protein folding, translational initiation and cytosolic small ribosomal subunit, nucleus localization	**SIS1*	Not described	No	Yes
CAGL0G08866g	-	4,38	1,96	Ortholog(s) have RNA polymerase II transcription factor binding, RNA polymerase II transcription factor binding transcription factor activity, sequence-specific DNA binding activity	*FKH2*	Yes	No	Yes
CAGL0C02321g	*CgPHM8*	4,25	2,36	Ortholog(s) have nucleotidase activity and role in pyrimidine nucleobase metabolic process	*SDT1*	Not described	No	Yes
CAGL0K10164g	-	4,08	2,54	Predicted GPI-linked protein; putative adhesin-like protein	*SPI1*	Yes	No	Yes
CAGL0I09724g	-	4,07	2,05	Unknown	-	-	No	Yes
CAGL0F04631g	-	4,03	1,84	No Description Available	*MOH1*	Not described	No	Yes
CAGL0F04631g		4,03	1,84	Protein of unknown function, has homology to kinase Snf7p; not required for growth on nonfermentable carbon sources; essential for survival in stationary phase	*MOH1*	Not described	No	Yes
CAGL0I10384g	*CgTPO3*	3,98	1,49	Predicted polyamine transporter of the major facilitator superfamily; required for azole resistance	*TPO3*	Yes	Yes	No
CAGL0J06050g	-	3,97	1,41	Has domain(s) with predicted role in cellular amino acid metabolic process	*YGP1*	Yes	No	Yes
CAGL0H00704g	-	3,95	2,86	Protein of unknown function; mobilized into polysomes upon a shift from a fermentable to nonfermentable carbon source; potential Cdc28p substrate	*ICY2*	Not described	Yes	Yes
CAGL0G08624g	*CgQDR2*	3,92	2,69	Drug:H+ antiporter of the Major Facilitator Superfamily, confers imidazole drug resistance, involved in quinidine/multidrug efflux; gene is activated by Pdr1p; upregulated in azole-resistant strain	*QDR1*	Not described	No	Yes
CAGL0F04521g	-	3,84	1,19	No Description Available	*ECM13*	Yes	No	Yes
CAGL0L07722g	*CgPGK1*	3,69	2,34	Putative 3-phosphoglycerate kinase; protein differentially expressed in azole resistant strain; protein abundance increased in ace2 mutant cells	**PGK1*	Not described	No	Yes
CAGL0H04279g	*CgMT-IIB*	3,65	2,73	Copper-binding metallothionein, involved in sequestration of metal ions; inducible by copper and silver; present in multiple (3-9) tandemly arranged copies in various strains	-	-	-	Yes
CAGL0F04895g	-	3,61	1,45	Ortholog(s) have glycogen phosphorylase activity, role in glycogen catabolic process and cell surface, cytoplasm, hyphal cell wall localization	*GPH1*	Not described	No	Yes
CAGL0K12254g		3,60	2,71	Peripheral membrane protein located at Vid (vacuole import and degradation) vesicles; regulates fructose-1,6-bisphosphatase (FBPase) targeting to the vacuole; promotes proteasome-dependent catabolite degradation of FBPase	*VID24*	Not described	No	No
CAGL0K04301g	-	3,47	1,45	Putative mitochondrial Ser/Thr protein kinase; gene is upregulated in azole-resistant strain	*FMP48*	Yes	Yes	No

CAGL0E06358g	CgGPM1	3,46	2,58	Tetrameric phosphoglycerate mutase, mediates the conversion of 3-phosphoglycerate to 2-phosphoglycerate during glycolysis and the reverse reaction during gluconeogenesis	GPM1	Not described	No	No
CAGL0H02101g	-	3,31	1,95	Ortholog(s) have role in RNA metabolic process and cytoplasm, nucleus localization	RTC3	Yes	No	Yes
CAGL0J10296g	-	3,30	1,40	Ortholog(s) have ATPase activator activity, unfolded protein binding activity, role in protein sumoylation and mitochondrion, nucleus localization	APJ1	Not described	No	Yes
CAGL0F08745g	-	3,22	1,70	Protein involved in resistance to dessication stress; Stf2p exhibits antioxidant properties, and its overexpression prevents ROS accumulation and apoptosis; binds to the F0 sector of mitochondrial F1F0 ATPase in vitro and is proposed to modulate the inhibitory action of Inh1p and Stf1p	STF2	Yes	No	Yes
CAGL0M02167g	-	3,22	1,83	Pheromone-regulated protein proposed to be involved in mating; predicted to have 1 transmembrane segment; transcriptionally regulated by Ste12p during mating and by Cat8p during the diauxic shift	PRM4	Not described	No	Yes
CAGL0A03102g	CgARO10	3,17	1,66	Phenylpyruvate decarboxylase, catalyzes decarboxylation of phenylpyruvate to phenylacetaldehyde, which is the first specific step in the Ehrlich pathway	ARO10	Not described	No	Yes
CAGL0H00418g		3,16	1,26	Unknown	-	-	-	Yes
CAGL0K04719g	-	3,15	1,09	Ortholog(s) have mitochondrion, ribosome localization	YNL208W	Not described	No	No
CAGL0I00418g	-	3,15	2,00	Ortholog(s) have electron carrier activity, stearoyl-CoA 9-desaturase activity	*OLE1	Not described	No	No
CAGL0G03795g	CgSSA1	3,13	1,84	Heat shock protein of the HSP70 family	SSA2	Not described	No	Yes
CAGL0I05934g	-	3,12	1,95	Ortholog(s) have role in cellular response to water deprivation and cytoplasm localization	YJL144W	Not described	No	Yes
CAGL0E05566g	-	3,11	1,41	Serine-rich protein that contains a basic-helix-loop-helix (bHLH) DNA binding motif; binds E-boxes of glycolytic genes and contributes to their activation; may function as a transcriptional activator in Ty1-mediated gene expression	TYE7	Yes	No	No
CAGL0F01111g	-	3,09	1,88	Protein with a possible role in phospholipid biosynthesis, based on inositol-excreting phenotype of the null mutant and its suppression by exogenous choline	OPI10	Not described	No	Yes
CAGL0I02486g		3,06	2,14	Enolase II, a phosphopyruvate hydratase that catalyzes the conversion of 2-phosphoglycerate to phosphoenolpyruvate during glycolysis and the reverse reaction during gluconeogenesis; expression is induced in response to glucose	ENO2	Not described	No	Yes
CAGL0M13651g	-	3,04	1,62	Ortholog(s) have serine-type carboxypeptidase activity, role in phytochelatin biosynthetic process and endoplasmic reticulum, fungal-type vacuole localization	PRC1	Not described	No	No
CAGL0J04466g		3,04	1,68	Plasma membrane protein with a role in cell wall integrity; co-localizes with Sur7p in punctate membrane patches; null mutant displays decreased thermotolerance; transcription induced upon cell wall damage and metal ion stress	PUN1	Not described	No	No
CAGL0C00275g		3,04	1,01	Putative cysteine protease	-	-	-	No
CAGL0C02893g	-	3,01	1,22	Ortholog(s) have protein serine/threonine kinase activity	HRK1	Yes	Yes	Yes
CAGL0H03971g	-	2,98	1,82	Ortholog(s) have membrane raft, mitochondrion, plasma membrane localization	YCP4	Not described	No	No
CAGL0M08492g	CgPIR3	2,88	1,66	Pir protein family member, predicted GPI-anchor	PIR1	Not described	No	Yes
CAGL0L02497g	CgFBA1	2,84	1,59	Fructose-bisphosphate aldolase; expression downregulated in biofilm vs planktonic cell culture; protein abundance increased in ace2 mutant cells	*FBA1	Not described	No	Yes
CAGL0L06424g		2,77	1,42		YDR134C	Yes		Yes
CAGL0H08393g	-	2,75	1,55	Has domain(s) with predicted amino acid transmembrane transporter activity, role in amino acid transmembrane transport, amino acid transport, transmembrane transport and integral to membrane, membrane localization	BAP3	Not described	No	Yes
CAGL0M12034g	CgPYK1	2,72	1,75	Pyruvate kinase, functions as a homotetramer in glycolysis to convert phosphoenolpyruvate to pyruvate, the input for aerobic (TCA cycle) or anaerobic (glucose fermentation) respiration	CDC19	Not described	No	No
CAGL0K07634g	CgGAT1	2,65	1,07	Ortholog(s) have RNA polymerase II core promoter proximal region sequence-specific DNA binding transcription factor activity involved in positive regulation of transcription, more	GAT1	Not described	No	No
CAGL0I01342g	-	2,63	1,00	Ortholog(s) have L-allo-threonine aldolase activity, role in carnitine biosynthetic process, glycine biosynthetic process, threonine catabolic process and cytosol, nucleus localization	GLY1	Not described	Yes	Yes
CAGL0E04774g	-	2,55	1,05	Ortholog(s) have cytoplasm localization	YDR222W	Not described	No	Yes
CAGL0M11000g		2,50	1,10	Putative protein of unknown function; expression is regulated by Msn2p/Msn4p	YNR034W-A	Yes	No	Yes
CAGL0G03531g	-	2,49	1,10	No Description Available	SPR6	Not described	No	No
CAGL0H01375g	CgSUR2	2,44	1,34	Predicted sphinganine hydroxylase with role in sphingolipid biosynthesis; mutants show reduced sensitivity to caspofungin and increased sensitivity to micafungin	SUR2	Yes	Yes	Yes

CAGL0F08041g	*CgPFK1*	2,43	1,23	Putative phosphofructokinase, alpha subunit; increased protein abundance in azole resistant strain	*PFK1*	Not described	Yes	No
CAGL0G08844g		2,35	1,16	Zinc cluster protein proposed to function as a transcriptional regulator involved in the stress response; null mutants have a respiratory deficiency, calcofluor white sensitivity and slightly increased cycloheximide resistance	*ASG1*	Yes	Yes	Yes
CAGL0F04917g	-	2,17	1,15	Putative targeting subunit for the type-1 protein phosphatase Glc7p that tethers it to the Gsy2p glycogen synthase	*PIG1*	Not described	No	Yes
CAGL0L11154g	-	2,14	1,13	Ortholog(s) have carboxylesterase activity, lysophospholipase activity, role in phosphatidylcholine catabolic process, regulation of phospholipid biosynthetic process and endoplasmic reticulum localization	*NTE1*	Not described	No	Yes
CAGL0G10219g	*AWP12*	2,11	1,02	Adhesin-like protein with 5 tandem repeats	-	-	-	Yes
CAGL0G02849g		2,08	1,15	Protein that interacts with Ulp1p, a Ubl (ubiquitin-like protein)-specific protease for Smt3p protein conjugates; detected in a phosphorylated state in the mitochondrial outer membrane; also detected in ER and nuclear envelope	*UIP4*	Not described	No	Yes
