# Supplementary material for: The CgHaa1-Regulon Mediates Response and Tolerance to Acetic Acid Stress in the Human Pathogen Candida glabrata
Source: G3 (Bethesda). 2016 Nov 4;7(1):1–18. doi: 10.1534/g3.116.034660 (PMC5217100; doi:10.1534/g3.116.034660)
Supplement: Supplementary file 9 [file 1TableS4.pdf]

Supplementary table S4 – Comparison of the ScHaa1- and CgHaa1- dependent regulatory networks. The dataset of *C. glabrata* genes herein found to be regulated by CgHaa1 under acetic acid stress was compared with the dataset of genes described to be regulated by ScHaa1, based on the information available at the YEASTRACT database. Identification of orthologues was performed using the Yeastmine algorithm available at the Candida Genome database website after which a manual curation of the data based on BLASTP was performed. The promoter region of *S. cerevisiae* or *C. glabrata* genes was searched for the described ScHaa1 binding site (5'-(G/C)(A/C)GG(G/C)-3', designated Haa1-responsive element – HRE; Mira et al., 2011) using the DNA pattern matching algorithms embedded in the RSA tools website. Cells highlighted in grey correspond to the *S. cerevisiae* genes found to be regulated by ScHaa1 specifically under acetic acid stress, based on the dataset of Mira et al., (2010).

Acetic acid-responsive genes regulated by ScHaa1 only

ORF	Function	*S. cerevisiae* orthologue	C. *glabrata* promoter harbors an HRE motif	*S. cerevisiae* promoter harbors an HRE motif
CAGL0A00891g	Ortholog(s) have actin binding, cytoskeletal regulatory protein binding, enzyme activator activity	YLR319C	No	No
CAGL0A00913g	Ortholog(s) have role in double-strand break repair, meiotic sister chromatid segregation, recombinational repair, replication fork processing and Cul8-RING ubiquitin ligase complex, nucleus localization	YLR320W	No	No
CAGL0A01001g	No Description Available	YLR326W	Yes	No
CAGL0A01023g	Ortholog(s) have nicotinamide-nucleotide adenylyltransferase activity, role in NAD biosynthetic process and cytosol, nucleus localization	YLR328W	Yes	Yes
CAGL0A01111g	Ortholog(s) have proton-transporting ATP synthase activity, rotational mechanism activity, role in ATP synthesis coupled proton transport and mitochondrial proton-transporting ATP synthase, central stalk localization	YPL271W	Yes	Yes
CAGL0A01540g	Ortholog(s) have pre-mRNA 5'-splice site binding, structural constituent of ribosome activity, role in negative regulation of mRNA splicing, via spliceosome, rRNA processing and cytosol localization	YGL030W	No	No
CAGL0A01760g	Ortholog(s) have role in mitochondrial translation and mitochondrion localization	YDR341C	No	No
CAGL0A02024g	Ortholog(s) have role in negative regulation of ubiquitin-protein transferase activity, replicative cell aging and mitochondrion localization	YOL025W	No	No
CAGL0A02365g	Ortholog(s) have role in cellular response to oxidative stress and cytosol, nucleus localization	YDR346C	No	No
CAGL0A02860g	Ortholog(s) have 5'-3' exonuclease activity, role in deadenylation-dependent decapping of nuclear-transcribed mRNA and cytoplasm localization	YDR370C	No	No
CAGL0A03168g	60S acidic ribosomal protein	YDR382W	No	No
CAGL0A03366g	Ortholog(s) have IMP cyclohydrolase activity, phosphoribosylaminoimidazolecarboxamide formyltransferase activity, role in 'de novo' IMP biosynthetic process and cytosol, plasma membrane localization	YLR028C	Yes	No
CAGL0A03652g	Ortholog(s) have role in maturation of 5.8S rRNA from tricistronic rRNA transcript (SSU-rRNA, 5.8S rRNA, LSU-rRNA), maturation of LSU-rRNA from tricistronic rRNA transcript (SSU-rRNA, 5.8S rRNA, LSU-rRNA) and nucleolus localization	YBR142W	No	No
CAGL0A04081g	Predicted GPI-linked cell wall protein	YLR194C	No	Yes
CAGL0A04169g	Ortholog(s) have role in mitochondrion inheritance and cellular bud neck, incipient cellular bud site, mitochondrial outer membrane localization	YLR190W	No	Yes
CAGL0A04213g	Has domain(s) with predicted nucleic acid binding, nucleotide binding activity	YBL051C	No	No
CAGL0A04433g	Ortholog(s) have thioredoxin peroxidase activity and role in cell redox homeostasis, cellular response to oxidative stress, response to cadmium ion, sporocarp development involved in sexual reproduction	YBL064C	Yes	No
CAGL0A04675g	Ortholog(s) have protein tag activity	YBL078C	No	Yes
CAGL0B00308g	Ortholog(s) have protein anchor activity, role in vacuole inheritance and Myo2p-Vac17p-Vac8p transport complex, fungal-type vacuole membrane localization	YCL063W	No	No
CAGL0B00726g	Has domain(s) with predicted ATP binding, phosphotransferase activity, alcohol group as acceptor activity and role in carbohydrate metabolic process	YCL040W	No	No
CAGL0B01100g	Phosphatidylethanolamine-binding protein	YLR178C	Yes	Yes
CAGL0B01188g	Ortholog(s) have chromatin binding activity, role in cellular response to DNA damage stimulus and Rpd3L-Expanded complex, mitochondrion localization	YLR183C	No	No
CAGL0B01203g	Ortholog(s) have cytosolic large ribosomal subunit localization	YDR500C	Yes	No
CAGL0B01375g	Ortholog(s) have RNA polymerase II C-terminal domain phosphoserine binding, RNA polymerase II core binding, RNA polymerase II transcription factor binding transcription factor activity, triplex DNA binding activity	YOL145C	Yes	No

CAGL0B01441g	Ortholog(s) have histone deacetylase activity, transcription coactivator activity, transcription corepressor activity	YNL330C	Yes	No
CAGL0B01683g	Ortholog(s) have role in cellular copper ion homeostasis, endosomal transport, invasive growth in response to glucose limitation, pseudohyphal growth, vacuolar transport	YLR083C	No	No
CAGL0B01815g	Ortholog(s) have structural constituent of ribosome activity and mitochondrial large ribosomal subunit, nucleolus localization	YDR116C	No	No
CAGL0B01881g	Ortholog(s) have tRNA (guanine-N2-)-methyltransferase activity, role in tRNA N2-guanine methylation and mitochondrion, nuclear inner membrane localization	YDR120C	Yes	No
CAGL0B01925g	Has domain(s) with predicted ATP binding, protein kinase activity, protein tyrosine kinase activity, transferase activity, transferring phosphorus-containing groups activity and role in protein phosphorylation	YDR122W	No	Yes
CAGL0B02563g	Expressed protein of unknown function	YML128C	Yes	Yes
CAGL0B02761g	Ortholog(s) have phosphoprotein phosphatase activity, role in endoplasmic reticulum unfolded protein response, protein dephosphorylation, traversing start control point of mitotic cell cycle and endoplasmic reticulum localization	YLR361C	No	No
CAGL0K04279g	Protein of unknown function	YGR049W	No	No
CAGL0B03091g	Protein of unknown function	YGR041W	No	Yes
CAGL0B03465g	Ortholog(s) have role in ethanol metabolic process and mitochondrial inner membrane localization	YLR251W	No	No
CAGL0B03575g	Ortholog(s) have histone acetyltransferase activity, histone binding activity	YEL056W	No	No
CAGL0B04301g	Ortholog(s) have cytosol localization	YBR028C	No	No
CAGL0C01199g	Putative Zn(2)-Cys(6) binuclear cluster transcription factor, involved in transcriptional regulation of ergosterol biosynthesis and uptake	YLR228C	Yes	No
CAGL0C01221g	Ortholog(s) have guanyl-nucleotide exchange factor activity, translation initiation factor activity and role in regulation of translational initiation	YDR211W	Yes	No
CAGL0C01325g	Ortholog(s) have cytochrome-c oxidase activity, nitrite reductase (NO-forming) activity, role in mitochondrial electron transport, cytochrome c to oxygen and mitochondrial respiratory chain complex IV localization	YNL052W	Yes	Yes
CAGL0C01435g	Ortholog(s) have role in ribosome biogenesis and mitochondrion localization	YPL183W-A	No	No
CAGL0C01441g	Ortholog(s) have role in endocytic recycling and cytosol, endosome, nucleus localization	YPL183C	No	No
CAGL0C01485g	Ortholog(s) have methylated histone binding, transcription factor binding activity	YPL182C	No	-
CAGL0C01793g	Ortholog(s) have nascent polypeptide-associated complex binding activity, role in protein targeting to mitochondrion, ribosome localization and integral component of mitochondrial outer membrane localization	YBR230C	No	No
CAGL0C01947g	Ortholog(s) have glutamate-tRNA ligase activity, mRNA binding activity, role in glutamyl-tRNA aminoacylation and cytosol, methionyl glutamyl tRNA synthetase complex, mitochondrion, nucleus localization	YGL245W	No	No
CAGL0C02607g	Ortholog(s) have ATP binding, phosphatidylinositol binding activity	YLR396C	No	No
CAGL0C02623g	Ortholog(s) have cytochrome-c oxidase activity, role in mitochondrial electron transport, cytochrome c to oxygen and mitochondrial respiratory chain complex IV, plasma membrane localization	YLR395C	No	No
CAGL0C02981g	Putative acyl-CoA:sterol acyltransferase involved in sterol esterification; gene is upregulated in azole-resistant strain	YCR048W	No	No
CAGL0C03597g	Putative actin-binding protein	YCR088W	No	No
CAGL0C04543g	Ortholog(s) have alditol:NADP+ 1-oxidoreductase activity, role in D-xylose catabolic process, arabinose catabolic process, cellular response to oxidative stress and cytosol, nucleus localization	YJR096W	No	Yes
CAGL0C04741g	Cytosolic copper-zinc superoxide dismutase	YJR104C	No	Yes
CAGL0C05093g	Ortholog(s) have dipeptidyl-peptidase activity and cytoplasm, nucleus localization	YOL057W	No	No
CAGL0C05291g	Ortholog(s) have cytosol localization	YOL066C	No	No
CAGL0C05313g	Ortholog(s) have peptide alpha-N-acetyltransferase activity, role in N-terminal protein amino acid acetylation and NatA complex, cytosolic ribosome, mitochondrion localization	YDL040C	No	No
CAGL0C05335g	Ortholog(s) have sequence-specific DNA binding RNA polymerase II transcription factor activity, transcription regulatory region DNA binding activity	YOL067C	No	No
CAGL0C05357g	Histone deacetylase; ortholog of S. cerevisiae SIR2; sensor of niacin limitation; regulates gene expression under niacin-limiting conditions	YOL068C	No	No
CAGL0C05489g	Ortholog(s) have GTPase activator activity, role in regulation of vacuole fusion, non-autophagic, vesicle-mediated transport and cytosol, nucleus localization	YDL234C	No	Yes
CAGL0D00418g	Protein of unknown function	YKL187C	No	Yes

CAGL0D00704g	Ortholog(s) have endoplasmic reticulum, plasma membrane localization	YDL072C	No	No
CAGL0D00748g	Ortholog(s) have cytochrome-c oxidase activity, role in mitochondrial electron transport, cytochrome c to oxygen and mitochondrial respiratory chain complex IV, plasma membrane localization	YDL067C	No	No
CAGL0D00858g	Ortholog(s) have structural constituent of ribosome activity and cytosolic small ribosomal subunit, nucleus localization	YDL061C	No	No
CAGL0D01078g	Ortholog(s) have role in cargo loading into COPII-coated vesicle and COPII vesicle coat, cytosol, endoplasmic reticulum exit site localization	YNL049C	No	No
CAGL0D01672g	Ortholog(s) have role in Golgi inheritance, Golgi to plasma membrane transport, endoplasmic reticulum inheritance, exocytosis	YPR055W	Yes	No
CAGL0D01804g	Ortholog(s) have D-serine ammonia-lyase activity, role in D-serine metabolic process and cytosol, nucleus localization	YGL196W	Yes	No
CAGL0D01958g	Ortholog(s) have mitochondrion, plasma membrane localization	YKL141W	Yes	No
CAGL0D02310g	Ortholog(s) have mRNA binding activity, role in reciprocal meiotic recombination and mitochondrion localization	YOR354C	No	Yes
CAGL0D03124g	Ortholog(s) have role in cellular response to drug, rRNA processing, ribosomal large subunit assembly and nucleolus, preribosome, large subunit precursor localization	YLL008W	No	No
CAGL0D03586g	Ortholog(s) have role in long-chain fatty acid biosynthetic process, mitochondrial respiratory chain complex I assembly and mitochondrion localization	YKL192C	No	No
CAGL0D03630g	Ortholog(s) have ATPase activity, polynucleotide 5'-phosphatase activity	YPL228W	No	No
CAGL0D03652g	Ortholog(s) have role in chromatin silencing at rDNA, chromatin silencing at silent mating-type cassette, chromatin silencing at telomere, regulation of transcription from RNA polymerase II promoter and nucleus localization	YMR179W	No	No
CAGL0D03674g	Ortholog(s) have ATP binding, ATPase activity, mRNA binding activity and role in poly(A)+ mRNA export from nucleus, ribosomal small subunit biogenesis	YPL226W	No	No
CAGL0D03762g	Protein of unknown function	YDR309C	No	Yes
CAGL0D03982g	Ortholog(s) have 1-pyrroline-5-carboxylate dehydrogenase activity, role in glutamate biosynthetic process, hyphal growth, proline catabolic process to glutamate and cytosol, mitochondrial matrix localization	YHR037W	Yes	No
CAGL0D04114g	Ortholog(s) have NADPH-hemoprotein reductase activity, electron carrier activity, role in cellular response to drug, ergosterol biosynthetic process and endoplasmic reticulum, mitochondrial outer membrane localization	YHR042W	No	Yes
CAGL0D04312g	Ortholog(s) have mRNA binding, ribosomal small subunit binding activity	YMR080C	Yes	No
CAGL0D04752g	Ortholog(s) have pyridoxine:NADP 4-dehydrogenase activity, role in pyridoxal biosynthetic process and cytosol, nucleus localization	YPR127W	Yes	No
CAGL0D05632g	Ortholog(s) have copper chaperone activity, role in intracellular copper ion transport, mitochondrial respiratory chain complex IV assembly and cytosol, mitochondrial intermembrane space, nucleus localization	YLL009C	No	Yes
CAGL0D06050g	Protein of unknown function	YJR054W	No	No
CAGL0D06468g	Ortholog(s) have NEDD8 transferase activity, role in protein neddylation, regulation of mitotic cell cycle and cytosol, nucleus localization	YLR306W	No	No
CAGL0D06556g	Ortholog(s) have RNA binding, aspartate-tRNA ligase activity, role in aspartyl-tRNA aminoacylation and cytosol, nucleus localization	YLL018C	Yes	No
CAGL0E00385g	Ortholog(s) have oligopeptide-transporting ATPase activity, role in oligopeptide export from mitochondrion and mitochondrial inner membrane localization	YLR188W	No	No
CAGL0E00451g	Ortholog(s) have role in cellular response to biotic stimulus, cellular response to starvation and filamentous growth of a population of unicellular organisms in response to biotic stimulus, more	YCR093W	No	Yes
CAGL0E00759g	Ortholog(s) have inositol tetrakisphosphate 3-kinase activity, inositol tetrakisphosphate 6-kinase activity and inositol-1,3,4,5,6-pentakisphosphate kinase activity, more	YDR173C	Yes	No
CAGL0E01177g	Cyclophilin; peptidyl-prolyl cis-trans isomerase	YDR154C	Yes	-
CAGL0E01331g	Transcription factor; mutants display increased fungal burdens in mouse lungs and brain	YDR146C	Yes	No
CAGL0E01573g	Ortholog(s) have role in fungal-type cell wall organization and cytosol, nucleus localization	YNL313C	Yes	No
CAGL0E01617g	Ortholog(s) have inorganic cation transmembrane transporter activity, role in magnesium ion transport and cytoplasm, plasma membrane localization	YOL130W	No	No
CAGL0E01903g	Ortholog(s) have tRNA methyltransferase activity, role in tRNA methylation and cytoplasm, nucleus localization	YOL125W	No	Yes

CAGL0E01947g	Ortholog(s) have mRNA binding activity, role in mRNA cleavage, mRNA polyadenylation, response to DNA damage checkpoint signaling and cytoplasmic stress granule, mRNA cleavage factor complex localization	YOL123W	Yes	Yes
CAGL0E02101g	Has domain(s) with predicted nucleotidyltransferase activity	YOL115W	No	No
CAGL0E02431g	Has domain(s) with predicted DNA binding, DNA topoisomerase type I activity, DNA topoisomerase type II (ATP-hydrolyzing) activity, role in DNA topological change and chromosome localization	YOL006C	No	No
CAGL0E02585g	Ortholog(s) have 3'-5'-exoribonuclease activity	YOR001W	No	No
CAGL0E04334g	Putative cytochrome P-450 lanosterol 14-alpha-demethylase; target enzyme of azole antifungal drugs; increased protein abundance in azole resistant strain	YHR007C	No	Yes
CAGL0E05214g	Ortholog(s) have cytosol, nucleus localization	YPL088W	No	Yes
CAGL0E05302g	Ortholog(s) have protein phosphatase 1 binding activity, role in actin filament organization, cortical actin cytoskeleton organization, endocytosis, protein secretion and actin cortical patch, nucleus localization	YOR329C	No	No
CAGL0E05324g	Ortholog(s) have 3'-5' exonuclease activity, DNA-directed DNA polymerase activity, role in DNA biosynthetic process, mitochondrial DNA replication and mitochondrion localization	YOR330C	No	No
CAGL0E05368g	Ortholog(s) have magnesium ion transmembrane transporter activity, role in mitochondrial magnesium ion transport and mitochondrial inner membrane localization	YOR334W	No	No
CAGL0E05434g	Ortholog(s) have sequence-specific DNA binding activity	YOR337W	No	No
CAGL0E05896g	Ortholog(s) have GTP binding, GTPase activity	YPL218W	No	No
CAGL0E05918g	Has domain(s) with predicted protein kinase binding activity and role in regulation of cyclin-dependent protein serine/threonine kinase activity	YGL134W	No	No
CAGL0E05984g	No Description Available	YPL223C	No	No
CAGL0E06248g	Regulator of calcineurin; required for cell growth and activation of the calcineurin-Crz1 pathway in the presence of micafungin; activates calcineurin-dependent signaling	YKL159C	No	No
CAGL0F00561g	Ortholog(s) have RNA polymerase I activity and role in termination of RNA polymerase I transcription, transcription of nuclear large rRNA transcript from RNA polymerase I promoter	YJR063W	No	No
CAGL0F00671g	Ortholog(s) have role in actin filament bundle retrograde transport, establishment or maintenance of cell polarity regulating cell shape, formin-nucleated actin cable assembly and hyphal growth, more	YOR326W	No	No
CAGL0F00737g	Ortholog(s) have ubiquitin protein ligase binding activity and role in positive regulation of receptor internalization, protein ubiquitination, ubiquitin-dependent endocytosis	YOR322C	No	No
CAGL0F00803g	Component of the transcriptional Mediator complex that provides interfaces between RNA polymerase II and upstream activator proteins; one of the two Gal11p homologs of S. cerevisiae Gal11p present in C. glabrata	YOL051W	Yes	Yes
CAGL0F00869g	Ortholog(s) have role in ascospore wall assembly and ascospore wall, lipid particle, prospore membrane localization	YOL048C	No	Yes
CAGL0F01023g	Ortholog(s) have role in maturation of LSU-rRNA from tricistronic rRNA transcript (SSU-rRNA, 5.8S rRNA, LSU-rRNA) and nucleolus, preribosome, large subunit precursor localization	YOL041C	Yes	No
CAGL0F01331g	Ortholog(s) have role in mitochondrial RNA 5'-end processing and mitochondrial envelope localization	YOR017W	No	No
CAGL0F01573g	Ortholog(s) have mRNA binding, translation regulator activity and role in mitochondrial respiratory chain complex IV biogenesis, positive regulation of mitochondrial translational initiation	YLR067C	Yes	No
CAGL0F01705g	Putative cytosolic phenylalanyl-tRNA synthetase, alpha subunit	YLR060W	No	No
CAGL0F01771g	Ortholog(s) have role in ER-associated ubiquitin-dependent protein catabolic process and endoplasmic reticulum localization	YLR057W	No	No
CAGL0F01969g	No Description Available	YLR049C	No	No
CAGL0F01991g	Ortholog(s) have endoplasmic reticulum localization	YLR050C	No	No
CAGL0F02233g	Ortholog(s) have dipeptidase activity	YFR006W	Yes	No
CAGL0F02937g	Ortholog(s) have role in ribosomal large subunit assembly and cytosolic large ribosomal subunit, nucleolus localization	YDR417C	Yes	-
CAGL0F03575g	Ortholog(s) have role in protein targeting to vacuole involved in ubiquitin-dependent protein catabolic process via the multivesicular body sorting pathway and fungal-type vacuole localization	YPL176C	No	No
CAGL0F03619g	Ortholog(s) have role in establishment of mitotic spindle orientation and astral microtubule, cell cortex, dynactin complex, spindle pole body localization	YPL174C	No	No

CAGL0F03773g	Ortholog(s) have role in establishment of protein localization to plasma membrane and mitochondrion, plasma membrane localization	YMR212C	No	No
CAGL0F03795g	Ortholog(s) have role in mitochondrial genome maintenance, plasmid maintenance and cytosol, mitochondrion localization	YMR211W	No	No
CAGL0F03905g	Ortholog(s) have RNA uridylyltransferase activity, protein serine/threonine kinase activity	YMR216C	No	No
CAGL0F03993g	Ortholog(s) have phosphomevalonate kinase activity	YMR220W	No	No
CAGL0F04697g	Ortholog(s) have cytoplasm localization	YLR257W	Yes	Yes
CAGL0F04719g	Ortholog(s) have glycogen (starch) synthase activity, role in glycogen biosynthetic process and cytoplasm, nucleus localization	YLR258W	Yes	Yes
CAGL0F04873g	Ortholog(s) have endoplasmic reticulum localization	YPR159W	No	No
CAGL0F04939g	Ortholog(s) have DNA replication origin binding, chromatin binding, single-stranded DNA binding, single-stranded DNA-dependent ATP-dependent DNA helicase activity	YLR274W	No	No
CAGL0F05005g	Ortholog(s) have cytosol, mRNA cleavage and polyadenylation specificity factor complex localization	YLR277C	No	No
CAGL0F05357g	Ortholog(s) have RNA polymerase II core promoter proximal region sequence-specific DNA binding, more	YDR207C	Yes	Yes
CAGL0F05863g	Ortholog(s) have succinate dehydrogenase (ubiquinone) activity, role in mitochondrial electron transport, succinate to ubiquinone and mitochondrial respiratory chain complex II, succinate dehydrogenase complex (ubiquinone) localization	YDR178W	Yes	Yes
CAGL0F05885g	Ortholog(s) have cytoskeletal protein binding activity	YMR032W	No	Yes
CAGL0F05907g	Ortholog(s) have DNA translocase activity	YMR033W	Yes	No
CAGL0F05929g	Ortholog(s) have role in calcium ion import and plasma membrane localization	YMR034C	Yes	No
CAGL0M13189g	Putative transcription factor similar to S. cerevisiae Msn2p; involved in response to oxidative stress	YKL062W	No	Yes
CAGL0F06171g	Has domain(s) with predicted role in transport and nuclear pore localization	YKL068W	No	No
CAGL0F06325g	Protein of unknown function	YKL072W	Yes	No
CAGL0F06567g	Ortholog(s) have translation regulator activity, role in regulation of translation and mitochondrion localization	YMR064W	No	No
CAGL0F06633g	Ortholog(s) have protein complex binding activity and role in ER-associated ubiquitin-dependent protein catabolic process, sporulation resulting in formation of a cellular spore	YMR067C	No	No
CAGL0F06897g	Putative protein with alcohol dehydrogenase domain; gene is downregulated in azole-resistant strain	YIR035C	No	No
CAGL0F07249g	Ortholog(s) have RNA polymerase II activating transcription factor binding, chromatin binding, protein complex scaffold activity	YGL112C	No	No
CAGL0F07403g	Ortholog(s) have role in ribosomal large subunit biogenesis and nucleolus, preribosome, large subunit precursor localization	YGL111W	No	Yes
CAGL0F07601g	Putative GPI-linked cell wall protein	YKL096W-A	No	No
CAGL0F07799g	Ortholog(s) have role in protein targeting to vacuole involved in ubiquitin-dependent protein catabolic process via the multivesicular body sorting pathway and endosome localization	YMR171C	No	No
CAGL0F08393g	Ortholog(s) have role in Golgi to vacuole transport, protein targeting to vacuole and AP-3 adaptor complex localization	YGR261C	No	No
CAGL0F08569g	Ortholog(s) have RSC complex, cytosol localization	YLR321C	No	No
CAGL0F08789g	Ortholog(s) have mitochondrial outer membrane localization	YGR012W	No	No
CAGL0F08965g	Ortholog(s) have role in reciprocal meiotic recombination and cytosol, endoplasmic reticulum, nucleus localization	YHR039C	No	No
CAGL0F09075g	Ortholog(s) have protein serine/threonine kinase activity	YHR205W	No	No
CAGL0G00726g	DNA replication factor A, 69 KD subunit; protein abundance increased in ace2 mutant cells	YAR007C	No	Yes
CAGL0G01056g	Putative glycoside hydrolase of the Gas/Phr family; predicted GPI-anchor	YLR343W	No	No
CAGL0G01122g	Putative protein; gene is upregulated in azole-resistant strain	YLR346C	No	No
CAGL0G01188g	Ortholog(s) have role in cellular sphingolipid homeostasis, negative regulation of sphingolipid biosynthetic process, response to unfolded protein and SPOTS complex localization	YLR350W	No	No
CAGL0G01364g	Ortholog(s) have glycine-tRNA ligase activity, role in glycyl-tRNA aminoacylation and cytosol, mitochondrion localization	YBR121C	Yes	No
CAGL0G01452g	Ortholog(s) have role in ER to Golgi vesicle-mediated transport and ER to Golgi transport vesicle, Golgi apparatus, endoplasmic reticulum localization	YNL043C	No	-
CAGL0G01584g	Ortholog(s) have TFIIIC-class transcription factor binding activity	YNL039W	No	No
CAGL0G01628g	Ortholog(s) have role in single-species biofilm formation on inanimate substrate and cytosol, nucleus localization	YNL035C	Yes	Yes

CAGL0G01991g	Ortholog(s) have endoribonuclease activity, small ribosomal subunit rRNA binding activity and role in maturation of SSU-rRNA from tricistronic rRNA transcript (SSU-rRNA, 5.8S rRNA, LSU-rRNA), proteasome assembly	YOR056C	No	No
CAGL0G02145g	Ortholog(s) have phosphatidylinositol-3-phosphate binding, protein transporter activity and role in protein retention in Golgi apparatus, retrograde transport, endosome to Golgi	YKR078W	No	No
CAGL0G02475g	Ortholog(s) have role in protein ubiquitination, ribosomal large subunit assembly, ribosomal large subunit export from nucleus and cytosol, mitochondrion, nucleolus localization	YIL148W	No	No
CAGL0G03047g	Ortholog(s) have protein serine/threonine kinase activity	YGR092W	No	Yes
CAGL0G03091g	Ortholog(s) have valine-tRNA ligase activity, role in valyl-tRNA aminoacylation and cytosol, mitochondrion localization	YGR094W	No	No
CAGL0G03487g	Ortholog(s) have role in cellular copper ion homeostasis, cellular response to drug, invasive growth in response to glucose limitation, pseudohyphal growth, vacuolar transport and COPI-coated vesicle, Golgi apparatus localization	YER113C	Yes	No
CAGL0G03575g	Has domain(s) with predicted structural constituent of ribosome activity, role in translation and ribosome localization; evidence for a extended second exon by 2 amino acids	YBL087C	Yes	No
CAGL0G03729g	Ortholog(s) have transcription coactivator activity, role in positive regulation of transcription involved in G2/M transition of mitotic cell cycle and nucleus localization	YOR372C	No	No
CAGL0G03773g	Ortholog(s) have structural constituent of nuclear pore activity	YLL023C	Yes	No
CAGL0G04389g	Has domain(s) with predicted sequence-specific DNA binding, sequence-specific DNA binding transcription factor activity, zinc ion binding activity and role in regulation of transcription, DNA-templated	YJL110C	No	No
CAGL0G04763g	Ortholog(s) have GTPase activator activity, role in adenylate cyclase-modulating G-protein coupled receptor signaling pathway and cytoplasm, nucleus localization	YOR107W	No	No
CAGL0G04961g	Ortholog(s) have glutathione-disulfide reductase activity and cytoplasm localization	YLR364W	No	No
CAGL0G05027g	Ortholog(s) have small ribosomal subunit rRNA binding, structural constituent of ribosome activity and role in maturation of SSU-rRNA from tricistronic rRNA transcript (SSU-rRNA, 5.8S rRNA, LSU-rRNA)	YDR064W	No	No
CAGL0G05335g	Ortholog(s) have trehalose-phosphatase activity	YDR074W	No	No
CAGL0G05445g	Ortholog(s) have role in sporulation resulting in formation of a cellular spore and cytoplasm localization	YDL226C	Yes	No
CAGL0G05830g	Protein of unknown function	YHR146W	Yes	No
CAGL0G05940g	Ortholog(s) have cytosolic large ribosomal subunit localization	YDL184C	Yes	No
CAGL0G05984g	Ortholog(s) have role in cell wall assembly and extracellular region localization	YHR139C	No	Yes
CAGL0G06028g	Putative aromatic aminotransferase II	YHR137W	Yes	No
CAGL0G06138g	Ortholog(s) have role in conidium formation, protein phosphorylation, regulation of cell shape, regulation of growth rate and fungal-type vacuole membrane localization	YHR135C	Yes	No
CAGL0G06380g	Ortholog(s) have role in aerobic respiration and mitochondrion, nucleus localization	YOR355W	No	No
CAGL0G06688g	bZIP domain-containing protein	YNL103W	No	No
CAGL0G07359g	Ortholog(s) have protein anchor activity and role in establishment of spindle pole body localization to nuclear envelope, mitotic spindle assembly, nuclear pore organization, spindle pole body duplication	YML031W	No	No
CAGL0G07403g	Has domain(s) with predicted integral component of nuclear inner membrane localization	YML034W	Yes	Yes
CAGL0G07425g	Ortholog(s) have AMP deaminase activity, role in guanine salvage, purine nucleotide metabolic process and cytosol localization	YML035C	Yes	No
CAGL0G07469g	Ortholog(s) have protein transporter activity and role in SRP-dependent cotranslational protein targeting to membrane, cytosol to ER transport, posttranslational protein targeting to membrane, translocation	YOR254C	No	No
CAGL0G07667g	Protein of unknown function	YJL079C	No	No
CAGL0G07953g	Ortholog(s) have structural constituent of ribosome activity and mitochondrial small ribosomal subunit localization	YNR037C	No	No
CAGL0G07975g	Ortholog(s) have role in maturation of 5.8S rRNA from tricistronic rRNA transcript (SSU-rRNA, 5.8S rRNA, LSU-rRNA), maturation of LSU-rRNA from tricistronic rRNA transcript (SSU-rRNA, 5.8S rRNA, LSU-rRNA)	YNR038W	No	No
CAGL0G08173g	Ortholog(s) have protein tag, structural constituent of ribosome activity and role in maturation of SSU-rRNA from tricistronic rRNA transcript (SSU-rRNA, LSU-rRNA,5S), protein ubiquitination, ribosomal small subunit assembly	YLR167W	No	No
CAGL0G09042g	Protein of unknown function	YPL202C	No	No
CAGL0G09713g	Ortholog(s) have cytosol, nucleus localization	YLR287C-A	Yes	No

CAGL0G09779g	Ortholog(s) have role in termination of RNA polymerase II transcription, exosome-dependent, termination of RNA polymerase II transcription, poly(A)-coupled and mRNA cleavage and polyadenylation specificity factor complex localization	YOR179C	No	No
CAGL0G10153g	Ortholog(s) have ubiquinol-cytochrome-c reductase activity and role in aerobic respiration, mitochondrial electron transport, ubiquinol to cytochrome c, mitochondrial respiratory chain complex III assembly	YDR529C	No	Yes
CAGL0H00308g	Ortholog(s) have chromatin binding activity, role in positive regulation of transcription elongation from RNA polymerase II promoter and NuA3b histone acetyltransferase complex, cytosol localization	YLR455W	No	No
CAGL0H00374g	Aspartyl protease that degrades alpha-factor	YLR452C	No	Yes
CAGL0H00616g	Ortholog(s) have transcription coactivator activity, role in histone acetylation, transcription from RNA polymerase II promoter and Ada2/Gcn5/Ada3 transcription activator complex, SAGA complex, SLIK (SAGA-like) complex localization	YPL254W	No	No
CAGL0H00737g	Has domain(s) with predicted intracellular localization	YMR192W	Yes	No
CAGL0H00781g	Ortholog(s) have Golgi apparatus, cytosol, nucleus localization	YPL247C	No	Yes
CAGL0H01023g	Ortholog(s) have RNA-DNA hybrid ribonuclease activity, role in RNA catabolic process and nucleus, ribonuclease H2 complex localization	YDR279W	No	Yes
CAGL0H01397g	Ortholog(s) have proton-transporting ATP synthase activity, rotational mechanism activity, role in ATP synthesis coupled proton transport and mitochondrial proton-transporting ATP synthase, stator stalk localization	YDR298C	Yes	No
CAGL0H01463g	Ortholog(s) have RNA binding activity and role in mRNA cleavage, mRNA polyadenylation, response to drug, termination of RNA polymerase II transcription	YDR301W	No	No
CAGL0H01606g	No Description Available	YDR525W-A	No	Yes
CAGL0H02343g	Ortholog(s) have histone deacetylase activity	YMR263W	No	No
CAGL0H02491g	Ortholog(s) have cytochrome-c oxidase activity, role in mitochondrial electron transport, cytochrome c to oxygen and mitochondrial respiratory chain complex IV, plasma membrane localization	YMR256C	No	No
CAGL0H02783g	Protein of unknown function	YJL076W	Yes	No
CAGL0H03267g	Ortholog(s) have mRNA binding, transcription regulatory region RNA binding activity	YPL190C	No	No
CAGL0H03487g	Ortholog(s) have RNA polymerase II core promoter proximal region sequence-specific DNA binding activity	YGL071W	Yes	No
CAGL0H04125g	Ortholog(s) have D-arabinono-1,4-lactone oxidase activity	YML086C	No	No
CAGL0H04191g	Ortholog(s) have proton-transporting ATP synthase activity, rotational mechanism activity and role in ATP synthesis coupled proton transport, mitochondrial proton-transporting ATP synthase complex assembly, protein complex oligomerization	YML081C-A	No	No
CAGL0H04411g	Ortholog(s) have 3'-5'-exoribonuclease activity, endoribonuclease activity, tRNA binding activity	YOL021C	No	No
CAGL0H04609g	Ortholog(s) have protein serine/threonine kinase activity, telomeric DNA binding activity and role in DNA damage induced protein phosphorylation, double-strand break repair, histone phosphorylation, telomere maintenance	YBL088C	No	No
CAGL0H04691g	Ortholog(s) have structural constituent of ribosome activity and mitochondrial large ribosomal subunit localization	YML009C	No	No
CAGL0H04697g	Ortholog(s) have RNA polymerase I core binding, RNA polymerase I transcription factor binding, RNA polymerase II core binding, core RNA polymerase II binding transcription factor activity, mRNA binding, rRNA binding activity	YML010W	No	No
CAGL0H04873g	Has domain(s) with predicted nucleic acid binding activity	YLR375W	No	No
CAGL0H05071g	Ortholog(s) have SUMO transferase activity, damaged DNA binding, double-stranded DNA-dependent ATPase activity	YLR383W	No	No
CAGL0H05093g	Ortholog(s) have tRNA binding activity, role in regulation of transcription from RNA polymerase II promoter, tRNA wobble uridine modification and Elongator holoenzyme complex, cytosol, nucleus localization	YLR384C	No	No
CAGL0H05159g	Ortholog(s) have mitochondrion targeting sequence binding, protein channel activity and role in protein import into mitochondrial matrix, regulation of mitochondrial membrane permeability	YPL063W	No	No
CAGL0H05203g	Ortholog(s) have role in protein localization to bud neck and cellular bud neck, cytoplasm localization	YPL066W	No	No
CAGL0H05291g	Putative heat shock protein	YPL070W	No	No
CAGL0H05313g	Ortholog(s) have cytoplasm, nucleus localization	YPL071C	Yes	No
CAGL0H05357g	Ortholog(s) have cell cortex localization	YPL074W	No	No

CAGL0H05555g	Ortholog(s) have tRNA-intron endonuclease activity, role in tRNA-type intron splice site recognition and cleavage and mitochondrial outer membrane, tRNA-intron endonuclease complex localization	YPL083C	Yes	No
CAGL0H05577g	Ortholog(s) have protein anchor activity, role in COPII vesicle coating, protein localization to endoplasmic reticulum exit site and ER to Golgi transport vesicle membrane, endoplasmic reticulum exit site localization	YPL085W	Yes	No
CAGL0H05599g	Has domain(s) with predicted hydrolase activity, acting on carbon-nitrogen (but not peptide) bonds, in linear amides activity, role in ceramide metabolic process and integral component of membrane localization	YBR183W	No	No
CAGL0H05709g	Ortholog(s) have role in assembly of large subunit precursor of preribosome, cellular response to drug, rRNA processing, ribosomal large subunit biogenesis, ribosomal subunit export from nucleus	YPL093W	No	No
CAGL0H05797g	Ortholog(s) have role in protein import into mitochondrial inner membrane, protein import into mitochondrial matrix and mitochondrial inner membrane presequence translocase complex, plasma membrane localization	YPL098C	No	No
CAGL0H05929g	Protein of unknown function	YPL105C	No	No
CAGL0H06435g	Ortholog(s) have mitochondrion localization	YLR390W	No	Yes
CAGL0H06479g	Ortholog(s) have role in ribosomal large subunit biogenesis and cytoplasm, cytosolic large ribosomal subunit, preribosome, large subunit precursor localization	YLR387C	Yes	No
CAGL0H06721g	Ortholog(s) have ubiquitin-specific protease activity	YIL156W	Yes	Yes
CAGL0H06743g	Ortholog(s) have role in mitochondrial respiratory chain complex IV assembly and integral component of mitochondrial inner membrane localization	YIL157C	No	No
CAGL0H07023g	Ortholog(s) have proton-transporting ATP synthase activity, rotational mechanism, structural molecule activity and role in ATP synthesis coupled proton transport, cristae formation, protein complex oligomerization	YDR322C-A	Yes	No
CAGL0H07095g	Ortholog(s) have clathrin binding, ubiquitin binding activity	YDR320C	No	No
CAGL0H07667g	Ortholog(s) have 3',5'-cyclic-AMP phosphodiesterase activity, 3',5'-cyclic-GMP phosphodiesterase activity	YGL248W	No	No
CAGL0H08085g	Ortholog(s) have 5'-3' exonuclease activity, 5'-flap endonuclease activity	YOR033C	No	No
CAGL0H08217g	Ortholog(s) have ubiquitin binding activity, role in mitotic DNA integrity checkpoint and condensed nuclear chromosome kinetochore, mitotic checkpoint complex localization	YOR026W	No	No
CAGL0H08541g	Ortholog(s) have DNA binding, bending, nucleosome binding, sequence-specific DNA binding activity	YPR053C	No	-
CAGL0H08734g	Ortholog(s) have cytosolic large ribosomal subunit localization	YJR094W-A	Yes	No
CAGL0H08976g	Ortholog(s) have cytosolic large ribosomal subunit localization	YPL220W	No	No
CAGL0H09108g	Ortholog(s) have cytosol, nucleus localization	YPL260W	Yes	No
CAGL0H09328g	Ortholog(s) have ubiquinol-cytochrome-c reductase activity and role in aerobic respiration, iron-sulfur cluster assembly, mitochondrial electron transport, ubiquinol to cytochrome c	YGR183C	No	Yes
CAGL0H10186g	Ortholog(s) have ubiquitin-specific protease activity, role in negative regulation of gluconeogenesis, proteasome-mediated ubiquitin-dependent protein catabolic process, protein deubiquitination and nucleoplasm localization	YBR058C	No	No
CAGL0I00264g	Has domain(s) with predicted glutathione peroxidase activity and role in oxidation-reduction process, response to oxidative stress; mass spectrometry data support an N-terminal extension of this ORF	YKL026C	Yes	Yes
CAGL0I00572g	Ortholog(s) have proton-transporting ATP synthase activity, rotational mechanism activity and role in ATP synthesis coupled proton transport, cristae formation, mitochondrial proton-transporting ATP synthase complex assembly	YLR295C	No	No
CAGL0I00770g	Ortholog(s) have nucleus localization	YMR144W	No	Yes
CAGL0I00792g	Ortholog(s) have role in maturation of SSU-rRNA from tricistronic rRNA transcript (SSU-rRNA, 5.8S rRNA, LSU-rRNA) and 90S preribosome, cytosol localization	YDL083C	Yes	No
CAGL0I00814g	Ortholog(s) have protein kinase activator activity, structural constituent of ribosome activity, role in cytoplasmic translation, positive regulation of protein kinase activity and cytosolic large ribosomal subunit localization	YDL081C	No	No
CAGL0I00836g	Ortholog(s) have Golgi apparatus, endoplasmic reticulum, fungal-type vacuole membrane localization	YMR140W	Yes	No
CAGL0I00946g	Ortholog(s) have protein serine/threonine kinase inhibitor activity	YOR039W	No	No
CAGL0I00968g	Ortholog(s) have DNA binding, nucleosome binding, transcription corepressor activity	YOR038C	No	No

CAGL0I01408g	Ortholog(s) have electron carrier activity, role in mitochondrial electron transport, cytochrome c to oxygen, mitochondrial electron transport, ubiquinol to cytochrome c and cytosol, mitochondrial intermembrane space, nucleus localization	YJR048W	No	Yes
CAGL0I01430g	Ortholog(s) have cytosol, nucleolus localization	YJR047C	No	No
CAGL0I01474g	Ortholog(s) have DNA replication origin binding activity	YJR046W	Yes	Yes
CAGL0I01518g	Ortholog(s) have role in late endosome to vacuole transport via multivesicular body sorting pathway and Golgi apparatus, Vps55/Vps68 complex, fungal-type vacuole membrane, late endosome localization	YJR044C	No	No
CAGL0I01848g	Protein of unknown function	YHR149C	No	Yes
CAGL0I02200g	Putative 6-phosphogluconolactonase	YGR248W	No	No
CAGL0I02266g	Ortholog(s) have U1 snRNA binding, U2 snRNA binding, U5 snRNA binding, U6 snRNA binding, mRNA binding, pre-mRNA intronic binding, second spliceosomal transesterification activity	YHR165C	No	No
CAGL0I02684g	Ortholog(s) have cytosol, nucleus localization	YMR285C	No	No
CAGL0I03718g	Ortholog(s) have ubiquitin binding activity, role in ER to Golgi vesicle-mediated transport, hyphal growth, nuclear division, retrograde vesicle-mediated transport, Golgi to ER and COPI vesicle coat, hyphal tip localization	YDL145C	No	No
CAGL0I04125g	Ortholog(s) have role in protein N-linked glycosylation and oligosaccharyltransferase complex localization	YGL226C-A	No	No
CAGL0I04862g	Predicted plasma membrane ATP-binding cassette (ABC) transporter, putative transporter involved in multidrug resistance; involved in Pdr1p-mediated azole resistance	YDR011W	Yes	No
CAGL0I05390g	Ortholog(s) have protein serine/threonine kinase activity	YPL026C	No	No
CAGL0I05676g	Ortholog(s) have RNA-dependent ATPase activity, role in generation of catalytic spliceosome for first transesterification step, snoRNA splicing and U2-type catalytic step 1 spliceosome localization	YNR011C	No	No
CAGL0I05852g	Protein of unknown function	YPL256C	No	No
CAGL0I06270g	Ortholog(s) have ubiquinol-cytochrome-c reductase activity, role in aerobic respiration, mitochondrial electron transport, ubiquinol to cytochrome c and mitochondrial respiratory chain complex III, plasma membrane localization	YJL166W	No	No
CAGL0I06534g	Ortholog(s) have ion channel activity, membrane insertase activity, mitochondrial ribosome binding activity	YER154W	No	No
CAGL0I07425g	Ortholog(s) have role in attachment of telomere to nuclear envelope, reciprocal meiotic recombination, regulation of reciprocal meiotic recombination, synapsis and nuclear chromosome, telomeric region localization	YOL104C	No	No
CAGL0I07447g	Ortholog(s) have myo-inositol transmembrane transporter activity, role in carbohydrate import into cell, myo-inositol transport and Golgi apparatus, fungal-type vacuole membrane, plasma membrane localization	YOL103W	Yes	No
CAGL0I07491g	Ortholog(s) have role in cellular zinc ion homeostasis and cytoplasm, nucleus localization	YOL101C	Yes	Yes
CAGL0I07579g	Ortholog(s) have tryptophan-tRNA ligase activity and cytosol localization	YOL097C	No	No
CAGL0I07623g	Ortholog(s) have ATP-dependent 3'-5' DNA helicase activity, role in mitochondrial DNA metabolic process and mitochondrial matrix localization	YOL095C	No	No
CAGL0I07645g	Putative fructose 1,6-bisphosphate aldolase; protein differentially expressed in azole resistant strain	YOL094C	No	No
CAGL0I07733g	Ortholog(s) have ATP binding, ATPase activity, Y-form DNA binding, double-strand/single-strand DNA junction binding, four-way junction DNA binding and guanine/thymine mispair binding, more	YOL090W	No	No
CAGL0I07865g	Ortholog(s) have Golgi apparatus, fungal-type vacuole, plasma membrane localization	YOL084W	Yes	Yes
CAGL0I07969g	Ortholog(s) have role in ATP synthesis coupled proton transport, protein complex oligomerization and mitochondrial proton-transporting ATP synthase complex, coupling factor F(o), plasma membrane localization	YOL077W-A	No	No
CAGL0I07997g	Ortholog(s) have peptide alpha-N-acetyltransferase activity, role in N-terminal peptidyl-methionine acetylation, cytoskeleton organization, mitochondrion inheritance and NatB complex localization	YOL076W	No	No
CAGL0I08019g	Ortholog(s) have fungal-type vacuole membrane localization	YOL075C	No	No
CAGL0I08327g	Ortholog(s) have GTP binding, tRNA binding, translation initiation factor activity, translation initiation factor binding activity	YER025W	No	No
CAGL0I08591g	No Description Available	YER158C	Yes	Yes
CAGL0I08987g	Ortholog(s) have argininosuccinate lyase activity, role in arginine biosynthetic process via ornithine and cytosol localization	YHR018C	Yes	No

CAGL0I09064g	Ortholog(s) have protein serine/threonine phosphatase activity, protein tyrosine/serine/threonine phosphatase activity	YFR028C	Yes	No
CAGL0I09680g	Ortholog(s) have mitochondrion localization	YOR305W	No	No
CAGL0I09768g	Ortholog(s) have role in mRNA splicing, via spliceosome, maturation of 5S rRNA and U4/U6 x U5 tri-snRNP complex localization	YOR308C	No	No
CAGL0I09878g	Ortholog(s) have myristoyl-CoA ligase activity, oleoyl-CoA ligase activity, palmitoyl-CoA ligase activity and role in long-chain fatty acid metabolic process, long-chain fatty-acyl-CoA metabolic process	YMR246W	Yes	Yes
CAGL0I09922g	Ortholog(s) have acetylglucosaminyltransferase activity, role in protein N-linked glycosylation and Golgi medial cisterna localization	YOR320C	No	No
CAGL0I10296g	No Description Available	YPR157W	No	No
CAGL0I10494g	Ortholog(s) have role in eisosome assembly, establishment of protein localization to plasma membrane and Golgi apparatus, eisosome, growing cell tip, plasma membrane localization	YGR131W	Yes	No
CAGL0I10879g	Ortholog(s) have role in peroxisome organization and peroxisomal membrane localization	YPL112C	Yes	Yes
CAGL0I10967g	Ortholog(s) have mRNA binding activity and role in intracellular mRNA localization, mRNA metabolic process, meiotic nuclear division, regulation of mitotic nuclear division	YOR198C	No	No
CAGL0J00319g	Ortholog(s) have inositol monophosphate 1-phosphatase activity, role in inositol phosphate dephosphorylation and cytoplasm, nucleus localization	YHR046C	No	No
CAGL0J00825g	Ortholog(s) have GTPase activator activity and clathrin-coated vesicle, endosome, trans-Golgi network localization	YJL044C	Yes	No
CAGL0J02288g	Ortholog(s) have aspartic-type endopeptidase activity and role in adaptation of signaling pathway by response to pheromone involved in conjugation with cellular fusion, peptide catabolic process	YIL015W	No	No
CAGL0J02442g	Ortholog(s) have chaperone binding, protein binding, bridging activity, role in protein import into mitochondrial matrix and plasma membrane, presequence translocase-associated import motor localization	YIL022W	No	No
CAGL0J02684g	Ortholog(s) have protein binding, bridging activity, role in ergosterol biosynthetic process and endoplasmic reticulum membrane localization	YER044C	No	Yes
CAGL0J02816g	Ortholog(s) have ferrous iron binding, oxidative DNA demethylase activity, peptidyl-proline dioxygenase activity, poly(A) binding activity	YER049W	No	No
CAGL0J03014g	Ortholog(s) have RNA polymerase II regulatory region sequence-specific DNA binding, sequence-specific transcription regulatory region DNA binding RNA polymerase II transcription factor recruiting transcription factor activity	YER064C	No	No
CAGL0J03652g	Ortholog(s) have aminoacyl-tRNA editing activity, leucine-tRNA ligase activity, role in leucyl-tRNA aminoacylation, regulation of TOR signaling and cytosol localization	YPL160W	No	Yes
CAGL0J03828g	Ortholog(s) have MAP kinase kinase activity, protein tyrosine kinase activity, structural constituent of cell wall activity	YOR231W	No	No
CAGL0J04312g	Ortholog(s) have signal sequence binding activity, role in Golgi to vacuole transport, protein targeting to vacuole and Golgi apparatus localization	YNR065C	No	No
CAGL0J04708g	Ortholog(s) have ATP-dependent RNA helicase activity and role in generation of catalytic spliceosome for first transesterification step, spliceosomal complex disassembly	YLR424W	No	No
CAGL0J04796g	Ortholog(s) have cytosol, nucleus localization	YLR427W	Yes	Yes
CAGL0J05236g	Ortholog(s) have mannose-ethanolamine phosphotransferase activity	YJL062W	No	No
CAGL0J05566g	Ortholog(s) have role in cellular response to DNA damage stimulus, interspecies interaction between organisms and cytoplasmic mRNA processing body, cytosol, nucleus, polysome localization	YNL085W	Yes	No
CAGL0J06374g	Ortholog(s) have structural constituent of ribosome activity, role in cytoplasmic translation and cytosolic large ribosomal subunit localization	YDL130W	No	No
CAGL0J06578g	Ortholog(s) have 7S RNA binding activity, role in SRP-dependent cotranslational protein targeting to membrane, signal sequence recognition and cytosol, nucleus, signal recognition particle, endoplasmic reticulum targeting localization	YML105C	No	No
CAGL0J06600g	Orotate phosphoribosyltransferase 1	YMR271C	No	No
CAGL0J06842g	Ortholog(s) have double-stranded DNA binding activity and role in nucleotide-excision repair, phosphorylation of RNA polymerase II C-terminal domain, transcription from RNA polymerase II promoter	YPL122C	No	No
CAGL0J06996g	Ortholog(s) have GTPase activator activity, phosphatidylinositol-3-phosphate binding, phosphatidylinositol-4,5-bisphosphate binding activity	YPL115C	No	Yes

CAGL0J07260g	Ortholog(s) have Rab GTPase binding, Rab geranylgeranyltransferase activity and role in ER to Golgi vesicle-mediated transport, activation of GTPase activity, protein geranylgeranylation, protein targeting to membrane	YOR370C	Yes	Yes
CAGL0J07326g	Ortholog(s) have role in mRNA splicing, via spliceosome, maturation of SSU-rRNA, positive regulation of ATPase activity, positive regulation of helicase activity	YNL224C	Yes	Yes
CAGL0J07436g	Putative ABC transporter	YNL231C	No	No
CAGL0J07546g	Subtilisin-like protease (proprotein convertase) involved in processing of proteins required for cell surface integrity	YNL238W	No	No
CAGL0J07656g	Ortholog(s) have protein binding, bridging activity	YNL243W	Yes	Yes
CAGL0J07744g	Ortholog(s) have cysteine-tRNA ligase activity, role in cysteinyl-tRNA aminoacylation and cytosol, ribosome localization	YNL247W	No	No
CAGL0J07920g	Ortholog(s) have 2-amino-4-hydroxy-6-hydroxymethyldihydropteridine diphosphokinase activity, dihydroneopterin aldolase activity, dihydropteroate synthase activity and role in tetrahydrofolate biosynthetic process	YNL256W	No	No
CAGL0J08272g	Ortholog(s) have glyoxylate reductase activity, role in glyoxylate catabolic process and cytosol, extracellular region, mitochondrion, nucleus localization	YNL274C	Yes	Yes
CAGL0J08657g	Ortholog(s) have protein serine/threonine phosphatase activity and role in cellular response to cadmium ion, cellular response to drug, positive regulation of catalytic activity, protein dephosphorylation	YOR090C	No	No
CAGL0J09372g	Ortholog(s) have hydrolase activity, role in nucleotide metabolic process and cytoplasm, nucleus localization	YDL125C	No	Yes
CAGL0J09504g	Ortholog(s) have methylenetetrahydrofolate reductase (NAD(P)H) activity and role in methionine biosynthetic process, one-carbon metabolic process	YPL023C	No	No
CAGL0J09548g	Ortholog(s) have DNA translocase activity, RNA polymerase II activating transcription factor binding activity	YPL016W	No	No
CAGL0J09900g	Ortholog(s) have extracellular region localization	YNL055C	No	No
CAGL0J10120g	Has domain(s) with predicted sequence-specific DNA binding, sequence-specific DNA binding transcription factor activity and role in regulation of transcription, DNA-templated	YNL068C	No	No
CAGL0J10164g	Putative ribosomal protein	YNL069C	Yes	No
CAGL0J10179g	Ortholog(s) have protein channel activity, role in protein import into mitochondrial matrix, protein import into mitochondrial outer membrane and mitochondrial outer membrane translocase complex localization	YNL070W	No	No
CAGL0J10274g	Ortholog(s) have role in mitochondria-nucleus signaling pathway, negative regulation of transcription from RNA polymerase II promoter, regulation of nitrogen utilization, regulation of pseudohyphal growth and cytoplasm localization	YNL076W	No	No
CAGL0J10406g	Ortholog(s) have enzyme activator activity, phosphatidylinositol-3,5-bisphosphate binding, phosphatidylinositol-3-phosphate binding, tRNA binding activity	YGL105W	No	No
CAGL0J10846g	Ortholog(s) have cyclin-dependent protein serine/threonine kinase regulator activity	YHR071W	Yes	Yes
CAGL0J11242g	Has domain(s) with predicted GTP binding, GTPase activity, role in protein transport, signal transduction, small GTPase mediated signal transduction and intracellular, membrane localization	YNL180C	No	No
CAGL0J11440g	Ortholog(s) have protein binding, bridging, protein transporter activity and role in proteasome localization, protein import into nucleus, protein targeting to membrane	YNL189W	No	No
CAGL0J11638g	Ortholog(s) have enzyme activator activity, protein kinase activity	YMR001C	No	No
CAGL0K00297g	Ortholog(s) have phosphatidylinositol-3,5-bisphosphate 5-phosphatase activity, phosphatidylinositol-3-phosphatase activity, phosphatidylinositol-4-phosphate phosphatase activity	YKL212W	No	No
CAGL0K00473g	Ortholog(s) have Ran GTPase binding activity, role in tRNA re-export from nucleus and cytosol, mitochondrion, nuclear envelope, nuclear matrix localization	YKL205W	No	No
CAGL0K00495g	Ortholog(s) have eukaryotic initiation factor 4E binding, mRNA binding activity and role in deadenylation-dependent decapping of nuclear-transcribed mRNA, negative regulation of translation	YKL204W	No	Yes
CAGL0K00671g	Has domain(s) with predicted structural constituent of ribosome activity, role in translation and ribosome localization	YCR031C	No	No
CAGL0K00803g	Protein described as thioredoxin involved in oxidative stress response; expression upregulated in biofilm vs planktonic cell culture	YGR209C	No	No
CAGL0K00913g	Ortholog(s) have formate-tetrahydrofolate ligase activity, methenyltetrahydrofolate cyclohydrolase activity, methylenetetrahydrofolate dehydrogenase (NADP+) activity, single-stranded DNA binding activity	YGR204W	No	No

CAGL0K00935g	Ortholog(s) have phosphatase activity, thiosulfate sulfurtransferase activity, role in dephosphorylation and cytosol, nucleus localization	YGR203W	No	No
CAGL0K02123g	Ortholog(s) have role in rRNA export from nucleus and cytosol localization	YGL189C	Yes	No
CAGL0K02167g	Ortholog(s) have protein serine/threonine kinase activity, role in glucose metabolic process, protein phosphorylation and cytoplasm localization	YGL179C	No	No
CAGL0K02409g	Ortholog(s) have proton-transporting ATP synthase activity, rotational mechanism activity and role in ATP synthesis coupled proton transport, cristae formation, protein complex oligomerization	YPR020W	No	No
CAGL0K02893g	Ortholog(s) have ubiquinol-cytochrome-c reductase activity, role in aerobic respiration, mitochondrial electron transport, ubiquinol to cytochrome c and mitochondrial respiratory chain complex III localization	YHR001W-A	No	No
CAGL0K03003g	Ortholog(s) have RNA polymerase II activating transcription factor binding and RNA polymerase II core promoter proximal region sequence-specific DNA binding, more	YMR070W	No	Yes
CAGL0K03465g	Ortholog(s) have acetolactate synthase activity, flavin adenine dinucleotide binding activity, role in isoleucine biosynthetic process, valine biosynthetic process and acetolactate synthase complex, mitochondrion localization	YMR108W	Yes	No
CAGL0K03487g	Ortholog(s) have role in actin cortical patch assembly, actin cortical patch localization, actin nucleation, ascospore wall assembly, cell tip growth and endocytosis, more	YMR109W	No	No
CAGL0K03619g	Ortholog(s) have structural constituent of cytoskeleton activity, role in chromosome segregation, microtubule nucleation and Ndc80 complex, condensed nuclear chromosome kinetochore localization	YMR117C	No	Yes
CAGL0K03729g	Ortholog(s) have mRNA binding activity	YMR125W	No	No
CAGL0K03861g	Ortholog(s) have role in ribosome biogenesis and cytosol, nucleolus localization	YMR131C	No	No
CAGL0K03905g	Ortholog(s) have role in reciprocal meiotic recombination and condensed nuclear chromosome localization	YMR133W	No	No
CAGL0K03971g	Ortholog(s) have role in protein targeting to mitochondrion and mitochondrial outer membrane, peroxisomal membrane localization	YGR028W	No	Yes
CAGL0K04125g	Ortholog(s) have long-chain fatty acid transporter activity, role in chronological cell aging, long-chain fatty acid transport and cytosol, extracellular region, nucleus localization	YGR037C	Yes	No
CAGL0K04169g	Ortholog(s) have MAP kinase activity, transcription factor binding activity	YGR040W	No	No
CAGL0K04543g	Ortholog(s) have rDNA binding activity	YGR063C	No	No
CAGL0K04543g	Ortholog(s) have rDNA binding activity	YGR064W	No	-
CAGL0K04587g	Ortholog(s) have structural constituent of ribosome activity and cytosolic small ribosomal subunit, nucleus localization	YLR367W	No	No
CAGL0H04873g	Ortholog(s) have sequence-specific DNA binding activity, role in filamentous growth and nucleus localization	YDL048C	No	Yes
CAGL0K04917g	Putative transcriptional regulator, complements lethality of S. cerevisiae rap1 mutant	YNL216W	No	No
CAGL0K04939g	Ortholog(s) have fungal-type vacuole localization	YNL217W	No	Yes
CAGL0K05049g	Ortholog(s) have ribonuclease MRP activity, ribonuclease P activity, tRNA binding activity and role in intronic box C/D snoRNA processing, mRNA cleavage, rRNA processing, tRNA processing	YNL221C	No	No
CAGL0K05093g	Ortholog(s) have ATP-dependent peptidase activity	YPR024W	Yes	No
CAGL0K05203g	Ortholog(s) have role in cellular protein localization, endoplasmic reticulum inheritance, endoplasmic reticulum tubular network organization, mitotic cytokinesis, site selection, nuclear pore complex assembly, vesicle-mediated transport	YPR028W	No	Yes
CAGL0K05269g	Ortholog(s) have methylated histone binding activity, role in histone acetylation and NuA3a histone acetyltransferase complex, NuA3b histone acetyltransferase complex, cytoplasm localization	YPR031W	No	No
CAGL0K05335g	Ortholog(s) have DNA translocase activity	YPR034W	Yes	No
CAGL0K05445g	Ortholog(s) have role in centromere complex assembly, establishment of protein localization to chromosome, kinetochore organization and maintenance of meiotic sister chromatid cohesion, more	YOR073W	No	Yes
CAGL0K05489g	Ortholog(s) have SNAP receptor activity, role in endoplasmic reticulum membrane fusion, retrograde vesicle-mediated transport, Golgi to ER and SNARE complex, endoplasmic reticulum localization	YOR075W	No	Yes
CAGL0K05813g	Ortholog(s) have glutathione disulfide oxidoreductase activity, glutathione peroxidase activity, glutathione transferase activity and role in glutathione metabolic process, pathogenesis, removal of superoxide radicals	YDR513W	No	No
CAGL0K05995g	Putative sphingoid long-chain base kinase; gene is upregulated in azole-resistant strain	YLR260W	Yes	Yes

CAGL0K06061g	Ortholog(s) have structural constituent of ribosome activity	YLR264W	No	No
CAGL0K06325g	Ortholog(s) have role in attachment of telomere to nuclear envelope, maintenance of rDNA, mitotic sister chromatid segregation, nuclear inner membrane organization	YDR458C	No	No
CAGL0K08360g	Ortholog(s) have double-stranded DNA 5'-3' exodeoxyribonuclease activity, single-stranded DNA endodeoxyribonuclease activity, tRNA (m5U54) methyltransferase activity and role in double-strand break repair, tRNA modification	YKR056W	Yes	No
CAGL0K08382g	Ortholog(s) have structural constituent of ribosome activity	YKR057W	No	Yes
CAGL0K08558g	Ortholog(s) have role in establishment or maintenance of cell polarity and cytosol localization	YMR084W	No	No
CAGL0K08800g	Ortholog(s) have acireductone dioxygenase (Ni2+-requiring) activity, role in L-methionine biosynthetic process from methylthioadenosine and cytosol, nucleus localization	YMR009W	No	Yes
CAGL0K08844g	Ortholog(s) have gamma-butyrobetaine dioxygenase activity, role in carnitine biosynthetic process, mitochondrion organization and mitochondrion localization	YHL021C	No	Yes
CAGL0K08888g	Ortholog(s) have guanyl-nucleotide exchange factor activity, translation initiation factor activity and role in negative regulation of cellular response to amino acid starvation, regulation of translational initiation	YOR260W	No	No
CAGL0K08976g	Has domain(s) with predicted peptidase activity and role in proteolysis	YOR256C	No	No
CAGL0K09504g	Ortholog(s) have ATPase activator activity, role in mRNA splicing, via spliceosome, positive regulation of ATPase activity and spliceosomal complex localization	YOR148C	Yes	No
CAGL0K09614g	Ortholog(s) have role in negative regulation of G0 to G1 transition, ribosomal small subunit biogenesis and 90S preribosome, nucleolus localization	YNL132W	No	No
CAGL0K09724g	Peptidyl-prolyl cis-trans isomerase; expression upregulated in biofilm vs planktonic cell culture	YNL135C	No	No
CAGL0K09966g	Translation initiation factor eIF3 subunit	YOR361C	No	No
CAGL0K10054g	Ortholog(s) have RNA polymerase II core promoter proximal region sequence-specific DNA binding activity	YDR081C	No	No
CAGL0K10626g	Ortholog(s) have glycogen (starch) synthase activity	YFR015C	No	Yes
CAGL0K10868g	Putative catalase A; gene is downregulated in azole-resistant strain; regulated by oxidative stress and glucose starvation	YGR088W	No	No
CAGL0K10906g	Ortholog(s) have RNA binding, structural constituent of ribosome activity, role in cytoplasmic translation and cytosolic large ribosomal subunit localization	YPL249C-A	Yes	No
CAGL0K10956g	Ortholog(s) have role in mitochondrial respiratory chain complex IV assembly, negative regulation of mitochondrial translation	YML129C	No	No
CAGL0K10978g	Ortholog(s) have homoaconitate hydratase activity, role in lysine biosynthetic process via aminoadipic acid and mitochondrion localization	YDR234W	No	Yes
CAGL0K11088g	Ortholog(s) have role in ER to Golgi vesicle-mediated transport and COPI vesicle coat, cytosol, plasma membrane localization	YDR238C	No	No
CAGL0K11440g	Ortholog(s) have role in DNA damage induced protein phosphorylation, DNA repair, protein localization to kinetochore, signal transduction in response to DNA damage, transfer RNA gene-mediated silencing	YDR225W	Yes	No
CAGL0K11506g	Ortholog(s) have 5'-flap endonuclease activity, single-stranded DNA 5'-3' exodeoxyribonuclease activity	YKL113C	No	Yes
CAGL0K12914g	Ortholog(s) have glycoprotein binding activity, role in ER to Golgi vesicle-mediated transport and ER to Golgi transport vesicle, Golgi membrane localization	YFL048C	No	No
CAGL0L00429g	Ortholog(s) have glycine dehydrogenase (decarboxylating) activity, role in glycine decarboxylation via glycine cleavage system, one-carbon metabolic process and mitochondrion localization	YMR189W	Yes	No
CAGL0L00495g	Putative heat shock protein	YMR186W	No	No
CAGL0L00803g	Ortholog(s) have role in regulation of glycogen biosynthetic process and cytoplasm localization	YIL045W	Yes	Yes
CAGL0L00935g	Ortholog(s) have role in cellular lipid metabolic process, mRNA export from nucleus, nuclear envelope organization and endoplasmic reticulum, nuclear envelope localization	YIL040W	No	No
CAGL0L01309g	Ortholog(s) have damaged DNA binding, peptide-N4-(N-acetyl-beta-glucosaminyl)asparagine amidase activity, proteasome binding, protein binding, bridging, ubiquitin binding activity	YEL037C	No	No
CAGL0L01353g	Ortholog(s) have cytosol, nucleolus localization	YEL034W	Yes	No
CAGL0L02057g	Ortholog(s) have RNA polymerase II core binding, TFIIH-class transcription factor binding, single-stranded DNA binding activity and role in transcriptional open complex formation at RNA polymerase II promoter	YKL028W	No	No
CAGL0L02673g	Ortholog(s) have DNA clamp loader activity, role in leading strand elongation, mismatch repair, mitotic DNA replication and DNA replication factor C complex, Elg1 RFC-like complex, nucleus localization	YOR217W	No	Yes
CAGL0L02871g	Ortholog(s) have mRNA binding activity	YOR206W	No	No

CAGL0L03960g	Ortholog(s) have ubiquitin-protein transferase activity	YNL116W	Yes	No
CAGL0L04004g	Ortholog(s) have chromatin binding, m7G(5')pppN diphosphatase activity, mRNA binding activity	YNL118C	Yes	No
CAGL0L04092g	Ortholog(s) have serine-type peptidase activity, role in apoptotic process, cellular lipid metabolic process, cellular response to heat, protein catabolic process and nucleus localization	YNL123W	No	No
CAGL0L04136g	Ortholog(s) have structural constituent of cytoskeleton activity	YNL126W	No	No
CAGL0L04158g	Ortholog(s) have role in ascospore formation, intra-S DNA damage checkpoint, positive regulation of macroautophagy, re-entry into mitotic cell cycle after pheromone arrest and regulation of meiosis I, more	YNL127W	No	No
CAGL0L04818g	Ortholog(s) have cytoplasm localization	YGR117C	No	No
CAGL0L05522g	Ortholog(s) have COPI-coated vesicle, Golgi apparatus, cytosol, ribosome localization	YJL123C	No	Yes
CAGL0L06160g	Ortholog(s) have cytochrome-c oxidase activity, zinc ion binding activity and role in aerobic respiration, mitochondrial electron transport, cytochrome c to oxygen	YGL187C	No	No
CAGL0L06292g	Ortholog(s) have role in vesicle-mediated transport and COPI-coated vesicle, integral component of Golgi membrane localization	YDR100W	No	Yes
CAGL0L06402g	Ortholog(s) have bilirubin transmembrane transporter activity, glutathione S-conjugate-exporting ATPase activity, phytochelatin transmembrane transporter activity	YDR135C	No	No
CAGL0L07480g	Ortholog(s) have RNA polymerase II core promoter proximal region sequence-specific DNA binding, more	YBR066C	No	No
CAGL0L07634g	No Description Available	YML002W	No	No
CAGL0L08404g	Ortholog(s) have role in mRNA cis splicing, via spliceosome and U2-type spliceosomal complex, cytosol localization	YPR152C	No	No
CAGL0L09603g	Ortholog(s) have role in attachment of spindle microtubules to kinetochore involved in meiotic sister chromatid segregation and nuclear MIS12/MIND complex, spindle pole localization	YIR010W	No	Yes
CAGL0L09955g	No Description Available	YOR043W	Yes	No
CAGL0L10015g	Ortholog(s) have protein channel activity, role in mitochondrial outer membrane translocase complex assembly, protein import into mitochondrial matrix and mitochondrial outer membrane translocase complex localization	YOR045W	No	No
CAGL0L10120g	Ortholog(s) have 5'-3' exoribonuclease activity, mRNA binding, microtubule binding, recombinase activity	YOR048C	No	No
CAGL0L10164g	Ortholog(s) have role in translational termination and nucleus localization	YOR051C	No	No
CAGL0L10252g	Ortholog(s) have microtubule binding activity and role in cell separation after cytokinesis, microtubule bundle formation, mitotic spindle elongation, mitotic spindle stabilization, spindle midzone assembly, spindle pole body separation	YOR058C	No	No
CAGL0L10428g	Protein of unknown function	YOR066W	No	Yes
CAGL0L11110g	Catalytic subunit of calcineurin, calcium/calmodulin-dependent Ser/Thr-specific protein phosphatase; regulates stress-responding transcription factor Crz1p; involved in thermotolerance, response to ER stress, cell wall integrity, virulence	YLR433C	No	No
CAGL0L11132g	Protein of unknown function	YLR437C	Yes	Yes
CAGL0L11616g	Ortholog(s) have cytoplasm localization	YOR118W	No	No
CAGL0L12166g	Ortholog(s) have nucleosome binding activity, role in chromatin silencing at telomere, histone exchange and Swr1 complex, cytosol, nuclear chromosome, telomeric region localization	YLR085C	No	No
CAGL0L12408g	Ortholog(s) have tRNA (uracil) methyltransferase activity, role in tRNA methylation and cytosol, nucleus localization	YPL030W	No	No
CAGL0L12452g	Has domain(s) with predicted NADP binding, coenzyme binding, oxidoreductase activity, oxidoreductase activity, acting on the CH-OH group of donors, NAD or NADP as acceptor activity and role in oxidation-reduction process	YPL032C	Yes	No
CAGL0L12650g	Ortholog(s) have RNA polymerase II carboxy-terminal domain kinase activity, cyclin-dependent protein serine/threonine kinase activity	YPL042C	No	No
CAGL0L12672g	Ortholog(s) have mRNA binding activity, role in cellular response to drug, maturation of LSU-rRNA from tricistronic rRNA transcript (SSU-rRNA, 5.8S rRNA, LSU-rRNA) and nucleolus, preribosome, large subunit precursor localization	YPL043W	No	No
CAGL0L12672g	Ortholog(s) have mRNA binding activity, role in cellular response to drug, maturation of LSU-rRNA from tricistronic rRNA transcript (SSU-rRNA, 5.8S rRNA, LSU-rRNA) and nucleolus, preribosome, large subunit precursor localization	YPL044C	No	-
CAGL0L12694g	Ortholog(s) have phosphatidylinositol binding activity	YPL045W	No	No
CAGL0L12870g	Ortholog(s) have role in cellular response to oxidative stress, cytoplasmic translation and cytosol, mitochondrion, nucleus, ribosome localization	YKL056C	No	No
CAGL0L12936g	Ortholog(s) have mitochondrion localization	YHR117W	Yes	No

CAGL0L12980g	Ortholog(s) have RNA binding, histone methyltransferase activity (H3-K4 specific) activity	YHR119W	No	No
CAGL0L13112g	Ortholog(s) have RNA polymerase II repressing transcription factor binding activity and role in negative regulation of transcription from RNA polymerase II promoter, positive regulation of transcription from RNA polymerase II promoter	YLR071C	No	No
CAGL0M00418g	Ortholog(s) have protein transporter activity, role in protein import into mitochondrial inner membrane and cytosol, mitochondrial intermembrane space protein transporter complex localization	YJR135W-A	No	No
CAGL0M00506g	Ortholog(s) have protein transporter activity, role in protein import into nucleus and cytosol, nuclear envelope, nuclear periphery localization	YJR132W	No	No
CAGL0M01892g	No Description Available	YBR076W	Yes	No
CAGL0M01936g	Ortholog(s) have cytosol, fungal-type vacuole membrane localization	YBR075W	No	-
CAGL0M02277g	Ortholog(s) have role in mRNA splicing, via spliceosome and Prp19 complex, spliceosomal complex localization	YPL151C	No	No
CAGL0M02409g	Ortholog(s) have rRNA binding activity, role in maturation of LSU-rRNA from tricistronic rRNA transcript (SSU-rRNA, 5.8S rRNA, LSU-rRNA), ribosomal large subunit export from nucleus and nucleolus localization	YPL146C	No	No
CAGL0M02497g	Ortholog(s) have structural constituent of ribosome activity, role in cytoplasmic translation and cytosolic large ribosomal subunit localization	YOR234C	Yes	No
CAGL0M02497g	Ortholog(s) have structural constituent of ribosome activity, role in cytoplasmic translation and cytosolic large ribosomal subunit localization	YPL142C	Yes	-
CAGL0M02519g	Ortholog(s) have protein kinase activity, role in protein phosphorylation and cytoplasm localization	YPL141C	No	Yes
CAGL0M02959g	Ortholog(s) have CTD phosphatase activity and role in dephosphorylation of RNA polymerase II C-terminal domain, negative regulation of G0 to G1 transition, regulation of mitotic cytokinesis	YMR277W	No	No
CAGL0M03003g	Ortholog(s) have role in borate transmembrane transport and endoplasmic reticulum localization	YMR279C	No	Yes
CAGL0M03531g	Ortholog(s) have role in ER to Golgi vesicle-mediated transport, retrograde vesicle-mediated transport, Golgi to ER and COPI vesicle coat, cytosol, endosome localization	YNL287W	No	No
CAGL0M03597g	Putative calcium transporter; putative regulatory subunit of a plasma membrane gated channel involved in Ca2+ uptake; required for viability upon prolonged fluconazole stress	YNL291C	No	No
CAGL0M03729g	Ortholog(s) have role in ascospore formation, conidiophore development, conidium formation, hyphal growth, sporocarp development involved in sexual reproduction and mitochondrion localization	YNL298W	No	No
CAGL0M03971g	Ortholog(s) have role in cellular protein catabolic process, regulation of sulfur amino acid metabolic process and SCF ubiquitin ligase complex, ribosome localization	YNL311C	No	No
CAGL0M04235g	Ortholog(s) have ubiquitin-protein transferase activity	YLR127C	No	No
CAGL0M04279g	Ortholog(s) have snoRNA binding activity	YLR129W	No	No
CAGL0M04323g	Putative transcription factor; null mutation results in hypervirulence in immunocompromised mice	YLR131C	No	Yes
CAGL0M04631g	Putative spliceosome component; gene is upregulated in azole-resistant strain	YLR147C	Yes	No
CAGL0M04675g	Ortholog(s) have thiosulfate sulfurtransferase activity and endoplasmic reticulum, mitochondrial outer membrane localization	YOR285W	No	Yes
CAGL0M04829g	Ortholog(s) have mRNA binding, poly(U) RNA binding activity	YMR229C	No	Yes
CAGL0M04895g	Ortholog(s) have phosphatidylinositol binding, ubiquitin-protein transferase activity	YMR231W	No	No
CAGL0M04961g	Ortholog(s) have GTPase activator activity	YMR235C	No	No
CAGL0M05907g	Ortholog(s) have role in cellular response to oleic acid, negative regulation of transcription from RNA polymerase II promoter and mitochondrion, nucleus localization	YKR064W	No	No
CAGL0M05929g	Ortholog(s) have role in protein import into mitochondrial matrix and plasma membrane, presequence translocase-associated import motor localization	YKR065C	No	No
CAGL0M06083g	Heat shock protein of HSP70 family	YBR169C	No	No
CAGL0M06237g	Ortholog(s) have alcohol O-octanoyltransferase activity, short-chain carboxylesterase activity and role in medium-chain fatty acid biosynthetic process	YPL095C	Yes	No
CAGL0M06369g	Ortholog(s) have histone acetyltransferase activity, role in regulation of transcription from RNA polymerase II promoter, tRNA wobble uridine modification and Elongator holoenzyme complex, cytosol, nucleus localization	YPL086C	No	No
CAGL0M06413g	Ortholog(s) have deubiquitinase activator activity	YPL084W	No	No
CAGL0M06633g	Ortholog(s) have mitochondrion localization	YIL087C	No	No
CAGL0M06787g	Ortholog(s) have cytosol, nucleus localization	YIL085C	No	No

CAGL0M06919g	Ortholog(s) have Golgi apparatus, cytosol localization	YNL023C	No	No
CAGL0M07001g	Protein of unknown function	YNR034W-A	No	No
CAGL0M07051g	Ortholog(s) have exoribonuclease II activity, role in mitochondrial RNA catabolic process and mitochondrial degradosome localization	YMR287C	No	No
CAGL0M07227g	Ortholog(s) have snoRNA binding activity and role in maturation of SSU-rRNA from tricistronic rRNA transcript (SSU-rRNA, 5.8S rRNA, LSU-rRNA), positive regulation of transcription from RNA polymerase I promoter	YHR196W	No	No
CAGL0M07271g	Ortholog(s) have glutathione disulfide oxidoreductase activity, role in cellular response to oxidative stress, response to osmotic stress and mitochondrial matrix localization	YPL059W	No	No
CAGL0M07337g	Ortholog(s) have role in histone methylation, histone ubiquitination, premeiotic DNA replication, protein monoubiquitination, regulation of cell size and nucleus localization	YPL055C	No	Yes
CAGL0M07722g	Ortholog(s) have mRNA binding activity, role in cytoplasmic translational initiation and eukaryotic translation initiation factor 3 complex localization	YMR012W	No	Yes
CAGL0M08074g	Ortholog(s) have role in hexose catabolic process	YJR030C	No	No
CAGL0M08514g	Pir protein family member, predicted GPI-anchor	YOL105C	No	No
CAGL0M08668g	Ortholog(s) have ubiquitin-protein transferase activity, role in histone catabolic process, histone ubiquitination and cytoplasm, ribosome localization	YDR266C	No	No
CAGL0M08712g	Ortholog(s) have Golgi apparatus, fungal-type vacuole membrane localization	YDR264C	No	No
CAGL0M08756g	Putative exo-1,3-beta-glucanase; predicted GPI-anchor	YDR261C	No	No
CAGL0M09229g	Ortholog(s) have endoplasmic reticulum, mitochondrion localization	YJR085C	No	No
CAGL0M10241g	Ortholog(s) have RNA binding activity and cytosolic large ribosomal subunit, nucleus localization	YHL001W	Yes	No
CAGL0M10571g	Ortholog(s) have ergosterol O-acyltransferase activity, role in ergosterol metabolic process and endoplasmic reticulum localization	YCR050C	No	No
CAGL0M10703g	Ortholog(s) have Sar guanyl-nucleotide exchange factor activity, role in regulation of COPII vesicle coating and Golgi apparatus, endoplasmic reticulum localization	YNR026C	No	No
CAGL0M10934g	Ortholog(s) have 4-amino-4-deoxychorismate synthase activity, role in para-aminobenzoic acid biosynthetic process and cytosol, nucleus localization	YNR033W	No	No
CAGL0M11264g	Ortholog(s) have role in RNA polymerase II transcriptional preinitiation complex assembly and transcription factor TFIID complex localization	YMR227C	No	No
CAGL0M11396g	Ortholog(s) have protein serine/threonine kinase activity	YLR096W	No	No
CAGL0M11440g	Ortholog(s) have sequence-specific DNA binding, sequence-specific DNA binding transcription factor activity, role in cellular amino acid catabolic process, positive regulation of transcription, DNA-templated and nucleus localization	YLR098C	No	No
CAGL0M11462g	Ortholog(s) have lysophosphatidic acid acyltransferase activity and role in phosphatidic acid biosynthetic process	YLR099C	Yes	Yes
CAGL0M11726g	Putative GPI-linked cell wall adhesin-like protein	YLR110C	Yes	No
CAGL0M11726g	Putative GPI-linked cell wall adhesin-like protein	YDR133C	Yes	-
CAGL0M12386g	Ortholog(s) have role in isoleucine biosynthetic process, mitochondrial genome maintenance, mitochondrial translation and cytosol, mitochondrial matrix, nucleus localization	YER057C	No	No
CAGL0M12430g	Putative DL-glycerol phosphatase	YER062C	No	Yes
CAGL0M12705g	Ortholog(s) have GTPase regulator activity, mRNA binding activity	YER072W	Yes	No
CAGL0M12793g	Ortholog(s) have cytoplasm, nucleus localization	YER079W	No	No
CAGL0M13255g	Ortholog(s) have ribosome localization	YKL065C	No	No
CAGL0M13255g	Ortholog(s) have ribosome localization	YMR040W	No	No
CAGL0M13519g	Ortholog(s) have ATP-dependent RNA helicase activity, RNA binding activity	YMR290C	Yes	Yes
CAGL0M13871g	Ortholog(s) have nuclear localization sequence binding, protein transporter activity and role in mRNA export from nucleus, protein import into nucleus, regulation of mitotic nuclear division, regulation of protein desumoylation	YMR308C	No	No
CAGL0M13893g	Ortholog(s) have mRNA binding, translation initiation factor activity, translation initiation factor binding activity and role in cellular response to drug, translational initiation	YMR309C	No	No

Acetic acid-responsive genes regulated by CgHaa1 only

ORF	Function	*S. cerevisiae* orthologue	*C. glabrata* promoter harbors an HRE motif	*S. cerevisiae* promoter harbors an HRE motif

CAGL0A00495g	Putative plasma membrane proton pump with a predicted role in pH homeostasis	YGL008C	No	No
CAGL0A00539g	Ortholog(s) have role in CVT pathway, intra-Golgi vesicle-mediated transport and Golgi transport complex, mitochondrion localization	YGL005C	No	No
CAGL0A01650g	Putative protein; gene is upregulated in azole-resistant strain	YGR146C	Yes	No
CAGL0A01716g	Ortholog(s) have nicotinamidase activity, role in chromatin silencing at rDNA, chromatin silencing at telomere, replicative cell aging and cytosol, nucleus, peroxisome localization	YGL037C	No	Yes
CAGL0A01782g	Ortholog(s) have glucose transmembrane transporter activity, pentose transmembrane transporter activity and plasma membrane localization	YHR092C	Yes	No
CAGL0A01804g	Ortholog(s) have fructose transmembrane transporter activity, pentose transmembrane transporter activity, role in glucose transport, mannose transport and plasma membrane localization	YHR094C	No	No
CAGL0A01870g	Has domain(s) with predicted integral to membrane localization	YBL017C	No	Yes
CAGL0A02145g	Ortholog(s) have actin filament binding activity, role in actin cortical patch localization, actin filament bundle assembly, endocytosis and actin cortical patch localization	YHR016C	No	No
CAGL0A03102g	Ortholog(s) have phenylpyruvate decarboxylase activity	YDR380W	Yes	Yes
CAGL0A03872g	Ortholog(s) have phosphatidylinositol-4,5-bisphosphate binding activity, role in actin cortical patch assembly, actin filament organization, endocytosis and actin cortical patch, mating projection tip localization	YLR206W	No	Yes
CAGL0B00264g	Homeobox-domain protein alpha2	YCL067C	No	No
CAGL0B00902g	Phosphoribosyl-AMP cyclohydrolase; phosphoribosyl-ATP pyrophosphatase; histidinol dehydrogenase	YCL030C	No	No
CAGL0B01265g	Mating-type regulatory protein alpha2; expressed in all MTLalpha strains and not in MTLa strains	YCR039C	No	No
CAGL0B01595g	No Description Available	-	Yes	-
CAGL0B01875g	Ortholog(s) have mitochondrial inner membrane localization	YDR119W-A	No	Yes
CAGL0B02431g	Ortholog(s) have NADH dehydrogenase (ubiquinone) activity and role in NADH oxidation, chronological cell aging, mitochondrial electron transport, NADH to ubiquinone, positive regulation of apoptotic process	YML120C	No	Yes
CAGL0B02475g	Ortholog(s) have inorganic phosphate transmembrane transporter activity, manganese ion transmembrane transporter activity and role in manganese ion transport, phosphate ion transport, polyphosphate metabolic process	YML123C	Yes	Yes
CAGL0B02755g	No Description Available	YLR361C-A	No	Yes
CAGL0B03421g	Has domain(s) with predicted DNA binding, sequence-specific DNA binding RNA polymerase II transcription factor activity, zinc ion binding activity and role in regulation of transcription, DNA-dependent, transcription, DNA-dependent	YLR256W	Yes	No
CAGL0B03817g	Ortholog(s) have cytosol, nucleus localization	YJR008W	No	No
CAGL0B04675g	No Description Available	YCL001W-B	No	No
CAGL0B04895g	Ortholog(s) have RNA polymerase II core promoter proximal region sequence-specific DNA binding activity	YLR176C	No	Yes
CAGL0C00275g	Putative cysteine protease; protein differentially expressed in azole resistant strain; gene is upregulated in azole-resistant strain	YDR533C	No	Yes
CAGL0C00319g	Ortholog(s) have carboxypeptidase activity, role in nitrogen compound metabolic process, proteolysis involved in cellular protein catabolic process and fungal-type vacuole lumen localization	YJL172W	No	No
CAGL0C01397g	Has domain(s) with predicted 6-phosphofructo-2-kinase activity, ATP binding, catalytic activity and role in fructose 2,6-bisphosphate metabolic process, fructose metabolic process	YIL107C	Yes	Yes
CAGL0C01551g	Has domain(s) with predicted DNA binding, sequence-specific DNA binding, sequence-specific DNA binding transcription factor activity and role in regulation of transcription, DNA-dependent	YPL177C	No	Yes
CAGL0C01595g	Ortholog(s) have imidazoleglycerol-phosphate synthase activity, role in histidine biosynthetic process and cytosol, nucleus localization	YBR248C	No	No
CAGL0C01771g	Ortholog(s) have role in protein targeting to vacuole and mitochondrion localization	YGL104C	No	No
CAGL0C02365g	No Description Available	-	No	-
CAGL0C02519g	Has domain(s) with predicted nucleic acid binding activity	YER028C	Yes	No
CAGL0C03267g	Glycerol transporter; 1 of 2 Fps1 orthologs in C. glabrata; double fps1/fps2 mutant accumulates glycerol, has constitutive cell wall stress, is hypersensitive to caspofungin in vitro and in vivo	YLL043W	Yes	Yes
CAGL0C03289g	Putative ABC transporter involved in bile acid transport; gene is upregulated in azole-resistant strain	YLL048C	Yes	No

CAGL0C03740g	Ortholog(s) have sequence-specific DNA binding, sequence-specific DNA binding transcription factor activity	YEL007W	Yes	Yes
CAGL0C03872g	Putative GPI-linked cell wall protein involved in sterol uptake	YIL011W	Yes	Yes
CAGL0C04213g	Ortholog(s) have role in vesicle-mediated transport and fungal-type vacuole, vesicle localization	YDR003W	No	No
CAGL0C04323g	Ortholog(s) have alpha,alpha-trehalase activity, role in trehalose catabolic process and cytoplasm localization	YDR001C	No	Yes
CAGL0C04785g	No Description Available	YJR115W	Yes	Yes
CAGL0C05027g	Has domain(s) with predicted transferase activity, transferring acyl groups activity	YAR035W	Yes	Yes
CAGL0C05533g	Has domain(s) with predicted phosphoric diester hydrolase activity and role in lipid metabolic process	YDL237W	No	No
CAGL0D00286g	Beta mannosyltransferase	-	No	-
CAGL0D01100g	Ortholog(s) have 6-phosphofructo-2-kinase activity and role in fructose 2,6-bisphosphate metabolic process	YIL107C	No	Yes
CAGL0D01276g	Ortholog(s) have role in cell cycle arrest in response to pheromone, establishment of protein localization to plasma membrane, fungal-type cell wall biogenesis and osmosensory signaling pathway, more	YPR075C	No	No
CAGL0D01298g	Putative transketolase	YPR074C	Yes	Yes
CAGL0D01474g	Ortholog(s) have role in actin cortical patch assembly and actin cortical patch, membrane raft localization	YBR108W	No	No
CAGL0D01980g	Ortholog(s) have sterol esterase activity, role in cellular lipid metabolic process, sterol metabolic process and integral to membrane, lipid particle localization	YKL140W	No	No
CAGL0D02948g	Protein with a predicted role in nuclear fusion	YJL034W	Yes	Yes
CAGL0D04026g	Ortholog(s) have 4-aminobutyrate transaminase activity, pyridoxal phosphate binding activity, role in gamma-aminobutyric acid catabolic process, glutamate metabolic process and cytosol localization	YGR019W	No	No
CAGL0D04422g	Ortholog(s) have endoplasmic reticulum localization	YPL279C	Yes	Yes
CAGL0D04510g	No Description Available	YPR117W	Yes	No
CAGL0D05566g	Ortholog(s) have sterol esterase activity, role in cell wall mannoprotein biosynthetic process, sterol metabolic process and integral to membrane, plasma membrane localization	YLR020C	Yes	No
CAGL0E01353g	Putative high-affinity zinc transporter; gene is downregulated in azole-resistant strain	YLR130C	Yes	Yes
CAGL0E01419g	Putative aspartic protease; predicted GPI-anchor; member of a YPS gene cluster that is required for virulence in mice; induced in response to low pH and high temperature	YLR120C	Yes	Yes
CAGL0E01749g	Putative aspartic protease; member of a YPS gene cluster that is required for virulence in mice; induced in response to low pH and high temperature	YLR120C	No	Yes
CAGL0E01859g	Putative aspartic protease; predicted GPI-anchor; member of a YPS gene cluster that is required for virulence in mice; induced in response to low pH and high temperature	YLR120C	Yes	Yes
CAGL0E01969g	Ortholog(s) have inorganic cation transmembrane transporter activity, solute:hydrogen symporter activity	YOL122C	No	Yes
CAGL0E02651g	Has domain(s) with predicted identical protein binding, serine-type endopeptidase activity and role in negative regulation of catalytic activity, proteolysis	YOR003W	No	No
CAGL0E03630g	Ortholog(s) have RNA binding activity and role in negative regulation of conjugation with cellular fusion, premeiotic DNA replication, reciprocal meiotic recombination, sporulation resulting in formation of a cellular spore	YHL024W	No	Yes
CAGL0E03740g	No Description Available	YHL026C	Yes	No
CAGL0E04004g	Ortholog(s) have L-methionine transmembrane transporter activity and role in amino acid transport	YHL036W	Yes	No
CAGL0E04548g	Ortholog(s) have mitochondrion localization	YOR020W-A	No	Yes
CAGL0E05148g	Ortholog(s) have alpha-mannosidase activity, role in oligosaccharide catabolic process and cytosol, fungal-type vacuole membrane localization	YGL156W	No	No
CAGL0E05588g	Ortholog(s) have DNA-directed DNA polymerase activity, deoxycytidyl transferase activity and role in error-free translesion synthesis, error-prone translesion synthesis	YOR346W	No	No
CAGL0E05610g	Ortholog(s) have role in cellular response to nitrogen starvation, negative regulation of G0 to G1 transition and cytosol localization	YAL038W	Yes	No
CAGL0E05808g	Bifunctional enzyme of thiamine biosynthesis, with thiamine-phosphate pyrophosphorylase and 4-methyl-5-beta-hydroxyethylthiazole kinase activities; active as a homohexamer	YPL214C	No	No
CAGL0E06072g	Ortholog(s) have cytoplasm localization	YPL229W	No	No
CAGL0E06116g	Has domain(s) with predicted nucleic acid binding activity	YPL230W	No	Yes

CAGL0E06292g	Has domain(s) with predicted GTP binding, GTPase activity, role in GTP catabolic process, protein transport, signal transduction, small GTPase mediated signal transduction and intracellular, membrane localization	YHR022C	No	Yes
CAGL0E06666g	Epithelial adhesion protein; predicted GPI-anchor; adhesin-like protein	YAR050W	No	Yes
CAGL0F00187g	Ortholog(s) have copper ion transmembrane transporter activity, iron ion transmembrane transporter activity	YMR319C	No	No
CAGL0F00605g	Aldohexose specific glucokinase	YDR516C	Yes	Yes
CAGL0F01111g	Ortholog(s) have role in inositol metabolic process and cytosol, nuclear envelope localization	YOL032W	Yes	No
CAGL0F01793g	Delta 5,6 sterol desaturase; C-5 sterol desaturase; predicted transmembrane domain and endolasmic reticulum (ER) binding motif; gene used for molecular typing of C. glabrata strain isolates	YLR056W	No	Yes
CAGL0F01947g	Ortholog(s) have microtubule binding activity	YPL017C	No	Yes
CAGL0F02101g	No Description Available	YFL007W	No	No
CAGL0F02167g	Ortholog(s) have DNA binding activity, role in reciprocal meiotic recombination and nuclear chromosome localization	YFL003C	No	No
CAGL0F02519g	Has domain(s) with predicted DNA binding, sequence-specific DNA binding RNA polymerase II transcription factor activity, zinc ion binding activity and role in regulation of transcription, DNA-dependent, transcription, DNA-dependent	YJL206C	No	Yes
CAGL0F03003g	Ortholog(s) have osmosensor activity and role in (1->3)-beta-D-glucan biosynthetic process, cellular bud site selection, fungal-type cell wall organization, hyperosmotic response, osmosensory signaling pathway via Sho1 osmosensor	YDR420W	Yes	Yes
CAGL0F03267g	Ortholog(s) have mitochondrion, ribosome localization	YHR080C	Yes	Yes
CAGL0F03399g	Ortholog(s) have fatty acid alpha-hydroxylase activity, role in cellular response to cadmium ion, detoxification of cadmium ion, mannosyl-inositol phosphorylceramide metabolic process and endoplasmic reticulum, membrane localization	YMR272C	Yes	Yes
CAGL0F03641g	Has domain(s) with predicted membrane localization	YML018C	No	No
CAGL0F03707g	Ortholog(s) have protein kinase activity, role in cellular ion homeostasis, protein phosphorylation and cytoplasm localization	YOR267C	No	No
CAGL0F04609g	Ortholog(s) have ubiquitin binding activity, role in endocytosis, endoplasmic reticulum unfolded protein response and actin cortical patch, cellular bud neck, cellular bud tip, mating projection tip localization	YBL047C	No	Yes
CAGL0F04631g	No Description Available	YBL049W	No	No
CAGL0F04829g	Ortholog(s) have role in negative regulation of Arp2/3 complex-mediated actin nucleation and actin cortical patch, nucleus localization	YPR154W	Yes	Yes
CAGL0F04895g	Ortholog(s) have glycogen phosphorylase activity, role in glycogen catabolic process, response to heat and cell surface, cytoplasm, hyphal cell wall localization	YPR160W	Yes	Yes
CAGL0F04917g	Putative regulatory subunit for protein phosphatase; gene is upregulated in azole-resistant strain	YOR178C	No	Yes
CAGL0F08041g	Putative phosphofructokinase, alpha subunit; increased protein abundance in azole resistant strain	YGR240C	No	No
CAGL0F08261g	Ortholog(s) have phosphopyruvate hydratase activity and role in glycolytic process, regulation of vacuole fusion, non-autophagic	YHR174W	No	Yes
CAGL0F09273g	Putative adhesin-like protein	-	No	-
CAGL0G00858g	No Description Available	YLR332W	Yes	Yes
CAGL0G01100g	Ortholog(s) have cytosol localization	YLR345W	No	Yes
CAGL0G01474g	Ortholog(s) have role in cellular response to methylmercury and cytoplasm, nucleus localization	YNL042W	Yes	Yes
CAGL0G01738g	Ortholog(s) have lipid binding activity and role in eisosome assembly, endocytosis, negative regulation of protein kinase activity, protein localization, response to heat	YGR086C	Yes	Yes
CAGL0G02101g	Putative omega class glutathione transferase; gene is downregulated in azole-resistant strain	YKR076W	Yes	Yes
CAGL0G02563g	Has domain(s) with predicted ubiquitin thiolesterase activity and role in ubiquitin-dependent protein catabolic process	YKR098C	No	No
CAGL0G02893g	Ortholog(s) have NADH kinase activity, role in NADP biosynthetic process, cellular response to oxidative stress and mitochondrial matrix localization	YPL188W	No	No
CAGL0G02937g	Ortholog(s) have pyruvate decarboxylase activity and role in L-phenylalanine catabolic process, aromatic amino acid family catabolic process to alcohol via Ehrlich pathway, ethanol metabolic process, tryptophan catabolic process	YGR087C	No	Yes
CAGL0G03179g	Has domain(s) with predicted phospholipid binding activity	YGR097W	No	Yes
CAGL0G03267g	Ortholog(s) have role in protein targeting to membrane and cytoplasm localization	YER101C	No	Yes
CAGL0G03531g	Ortholog(s) have mitochondrion localization	YER115C	No	No

CAGL0G03795g	Heat shock protein of the HSP70 family	YLL024C	No	No
CAGL0G04081g	Ortholog(s) have Golgi apparatus, cell division site, cell tip, endoplasmic reticulum localization	YLR004C	Yes	Yes
CAGL0G05093g	Ortholog(s) have mitochondrion localization	YDR061W	No	No
CAGL0G05269g	Putative mitochondrial protein; gene is downregulated in azole-resistant strain	YDR070C	No	No
CAGL0G05632g	Ortholog(s) have cytoplasm localization	YDL218W	No	No
CAGL0G05698g	Ortholog(s) have glutamate dehydrogenase (NAD+) activity, role in nitrogen compound metabolic process and cytosol, mitochondrion localization	YDL215C	No	Yes
CAGL0G05962g	Ortholog(s) have endoplasmic reticulum localization	YHR140W	Yes	Yes
CAGL0G06050g	No Description Available	-	No	-
CAGL0G06182g	No Description Available	YHR131C	No	No
CAGL0G06798g	No Description Available	YJR005C-A	No	No
CAGL0G08624g	Drug:H+ antiporter of the Major Facilitator Superfamily, confers imidazole drug resistance, involved in quinidine/multidrug efflux; gene is activated by Pdr1p; upregulated in azole-resistant strain	YIL120W	Yes	No
CAGL0G08866g	Ortholog(s) have DNA replication origin binding, RNA polymerase II transcription factor binding, RNA polymerase II transcription factor binding transcription factor activity	YNL068C	Yes	No
CAGL0G08888g	Ortholog(s) have role in maintenance of rDNA, meiotic chromosome segregation, recombinational repair and Shu complex, cytoplasm, nucleus localization	YIL132C	No	No
CAGL0G09295g	Ortholog(s) have cytoplasm localization	YGR196C	No	No
CAGL0G09515g	Ortholog(s) have glucan exo-1,3-beta-glucosidase activity, role in ascospore formation and ascospore wall localization	YOR190W	No	Yes
CAGL0G09603g	Putative protein; gene is upregulated in azole-resistant strain	-	No	-
CAGL0G09977g	Ortholog(s) have 4-alpha-glucanotransferase activity, amylo-alpha-1,6-glucosidase activity, role in glycogen catabolic process and mitochondrion localization	YPR184W	Yes	Yes
CAGL0G10219g	Adhesin-like protein with 5 tandem repeats; predicted GPI-anchor; similarity to S. cerevisiae flocculins, cell wall proteins that mediate adhesion	-	Yes	-
CAGL0H00110g	Adhesin-like protein with internal repeats; predicted GPI-anchor; likely a C-terminal fragment of a single ORF with CAGL0H00132g	-	Yes	-
CAGL0H00418g	No Description Available	YER175C	No	Yes
CAGL0H00704g	Ortholog(s) have fungal-type vacuole membrane localization	YMR195W	Yes	Yes
CAGL0H01177g	Ortholog(s) have diacylglycerol diphosphate phosphatase activity, phosphatidate phosphatase activity and role in farnesol biosynthetic process, phospholipid metabolic process	YDR284C	No	No
CAGL0H01287g	Ortholog(s) have mRNA 5'-UTR binding, translation repressor activity, nucleic acid binding activity	YDR293C	Yes	Yes
CAGL0H02519g	Ortholog(s) have cytoplasm localization	YMR253C	Yes	No
CAGL0H02541g	Ortholog(s) have mitochondrion localization	YMR252C	No	No
CAGL0H02563g	Predicted GPI-linked protein	YOL052C-A	Yes	No
CAGL0H02695g	Ortholog(s) have glycogenin glucosyltransferase activity and role in glycogen biosynthetic process	YKR058W	Yes	No
CAGL0H02893g	Ortholog(s) have mitochondrion localization	YJL070C	No	No
CAGL0H03707g	Ortholog(s) have misfolded protein binding activity and role in misfolded protein transport, nucleus-associated proteasomal ubiquitin-dependent protein catabolic process, protein folding, translational initiation	YNL007C	Yes	No
CAGL0H03971g	Ortholog(s) have membrane raft, mitochondrion, plasma membrane localization	YCR004C	No	Yes
CAGL0H04037g	Ortholog(s) have heat shock protein binding activity, role in glycogen metabolic process, meiosis, mitotic spindle assembly checkpoint, response to heat and protein phosphatase type 1 complex localization	YOR178C	No	Yes
CAGL0H04213g	Ortholog(s) have sequence-specific DNA binding activity and nucleus localization	YML081W	No	No
CAGL0H04279g	Copper-binding metallothionein, involved in sequestration of metal ions; inducible by copper and silver; present in multiple (3-9) tandemly arranged copies in various strains	-	Yes	-
CAGL0H04851g	Ortholog(s) have 4-nitrophenylphosphatase activity, protein serine/threonine phosphatase activity and role in cellular protein localization, cellular sodium ion homeostasis, protein dephosphorylation	YML016C	No	No
CAGL0H07469g	Putative adhesin-like protein	YBR157C	No	No
CAGL0H07997g	Protein involved in cell wall beta 1,6-glucan synthesis, similar to Kre9p,	YDL049C	No	Yes
CAGL0H08195g	Ortholog(s) have ATPase inhibitor activity, Hsp70 protein binding, Hsp90 protein binding, mRNA binding activity, role in protein folding and cytosol, nucleus localization	YOR027W	Yes	No
CAGL0H08261g	No Description Available	YOR019W	Yes	Yes
CAGL0H08305g	No Description Available	YDR051C	No	No

CAGL0H08393g	Has domain(s) with predicted amino acid transmembrane transporter activity, role in amino acid transmembrane transport, amino acid transport, transmembrane transport and integral component of membrane, membrane localization	YDR046C	Yes	No
CAGL0H08778g	Ortholog(s) have mRNA binding activity, role in nuclear-transcribed mRNA catabolic process, deadenylation-dependent decay and cytoplasm localization	YPR042C	No	Yes
CAGL0H08888g	Ortholog(s) have FAD transmembrane transporter activity, role in FAD transport, fungal-type cell wall biogenesis, protein folding and cellular bud neck, endoplasmic reticulum, fungal-type vacuole localization	YPL221W	Yes	Yes
CAGL0H09592g	Putative GPI-linked cell wall protein	YER011W	Yes	No
CAGL0H09944g	Ortholog(s) have 2-alkenal reductase [NAD(P)] activity, AU-rich element binding, NADPH:quinone reductase activity, role in cellular response to oxidative stress and cytosol, nucleus localization	YBR046C	No	No
CAGL0H09966g	Ortholog(s) have mitochondrion localization	YBR047W	No	Yes
CAGL0H10054g	No Description Available	YBR053C	Yes	Yes
CAGL0H10120g	Ortholog(s) have glucan endo-1,6-beta-glucosidase activity and cytosol, mitochondrial intermembrane space, nucleus localization	YBR056W	No	No
CAGL0H10164g	Ortholog(s) have role in premeiotic DNA replication and cytoplasm localization	YBR057C	No	No
CAGL0H10626g	Predicted cell wall adhesin with a predicted role in adhesion; predicted GPI anchor; contains tandem repeats	-	No	-
CAGL0I00418g	Ortholog(s) have electron carrier activity, stearoyl-CoA 9-desaturase activity	YGL055W	No	No
CAGL0I01276g	Ortholog(s) have cytoplasm, nucleus localization	YHR112C	No	Yes
CAGL0I01342g	Ortholog(s) have L-allo-threonine aldolase activity, role in carnitine biosynthetic process, glycine biosynthetic process, threonine catabolic process and cytosol, nucleus localization	YEL046C	No	Yes
CAGL0I01980g	Putative activator of transcription; gene is upregulated in azole-resistant strain	YNL257C	Yes	No
CAGL0I02530g	Ortholog(s) have N,N-dimethylaniline monooxygenase activity, role in protein folding and endoplasmic reticulum membrane, mitochondrion localization	YHR176W	No	No
CAGL0I03168g	No Description Available	YEL023C	No	No
CAGL0I03872g	Has domain(s) with predicted substrate-specific transmembrane transporter activity, transmembrane transporter activity, role in transmembrane transport and integral to membrane, membrane localization	YDL138W	No	No
CAGL0I04180g	Metal-activated transcription factor; binds promoters of metallothionein genes; autoregulates its own expression; gene is downregulated in azole-resistant strain	YGL166W	No	Yes
CAGL0I04246g	Putative transcription factor involved in sterol uptake; gene is upregulated in azole-resistant strain	YPR009W	No	No
CAGL0I05060g	Has domain(s) with predicted DNA binding, chromatin binding activity	YER088C	No	Yes
CAGL0I05082g	Ortholog(s) have role in DNA damage checkpoint, G2/M transition of mitotic cell cycle, cellular response to biotic stimulus and cellular response to starvation, more	YBL051C	No	No
CAGL0I05148g	D-lactate ferricytochrome C oxidoreductase	YDL174C	No	No
CAGL0I05522g	Has domain(s) with predicted ubiquitin thiolesterase activity and role in ubiquitin-dependent protein catabolic process	YER098W	Yes	Yes
CAGL0I05698g	Ortholog(s) have 6-phosphofructokinase activity, mRNA binding activity, role in glycolysis, proton transport and 6-phosphofructokinase complex, mitochondrion localization	YMR205C	Yes	Yes
CAGL0I05934g	Ortholog(s) have role in cellular response to water deprivation and cytoplasm localization	YJL144W	Yes	No
CAGL0I06182g	Pir protein family member, predicted GPI-anchor	YKL164C	No	Yes
CAGL0I07227g	Putative isocitrate dehydrogenase	YOR136W	Yes	Yes
CAGL0I07249g	Putative GTPase-activating protein involved in cell wall and cytoskeleton homeostasis; gene is upregulated in azole-resistant strain	YOR134W	No	Yes
CAGL0I07843g	Putative alcohol dehydrogenase isoenzyme III; increased protein abundance in azole resistant strain	YOL086C	Yes	Yes
CAGL0I07887g	Ortholog(s) have protein binding, bridging activity and role in CVT pathway, ER-associated protein catabolic process, protein complex localization, protein processing, vesicle organization	YOL082W	No	Yes
CAGL0I08305g	Ortholog(s) have carnitine O-acetyltransferase activity	YER024W	Yes	No
CAGL0I08613g	Putative plasma membrane polyamine transporter	YHL016C	No	Yes
CAGL0I09009g	Ortholog(s) have histidinol-phosphatase activity, role in histidine biosynthetic process and cytosol, nucleus localization	YFR025C	No	No
CAGL0I09086g	Ortholog(s) have endoplasmic reticulum, mitochondrion localization	YNL125C	Yes	Yes
CAGL0I09108g	Has domain(s) with predicted role in transmembrane transport and integral to membrane localization	YNL125C	Yes	Yes

CAGL0I09724g	Has domain(s) with predicted role in transmembrane transport and integral component of membrane localization	YOR306C	No	No
CAGL0I10010g	Ortholog(s) have chaperone binding activity, role in cellular response to heat, intracellular protein transport, protein folding, protein localization to nucleus and nucleus localization	YPR158W	Yes	No
CAGL0I10626g	Ortholog(s) have Golgi apparatus, endoplasmic reticulum, fungal-type vacuole membrane localization	YGR125W	No	No
CAGL0I10648g	Putative asparagine synthetase; protein abundance increased in ace2 mutant cells	YPR145W	No	No
CAGL0I10747g	Ortholog(s) have ammonium transmembrane transporter activity, role in ammonium transport, nitrogen utilization and plasma membrane localization	YPR138C	Yes	Yes
CAGL0J00451g	Putative glyceraldehyde-3-phosphate dehydrogenase; protein differentially expressed in azole resistant strain; expression downregulated in biofilm vs planktonic cell culture	YGR192C	No	Yes
CAGL0J00715g	Has domain(s) with predicted GTP binding, GTPase activity, role in GTP catabolic process, protein transport, signal transduction, small GTPase mediated signal transduction and intracellular, membrane localization	YHR022C	No	Yes
CAGL0J00803g	Ortholog(s) have structural constituent of cytoskeleton activity	YJL042W	Yes	No
CAGL0J01529g	Ortholog(s) have role in histone deacetylation, negative regulation of antisense RNA transcription, regulation of DNA-dependent DNA replication initiation, transcription elongation from RNA polymerase II promoter	YMR075W	No	No
CAGL0J01699g	No Description Available	YPR010C-A	No	Yes
CAGL0J01892g	Ortholog(s) have protein binding, bridging activity and role in actin cortical patch assembly, axial cellular bud site selection, bipolar cellular bud site selection, endocytosis, unidimensional cell growth	YIR006C	No	No
CAGL0J02948g	Purine-cytosine transporter	YER060W-A	Yes	No
CAGL0J03256g	Ortholog(s) have protein tyrosine phosphatase activity	YER075C	No	Yes
CAGL0J04026g	No Description Available	YOR227W	No	No
CAGL0J04466g	Ortholog(s) have role in fungal-type cell wall organization, protein oligomerization and Golgi apparatus, cell cortex of cell tip, cell division site, cellular bud, membrane raft, plasma membrane localization	YLR414C	No	No
CAGL0J05060g	Ortholog(s) have RNA polymerase II regulatory region sequence-specific DNA binding, sequence-specific DNA binding RNA polymerase II transcription factor activity, zinc ion binding activity	YJL056C	Yes	Yes
CAGL0J06028g	Ortholog(s) have high affinity secondary active ammonium transmembrane transporter activity, methylammonium transmembrane transporter activity	YNL142W	No	Yes
CAGL0J07502g	Putative protein similar to globins with a heme-binding domain; gene is upregulated in azole-resistant strain	YNL234W	No	No
CAGL0J08613g	Ortholog(s) have calcium activated cation channel activity, calcium channel activity, potassium channel activity, sodium channel activity, voltage-gated ion channel activity and role in cellular cation homeostasis	YOR087W	No	Yes
CAGL0J09262g	Ortholog(s) have role in negative regulation of ATPase activity and mitochondrion localization	YDL130W-A	No	Yes
CAGL0J10296g	Ortholog(s) have ATPase activator activity, unfolded protein binding activity, role in protein sumoylation and mitochondrion, nucleus localization	YNL077W	Yes	No
CAGL0J11176g	Putative adhesin-like protein	YNL176C	Yes	No
CAGL0J11308g	Has domain(s) with predicted ATP binding, protein kinase activity, protein tyrosine kinase activity, transferase activity, transferring phosphorus-containing groups activity and role in protein phosphorylation	YNL183C	No	No
CAGL0J11462g	Predicted GPI-linked cell wall protein	YNL190W	Yes	No
CAGL0J11770g	Putative phospholipase B; predicted GPI-anchor	YOL011W	Yes	Yes
CAGL0K01133g	Ortholog(s) have actin monomer binding activity, role in sequestering of actin monomers and cell division site, cell tip, mating projection tip localization	YGR080W	No	No
CAGL0K01155g	No Description Available	YGR079W	No	No
CAGL0K01683g	Putative cytoplasmic glycerol-3-phosphate dehydrogenase (NAD+); protein abundance increased in ace2 mutant cells	YOL059W	No	Yes
CAGL0K01705g	Ortholog(s) have cytoplasm localization	YOL056W	No	No
CAGL0K02101g	Ortholog(s) have role in TOR signaling cascade, cellular response to chemical stimulus, cellular response to heat, cellular response to pH and chlamydospore formation, more	YGL197W	No	Yes
CAGL0K02651g	Ortholog(s) have role in chloride transport and fungal-type vacuole localization	YHL008C	Yes	Yes
CAGL0K04257g	Ortholog(s) have ATPase activity, coupled to transmembrane movement of ions, RNA polymerase II core promoter proximal region sequence-specific DNA binding, sequence-specific DNA binding RNA polymerase II transcription factor activity	YCL005W-A	Yes	No

CAGL0K04367g	Has domain(s) with predicted amino acid transmembrane transporter activity, role in amino acid transmembrane transport and membrane localization	YGR055W	Yes	Yes
CAGL0K04675g	No Description Available	-	No	-
CAGL0K04719g	Ortholog(s) have mitochondrion, ribosome localization	YNL208W	No	Yes
CAGL0K05665g	Has domain(s) with predicted catalytic activity, pyridoxal phosphate binding activity	YHR112C	No	Yes
CAGL0K06853g	Ortholog(s) have mRNA binding activity, role in cellular response to cadmium ion, detoxification of cadmium ion and cytosol, peroxisomal matrix, peroxisomal membrane localization	YBR222C	No	Yes
CAGL0K07007g	Protein of unknown function	YBR238C	No	Yes
CAGL0K07183g	No Description Available	-	No	-
CAGL0K07205g	No Description Available	YGL117W	No	No
CAGL0K07458g	Has domain(s) with predicted ATP binding, protein kinase activity, protein serine/threonine kinase activity, protein tyrosine kinase activity, transferase activity, transferring phosphorus-containing groups activity	YKL126W	Yes	No
CAGL0K07546g	Putative phosphate starvation inducible acid phosphatase; contains a phosphomutase-like domain; functionally complements a S. cerevisiae pho5 mutant; transcript abundance during phosphate starvation regulated by Pho4p	YKL128C	No	Yes
CAGL0K07590g	Putative myosin	YMR109W	No	No
CAGL0K07634g	Ortholog(s) have RNA polymerase II core promoter proximal region sequence-specific DNA binding transcription factor activity involved in positive regulation of transcription, more	YFL021W	No	Yes
CAGL0K07678g	No Description Available	-	No	-
CAGL0K08624g	Ortholog(s) have RNA polymerase II activating transcription factor binding, more	YKL109W	Yes	Yes
CAGL0K09372g	Ortholog(s) have RNA polymerase II core promoter proximal region sequence-specific DNA binding, more	YGL209W	Yes	Yes
CAGL0K10164g	Predicted GPI-linked protein; putative adhesin-like protein	YER150W	Yes	No
CAGL0K10428g	No Description Available	YOL024W	No	No
CAGL0K10824g	No Description Available	YLR149C	Yes	No
CAGL0K11297g	Ortholog(s) have gluconokinase activity, role in D-gluconate metabolic process and cytosol, nucleus localization	YDR248C	No	No
CAGL0K12100g	Putative coproporphyrinogen III oxidase; protein differentially expressed in azole resistant strain	YDR044W	Yes	No
CAGL0K12144g	Putative Hsp70p nucleotide exchange factor; protein abundance decreased in ace2 mutant cells	YBR101C	No	No
CAGL0K12716g	Has domain(s) with predicted substrate-specific transmembrane transporter activity, transmembrane transporter activity, role in transmembrane transport and integral to membrane, membrane localization	YFL040W	No	No
CAGL0K12760g	Ortholog(s) have cytoplasm localization	YFL042C	Yes	Yes
CAGL0L00583g	Ortholog(s) have sequence-specific DNA binding, sequence-specific DNA binding transcription factor activity	YPL230W	No	Yes
CAGL0L00957g	No Description Available	YER048C	Yes	No
CAGL0L01287g	Haloacid dehalogenase-like hydrolase	YEL038W	No	Yes
CAGL0L02453g	No Description Available	YEL007W	No	Yes
CAGL0L02497g	Fructose-bisphosphate aldolase; expression downregulated in biofilm vs planktonic cell culture; protein abundance increased in ace2 mutant cells	YKL060C	Yes	No
CAGL0L03135g	Putative phospholipase D; gene is upregulated in azole-resistant strain	YKR031C	Yes	No
CAGL0L03157g	Ortholog(s) have sequence-specific DNA binding, sequence-specific DNA binding RNA polymerase II transcription factor activity	YKR034W	Yes	No
CAGL0L03267g	Ortholog(s) have L-proline transmembrane transporter activity, polyamine transmembrane transporter activity and role in amino acid transport, polyamine transport, regulation of nitrogen utilization	YKR039W	Yes	No
CAGL0L03696g	Has domain(s) with predicted role in transmembrane transport and integral to membrane localization	YOR092W	No	No
CAGL0L03916g	Has domain(s) with predicted nucleic acid binding activity	YOR113W	No	No
CAGL0L04598g	Ortholog(s) have RNA 7-methylguanosine cap binding, m7G(5')pppN diphosphatase activity, role in deadenylation-dependent decapping of nuclear-transcribed mRNA and cytoplasmic mRNA processing body, cytosol, nucleus localization	YOR173W	Yes	No
CAGL0L05016g	Ortholog(s) have Sin3-type complex localization	YMR053C	No	No
CAGL0L05236g	Malate dehydrogenase	YKL085W	Yes	No
CAGL0L05654g	Ortholog(s) have chloride transmembrane transporter activity, potassium ion transmembrane transporter activity	YKR050W	Yes	No

CAGL0L05786g	Ortholog(s) have sequence-specific DNA binding activity	YPR013C	No	No
CAGL0L06006g	Ortholog(s) have protein serine/threonine kinase activity	YGL180W	No	No
CAGL0L06248g	Ortholog(s) have cytoplasm, nucleus localization	YBR085C-A	Yes	Yes
CAGL0L06424g	Predicted GPI-linked adhesin-like protein	YLR110C	Yes	No
CAGL0L06864g	Putative protein related to ECM3; gene is downregulated in azole-resistant strain	YMR140W	No	No
CAGL0L07722g	Putative 3-phosphoglycerate kinase; protein differentially expressed in azole resistant strain; protein abundance increased in ace2 mutant cells	YCR012W	Yes	No
CAGL0L07744g	Ortholog(s) have endoplasmic reticulum localization	YCR011C	Yes	No
CAGL0L07810g	Ortholog(s) have protein kinase activity	YCR008W	No	No
CAGL0L08008g	No Description Available	YEL017C-A	No	Yes
CAGL0L08030g	Ortholog(s) have asparagine-tRNA ligase activity, role in mitochondrial asparaginyl-tRNA aminoacylation and mitochondrion localization	YCR024C	No	No
CAGL0L08074g	No Description Available	YCR030C	No	Yes
CAGL0L09251g	Ortholog(s) have role in positive regulation of transcription from RNA polymerase II promoter, response to salt stress and cytoplasm localization	YPR005C	Yes	No
CAGL0L09273g	Ortholog(s) have methylisocitrate lyase activity, role in propionate catabolic process, 2-methylcitrate cycle and mitochondrial matrix localization	YPR006C	No	Yes
CAGL0L09691g	Ortholog(s) have RNA polymerase II core promoter proximal region sequence-specific DNA binding, more	YKL015W	Yes	No
CAGL0L09933g	No Description Available	YOR042W	No	No
CAGL0L10142g	Putative sphingolipid flippase; gene is upregulated in azole-resistant strain	YOR049C	Yes	No
CAGL0L10582g	Ortholog(s) have cytoplasm localization	YMR196W	Yes	Yes
CAGL0L10758g	Putative 6-phosphofructokinase, beta subunit; protein abundance increased in ace2 mutant cells	YMR205C	No	Yes
CAGL0L10912g	Ortholog(s) have spermidine transmembrane transporter activity, spermine transmembrane transporter activity, role in spermidine transport, spermine transport and fungal-type vacuole membrane, plasma membrane localization	YOR273C	No	Yes
CAGL0L11154g	Ortholog(s) have lysophospholipase activity, role in phosphatidylcholine catabolic process, regulation of phospholipid biosynthetic process and endoplasmic reticulum localization	YML059C	No	No
CAGL0L11440g	Ortholog(s) have lipid binding activity and role in endoplasmic reticulum membrane organization, regulation of phosphatidylinositol dephosphorylation	YML072C	No	No
CAGL0L11990g	Ortholog(s) have disulfide oxidoreductase activity, role in actin cytoskeleton organization, cellular iron ion homeostasis, cellular response to oxidative stress and nucleus localization	YER174C	Yes	No
CAGL0L12012g	Ortholog(s) have trans-aconitate 3-methyltransferase activity and cytosol localization	YER175C	No	Yes
CAGL0L12056g	No Description Available	YER177W	No	Yes
CAGL0L12892g	Ortholog(s) have role in actin cytoskeleton organization, inositol lipid-mediated signaling, vacuole organization and plasma membrane localization	YKL051W	No	No
CAGL0M00550g	Ortholog(s) have cytosol, nucleus localization	YJR130C	No	No
CAGL0M00748g	Putative integral membrane protein required for high-affinity Ca2+ influx	YLR443W	No	No
CAGL0M01166g	Ortholog(s) have ferrous iron binding activity, role in mitochondrial genome maintenance, thiamine biosynthetic process, thiazole biosynthetic process and cytosol, nucleus localization	YGR144W	No	Yes
CAGL0M02167g	No Description Available	YPL156C	Yes	Yes
CAGL0M02915g	Ortholog(s) have role in peroxisome degradation and peroxisome localization	YJL185C	No	No
CAGL0M03135g	Ortholog(s) have cell division site, cell tip, cytosol localization	YJL084C	No	No
CAGL0M03157g	Ortholog(s) have role in autophagy, cellular response to starvation, chromatin silencing at rDNA, fungal-type cell wall organization, inositol lipid-mediated signaling and mitochondrion, pre-autophagosomal structure localization	YKR019C	No	No
CAGL0M03179g	Ortholog(s) have cytosol, nucleus localization	YJL082W	No	No
CAGL0M03245g	Ortholog(s) have cytosol localization	YEL020C	No	No
CAGL0M04807g	Component of the chromatin remodelling Swi/Snf complex; involved in regulation of biofilm formation	YOR290C	No	Yes
CAGL0M06347g	Ortholog(s) have dihydroceramidase activity, role in ceramide biosynthetic process, ceramide catabolic process and endoplasmic reticulum, fungal-type vacuole membrane localization	YPL087W	Yes	Yes
CAGL0M06765g	Ortholog(s) have role in actin filament organization, endocytosis, response to stress and cytosol, nucleus localization	YNL032W	No	No
CAGL0M06963g	Ortholog(s) have role in tRNA export from nucleus and cytoplasm, nucleus localization	YNR034W	Yes	No
CAGL0M07568g	Ortholog(s) have ubiquitin-protein ligase activity	YMR022W	No	No
CAGL0M07612g	Ortholog(s) have polyamine oxidase activity, role in pantothenate biosynthetic process, polyamine catabolic process and cytoplasm localization	YMR020W	Yes	No
CAGL0M08492g	Pir protein family member, predicted GPI-anchor	YKL164C	No	Yes

CAGL0M08800g	bZIP domain-containing protein	YOR028C	No	Yes
CAGL0M08822g	Ortholog(s) have ATPase activity, misfolded protein binding activity, role in cellular response to heat, mitochondrial genome maintenance, protein refolding, protein stabilization, protein unfolding and mitochondrial matrix localization	YDR258C	Yes	No
CAGL0M09108g	Has domain(s) with predicted RNA binding, nucleic acid binding, nucleotide binding activity	YPR042C	No	Yes
CAGL0M09449g	No Description Available	YBL101C	No	No
CAGL0M10153g	Ortholog(s) have MAP kinase kinase kinase kinase activity, histone serine kinase activity	YHL007C	No	No
CAGL0M10439g	Ortholog(s) have alpha,alpha-trehalase activity, calcium ion binding activity	YDR001C	No	Yes
CAGL0M10571g	Ortholog(s) have ergosterol O-acyltransferase activity, role in ergosterol metabolic process and endoplasmic reticulum localization	YNR019W	No	Yes
CAGL0M11000g	No Description Available	YCR075W-A	Yes	No
CAGL0M12947g	Mitochondria-localized protein; gene is upregulated in azole-resistant strain	YIL077C	No	No
CAGL0M13651g	Ortholog(s) have serine-type carboxypeptidase activity, role in phytochelatin biosynthetic process and endoplasmic reticulum, extracellular region, fungal-type vacuole localization	YMR297W	No	Yes

Acetic acid-responsive genes regulated by ScHaa1 and CgHaa1

ORF	Function	*S. cerevisiae* orthologue	*C. glabrata* promoter harbors an HRE motif	*S. cerevisiae* promoter harbors an HRE motif
CAGL0C02321g	Ortholog(s) have nucleotidase activity and role in pyrimidine nucleobase metabolic process	YGL224C	No	No
CAGL0C02893g	Ortholog(s) have protein serine/threonine kinase activity	YOR267C	No	No
CAGL0C05071g	Ortholog(s) have hydroxymethylpyrimidine kinase activity, phosphomethylpyrimidine kinase activity, thiaminase activity, role in thiamine biosynthetic process, thiamine catabolic process and cytosol localization	YOL055C	No	Yes
CAGL0D03322g	Ortholog(s) have role in cellular zinc ion homeostasis and endoplasmic reticulum localization	YLR023C	Yes	No
CAGL0E00803g	Putative small cytosolic stress-induced chaperone; gene is upregulated in azole-resistant strain	YDR171W	Yes	Yes
CAGL0E01529g	Ortholog(s) have role in fructose 2,6-bisphosphate metabolic process, regulation of glycolysis	YOL136C	Yes	No
CAGL0E02035g	Ortholog(s) have fungal-type vacuole membrane localization	YOL119C	Yes	Yes
CAGL0E02321g	Putative phospholipase B; predicted GPI-anchor	YOL011W	No	Yes
CAGL0E03025g	No Description Available	YGR146C	Yes	No
CAGL0E04774g	Ortholog(s) have cytoplasm localization	YLR225C	Yes	Yes
CAGL0E05566g	Has domain(s) with predicted protein dimerization activity	YOR344C	No	No
CAGL0E06380g	Ortholog(s) have ATP-dependent NAD(P)H-hydrate dehydratase activity, role in nicotinamide nucleotide metabolic process and cytosol localization	YKL151C	No	Yes
CAGL0F04521g	No Description Available	YBL043W	No	Yes
CAGL0F05687g	Ortholog(s) have cytoplasm, ribosome localization	YDR186C	No	Yes
CAGL0F07117g	Putative subunit of the heterotrimeric G protein; gene is upregulated in azole-resistant strain	YGL121C	No	No
CAGL0F08217g	Ortholog(s) have cytoplasmic stress granule localization	YGR250C	No	No
CAGL0F08745g	Ortholog(s) have cytoplasm, nucleus, ribosome localization	YLR327C	Yes	Yes
CAGL0F09207g	Has domain(s) with predicted branched-chain-amino-acid transaminase activity, catalytic activity and role in branched-chain amino acid metabolic process, metabolic process	YJR148W	No	No
CAGL0L10362g	Ortholog(s) have cytoplasm, nucleus localization	YKR075C	No	Yes
CAGL0G03289g	Heat shock protein of the HSP70 family	YER103W	Yes	No
CAGL0G03883g	Ortholog(s) have ADP binding, ATP binding, ATPase activity, coupled, chaperone binding, misfolded protein binding, unfolded protein binding activity	YLL026W	No	Yes
CAGL0G05357g	Ortholog(s) have NADHX epimerase activity, role in nicotinamide nucleotide metabolic process and cytosol, mitochondrion, nucleus localization	YNL200C	No	No
CAGL0G08844g	Ortholog(s) have sequence-specific DNA binding activity	YIL130W	No	No
CAGL0H01375g	Predicted sphinganine hydroxylase with role in sphingolipid biosynthesis; mutants show reduced sensitivity to caspofungin and increased sensitivity to micafungin	YDR297W	Yes	No
CAGL0H02101g	Ortholog(s) have role in RNA metabolic process and cytoplasm, nucleus localization	YHR087W	No	No
CAGL0H02387g	Putative trehalose-6-phosphate synthase/phosphatase subunit; gene is upregulated in azole-resistant strain	YML100W	Yes	Yes
CAGL0H02585g	Ortholog(s) have glutamate decarboxylase activity, role in cellular response to oxidative stress, glutamate catabolic process and cytoplasm localization	YMR250W	Yes	No
CAGL0H05379g	Ortholog(s) have RNA polymerase II core promoter proximal region sequence-specific DNA binding, more	YPL075W	No	Yes

CAGL0H10076g	Has domain(s) with predicted ion channel activity, role in ion transport and membrane localization	YBR054W	No	No
CAGL0I00550g	Ortholog(s) have fungal-type vacuole membrane, nucleus localization	YLR297W	No	Yes
CAGL0I06644g	Putative GPI-linked cell wall protein	YER150W	No	No
CAGL0I09702g	Ortholog(s) have riboflavin transporter activity, role in riboflavin transport and plasma membrane localization	YOR306C	No	No
CAGL0I10384g	Predicted polyamine transporter of the major facilitator superfamily; required for azole resistance	YPR156C	No	Yes
CAGL0J03080g	No Description Available	YER067W	Yes	Yes
CAGL0J06050g	Has domain(s) with predicted role in cellular amino acid metabolic process	YNL160W	Yes	Yes
CAGL0J07876g	Ortholog(s) have cytoplasm, nucleus localization	YNL254C	No	No
CAGL0J08074g	Ortholog(s) have phosphatidylinositol transporter activity	YNL264C	No	No
CAGL0K02145g	Has domain(s) with predicted nucleic acid binding activity	YER130C	Yes	Yes
CAGL0K03421g	Ortholog(s) have cytosol, nucleus localization	YMR105C	No	Yes
CAGL0K04301g	Putative mitochondrial Ser/Thr protein kinase; gene is upregulated in azole-resistant strain	YGR052W	No	No
CAGL0K05357g	Ortholog(s) have glutamate-ammonia ligase activity, role in cellular response to nitrogen starvation, glutamine biosynthetic process, nitrogen utilization and cytosol, nucleus localization	YPR035W	Yes	Yes
CAGL0K07337g	Has domain(s) with predicted ion channel activity, role in ion transport and membrane localization	YCR021C	No	Yes
CAGL0K12078g	Has domain(s) with predicted nucleic acid binding activity	YDR043C	No	Yes
CAGL0L08712g	Ortholog(s) have cytoplasm, nucleus localization	YPL014W	No	Yes
CAGL0L09108g	Ortholog(s) have role in propionate metabolic process and mitochondrial outer membrane localization	YPR002W	No	No
CAGL0L10043g	Ortholog(s) have protein kinase activator activity	YDR277C	Yes	Yes
CAGL0M04763g	Ortholog(s) have role in cellular response to drug and cytosol, nucleus localization	YOR289W	No	No
CAGL0M06897g	Ortholog(s) have cytoplasm localization	YNL024C	Yes	No
CAGL0M07293g	Putative ABC transporter of weak organic acids; gene is downregulated in azole-resistant strain	YPL058C	No	Yes
CAGL0M07634g	Ortholog(s) have sequence-specific DNA binding, sequence-specific DNA binding transcription factor activity	YMR016C	No	No
CAGL0M08552g	Ortholog(s) have role in cation transport, regulation of membrane potential and plasma membrane localization	YDR276C	Yes	No
